# Neolithic millet farmers contributed to the permanent settlement of the Tibetan Plateau by adopting barley agriculture

**DOI:** 10.1093/nsr/nwz080

**Published:** 2019-06-21

**Authors:** Yu-Chun Li, Jiao-Yang Tian, Feng-Wen Liu, Bin-Yu Yang, Kang-Shu-Yun Gu, Zia Ur Rahman, Li-Qin Yang, Fa-Hu Chen, Guang-Hui Dong, Qing-Peng Kong

**Affiliations:** 1 State Key Laboratory of Genetic Resources and Evolution/Key Laboratory of Healthy Aging Research of Yunnan Province, Kunming Institute of Zoology, Chinese Academy of Sciences, Kunming 650223, China; 2 Key Laboratory of Western China's Environmental Systems (Ministry of Education), Lanzhou University, Lanzhou 730000, China; 3 CAS Center for Excellence in Animal Evolution and Genetics, Chinese Academy of Sciences, Kunming 650223, China; 4 Kunming Key Laboratory of Healthy Aging Study, Kunming 650223, China; 5 KIZ/CUHK Joint Laboratory of Bioresources and Molecular Research in Common Diseases, Kunming 650223, China; 6 Kunming College of Life Science, University of Chinese Academy of Sciences, Kunming 650204, China; 7 CAS Center for Excellence in Tibetan Plateau Earth Sciences and Key Laboratory of Alpine Ecology, Institute of Tibetan Plateau Research (ITPCAS), Chinese Academy of Sciences, Beijing 100101, China

**Keywords:** Tibetans, millet farmers, barley agriculture, archaeology, mitochondrial genomes

## Abstract

The permanent human settlement of the Tibetan Plateau (TP) has been suggested to have been facilitated by the introduction of barley agriculture ∼3.6 kilo-years ago (ka). However, how barley agriculture spread onto the TP remains unknown. Given that the lower altitudes in the northeastern TP were occupied by millet cultivators from 5.2 ka, who also adopted barley farming ∼4 ka, it is highly possible that it was millet farmers who brought barley agriculture onto the TP ∼3.6 ka. To test this hypothesis, we analyzed mitochondrial DNA (mtDNA) from 8277 Tibetans and 58 514 individuals from surrounding populations, including 682 newly sequenced whole mitogenomes. Multiple lines of evidence, together with radiocarbon dating of cereal remains at different elevations, supports the scenario that two haplogroups (M9a1a1c1b1a and A11a1a), which are common in contemporary Tibetans (20.9%) and were probably even more common (40–50%) in early Tibetans prior to historical immigrations to the TP, represent the genetic legacy of the Neolithic millet farmers. Both haplogroups originated in northern China between 10.0–6.0 ka and differentiated in the ancestors of modern Tibetans ∼5.2–4.0 ka, matching the dispersal history of millet farming. By showing that substantial genetic components in contemporary Tibetans can trace their ancestry back to the Neolithic millet farmers, our study reveals that millet farmers adopted and brought barley agriculture to the TP ∼3.6–3.3 ka, and made an important contribution to the Tibetan gene pool.

## INTRODUCTION

With an extreme and harsh environment that is generally considered inhospitable to humans, the Tibetan Plateau (TP) has been inhabited since the late upper Pleistocene [1–3]. In contrast to the earliest occupation by hunter gatherers during the Late Paleolithic era, whose contribution to the current Tibetan gene pool was rather limited [1], migration onto the plateau appears to have occurred during the Neolithic period [1,4–7]. Archaeological evidence further suggests that permanent human occupation began ∼3.6 kilo-years ago (ka), and was most likely facilitated by the introduction and utilization of cold-tolerant barley agriculture and sheep [8], which were first domesticated in west Asia around 10 ka [9,10]. However, it remains unknown who brought this exotic crop and livestock to the TP.

As revealed by archaeological evidence, modern humans settled extensively in the northeastern TP (below 2500 m above sea level (masl)) with millet cultivation around ∼5.2 ka, and further expanded to high-altitude plateau areas (above 2500 masl) ∼3.6 ka with barley and sheep from west Asia [8]. These observations have raised the question of whether barley agriculture was introduced to the TP by millet farmers from lower elevations or mediated by immigrations from the west. According to previous genetic studies, most Tibetan genetic components can be traced back to Neolithic immigrations from northern China [1,5,6], raising the possibility that it was millet farmers who brought barley agriculture to the TP around 3.6 ka. However, these studies, especially those on mitochondrial DNA (mtDNA), have also observed abundant lineages likely introduced to the Tibetans during the Holocene (which began ∼11.7 ka) [1,5,6,11]. Given the limited molecular resolution, and a lack of both genetic and archaeological evidence in previous studies, it remains unclear whether these lineages are associated with millet farmers or with the immigration of foragers during the early Holocene (∼11.7–8.3 ka) [12,13].

**Figure 1.**
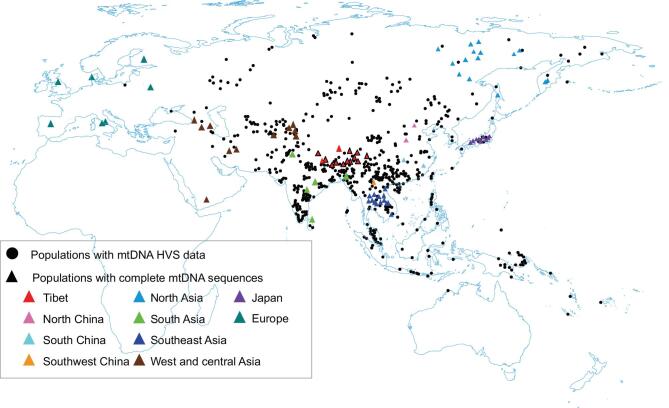
Locations of samples in the present study. Black dots: populations with hypervariable segments (HVS) sequence data. Triangles: populations with complete mtDNA sequence data, including 671 newly sequenced samples from 11 Tibetan populations in this study (black outline) and 9789 from 109 populations from previous literature. Locations are indicated by different colors.

In this study, by exploring mitogenomes in Tibetans and surrounding populations (especially those with suggested Neolithic millet farmer ancestry) (Fig. 1 and Supplementary Tables S1-S3), as well as radiocarbon dating of cereal remains from archaeological sites on the TP and northern China (Supplementary Tables S4-S6), we aimed to address whether millet farmers contributed to the Tibetan gene pool at the time of barley dispersal to the TP (∼3.6 ka), thus providing deeper insight into the origin of Tibetans.

## RESULTS

### Haplogroups M9a1a1c1b1a and A11a1a are genetic legacies of Neolithic millet farmers

Comparing mtDNA variations between Tibetans and populations from surrounding regions (Supplementary Tables S1-S3), we found that 70.5% (473/671) of lineages—e.g. M9a1a1c1b1a, A11a1a, G3a1a, M13a1b, M13a2, M62b1 and M62b2—displayed *de novo* differentiation in contemporary Tibetans (Supplementary Fig. S1), with the remaining (29.5%) possibly introduced via recent gene flow (Supplementary Information (SI)-1; Supplementary Fig. S1, Supplementary Table S7). Among the *de novo* differentiation lineages, most (78.9%; 373/473) were <6 ka in age (Supplementary Table S8) when the Neolithic period on the TP began [13], consistent with previous suggestions of immigrations to the TP during the Neolithic period [1,5,6].

**Figure 2.**
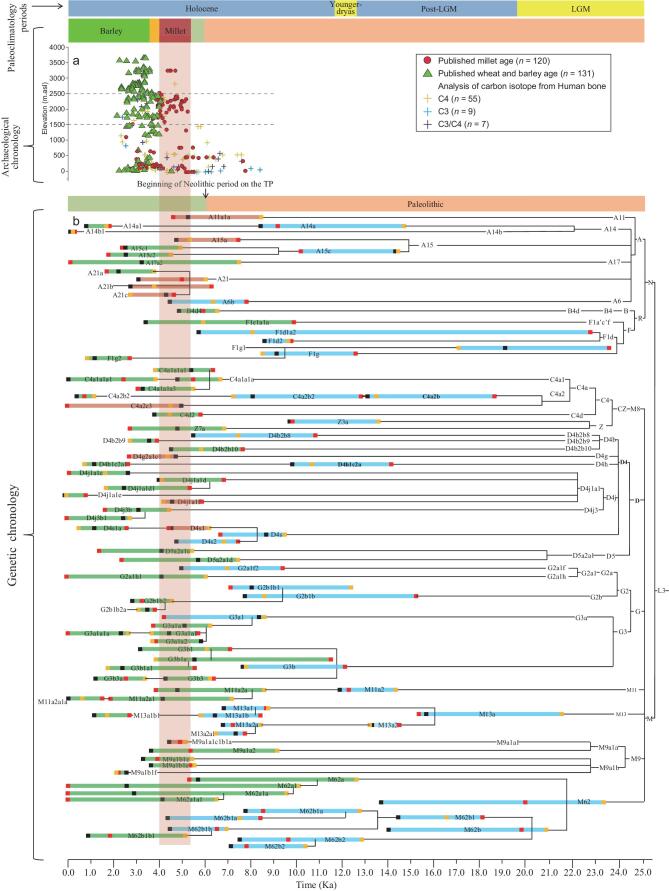
Paleoclimatological, archaeological and genetic chronologies on the Tibetan Plateau. (a) Radiocarbon dates of cereal remains (Supplementary Tables S4 and S5) and human bones with carbon isotope signals from different elevations in northern China (Supplementary Table S6). (b) Schematic tree of mtDNA lineages in Tibetans. Ages of independently differentiated nodes are shown. Coalescent ages estimated by coding regional variants, synonymous positions, and complete mitogenome variants are indicated by black, red and orange dots, respectively. Haplogroups within the 5.2–4.0 ka timeframe are in red. Paleolithic and other Neolithic haplogroups are in blue and green, respectively.

We analyzed the radiocarbon dates of cereal remains from different elevations along the TP and northern China, and found that millet was cultivated at Loess Plateau (<2500 masl and located to the northeast of TP) between 5.2 and 3.6 ka (Fig. 2a). Of note, barley and wheat remains began to appear in the Hexi Corridor, and the lower elevations of the northeastern TP, ∼4 ka [8,14], leading to a coexistence of indigenous millet and exotic barley–wheat cultivation in the area ∼4.0–3.6 ka (Fig. 2a). Thus, we shifted attention to haplogroups with ages within 5.2–4.0 ka, including A11a1a, A15a, A21, D4g2a1c1, D4j1a1f, D4s1, C4a1a1 and M9a1a1c1b1a (Fig. 2b, Supplementary Table S8).

**Figure 3.**
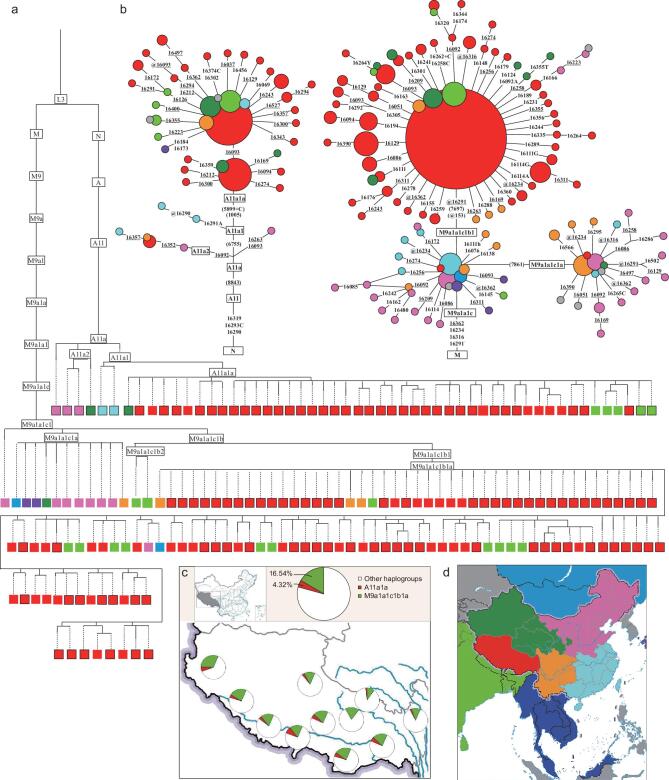
Phylogeographic analysis of haplogroups A11a and M9a1a1c1. (a) Phylogenetic tree of haplogroups A11a and M9a1a1c1 based on whole mtDNA sequences from this study (black outline) and previous literature (no outline). (b) Median-joining networks of A11a and M9a1a1c1 based on HVS data set (Supplementary Table S3). Mutations outside of HVS-I and HVS-II are in parentheses. Recurrent mutations are underlined. `@' denotes a reverse mutation. (c) Frequencies of haplogroups M9a1a1c1b1a (green) and A11a1a (red) in Tibetan populations. Locations are indicated by different colors (d).

Phylogeographic analyses, very useful in identifying the origins and migrations of haplogroups [15], were performed to determine whether these haplogroups have roots in northern China (SI-2), where millet agriculture originated [16,17]. As a result, we identified two haplogroups (A11a1a and M9a1a1c1b1a), both with ancestor nodes (A11a and M9a1a1c1, respectively) distributed mainly in northern China (Fig. 3, Supplementary Fig. S2 and S3) and thus most likely originating from northern China. Coalescent ages of the two ancestor nodes were estimated to be 12.7–11.7 ka (A11a) and 10.1–6.4 ka (M9a1a1c1) (Table 1). These results matched the origin and development of millet agriculture, which originated about 11–9.5 ka [16] and subsequently became dominant in northern China from 10–6 ka [18,19], suggesting a close relationship between these two haplogroups and millet farming. Further support came from the observation that A11a2 (a subhaplogroup of A11a) and M9a1a1c2 (a sister haplogroup of M9a1a1c1) were also distributed in northern China (Fig. 3 and Supplementary Fig. S2) with ages estimated to be 7.0–5.0 ka (Table 1), thus showing a strong association with the intensive expansion of millet farming in the Yangshao period (7.0–5.0 ka) [18,19]. These findings strongly link A11a1a and M9a1a1c1b1a with the origin, development and westward spread of millet agriculture, and thus suggest that both haplogroups most likely represent the genetic legacy of the Neolithic millet farmers.

**Table 1.** Age estimations of haplogroups M9a1a1c1b1a and A11a1a, as well as their ancestor and sister lineages.

		Coding region synonymous substitution^†^		Coding region (577–16 023)^†^		Complete mtDNA genome^†^	
Haplogroup	*N*	ρ ± σ	Age (ka)	ρ ± σ	Age (ka)	ρ ± σ	Age (ka)
A11a	53	1.51 ± 0.93	11.88 ± 7.36	3.30 ± 1.30	11.67 ± 4.58	4.92 ± 1.49	12.73 ± 3.86
A11a2 (Northern China)	3	1.00 ± 1.00	7.87 ± 7.87	1.67 ± 1.00	5.89 ± 3.53	2.67 ± 1.25	6.89 ± 3.22
A11a1a (Tibet)	47	0.55 ± 0.13	4.36 ± 1.06	1.45 ± 0.21	5.11 ± 0.74	3.10 ± 0.85	8.03 ± 2.21
M9a1a1c1	166	0.81 ± 0.13	6.40 ± 1.05	2.19 ± 0.95	7.73 ± 3.37	3.89 ± 1.34	10.06 ± 1.47
M9a1a1c1a (Northern China)	11	0.64 ± 0.24	5.01 ± 1.89	1.09 ± 0.30	3.85 ± 1.06	2.00 ± 0.43	5.17 ± 1.10
M9a1a1c1b1a (Tibet)	154	0.65 ± 0.09	5.11 ± 0.75	1.20 ± 0.15	4.24 ± 0.52	1.92 ± 0.19	4.97 ± 0.51

^†^Mutation rates from Soares *et al*. [36].

### M9a1a1c1b1a and A11a1a were more frequent in Tibetans ∼3.6 ka

**Figure 4.**
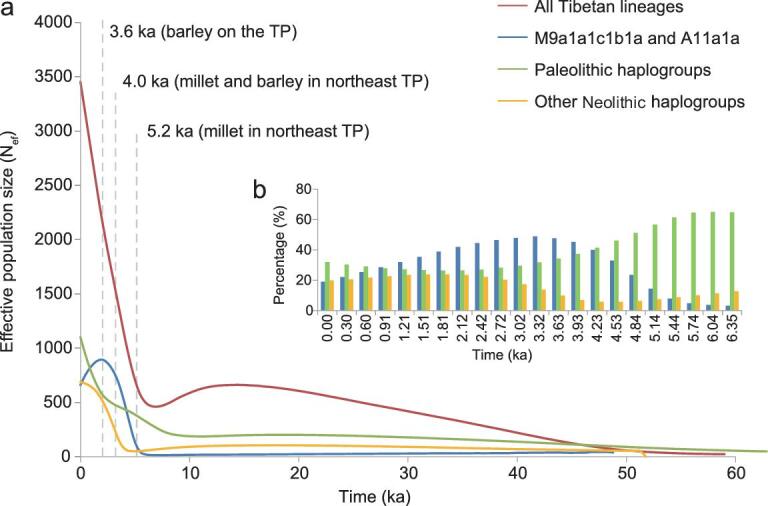
Proportional changes of two lineages of millet farmers (A11a1a and M9a1a1c1b1a) in Tibetans through time. (a) BSPs of all Tibetans (red), M9a1a1c1b1a and A11a1a (blue), Paleolithic lineages (green) and other Neolithic haplogroups (orange). (b) Proportions of M9a1a1c1b1a and A11a1a (blue), Paleolithic lineages (green) and other Neolithic haplogroups (orange) in Tibetans through time since the beginning of the Neolithic period.

Of note, both haplogroups showed the highest frequencies (M9a1a1c1b1a: 16.5% and A11a1a: 4.3%) in Tibetans, accounting for 20.9% of the Tibetan gene pool (Supplementary Fig. S4). As this result does not necessarily represent their past distribution frequencies, we estimated their proportions through time and found that their percentages continued to increase since 5.14 ka, reaching 48.8% at 3.3 ka (Fig. 4 and Supplementary Table S9). After this, the proportion began to decrease and reached 19.1% by the present day (a result very close to the observed value of 20.9% in our Tibetan samples; Supplementary Table S9), which is probably due to immigrations to the TP during the historical period [20]. Thus, these results suggest a substantial genetic contribution of the Neolithic millet farmers from the Loess Plateau of northern China to the Tibetans ∼3.3 ka (Fig. 4; Supplementary Table S9), a time frame very close to that (3.6 ka) revealed by archaeological evidence when higher altitudes began to be permanently populated by groups who engaged in barley cultivation and sheep herding [8].

### M9a1a1c1b1a and A11a1a played important roles in shaping the genetic landscape of current Tibetans

**Figure 5.**
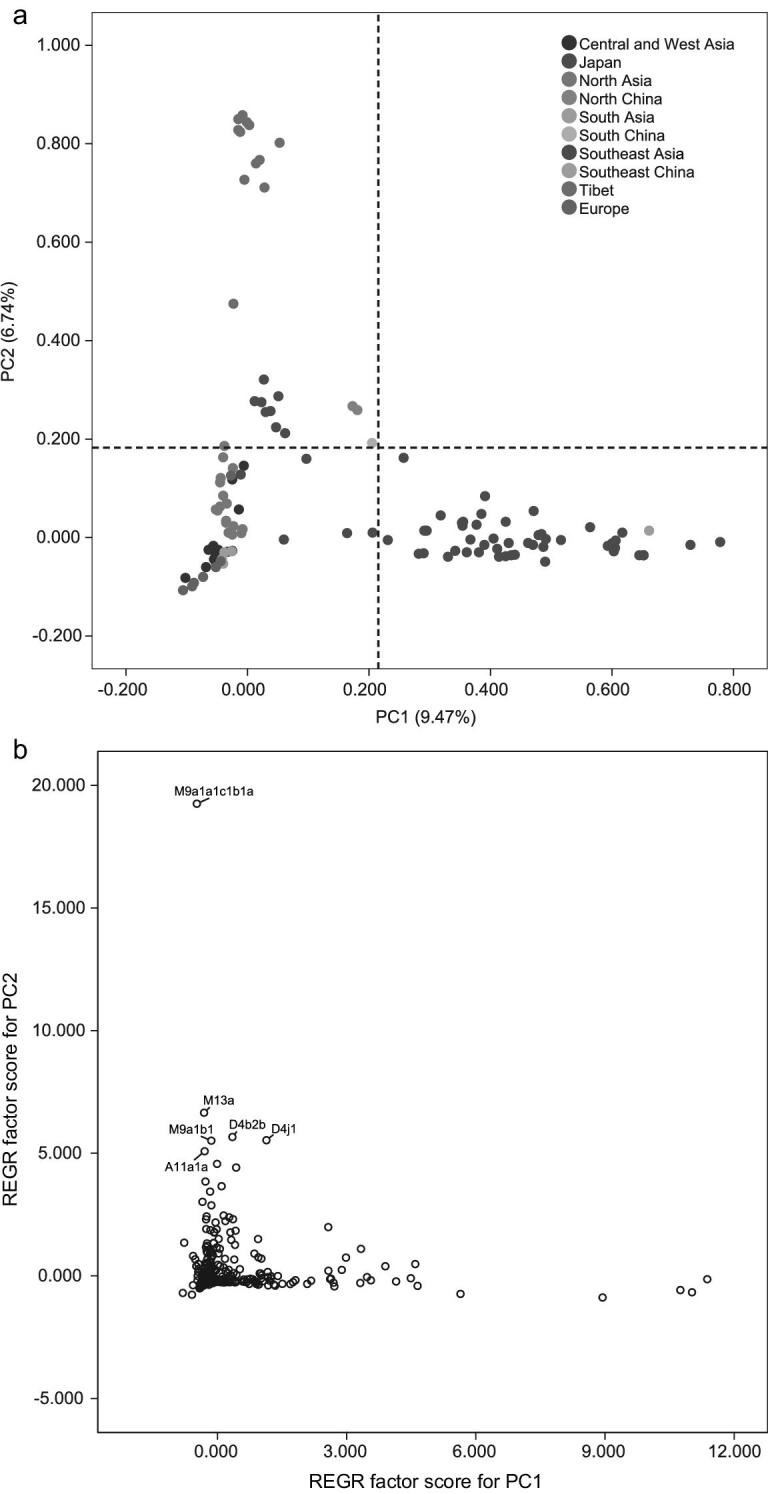
PCA of Tibetans and other Eurasian populations based on whole mitochondrial genome data. (a) PC map of 120 populations based on haplogroup frequencies. Populations from different regions are indicated by different colors. (b) Plot of haplogroup contribution of first and second PCs. Contribution of each haplogroup was calculated as the factor scores for PC1 and PC2 with REGR in SPSS.

Both M9a1a1c1b1a and A11a1a are distributed ubiquitously in contemporary Tibetans, with total frequencies ranging from 12.0% (Baqing County, Naqu) to 37.5% (Ngari) across all regional Tibetan populations studied (Fig. 3c). This suggests that the millet farmer lineages contributed to and existed in the proto-gene pool of Tibetans before their expansion to different regions on the TP. Indeed, much closer genetic affinity was observed among all regional Tibetan populations (Fig. 5a), with haplogroup M9a1a1c1b1a contributing most to this clustering pattern (Fig. 5b), implying that millet farmer components played an important role in shaping the genetic landscape of Tibetans.

## DISCUSSION

In this study, we combined archaeological and genetic data to investigate the mode of spread of barley agriculture to the TP. Specifically, we identified that haplogroups A11a1a and M9a1a1c1b1a, which can trace their ancestry back to northern China ∼10 ka and underwent *in situ* differentiation in Tibetans 5.2–4.0 ka, likely represent the genetic legacy of Neolithic millet farmers in contemporary Tibetans. Interestingly, ancient samples excavated from different Neolithic sites in northern China, in which millet was the most important crop and thus most likely represent the remains of the Neolithic millet farmers, show close genetic affinity to our identified genetic legacy of millet farmers in the Tibetans. For example, haplogroup M9a1a1c (defined by HVS-I variants 16223–16291-16234–16316-16362), an ancestor type to M9a1a1c1b1a, has been observed at the Miaozigou (5.5–5.0 ka) [21,22] and Zhukaigou sites (4.2–3.5 ka) [23,24]. The above evidence argues for the existence of an ancestor lineage of haplogroup M9a1a1c1b1a in northern and northwestern China during the Neolithic period, thus strongly supporting their close relationship with Neolithic millet farmers.

The maternal genetic components of millet farmers, which accounted for 20.9% of current Tibetans and may have been more frequent prior to historical immigrations (40–50%), demonstrated substantial genetic contributions of Neolithic millet farmers from northern China into Tibetans. Moreover, this contribution, represented at least by the two haplogroups (A11a1a and M9a1a1c1b1a) identified in the present study, existed in Tibetans at relatively high frequencies for >3 ka, and even contributed to the genetic differentiation observed between the Tibetans and other ethnic groups. Therefore, by showing that the ancestry of the Tibetans on the TP can largely be traced back to millet farmers from northern China, our study indicates substantial migration of millet farmers onto the TP, which probably occurred during the Neolithic period. Although it was barley agriculture that finally promoted the permanent settlement of humans at high altitudes after 3.6 ka [8], the genetic origins of these settlers were primarily from Neolithic millet farmers in the Yellow River Basin, rather than immigrants from the west. Given that barley joined or displaced millet at some archaeological sites on the northeast TP during 4.0–3.6 ka [8], the most reasonable explanation for our observation is that millet farmers adopted barley agriculture after their arrival to the northeast TP, and further migrated to high altitudes after 3.6 ka with barley cultivation technology.

Of note, our observation based on mtDNA is somewhat similar to the Neolithic migration of males from northern China, in which a westward expansion of males initiated in the middle Yellow River Basin during 10.0–5.0 ka [4,25]. However, controversy still exists regarding whether these male lineages from northern China dispersed onto the TP [4], or just moved to the upper basin of the Yellow River and mixed with local people therein, who further occupied high altitudes after 3.6 ka [25]. To better understand the migration history of the Tibetans, investigations based on large-scale and high-resolution Y chromosome data are needed to explore whether the migration of millet farmers onto the TP also involved the expansion of males.

## CONCLUSIONS

In summary, by combining archaeological and high-resolution genetic data, we have identified a substantial genetic contribution of Neolithic millet farmers, represented by haplogroups A11a1a and M9a1a1c1b1a, to contemporary Tibetans, which has even played an important role in shaping the Tibetan matrilineal landscape. Therefore, our study provides the first piece of evidence demonstrating that it was Neolithic millet farmers originating from northern China, rather than immigrants from the west, who brought barley agriculture to the TP ∼3.6 ka and contributed to the permanent human occupation of the TP.

## METHODS

### mtDNA sequencing

Blood samples from 671 Tibetans from 11 Tibetan populations were collected, covering all seven districts of Tibet (Fig. 1, Supplementary Table S1). Blood samples from an additional 11 Chinese individuals belonging to haplogroups of interest were also collected for mitogenome sequencing. The experimental protocol was approved by the Ethics Committee at the Kunming Institute of Zoology, Chinese Academy of Sciences. Informed consent was obtained from each individual before the study. Complete mtDNA genomes of the samples were enriched by capture-based strategies using a MyGenostics Human Mitochondria Capture Kit (MyGenostics Inc., Beijing, China). Sequencing was carried out using an Illumina HiSeq X Ten platform at MyGenostics. The average depth of sequencing was 4212×, ranging from 1003× to 40 278×.

### mtDNA data collection

Additionally, 201 whole mitogenomes (Supplementary Table S2) and 7405 mtDNA hypervariable segments (HVS) sequences (Supplementary Table S3) from Tibetans were collected from previous literature and analyzed. For comparison, mtDNA data from surrounding areas, including 9588 mitogenomes (Supplementary Table S2) and 41 320 HVS data (Supplementary Table S3), were also included.

### Archaeological data

Published radiocarbon dates of millet (*n* = 120, Supplementary Table S4), wheat and barley remains (*n* = 131, Supplementary Table S5) from archaeological sites at different elevations in northern China were collected. In addition, analysis of human bones with carbon isotope (δ^13^C) values (*n* = 81, Supplementary Table S6), which reflect the dietary consumption of C4 (millets) and C3 plants (rice, barley, wheat, and other natural vegetation) [26], were also collected from the literature.

### Quality control and haplogroup allocations

Quality control was performed according to previous research [27]. Potential phantom mutations were checked and corrected. The sequencing outputs were edited and aligned by Lasergene (DNAStar Inc., Madison, Wisconsin, USA) and compared with the revised Cambridge Reference Sequence [28]. Haplogroup allocations of all mtDNAs (including fresh mitogenomes; Supplementary Table S10) were determined according to our previous work [29–31] and mtDNA tree Build 17 (http://phylotree.org/) [32].

### Phylogeographic analysis

Phylogenetic trees of lineages of interest were reconstructed manually based on the complete sequences and confirmed by mtphyl software [33]. Rho (ρ) and standard errors (σ) were used to evaluate the coalescent ages of haplogroups [34,35]. The adopted mutation rates were obtained from previous research [36]. Contour maps of haplogroup spatial frequencies were constructed using the Kriging algorithm in Surfer 8.0 (Golden Software Inc. Golden, Colorado, USA).

### Bayesian skyline plot analysis

Bayesian skyline plots (BSP) for effective population size (*N*_ef_) through time were reconstructed based on 13 protein coding regions using BEAST v1.7.5. [37], as described elsewhere [38,39]. In the BSP analysis, a general time-reversible substitution model with site-specific rates for the first, second and third codons was adopted to infer the ancestral gene trees. To estimate the timescale to the *N*_ef_ change, a strict molecular clock with a fixed rate of 1.691e^−8^ substitutions per site per year [40] was chosen. Each Markov chain Monte Carlo simulation was run for 40 000 000 generations and sampled every 4000 generations, with the first 40 000 generations discarded as burn-in. The results were visualized with Tracer v1.5 (http://tree.bio.ed.ac.uk/software/tracer/). Population growth rate and time of population growth were calculated based on skyline plots, as per our previous study [41]. To avoid potential biases caused by sampling, we applied the pooled sampling strategy, which provides the best scheme for inferring demographic history [42], for BSP analysis. In detail, 20 samples were picked randomly from each of the 11 Tibetan populations and pooled as one data set to perform BSP analysis. Proportional changes of certain haplogroups in Tibetans through time were calculated based on *N*_ef_ values of the haplogroups and all Tibetans in the BSP analyses.

## Supplementary Material

nwz080_Supplemental_File
