# Supplementary material for: Neolithic millet farmers contributed to the permanent settlement of the Tibetan Plateau by adopting barley agriculture
Source: Natl Sci Rev. 2019 Jun 21;6(5):1005–13. doi: 10.1093/nsr/nwz080 (PMC8291429; doi:10.1093/nsr/nwz080)
Supplement: nwz080_Supplemental_File [file nwz080_supplemental_file.pdf]

Supplementary Information (SI)

SI text

1. Identification of independently differentiated haplogroups in Tibetans.

We first identified haplogroups that differentiated independently in Tibetans based on the following. (1) If millet farmers moved onto the plateau 3.6 thousand years ago (ka), genetic relics should exist and display *de novo* differentiation in contemporary Tibetans. (2) Age estimations are essential in the identification of genetic relics of Neolithic millet farmers. However, biases will be introduced if one roughly estimates the age of a haplogroup based only on the Tibetan sequences belonging to this haplogroup, because even within one haplogroup Tibetan individuals would distribute sporadically on different branches. Therefore, only ages of independently differentiated haplogroups in Tibetans were precisely estimated.

By combining mitochondrial genome data from previous literature and the present study (Table S2), we identified independently differentiated haplogroups in Tibetans by employing phylogeographic analysis adopted in earlier research [1] according to the following criteria: (1) lineages/branches should be occupied mainly by Tibetan samples; (2) samples from other regions should have sporadic distribution in these lineages/branches based on from recent gene flow from the Tibetan Plateau into these areas. Following these criteria, we identified 140 lineages specific in Tibetans (e.g., M9a1a1c1b1a, A11a1a, G3a1a, M13a1b, M13a2, M62b1, M62b2) with *de novo* differentiations (Figure S3).

2. Identification of millet farmer genetic relics among Neolithic haplogroups

We found haplogroups A11a1a, A15a, A21, C4a2c3, D4g2a1c1, D4j1a1f, D4s1, C4a1a1, and

M9a1a1c1b1a with ages between 5.2–4.0 ka. Further phylogeographic analyses indicated that haplogroup D4s1, with ancestor node (D4s, which was also specific in Tibetans) had an age during the Paleolithic period (Figure S3; Table S7) and likely descended from the Paleolithic hunter-gatherers on the plateau [2] and further differentiated during the Neolithic period. It is therefore probable that some Paleolithic haplogroups also experienced population expansions during the Neolithic period, especially during 5.2–4.0 ka, which was likely facilitated by the introduction of millet agriculture. This finds support from the rapid population growth of Paleolithic haplogroups during the Neolithic era (main text, Fig. 4). Haplogroup A21, which originated *in situ* in Tibetans during the Neolithic period, would also have been affected by the arrival of millet agriculture. However, whether it originated from northern China during the early Holocene remains unknown according to the currently available mitogenome datasets. Haplogroups D4j1a1f, D4g2a1c1 A15a, and C4a1a1, which show close genetic relationships between Tibetans and populations from southeast and South Asia (D4j1a1f and D4g2a1c1), northern China and Japan (A15a), and central and northern Asia (C4a1a1) (Figure S3), reflect Neolithic connections between Tibetans and populations from other regions. Specifically, we found that haplogroups A11a1a and M9a1a1c1b1a can be traced to northern China, with an origin time similar to the origin and development of millet agriculture, and thus could be considered as genetic relics of millet farmers that are still retained in contemporary Tibetans (main text).

References

1. Olivieri A, *et al.* Mitogenome Diversity in Sardinians: A Genetic Window onto an Island's Past. *Mol Biol Evol* **34**:1230-1239 (2017).

2. Zhao M, *et al.* Mitochondrial genome evidence reveals successful Late Paleolithic settlement on the Tibetan Plateau. *Proc Natl Acad Sci USA* **106**:21230-21235 (2009).

Supplementary files:

Table S1. Information on 11 Tibetan populations newly sequenced in this study.

Table S2. Whole mitogenome dataset from previous literature.

Table S3. HVS dataset from previous literature.

Table S4. ^{14}C dates of archaeological sites with millet remains (n = 120).

Table S5. ^{14}C dates of archaeological sites with wheat and barley remains (n = 131).

Table S6. Radiocarbon dates of sites with human bones and stable isotope signals (n = 81).

Table S7. Distribution of haplogroups in different geographic regions based on HVS and whole mitogenome datasets.

Table S8. Coalescent ages of haplogroups with *de novo* differentiations in Tibetans.

Table S9. Estimated proportions of different components in Tibetans since the Neolithic period.

Table S10. Complete mtDNA variants and haplogroup allocations of the 682 samples in this study.

Figure S1. Schematic phylogenetic trees of Tibetan haplogroups based on whole mtDNA sequences from this study (black outline) and previous literature (no outline). Only haplogroups in more than two Tibetan samples were included in the tree. Lengths of each branch are in proportion to variants. Locations are indicated by different colors. Haplogroups with *de novo* differentiation in Tibetans are indicated in orange.

Figure S2. Contour maps of A11a and M9a1a1c1 and their sub-lineages (A11a2, A11a1a, M9a1a1c1a, and M9a1a1c1b1a) based on the HVS dataset (Table S3).

Figure S3. Phylogenetic trees of A11a1a and M9a1a1c1b1a. Nucleotide position numbers are consistent with the revised Cambridge reference sequence (rCRS). Suffixes C, G, and T refer to transversions; ‘d’, deletion; ‘s’, synonymous mutation; ‘ns’, non-synonymous mutation; ‘+’, insertion; recurrent mutations are underlined; ‘@’, reverse mutation; ‘h’, heterogeneity; ‘t’, change in transfer RNA; ‘r’, change in ribosomal RNA gene; nc, mutations at intergenic noncoding regions in segments 577–16023. Com, syn, and cod: coalescent age calculated based on complete genome substitutions, coding region synonymous substitutions, and coding region variants, respectively.

Figure S4. Frequencies of haplogroups in Tibetans. Haplogroups M9a1a1c1b1a and A11a1a are indicated in red, with remaining haplogroups indicated in blue.

Table S1. Information on 11 Tibetan populations newly sequenced in this study.

Population	Location	Ethnic group	Latitude	Longitude	N	Altitude (masl)
Tib_1	Ngari	Tibetan	32.50	80.11	16	4280
Tib_2	Chamdo	Tibetan	31.14	97.17	85	3230
Tib_3	Lhasa	Tibetan	29.64	91.11	68	3660
Tib_4	Nyingchi	Tibetan	29.65	94.36	71	2994
Tib_5	Anduo, Naqu	Tibetan	32.26	91.68	63	4679
Tib_6	Baqing, Naqu	Tibetan	31.92	94.05	50	4148
Tib_7	Jiali, Naqu	Tibetan	30.64	93.23	44	4501
Tib_8	Kangma, Shigatse	Tibetan	28.56	89.68	77	4360
Tib_9	Lazi, Shigatse	Tibetan	29.08	87.64	75	4026
Tib_10	Zhongba, Shigatse	Tibetan	29.77	84.03	48	4587
Tib_11	Shannan	Tibetan	28.35	92.21	74	3572
**Total**					**671**	

Table S2. Whole mitogenome dataset from previous literature.

Population code or accession number	Information	Size	Reference	Log	Lat
**Tibet**		**201**			
HM346916, HM346928	Tibet, China	13	(1)	NA	NA
FJ544230-FJ544243, FJ968772-FJ968775, GU014563-GU014569	Tibet, China	25	(2)	NA	NA
GQ895140-GQ895170	Tibet, China	31	(3)	NA	NA
FJ748704-FJ748759	Tibet, China	56	(4)	NA	NA
KF056243-KF056318	Tibet, China	76	(5)	85.98	28.15
**Northwest China**		**53**			
HGDP00945-HGDP01251	Northwest China	53	HGDP	NA	NA
**North China**		**232**			
CHD	Beijing, China	139	1000 genomes	116.41	39.90
JF824905-JF824997	Henan, China	93	(6)	113.75	34.77
**South China**		**97**			
CHS	Hunan and Fujian, China	97	1000 genomes	119.30	26.10
**Southwest China**		**98**			
CDX	Yunnan, China	98	1000 genomes	100.80	22.01
**North Asia**		**832**		135.10	48.52
FJ951438-FJ951618	Complete sequences of C and D	181	(7)	142.95	50.69
JN857009–JN857063	Complete sequences of North Asian rare haplogroups	55	(8)	124.12	66.76
EF153771–EF153833, EF397558–EF397562, EF486517–EF486519	71 complete mtDNA from Siberia	71	(9)	158.71	55.92
KF148067-KF148359, KF148361-KF148592	Evenks and Evens, Udegey from Amur-Ussuri	525	(10)		

	Taimyr	24		87.11	66.31
	Stony Tunguska	39		94.75	63.17
	Nyukzha	46		98.97	59.81
	Iengra	21		141.86	60.94
	Sakkyryyr	23		125.86	69.11
	Sebjan	18		127.97	66.80
	Tompo	27		134.57	66.17
	Berezovka	15		150.56	67.42
	Kamchatka	39		158.21	54.59
	Udegey	31		133.76	43.61
	Vilyuy	49		105.12	63.17
	Central	88		129.64	61.37
	Northeast	32		135.18	69.48
	Yukaghir	20		147.31	65.01
	Koryak	15		159.53	56.13
	Nivkh	38		134.10	46.38
**Southeast Asia**		**1266**			
KHV	Kinh in Ho Chi Minh City, Vietnam	32	1000 Genomes	106.63	10.82
KX456435–KX457668	Thailand and Laos	1234	(11)		
	Khon Mueang KM1, Thailand	25		99.45	19.53
	Khon Mueang KM2, Thailand	25		98.51	18.84
	Khon Mueang KM3, Thailand	24		98.49	19.03
	Khon Mueang KM4, Thailand	25		98.68	18.84
	Khon Mueang KM5, Thailand	23		99.08	18.36
	Khon Mueang KM6, Thailand	25		98.53	18.36
	Khon Mueang KM7, Thailand	25		98.31	18.09

Khon Mueang KM8, Thailand	25	98.95	18.07
Khon Mueang KM9, Thailand	24	99.17	18.05
Khon Mueang KM10, Thailand	25	99.21	17.88
Yuan YU1, Thailand	17	100.22	17.38
Yuan YU2, Thailand	25	99.77	13.36
Shan SH, Thailand	25	98.18	19.45
Lao Isan IS1, Thailand	25	103.44	15.53
Lao Isan IS2, Thailand	25	102.04	15.68
Lao Isan IS3, Thailand	25	101.77	17.21
Lao Isan IS4, Thailand	25	105.40	14.70
Lao LA1, Laos	25	102.47	19.49
Lao LA2, Laos	24	102.63	17.98
Phutai PT, Thailand	25	103.66	17.19
Kalueng KL, Thailand	25	104.50	17.15
Seak SK, Thailand	26	104.56	17.36
Nyaw NY, Thailand	25	104.17	17.49
Black Tai BT1, Thailand	25	101.75	17.70
Black Tai BT2, Thailand	25	99.73	13.83
Phuan PU1, Thailand	25	99.76	17.44
Phuan PU2, Thailand	25	100.52	16.14
Phuan PU3, Thailand	25	99.89	17.84
Phuan PU4, Thailand	25	100.59	14.91
Phuan PU5, Thailand	25	99.95	14.17
Mon MO1, Thailand	25	102.21	14.85
Mon MO2, Thailand Myanmar border	23	98.50	14.98
Mon MO3, Thailand Myanmar border	15	98.34	15.06

	Mon MO4, Thailand	25		100.54	14.70
	Mon MO5, Thailand	22		99.88	13.64
	Khmer KH1, Thailand	19		103.13	14.66
	Khmer KH2, Thailand	25		103.07	15.23
	Nyahkur BO, Thailand	23		101.46	15.51
	Suay SU, Thailand	25		103.13	14.81
	Soa SO, Thailand	25		104.25	17.15
	Bru BU, Thailand	24		103.68	17.36
	H’tin TN1, Thailand	25		100.38	18.93
	H’tin TN2, Thailand	25		100.31	19.30
	H’tin TN3, Thailand	25		100.48	19.20
	Khamu KA, Thailand	25		100.88	19.36
	Blang BL1, Thailand	25		99.60	20.07
	Blang BL2, Thailand	25		99.51	19.92
	Paluang PL, Thailand	25		99.10	19.55
	Lawa LW1, Thailand	22		97.78	18.20
	Lawa LW2, Thailand	24		98.11	17.95
	Lawa LW3, Thailand	24		98.62	18.16
**South Asia**		**688**			
BEB	Bengali in Bangladesh	86	1000 genomes	90.91	23.39
GIH	Gujarati Indians in Houston, Texas, USA	106	1000 genomes	78.96	20.59
ITU	Indian Telugu in the UK	103	1000 genomes	82.22	22.43
PJL	Punjabi in Lahore, Pakistan	290	1000 genomes	74.36	31.52
STU	Sri Lankan Tamil in the UK	103	1000 genomes	80.77	7.87
**Central and West Asia**		**997**			
KM986560-KM986627	Yemen	113	(12)	48.52	15.55

HM852756-HM852785	Armenia	30	(13)	45.04	40.07
HM852786-HM852815	Azerbaijan	30	(13)	47.58	40.14
HM852875-HM852902	Georgia	28	(13)	43.36	42.32
HM852846-HM852874	Turkey	29	(13)	35.24	38.96
HM852816-HM852845	Iran	30	(13)	53.69	32.43
KC911275-KC911629	Iran	355	(14)	55.81	32.77
MF522841-MF523222	Pamir	382	(15)		
	Sarikoli Tajik, Taxkorgan, Xinjiang, China	86		75.23	37.77
	Wakhi Tajik, Taxkorgan, Xinjiang, China	66		75.44	37.64
	Pamiri Tajik, Gorno-Badakhshan, Tajikistan	50		73.09	38.41
	Lowland Tajik, Dushanbe, Tajikistan	28		68.79	38.56
	East Pamir Kyrgyz, Taxkorgan, Xinjiang, China	68		75.30	37.49
	West Pamir Kyrgyz, Gorno-Badakhshan, Tajikistan	3		73.17	38.39
	Lowland Kyrgyz, Artux, Xinjiang, China	54		76.17	39.72
	Lowland Uygur, Artux, Xinjiang, China	27		75.72	39.67
**Western Eurasia**		**4535**			
GBR	British from England and Scotland	92	1000 genomes	-2.07	53.72
FIN	Finnish in Finland	99	1000 genomes		
IBS	Iberian populations in Spain	107	1000 genomes		
TSI	Toscani in Italia	107	1000 genomes		
KX358471-KX358508	Belarus	38	(16)	25.75	61.92
KY408145-KY410236	Italy	2092	(17)	-3.75	40.46
KF163059-KF162886	Danish	2000	Li et al. submitted to NCBI	12.57	41.87
**Japan**		**790**			
JPT	Japan	118	1000 genomes	139.79	35.75

AP008249-AP008920	Complete sequences of Japanese	672	(18)		
	Japanese centenarian from Gifu	11		136.76	35.42
	Japanese centenarian from Tokyo	85		139.69	35.69
	Japanese patient with Parkinson's Disease from Tokyo	96		139.55	35.67
	Japanese patient with Alzheimer's Disease from Chiba	96		140.11	35.61
	Obese young Japanese male from Aichi	96		136.91	35.18
	Non-obese young Japanese male from Aichi	96		137.05	35.10
	Japanese diabetic patient from Aichi	96		137.24	35.03
	Japanese diabetic patient with angiopathy from Tokyo	96		139.85	35.71
Total		**9789**			

References

1 Peng MS, et al. (2011) Inland post-glacial dispersal in East Asia revealed by mitochondrial haplogroup M9a'b. BMC Biology 9.

2 Zhao M, et al. (2009) Mitochondrial genome evidence reveals successful Late Paleolithic settlement on the Tibetan Plateau. Proceedings of the National Academy of Sciences of the United States of America 106(50):21230-21235.

3 Qin ZD, et al. (2010) A Mitochondrial Revelation of Early Human Migrations to the Tibetan Plateau Before and After the Last Glacial Maximum. (Translated from English) American Journal of Physical Anthropology 143(4):555-569 (in English).

4 Ji F, et al. (2012) Mitochondrial DNA variant associated with Leber hereditary optic neuropathy and high-altitude Tibetans. Proceedings of the National Academy of Sciences.

5 Kang L, et al. (2013) MtDNA lineage expansions in Sherpa population suggest adaptive evolution in Tibetan highlands. Mol. Biol. Evol.

6 Liu J, et al. (2011) Deciphering the signature of selective constraints on cancerous mitochondrial genome. Mol. Biol. Evol.

7 Derenko M, et al. (2010) Origin and post-glacial dispersal of mitochondrial DNA haplogroups C and D in northern Asia. PLoS One 5:e15214.

8 Derenko M, et al. (2012) Complete Mitochondrial DNA Analysis of Eastern Eurasian Haplogroups Rarely Found in Populations of Northern Asia and Eastern Europe. PLoS One 7(2):e32179.

9 Derenko M, et al. (2007) Phylogeographic analysis of mitochondrial DNA in northern Asian Populations. American Journal of Human Genetics 81(5):1025-1041.

10 Duggan AT, et al. (2013) Investigating the Prehistory of Tungusic Peoples of Siberia and the Amur-Ussuri Region with Complete mtDNA Genome Sequences and

Y-chromosomal Markers. PLoS One 8(12).
11 Kutanan W, et al. (2016) Complete mitochondrial genomes of Thai and Lao populations indicate an ancient origin of Austroasiatic groups and demic diffusion in the spread of Tai–Kadai languages. Human genetics:1-14.
12 Vyas DN, et al. (2016) Bayesian analyses of Yemeni mitochondrial genomes suggest multiple migration events with Africa and Western Eurasia. (Translated from eng) American Journal of Physical Anthropology 159(3):382-393 (in eng).
13 Schonberg A, Theunert C, Li M, Stoneking M, & Nasidze I (2011) High-throughput sequencing of complete human mtDNA genomes from the Caucasus and West Asia: high diversity and demographic inferences. Eur J Hum Genet 19(9):988-994.
14 Derenko M, et al. (2013) Complete Mitochondrial DNA Diversity in Iranians. PLoS One 8(11):e80673.
15 Peng M-S, et al. (2017) Mitochondrial genomes uncover the maternal history of the Pamir populations. Eur. J. Hum. Genet.
16 Pankratov V, et al. (2016) East Eurasian ancestry in the middle of Europe: genetic footprints of Steppe nomads in the genomes of Belarusian Lipka Tatars. Sci Rep 6:30197.
17 Olivieri A, et al. (2017) Mitogenome Diversity in Sardinians: A Genetic Window onto an Island's Past. Mol. Biol. Evol. 34(5):1230-1239.
18 Tanaka M, et al. (2004) Mitochondrial Genome Variation in Eastern Asia and the Peopling of Japan. Genome Research 14(10a):1832-1850.

Table S3. HVS dataset from previous literature.

	Ethnic group/Population	Location	Size	Log.	Lat.	Reference
**Tibet**			**7405**			
	Tibetan	Tibet, China	156	88.90	29.46	(1)
	Tibetan	Chamdo, China	29	97.17	31.27	(2)
	Tibetan	Lhasa, China	44	91.14	29.65	(2)
	Tibetan	Nyingchi, China	54	93.55	27.63	(2)
	Tibetan	Shigatse, China	29	88.90	29.40	(2)
	Tibetan	Shannan, China	55	95.21	28.94	(2)
	Tibetan	Nakchu, China	5	92.04	31.64	(2)
	Tibetan	Tibet, China	73	90.07	30.13	(2)
	Tibetan	Tibet, China	6109	88.79	30.15	(3)
	Tibetan	Chamdo, Tibet, China	61	97.17	31.27	(4)
	Tibetan	Lhasa, Tibet, China	59	91.14	29.65	(4)
	Tibetan	Nagqu, Tibet, China	58	92.04	31.75	(4)
	Tibetan	Ngari, Tibet, China	46	80.10	32.68	(4)
	Monba	Nyingchi, Tibet, China	51	93.55	27.63	(4)
	Tibetan	Nyingchi, Tibet, China	53	94.54	30.57	(4)
	Lhoba	Shannan, Tibet, China	20	95.21	28.94	(4)
	Tibetan	Shannan, Tibet, China	56	92.43	27.78	(4)
	Tibetan	Shigatse, Tibet, China	59	88.62	28.87	(4)
	Tibetan	Nakchu, China	168	92.04	31.64	(5)
	Tibetan	Rikaze, China	220	88.90	29.40	(5)
**Northwestern China**			**1195**			
	Bonan	Gansu, China	95	103.49	37.25	(6)

	Yugur	Gansu, China	100	103.83	36.21	(7)
	Dongxiang	Gansu, China	96	103.39	35.71	(8)
	Salar	Qinghai, China	99	101.01	36.24	(9)
	Han	Xi'an, China	85	108.94	34.29	(10)
	Tibetan	Yushu, Qinghai, China	44	97.01	33.01	(4)
	Salar	Jishishan, Linxia, Gansu, China	10	103.21	35.63	(11)
	Dongxiang	Jishishan, Linxia, Gansu, China	10	103.21	35.63	(11)
	Tibetan	Qinghai, China	56	101.14	36.35	(12)
	Han	Xining, Qinghai (NH), China	44	101.78	36.63	(13)
	Han	Gansu (NH), China	45	103.86	36.51	(13)
	Han	Xi'an, Shaanxi (NH), China	53	108.94	34.26	(13)
	Hui	Yili, Xinjiang, China	45	81.32	43.92	(14)
	Kazak	Kashen, Xinjiang, China	53	75.99	39.49	(14)
	Mongoloid	Yili, Xinjiang, China	49	81.29	43.99	(14)
	Uygur	Yili/Kashen, Xinjiang, China	47	75.95	39.47	(14)
	Uzbek	Yili, Xinjiang, China	58	81.33	43.91	(14)
	Han	Yili, Xinjiang, China	47	81.32	43.92	(15)
	Tibetan	Qinghai, China	76	101.01	37.11	(5)
	Tibetan	Gannan, Gansu, China	83	102.91	34.99	(5)
**North China**			**975**			
	Mongolian	Inner Mongolia, China	107	111.75	40.85	(16)
	Mongolian	Inner Mongolia, China	48	116.01	42.31	(16)
	Barghuts	Hulun Buir Aimak, Inner Mongolia, China, China	149	119.73	49.23	(17)
	Daur	Inner Mongolia, China	45	119.03	46.18	(18)

Ewenki	Inner Mongolia, China	47	120.70	48.22	(18)
Korean	Inner Mongolia, China	48	120.83	47.47	(18)
Mongolian	Inner Mongolia, China	48	122.10	49.43	(18)
Oroqen	Inner Mongolia, China	44	121.93	50.43	(18)
Han	Han, Northern China, China	60	125.50	47.13	(19)
Han	Dalian, Liaoning, China	51	121.62	38.93	(13)
Han	Chifeng, Neimeng, China	45	118.89	42.29	(13)
Han	Fengcheng, Liaoning, China	51	124.07	40.50	(15)
Han	Qingdao, Shandong, China	50	120.39	36.10	(15)
Han	Tai'an Shandong, China	76	117.08	36.23	(20)
Han	Yan Bian, Jilin, China	51	129.51	42.91	(21)
Korean	Yan Bian, Jilin, China	55	129.51	42.90	(21)
**South China**		**3975**			
Han	Dongguan, Guangdong, China	105	113.76	23.05	(22)
Zhuang	Fangchenggang, Guangxi, China	35	108.35	21.69	(23)
Zhuang	Liuzhou, Guangxi, China	70	109.42	24.34	(23)
Zhuang	Baise, Guangxi, China	107	106.62	23.91	(23)
Zhuang	Nanning, Guangxi, China	65	108.37	22.82	(23)
Li	Hainan, China	198	110.35	20.03	(23)
Han	Baise, Guangxi, China	62	106.62	23.91	(23)
Han	Fangchenggang, Guangxi, China	49	101.96	30.05	(23)
Han	Liuzhou, Guangxi, China	64	109.42	24.34	(23)
Han	Nanning, Guangxi, China	47	108.37	22.82	(23)
Han	Hainan, China	191	110.35	20.03	(23)
Bunun	Taiwan, China	18	120.89	23.68	(24)

Atayal	Taiwan, China	18	120.96	23.45	(24)
Amis	Taiwan, China	21	121.34	24.59	(24)
Paiwan	Taiwan, China	21	120.95	24.04	(24)
Han	Guangdong, China	69	113.31	23.49	(25)
Pou	Guangdong, China	34	112.18	23.94	(26)
Caolan	Guangxi, China	30	108.35	21.79	(26)
Zhuang	Guangxi, China	25	106.23	24.31	(26)
Zhuang	Guangxi, China	12	107.36	22.61	(26)
Sui	Guangxi, China	30	105.34	24.79	(26)
Mulam	Guangxi, China	39	108.90	24.80	(26)
Maonan	Guangxi, China	32	108.25	24.84	(26)
Palyu (Gelao)	Guangxi, China	30	105.34	24.78	(26)
Zhuang	Guangxi, China	33	109.26	25.08	(26)
Yao	Guangxi, China	15	105.83	23.40	(26)
Dong	Hubei, China	10	109.49	30.28	(26)
DornQdayc	Shanghai, China	17	121.40	31.10	(26)
Cun	Hainan, China	30	108.65	19.11	(26)
Danga	Hainan, China	40	110.04	18.51	(26)
Hlai–Qi	Hainan, China	34	109.52	18.78	(26)
Jiamao	Hainan, China	27	109.70	18.64	(26)
Lingao	Hainan, China	31	109.69	19.91	(26)
Han	Shanghai, China	120	121.48	31.28	(27)
Han	Changsha, China	82	112.95	28.25	(10)
Kam	Sanjiang, China	72	109.61	25.81	(28)
Laka	Jinxiu, China	67	110.19	24.16	(28)
Mien	Fuchuan, China	29	111.28	24.83	(28)

Mulam	Luocheng, China	27	110.80	24.66	(28)
Pinghua_Han	Hezhou, China	39	111.56	24.42	(28)
Pinghua_Han	Fuchuan, China	48	111.28	24.83	(28)
Pinghua_Han	Wuxuan, China	111	109.66	23.61	(28)
Zhuang	Hezhou, China	54	111.56	24.42	(28)
Zhuang	Luocheng, China	4	110.80	24.66	(28)
Zhuang	Jinxiu, China	9	110.19	24.16	(28)
Zhuang	Wuxuan, China	10	109.66	23.61	(28)
Philippine descent residing in Taiwan	Taiwan, China	64	121.01	15.12	(29)
Taiwanese	Taiwan, China	180	120.98	24.01	(30)
Han	Taiwan, China	155	120.89	23.43	(31)
Mulam	Luocheng, Guangxi, China	91	108.90	24.79	(32)
Han	Chaoshan, Guangdong, China	102	116.63	23.67	(33)
Han	Meizhou, Guangdong, China	170	116.13	24.31	(33)
Tujia	Western Hunan, China	66	109.73	28.41	(12)
Tujia	Yongshun, Hunan, China	31	109.85	29.03	(12)
Han	Hefei, Anhui, China	42	117.22	31.86	(13)
Han	Changtin, Fujian, China	51	116.36	25.86	(13)
Han	Tianlin, Guangxi, China	26	106.23	24.33	(13)
Han	Changsha, Hunan, China	16	112.93	28.26	(13)
Han	Nanjing, Jiangsu, China	67	118.79	32.11	(13)
Han	Nanchang, Jiangxi, China	23	115.86	28.72	(13)
Han	Shanghai, China	56	121.46	31.29	(13)
Han	Hangzhou, Zhejiang, China	61	120.16	30.31	(13)
Miao	Jishou, Hunan, China	103	109.70	28.28	(34)

Yao	Dahua, Guangxi, China	19	107.97	23.77	(34)
Yao	Liannan, Guangdong, China	35	112.29	24.74	(34)
Yao	Jianghua, Hunan, China	24	111.59	25.21	(34)
Yao	Fangcheng, Guangxi, China	19	108.36	21.80	(34)
Yao	Fuchuang, Guangxi, China	42	111.28	24.84	(34)
Yao	Tianlin, Guangxi, China	26	106.23	24.33	(34)
Yao	Tianlin, Guangxi, China	6	106.13	24.21	(34)
Yao	Shangsi, Guangxi, China	32	108.01	22.21	(34)
Yao	Tianlin, Guangxi, China	32	106.09	24.41	(34)
Yao	Hezhou, Guangxi, China	41	111.57	24.42	(34)
Yao	Fuchuang, Guangxi, China	31	111.28	24.84	(34)
Yao	Fangcheng, Guangxi, China	11	108.36	21.80	(34)
Han	Zhanjiang, Guangdong, China	30	110.36	21.29	(15)
Han	Wuhan, Hubei, China	42	114.30	30.63	(15)
**Southwest China**		**2996**			
TH	Yunnan, China	46	102.76	24.13	(16)
Dai	Dehong, China	46	101.98	23.60	(35)
Dai	Lancang, China	25	99.92	22.56	(35)
Dai	Xishuangbanna, China	30	101.98	23.60	(35)
Dai	Yuanjiang, China	27	105.06	24.07	(35)
Dai	Jianshui, Lincang, Yunnan, China	85	99.39	23.57	(35)
Dai	Dehong, Yunnan, China	51	97.92	24.29	(35)
Ai-Cham	Guizhou, China	6	107.89	25.44	(26)
Qau	Guizhou, China	42	105.28	27.33	(26)
Lolo	Guizhou, China	4	105.63	27.16	(26)

Mak	Guizhou, China	33	107.89	25.42	(26)
Mollao	Guizhou, China	29	107.59	26.50	(26)
Red-Gelao	Guizhou, China	31	105.61	27.16	(26)
Then	Guizhou, China	30	107.32	25.85	(26)
Bugan	Yunnan, China	32	104.67	23.45	(26)
Buyang	Yunnan, China	31	105.06	24.07	(26)
Dai-lu	Yunnan, China	56	100.80	22.05	(26)
Lachi	Yunnan, China	30	104.40	23.03	(26)
Pubiao	Yunnan, China	25	104.70	23.14	(26)
White-Gelao	Yunnan, China	14	104.70	23.14	(26)
Lahu	Simao, Yunnan, China	32	100.98	22.80	(36)
Tibetan	Diqing, Yunnan, China	24	99.70	27.83	(36)
Va	Simao, Yunnan, China	22	102.74	25.01	(36)
Dai	Xishuangbanna, Yunnan, China	21	100.78	21.99	(36)
Tibetan	Garze, Western Sichuan, China	55	101.96	30.06	(4)
Qiang	Bamei Town, Daofu county, Sichuan, China	43	101.12	30.98	(37)
Qiang	Danba county, Sichuan, China	47	101.89	30.88	(37)
Qiang	Xinlong County of Sichuan, China	124	99.05	32.47	(37)
Qiang	Hekou Town, Yajiang County of Sichuan, China	193	101.01	30.03	(37)
Han	Yuxi, Yunnan, China	850	102.55	24.36	(38)
Bai	Dali, Yunnan, China	40	100.27	25.62	(12)
Hani	Xishuangbanna, Yunnan, China	33	100.80	22.03	(12)
Lahu	Xishuangbanna, Yunnan, China	15	99.94	22.60	(12)
Naxi	Lijiang, Yunnan, China	45	100.23	26.88	(12)
Pumi	Ninglang, Yunnan, China	35	100.86	27.32	(12)

Tibetan	Zhongdian, Yunnan, China	35	103.23	25.23	(12)
Yi	Xishuangbanna, Yunnan, China	56	101.64	27.71	(12)
Aini	Xishuangbanna, Yunnan, China	47	100.64	22.12	(12)
Bai	Xishuangbanna, Yunnan, China	19	100.88	21.87	(12)
Jino	Xishuangbanna, Yunnan, China	18	101.32	21.73	(12)
Han	Weicheng, Sichuan (SH), China	70	104.96	31.55	(13)
Han	Huize, Yunnan (SH), China	58	103.29	26.47	(13)
Miao	Wenshan, Yunnan (Hmong), China	39	104.25	23.38	(34)
Yao	Mengla, Yunnan (Mien), China	10	101.58	21.50	(34)
Yao	Malipo, Yunnan (Mien), China	40	104.69	23.14	(34)
Yao	Mengla, Yunnan (Mien), China	27	101.58	21.50	(34)
Tibetan	Deqian, Diqing, Yunnan; Qinghai, China	40	99.70	27.83	(39)
Lahu	Lancang, Yunnan, China	35	99.94	22.59	(40)
Va	Ximeng and Gengma, Yunnan, China	36	102.74	25.01	(40)
Han	Kunming, Yunnan, China	43	102.72	25.06	(15)
Dai	Xishuangbanna, Yunnan, China	38	100.60	22.00	(39)
Tibetan	Diqing, Yunnan, China	71	99.70	27.82	(5)
Tibetan	Liangshan, Sichuan, China	62	102.27	27.89	(5)
**North Asia**		**4451**			
Ba	Bashkir	221	56.15	54.32	(41)
Ch	Chuvash	55	46.93	55.71	(41)
Er	Erza-Moksha	102	50.47	53.01	(41)
Ko	Komi	138	52.60	62.89	(41)
Ma	Mari	139	48.12	56.68	(41)
Ta	Tatar	228	50.71	55.54	(41)

Ud	Udmurt	102	57.00	55.94	(41)
Kets	Sulomai and Turukhansk	38	81.70	70.71	(42)
Ngana-sans	Dudinka	24	82.00	70.19	(42)
Mansi	Northwest Siberia	98	61.40	60.63	(43)
Even	Even	65	159.50	57.56	(44)
Koryak	Koryak	35	164.40	64.26	(44)
Yakutian	Yakut	22	129.73	62.04	(44)
Buryat	South Siberia	40	112.81	54.39	(45)
Tuvinian	South Siberia	36	95.59	52.15	(45)
Altaian	South Siberia	110	86.10	50.99	(46)
Khakassian	South Siberia	53	87.50	54.91	(46)
Buryat	South Siberia	91	110.50	53.38	(46)
Sojot	South Siberia	30	105.90	56.58	(46)
Todjin	South Siberia	48	96.60	53.28	(46)
Tuvinian	South Siberia	90	95.40	51.75	(46)
Tofalar	South Siberia	58	97.73	54.62	(46)
Yakut	Northeastern Siberia	191	129.76	62.06	(47)
Mongoloid	Mongolian	46	102.77	50.00	(48)
AEG	Mongolia	39	103.52	49.87	(49)
PDEG	Mongolia	124	103.44	49.51	(49)
PDM	Mongolia	104	102.56	49.02	(49)
PDY	Mongolia	44	103.74	48.76	(49)
Mongoloid	Mongolia	103	101.62	45.72	(50)
Russian	North-Eastern Siberia	50	150.77	59.71	(51)
Ukrainian	North-Eastern Siberia	18	150.66	59.86	(51)
Buryat	Ulan-Ude, South-Central Siberia	126	107.63	51.97	(52)

Yakut	Yakutsk, northeastern Siberia	117	129.73	62.04	(52)
Khanty	Lower Ob-River Valley, Siberia	106	66.69	66.23	(53)
Mansi	Lower Ob-River Valley, Siberia	63	67.70	66.83	(53)
TB	Southern Siberia	72	88.75	51.84	(54)
TV	Southern Siberia	96	91.00	51.23	(54)
BR	Southern Siberia	25	99.19	54.65	(54)
TF	Southern Siberia	46	96.98	54.64	(54)
EV	Southern Siberia	37	94.68	62.00	(54)
NG	Southern Siberia	33	60.60	59.15	(54)
UL	Southern Siberia	87	134.85	51.14	(54)
NV	Southern Siberia	56	135.23	48.35	(54)
UD	Southern Siberia	46	136.12	47.87	(54)
Tubalar	Altai region, Siberia	144	86.22	50.62	(55)
Evens	NE Siberia, Siberia	87	140.46	64.98	(55)
Ulchi	Lower Amur R., Siberia	160	138.52	52.71	(55)
Buryat	Buryat, Buryat	61	107.90	54.70	(19)
Even	Even, Siberia	35	159.65	55.39	(19)
Nga	Arctic Siberians	39	94.50	71.26	(56)
Ind	Arctic Siberians	82	146.73	69.47	(56)
Kolyma	Arctic Siberians	18	151.26	65.76	(56)
Chv	Arctic Siberians	32	170.41	64.67	(56)
Chu	Arctic Siberians	182	171.80	66.70	(56)
Sir	Arctic Siberians	34	-173.96	64.45	(56)
Cha	Arctic Siberians	50	-172.26	64.41	(56)
Nau	Arctic Siberians	39	-171.02	65.60	(56)
Ale	Arctic Siberians	36	166.03	55.24	(56)

**Southeast Asia**		**8040**			
Burmans	Sagaing, Myanmar	32	95.43	21.83	(57)
Burmans	Sagaing, Myanmar	54	94.05	23.36	(57)
Burmans	Sagaing, Myanmar	51	94.56	23.84	(57)
Burmans	Magway, Myanmar	122	94.58	20.56	(57)
Burmans	Bago, Myanmar	69	95.74	17.78	(57)
Burmans	Ayeyarwady, Myanmar	72	95.25	18.35	(57)
Chin	Chin, Myanmar	58	93.69	23.48	(57)
Chin	Magway, Myanmar	187	94.26	20.15	(57)
Chin	Chin, Myanmar	13	93.45	21.56	(57)
Naga	Sagaing, Myanmar	30	94.92	25.50	(57)
Naga	Sagaing, Myanmar	39	94.98	25.18	(57)
Naga	Sagaing, Myanmar	32	95.30	25.90	(57)
Rakhine	Magway, Myanmar	24	94.10	21.70	(57)
Rakhine	Rakhine, Myanmar	63	94.78	18.69	(57)
Akha	Chiang Rai, Thailand	91	99.72	19.91	(58)
Lahu	Chiang Mai, Thailand	39	99.72	19.91	(58)
Lisu	Chiang Mai, Thailand	25	99.72	19.91	(58)
Lisu	Chiang Rai, Thailand	42	99.72	19.91	(58)
Lisu	Mae Hong Son, Thailand	53	97.87	18.78	(58)
Mussur	Chiang Mai, Thailand	21	99.72	19.91	(58)
Vietnamese	Hanoi area, Vietnam	187	105.83	21.04	(59)
Cham	Vietnam, Vietnam	168	108.14	11.26	(60)
Kinh	Vietnam, Vietnam	139	105.83	21.06	(60)
Thai	Chiang Mai, Thailand	190	99.72	19.91	(61)

Thai	North Thailand	32	99.58	19.61	(39)
Cambodia	Northwest Cambodia	31	104.06	13.91	(62)
Thai	Chiang Mai, Thailand	30	99.72	19.91	(63)
Thai	Khon Kaen, Thailand	44	102.62	16.04	(63)
Lao Song	Chiang Rai/Mae Hong Son, Thailand	25	97.87	18.78	(63)
ChB	Nakhon Ratchasima, Thailand	20	102.25	14.93	(64)
Khm	Nakhon Ratchasima, Thailand	22	102.25	14.93	(64)
Th_K	Nakhon Ratchasima, Thailand	32	102.25	14.93	(64)
Phuthai	Nakhon Pathom, Thailand	25	100.17	13.99	(63)
Chong	Chanthaburi, Thailand	25	102.16	12.82	(63)
Java	Java	46	110.70	-7.36	(24)
Banjarmasin	Banjumas, Indonesian	89	114.59	-3.33	(24)
Borneo	Borneo, Malaysia	61	116.12	5.98	(24)
Manado	Sulawesi, Indonesian	89	124.85	1.51	(24)
Palu	Sulawesi, Indonesian	38	119.85	-0.88	(24)
Ujung Padang	Sulawesi, Indonesian	46	99.45	3.02	(24)
Toraja	Sulawesi, Indonesian	64	120.04	-2.87	(24)
Bali	Balinese, Indonesian	82	115.21	-8.34	(24)
Mataram	Mataram, Indonesian	44	116.12	-8.58	(24)
Flores	Flores, Indonesian	2	121.10	-8.58	(24)
Waingapu	Sumba, Indonesian	50	120.02	-9.58	(24)
Alor	Alor, Malaysia	45	100.37	6.13	(24)
Ambon	Ambon, Indonesian	43	128.17	-3.64	(24)
Bangka	Indonesian/Sumatrans	34	101.32	0.02	(65)
Batek	Indonesian/Sumatrans	45	101.96	5.62	(65)
Jahai	Indonesian/Sumatrans	39	101.37	5.73	(65)

Medan	Indonesian/Sumatrans	42	98.67	3.64	(65)
Melayu	Indonesian/Sumatrans	6	114.49	-8.24	(65)
Mendriq	Indonesian/Sumatrans	31	102.03	5.23	(65)
Padang	Indonesian/Sumatrans	24	100.39	‘-0.921	(65)
Palembang	Indonesian/Sumatrans	50	104.76	-2.98	(65)
Pekanbaru	Indonesian/Sumatrans	52	101.45	0.57	(65)
Semai	Senoi, Malaysia	6	101.02	4.67	(65)
Semelai	Aboriginal Malay, Malaysia	61	102.40	3.44	(65)
Temiar	Senoi, Malaysia	46	100.83	5.41	(65)
Temuan	Aboriginal Malay, Malaysia	33	101.67	3.57	(65)
Philippines	Philippine	61	121.35	17.39	(24)
Philippine	Philippine	29	122.94	12.68	(19)
Philippine	Philippine	30	121.03	16.13	(19)
Malaysian-MAL	Malaysian	52	101.69	3.15	(19)
Indonesian	Indonesian	54	111.00	-0.60	(19)
Luzon	National Capital Philippine	168	121.32	16.85	(29)
Visayas	Cebu Philippine	103	121.04	14.66	(29)
Mindanao	Zamboanga Philippine	70	122.90	7.96	(29)
Mal	Kuala Lumpur, Malaysia	124	101.69	3.13	(66)
Aita	Bougainville	54	155.08	-5.90	(67)
An êm	New Britain	19	155.08	-5.45	(67)
Ata	New Britain	58	155.08	-5.64	(67)
Buka	Bougainville	15	155.08	-5.25	(67)
Eastern Highlands	New Guinea	4	155.08	-6.08	(67)
Eivo	Bougainville	19	155.08	-6.18	(67)
Fiji	Fiji	15	155.08	-18.00	(67)

Fringe Highlands	New Guinea	18	155.08	-5.73	(67)
Kaket (Baining)	New Britain	59	155.08	-4.50	(67)
Kol	New Britain	57	155.08	-5.38	(67)
Kove	New Britain	20	155.08	-5.47	(67)
Kuot	New Ireland	62	155.08	-3.03	(67)
Lavongai	New Hanover	18	155.08	-2.50	(67)
Lowland Riverine	New Guinea	27	155.08	-8.50	(67)
Madak	New Ireland	31	155.08	-3.10	(67)
Malaita - Baegu	Solomon Islands	134	155.08	-8.78	(67)
Malaita - Kwaio	Solomon Islands	54	155.08	-8.78	(67)
Malaita -Lau	Solomon Islands	57	155.08	4.00	(67)
Mali (Baining)	New Britain	58	155.08	-4.63	(67)
Mamusi	New Britain	63	155.08	-5.80	(67)
Mangseng	New Britain	17	155.08	-5.93	(67)
Manus	Manus	2	155.08	-1.86	(67)
Markham	New Guinea	67	155.08	-6.73	(67)
Melamela	New Britain	23	155.08	-5.00	(67)
Mengen	New Britain	23	155.08	-5.10	(67)
Micronesia	Micronesia	47	155.08	-2.71	(67)
Misima	PNG Island	7	155.08	5.79	(67)
Morobe Highlands	New Guinea	20	155.08	-7.75	(67)
Mussau	Mussau	16	155.08	-1.58	(67)
Nagovisi	Bougainville	16	155.08	-6.43	(67)
Nakanai	New Britain	64	155.08	-5.75	(67)
Nakanai (Loso)	New Britain	17	155.08	-5.48	(67)
Nalik	New Ireland	24	155.08	-2.98	(67)

Nasioi	Bougainville	33	155.08	-6.48	(67)
New Caledonia	New Caledonia	25	155.08	-22.00	(67)
North Coast	New Guinea	14	155.08	-3.58	(67)
Notsi	New Ireland	23	155.08	-3.05	(67)
Rigo	New Guinea	18	155.08	-9.80	(67)
Rossel	PNG Island	5	155.08	25.00	(67)
Rotokas	Bougainville	19	155.08	-6.18	(67)
Saposa	Bougainville	25	155.08	-5.58	(67)
Simeku	Bougainville	7	155.08	-6.18	(67)
Siwai	Bougainville	19	155.08	-6.67	(67)
Solomon Islands	Solomon Islands	69	155.08	159.15	(67)
Solomons	Solomon Islands	28	155.08	159.21	(67)
Southwest Riverine	New Guinea	33	155.08	141.60	(67)
Sulka	New Britain	28	155.08	-4.89	(67)
Teop	Bougainville	20	155.08	-5.85	(67)
Tigak	New Ireland	27	155.08	-2.60	(67)
Tolai	New Britain	78	155.08	-4.34	(67)
Torau	Bougainville	46	155.08	-6.17	(67)
Vanuatu	Vanuatu	23	155.08	-16.00	(67)
Western Highlands	New Guinea	18	155.08	-5.90	(67)
HA	Singapore	205	103.78	1.37	(68)
Sakai	Trang Thailand	20	99.61	7.70	(63)
Mussur	Chiang Mai Thailand	21	98.69	19.05	(63)
White Karen	Mae Hong Son Thailand	40	97.88	18.77	(58)
Red Karen	Mae Hong Son Thailand	39	97.91	18.85	(58)
BK	Northeast Thailand	13	102.35	17.53	(64)

NUL	Northeast Thailand	17	103.00	17.57	(64)
Benu	Benut, Johor, Malaysia	10	103.30	1.68	(69)
Gopeng	Gopeng, Perak, Malaysia	22	101.17	4.46	(69)
Kuala Kurau	Kuala Kurau, Perak, Malaysia	17	100.41	5.03	(69)
Lembah Bujang	Lembah Bujang, Kedah, Malaysia	26	100.59	5.91	(69)
Lenggeng	Lenggeng, Negeri Sembilan, Malaysia	12	101.93	2.89	(69)
Machang	Machang, Kelantan, Malaysia	24	102.22	5.77	(69)
Kota Bharu	Kota Bharu, Kelantan, Malaysia	5	102.25	6.13	(69)
Muar	Muar, Johor, Malaysia	17	102.57	2.04	(69)
Parit Buntar	Parit Buntar, Perak, Malaysia	16	100.48	5.11	(69)
Parit Buntar	Parit Buntar, Perak, Malaysia	1	100.49	5.11	(69)
Pontian	Pontian, Johor, Malaysia	20	103.40	1.49	(69)
Rantau Panjang	Rantau Panjang, Kelantan, Malaysia	32	101.98	6.02	(69)
Semerah	Semerah, Johor, Malaysia	13	102.77	1.89	(69)
Sri Menanti	Sri Menanti, Negeri Sembilan, Malaysia	22	102.16	2.71	(69)
Yan	Yan, Kedah, Malaysia	11	100.39	5.84	(69)
Moor	Sri Lanka	43	80.97	6.89	(70)
Sinhalese	Sri Lanka	59	80.44	7.46	(70)
Laos	Laos	214	102.61	19.86	(71)
Brao	Cambodia	123	106.85	13.92	(72)
Jarai	Cambodia	102	107.24	13.44	(72)
Kachac	Cambodia	33	107.23	14.02	(72)
Khmer	Cambodia	80	106.22	12.43	(72)
Kravet	Cambodia	71	106.74	13.86	(72)
Kreung	Cambodia	46	106.77	13.96	(72)
Kuy	Cambodia	137	105.62	13.32	(72)

Lao	Cambodia	91	106.05	13.34	(72)
Lun	Cambodia	38	106.95	13.91	(72)
Mel	Cambodia	107	105.77	12.72	(72)
Phnong	Cambodia	63	105.87	12.81	(72)
Stieng	Cambodia	50	106.39	11.96	(72)
Tompuon	Cambodia	111	107.35	13.55	(72)
Kraol	Cambodia	2	106.33	12.46	(72)
Myanmar	Myanmar	327	96.06	21.89	(73)
Batak	Philippine	31	118.65	9.97	(74)
Palawan Non-Negrito	Philippine	20	118.65	9.97	(74)
**South Asia**		**6760**			
Adi	Assam, India	45	92.92	26.30	(75)
Apatani	Arunachal Pradesh, India	26	94.06	28.31	(75)
Apatani	Tripura, India	21	91.99	23.99	(75)
Naga	Nagaland, India	43	94.72	26.19	(75)
Nishi	Tripura, India	44	91.99	23.99	(75)
Tipperah	Tripura, India	20	91.99	23.99	(76)
Garo	Meghlaya, India	76	90.55	25.58	(77)
Lyngnga	Meghlaya, India	74	90.92	25.38	(77)
Nongtrai	Meghlaya, India	27	91.13	25.85	(77)
Maram	Meghlaya, India	60	91.23	25.50	(77)
Bhoi	Meghlaya, India	29	91.75	25.96	(77)
Khynriam	Meghlaya, India	82	91.79	25.54	(77)
War_Khas	Meghlaya, India	29	91.94	25.21	(77)
Pnar	Meghlaya, India	51	92.31	25.47	(77)

War_Jaint	Meghlaya, India	17	92.32	25.20	(77)
Pakistania	Southeast Pakistan	100	67.04	25.96	(78)
Baluch	Baluchistan, Southwestern Pakistan	39	67.59	30.39	(78)
Brahui	Baluchistan, Southwestern Pakistan	38	62.47	29.06	(78)
Gujarati	Gujarat, Northwestern India	34	69.35	25.15	(78)
Hazara	North West Frontier Province and Balochistan, Pakistan	23	71.93	33.38 7	(78)
Hunza Burusho	Karakorum Mountains, Northern Pakistan	44	76.27	35.25	(78)
Kalash	North West Frontier Province, Pakistan	44	72.54	36.30	(78)
Makrani	Makran Coast, South Pakistan	33	63.30	26.40	(78)
Parsi	Karachi, Southeastern Pakistan	44	69.00	26.20	(78)
Pathan	North West Frontier Province and Balochistan, Pakistan	44	74.56	32.39	(78)
Sindhi	Sindh, Southeastern Pakistan	23	71.74	29.35	(78)
Ha, S, SHC, SST, SR, TA, Tamil nadu,	Tamil Nadu, India	74	79.26	11.61	(79-81)
Baduga (Bad)	Tamil Nadu, India	2	78.62	11.45	(81)
Irula, Ir	Tamil Nadu, India	16	78.63	11.03	(76, 81)
Kota	Tamil Nadu, India	25	78.48	11.44	(75)
Kurumba	Tamil Nadu, India	8	78.11	11.75	(75)
Kurumba Betta	Tamil Nadu, India	19	79.17	11.96	(75)
Kurumba Mullu, Mulla Krumba	Tamil Nadu, India	15	79.26	11.61	(75)
Oorali (Oo)	Tamil Nadu, India	3	79.43	11.20	(81)
Sakkili (Sak)	Tamil Nadu, India	6	78.85	11.19	(81)
Soligas	Tamil Nadu, India	12	78.74	12.03	(75)

Kadar	Kerala, India	7	76.46	10.75	(82)
Malayalam (ML)	Kerala, India	3	76.73	9.75	(80)
Cochin	Kerala, India	52	77.40	9.24	(70)
Cochin Jews	Kerala, India	37	76.72	9.33	(70)
Kattunaiken	Kerala, India	16	76.85	8.95	(75)
Kuruchian	Kerala, India	46	76.67	9.93	(75)
Kuruman Mullu, Mullu Kurunan	Kerala, India	37	76.50	10.29	(75)
Paniya	Kerala, India	11	76.16	11.10	(75)
Havik	Karnataka, India	38	75.72	15.58	(82)
Mukri	Karnataka, India	42	75.57	14.91	(82)
Kannada (KA)	Karnataka, India	5	75.99	14.26	(80)
Koragas	Karnataka, India	31	75.95	13.70	(75)
Kuruva	Karnataka, India	25	77.03	13.19	(83)
Kurumba Jenu	Karnataka, India	6	76.40	12.64	(75)
Yerava	Karnataka, India	53	76.10	13.21	(75)
Bhovi	Karnataka, India	30	77.01	13.56	(84)
Gowda	Karnataka, India	37	76.16	14.51	(84)
Brahmin	Karnataka, India	29	75.34	14.35	(84)
Lingayat	Karnataka, India	17	76.22	13.94	(84)
Christians	Karnataka, India	21	76.09	13.38	(83)
Muslim	Karnataka, India	24	75.00	13.85	(83)
Siddi	Andhra Pradesh, India	7	79.88	17.33	(85)
Telugu	Andhra Pradesh, India	15	80.49	17.03	(80, 86, 87)
Lambadi	Andhra Pradesh, India	75	80.29	17.30	(86)
Andh, AndhSA, AndhX	Andhra Pradesh, India	95	80.00	17.69	(88)

Brahmin	Andhra Pradesh, India	34	79.42	17.41	(86)
Kapu	Andhra Pradesh, India	67	79.37	16.74	(86, 89)
Madiga	Andhra Pradesh, India	24	80.27	15.90	(86)
Yadava	Andhra Pradesh, India	40	79.76	16.01	(86)
Wadabahija	Andhra Pradesh, India	8	81.69	17.05	(86)
Mala	Andhra Pradesh, India	23	79.13	17.66	(86)
Kshatriya	Andhra Pradesh, India	9	78.76	17.15	(86)
Relli	Andhra Pradesh, India	20	78.37	17.35	(86)
Jalari	Andhra Pradesh, India	7	78.82	18.23	(86)
Vysya	Andhra Pradesh, India	9	77.99	16.14	(86)
Chenchu	Andhra Pradesh, India	96	78.05	16.68	(90)
Koya	Andhra Pradesh, India	81	79.07	18.28	(90)
Erukula(Er)	Andhra Pradesh, India	5	78.38	18.90	(81)
Yanadi(Yn)	Andhra Pradesh, India	10	79.22	15.89	(81)
Pardhi	Andhra Pradesh, India	38	78.37	17.35	(75)
Thoti	Andhra Pradesh, India	39	78.82	18.23	(75)
Akhutota(AK)	Andhra Pradesh, India	22	77.99	16.14	(89)
Panta(PK)	Andhra Pradesh, India	29	78.05	16.68	(89)
Pokanati(PO)	Andhra Pradesh, India	36	79.07	18.28	(89)
Vanne(VN)	Andhra Pradesh, India	26	78.38	18.90	(89)
Irula	Andhra Pradesh, India	19	79.22	15.89	(91)
Chenchu	Andhra Pradesh, India	75	78.05	16.68	(92)
Kolam	Andhra Pradesh, India	128	79.07	18.28	(92)
Gond	Andhra Pradesh, India	75	78.38	18.90	(92)
Naikpod	Andhra Pradesh, India	83	79.37	16.74	(88)
Pardhan	Andhra Pradesh, India	170	80.27	15.90	(88)

Chitpavan Brahmin	Maharashtra, India	17	75.66	20.05	(93)
Desasth Brahmin	Maharashtra, India	15	75.30	19.38	(93)
Brahmin	Maharashtra, India	9	76.54	20.19	(94)
Konkanastha Brahmin	Maharashtra, India	50	77.64	20.43	(70)
Dhangar	Maharashtra, India	16	76.54	20.43	(93)
Maratha	Maharashtra, India	30	75.50	20.48	(93-95)
Maharashtra, Marathi	Maharashtra, India	29	76.22	18.75	(80, 86)
Nav-Baudh	Maharashtra, India	17	76.35	19.17	(94, 95)
Parsi	Maharashtra, India	37	76.54	20.19	(70)
Bohra	Maharashtra, India	5	77.64	20.43	(95)
Irani	Maharashtra, India	5	76.54	20.43	(95)
Korku	Maharashtra, India	9	75.50	20.48	(95)
Madia Gond	Maharashtra, India	13	76.22	18.75	(95)
Kolam	Maharashtra, India	8	76.35	19.17	(95)
Kathodi (Ka)	Gujarat, India	1	71.18	22.58	(81)
Koli (Ko)	Gujarat, India	6	70.92	21.99	(81)
Gujarat (Guj, GJ)	Gujarat, India	61	70.65	22.33	(70, 78)
Brahmin	Punjab, India	29	72.67	31.44	(96)
Khatri	Punjab, India	11	72.96	30.90	(96)
Jat Singh	Punjab, India	27	72.67	31.28	(70, 96)
Sikh	Punjab, India	23	72.89	31.14	(75)
Scheduled caste	Punjab, India	19	73.10	31.31	(70, 96)
Lobana	Punjab, India	49	73.11	31.52	(86)
Brahmin (Puj)	Punjab, India	18	72.84	31.56	(70)
Kshatriya	Punjab, India	22	73.02	31.64	(70)
Punjab	Punjab, India	42	73.37	31.72	(79, 87)

Bhoksa	Uttar Pradesh, India	22	80.09	27.78	(86)
Tharu	Uttar Pradesh, India	31	79.62	27.87	(70, 86)
Munda (Mun)	Uttar Pradesh, India	4	79.90	28.11	(81)
Kori (Kor)	Uttar Pradesh, India	1	80.04	27.35	(81)
Rohidas (Ro)	Uttar Pradesh, India	1	80.96	27.32	(81)
Yadava (Yd)	Uttar Pradesh, India	6	79.22	27.38	(81)
Uttar Pradesh Brahmin	Uttar Pradesh, India	23	81.00	27.65	(70)
Rajput (Rj)	Uttar Pradesh, India	14	80.95	26.87	(81)
Lodhe (Lo)	Uttar Pradesh, India	1	80.35	26.31	(81)
HI, U, Uttar Pradesh, USIN, UKIR, UANI, UBHA, UBOB, UCHA, UD, UHC, UJEA	Uttar Pradesh, India	93	81.27	26.21	(79, 80, 86)
Delhi	Delhi, India	10	77.23	28.65	(87)
Kanet	Kanet, India	29	72.40	23.00	(70)
Rajput, Rajsthan	Rajput, Rajasthan, India	33	76.60	30.49	(70, 86)
Kashmir	Kashmir, India	13	76.16	34.01	(86)
Baluch	Pakistan, Pakistan	14	67.97	31.70	(78)
Brahui	Pakistan, Pakistan	12	66.38	30.88	(78)
Hazara	Pakistan, Pakistan	12	69.28	32.52	(78)
Hunza Burusho	Pakistan, Pakistan	23	78.22	34.85	(78)
Karachi (KAR, Pakistan, UR)	Pakistan, Pakistan	74	67.04	25.00	(78, 80, 86)
Parsi	Pakistan, Pakistan	23	70.50	25.50	(78)
Pathan	Pakistan, Pakistan	23	73.71	32.30	(78)
Sindhi (SI)	Pakistan, Pakistan	14	71.24	28.79	(78)
Pushtoons	Pakistan, Pakistan	16	72.03	33.58	(75)
Makrani	Pakistan, Pakistan	8	62.31	25.90	(78)

BI, BD, BHC, BSUY, BVIJ, Bihar	Bihar, India	43	85.27	26.07	(79, 80)
Bhumij (BhuJ, BM)	Bihar, India	60	85.36	25.88	(96, 97)
Ho	Bihar, India	20	85.11	25.52	(97)
Kharia (KhaJ, KHA)	Bihar, India	39	85.51	25.67	(89, 97)
Kanwar (Kan)	Bihar, India	6	84.91	25.90	(81)
Satnami (Sat)	Bihar, India	3	84.87	26.03	(81)
Santhal (Sa, SA)	Bihar, India	45	85.81	26.02	(81, 89)
Asur (AS)	Bihar, India	30	85.84	25.76	(89)
Munda (MU)	Bihar, India	23	85.32	25.73	(89)
Bharia (Bh)	Madhya Pradesh, India	14	78.68	23.44	(81)
Gond (Go), Madia Gond	Madhya Pradesh, India	4	78.67	22.98	(81, 91)
Oran (Or)	Madhya Pradesh, India	16	79.54	23.26	(81)
Muria	Madhya Pradesh, India	12	80.08	23.42	(76)
Brahmin	Orissa, India	16	84.25	20.49	(98)
Gope	Orissa, India	17	84.25	20.91	(98)
Juang	Orissa, India	20	83.74	20.20	(98)
Karan	Orissa, India	14	83.55	20.70	(98)
Khandayat	Orissa, India	14	83.98	21.24	(98)
Paroja	Orissa, India	21	83.34	20.89	(98)
Saora	Orissa, India	17	85.42	20.67	(98)
Orisa	Orissa, India	2	85.91	20.30	(86)
Bengali	West Bengal, India	15	87.90	23.24	(80, 81, 86)
Bengal Brahmin	West Bengal, India	36	88.22	22.76	(70)
Bengal Sudra	West Bengal, India	8	87.65	22.82	(70)
Kurmi	West Bengal, India	55	88.01	23.35	(70)

Lodha	West Bengal, India	68	87.65	22.95	(70, 76)
Munda	West Bengal, India	6	87.28	22.50	(76)
Santhal	West Bengal, India	14	88.65	22.43	(76)
Bangladesh	Bangladesh	1	90.31	24.19	(86)
Bangladesh	Bangladesh	27	90.07	24.39	(75)
Great Andamanese	Andaman Archipelago, India	20	92.82	12.65	(99)
Jarawa	Andaman Archipelago, India	4	92.82	12.56	(99)
Onge	Andaman Archipelago, India	62	92.65	12.00	(99)
Nicobarese	Andaman Archipelago, India	46	92.66	11.81	(99, 100)
Ao	Nagaland, India	19	94.57	26.23	(81)
Ladakhis-Bud	Ladak, Kashmir	23	76.41	33.64	(101)
Ladakhis-Mus	Ladak, Kashmir	9	76.57	33.77	(101)
Shia Muslim	India	120	80.47	26.54	(102)
Sunni Muslim	India	131	79.52	27.05	(102)
Dawoodi Bohra (TN)	India	62	78.16	10.47	(102)
Dawoodi Bohra (GUJ)	India	50	71.50	23.02	(102)
Mappla	India	61	77.94	9.73	(102)
Iranian Shia	India	48	78.84	16.48	(102)
Tharu-C I	Nepal	57	84.46	27.72	(103)
Tharu-C II	Nepal	76	84.38	27.83	(103)
Tharu-E	Nepal	40	87.71	26.88	(103)
Nepal-Kathmandu	Kathmandu, Nepal	200	85.32	27.74	(104)
Nepal-East Nepal	East Nepal, Nepal	46	87.99	26.70	(104)
Tamang	Nepal	45	84.94	28.05	(1)
Newar	Nepal	66	86.07	27.80	(1)
Kathmandu	Nepal	77	85.33	27.69	(1)

Chakma	Chittagong hill tract, Bangladesh	108	91.83	22.40	(105)
Marma	Chittagong hill tract, Bangladesh	97	91.80	22.37	(105)
Tripura	Chittagong hill tract, Bangladesh	97	91.82	22.29	(105)
Bengali	Lalmanir Hat, Bangladesh	64	87.13	52.34	(106)
Bengali	Dinajpur, Bangladesh	176	87.24	52.19	(106)
**Central and west Asia**	**4601**				
B-Arab	Bukharan Arabs	20	65.83	38.66	(107)
C-Tatar	Crimean Tatars	20	34.28	45.38	(107)
Dungan	Dungans	16	73.39	39.83	(107)
Iranian	Iranians	20	53.99	35.35	(107)
Karakalpak	Karakalpaks	20	59.48	42.78	(107)
Kazak	Kazaks	20	69.24	41.77	(107)
K-Uzbek	Khoremian Uzbeks	20	61.69	41.77	(107)
Kyrgyz	Kyrgyz	20	72.21	42.06	(107)
Tajik	Tajiks	20	68.93	37.94	(107)
Turkmen	Turkmen	20	61.66	38.69	(107)
Uighur	Uighurs	16	78.48	42.26	(107)
Uzbek	Uzbeks	20	67.75	40.46	(107)
Kazak	Kazakh, Kazakhstan	55	79.10	44.57	(108)
KIR	Kirghiz, Kirghizstan	47	71.58	42.64	(108)
KIT	Kirghiz, Kirghizstan	48	72.65	39.67	(108)
UIG	Uighur, Kazakhstan	55	82.81	47.52	(108)
Kurdish	Turkmenistan	32	59.18	40.20	(78)
Lur	Southwestern Iran (Zagros Mountains)	17	52.10	30.40	(78)
Mazandarian	Northern Iran, southeastern Caspian Sea area	21	56.36	37.56	(78)

Persian	Central and southern central Iran	42	55.17	33.50	(78)
Shugnan	High Pamirs, Tajikistan	44	73.84	38.18	(78)
Turkmen	Turkmenistan	41	62.80	38.50	(78)
Turkish	Mostly eastern and western Azerbaijan	40	47.48	38.70	(78)
Uzbek	Surkhandarya, Uzbekistan	42	65.48	41.15	(78)
Turkisha	Anatolia, Turkey	50	35.62	38.98	(78)
FER	Fergana, Uzbekistan	53	71.71	41.05	(109)
KAR	Karakalpakstan, Uzbekistan	46	60.68	41.70	(109)
KAZ	Kazakhstan, Kazakhstan	256	70.43	41.95	(109)
KYR	Kyrgyzstan, Kyrgyzstan	249	71.62	40.86	(109)
QAS	Qashkadarya, Uzbekistan	75	67.16	38.52	(109)
Gilaki	Northern Iran, southwestern Caspian Sea area	37	51.61	36.30	(78)
Kurdish	Western Iran	20	47.97	33.80	(78)
Afghanistan	Afghanistan	98	67.85	37.98	(109)
Tajikistan	Tajikistan	244	68.95	39.42	(109)
Tashkent	Uzbekistan	55	69.38	41.39	(109)
Turkmenistan	Turkmenistan	249	66.86	38.06	(109)
Khorezm	Uzbekistan	99	61.02	41.48	(109)
Karakalpaks Qongirat	Uzbekistan	55	59.39	43.77	(110)
Kazakhs	Kazakhstan	50	59.03	43.72	(110)
OTU	Uzbekistan/Turkmenistan border	53	62.11	41.40	(110)
Turkmen	Uzbekistan/Turkmenistan border	51	62.94	41.03	(110)
Uzbeks	Karakalpakia	40	59.56	43.66	(110)
Kazakhstan	Kazakhstan	27	67.30	48.34	(111)
Chelkan	Northern Altaians	91	87.07	52.47	(112)
Kumandin	Northern Altaians	52	87.13	52.34	(112)

Tubalar	Northern Altaians	71	87.24	52.19	(112)
Altai-kizhi	Southern Altaians	276	87.65	51.87	(112)
Altaian Kazakhs	Kosh-Agach district of Altai Republic	98	87.04	50.00	(17)
Persians	Eastern Iran	82	55.35	37.41	(113)
Kurds	Northwestern Iran	25	48.10	38.44	(113)
Tajiks	Tajikistan	44	72.42	38.63	(113)
Koreans	South Korea	103	127.90	37.09	(113)
Mongolians	Ulaanbaatar, Mongolia	47	106.90	47.93	(113)
Kalmyks	Kalmyk Republic	110	45.80	46.78	(113)
Buryats	Buryat Republic	295	110.30	54.93	(113)
Khamnigans	Buryat Republic	99	109.84	52.17	(113)
Tuvinians	Tuva Republic	105	95.63	51.96	(113)
East Evenks	Buryat Republic	45	113.82	56.29	(113)
West Evenks	Krasnoyarsk	73	92.99	56.18	(113)
Yakuts	Sakha	36	124.40	67.44	(113)
Shors	Kemerovo	82	86.05	55.36	(113)
Khakassians	Khakassian Republic	57	90.38	53.21	(113)
Altaians-Kizhi	South Altai	90	85.22	51.34	(113)
Telenghits	South Altai	71	85.32	50.98	(113)
Teleuts	Kemerovo	53	86.08	55.35	(113)
Chukchi	Anadyr, Chukotka Autonomous Okrug	15	177.49	64.73	(113)
BAL	Georgia	20	43.12	42.27	(78)
CHE	Georgia	18	43.23	41.98	(78)
GEO	Georgia	20	44.20	42.13	(78)
**Europe**		**6139**			

RUS	Russia	151	69.27	41.30	(109)
Aznakaevo	Aznakaevo, Russia	71	53.09	55.05	(114)
Buinsk	Buinsk, Russia	125	48.28	54.97	(114)
Pol	Northern Poland	436	18.83	54.12	(115)
Rus	Russia	201	41.98	45.06	(115)
Etruscans	Tuscany, Italy	322	11.25	43.77	(116)
T2DM patients	Marche, Italy	466	13.36	43.05	(117)
Controls	Marche, Italy	438	13.36	43.05	(117)
Patients of sporadic prostate cancer	Patients of sporadic prostate cancer, Southwest Europe	239	-3.60	37.18	(118)
Controls of sporadic prostate cancer	Southwest Europe	150	-3.60	37.18	(118)
Belorussian	Belarus	55	27.49	53.81	Belyaeva et al. GenBank AY005336-AY005390
Patients with schizophrenia and bipolar Disorder	Italy	89	12.29	45.21	(119)
Ancient Cumanians	Hungary	11	19.87	46.53	(120)
Nefedjevo inhabitant	Russia	8	32.92	52.05	Buzhilova et al. GenBank AF466686-AF466693
Italian	Trexenta, Sardinia, Italy	47	9.10	39.57	Calo et al. GenBank DQ081669-DQ081715
Autochthonous Basque lineages	Spain	51	-4.27	40.50	Cardoso et al. GenBank AN. JX669021-JX669071
Autochthonous Basque lineages	Spain	56	-4.27	40.50	Cardoso et al. GenBank AN. JX669072-JX669127
Spanish	Pas Valley, Spain	61	-3.85	43.20	(121)

Basque	Navarre, Spain	110	-1.73	42.62	(122)
Basque	Northern Navarre, Spain	100	-1.68	43.08	(123)
Basque	Basque Country northern Spain	106	-2.61	43.07	(123)
Basque	Franco-Cantabrian Region	35	-0.26	42.40	(123)
Basque	Basque Country northern Spain	55	-2.61	43.07	(123)
Alps	Italy	3	9.92	45.97	(124)
Italian	Sant Antioco, Sardinia, Italy	42	9.02	40.11	Falchi et al. GenBank DQ081522-DQ081563
French	South Corsica, France	53	9.15	41.71	Giovannoni et al. GenBank DQ081367-DQ081419
Scotch	Scotland, UK	1341	-4.17	56.74	(125)
Norse	Norway	323	7.61	61.88	(125)
Greek	northern Greece	319	21.42	40.03	(126)
Cyprus	Cyprus	91	33.18	34.95	(126)
Magyars/Hungarians	Carpathian basin	35	19.99	46.50	Kalmar et al. GenBank AF487581-AF487615
Veneto speakers Barco	Veneto, Italy	30	11.94	45.61	(127)
Veneto speakers Barco	Veneto, Italy	38	11.94	45.61	(127)
French	France	142	1.44	43.63	(128)
Italian	Nuoro, Sardinia, Italy	51	9.33	40.32	Varesi et al. GenBank DQ067827-DQ067877
Spanish	Baleares, Spain	67	2.93	39.63	Varesi et al. GenBank DQ081300-DQ081366
Italian	Gallura, Sardinia, Italy	50	9.30	40.99	Varesi et al. GenBank DQ081420-DQ081469
Italian	San Pietro, Sardinia, Italy	44	8.80	40.93	Varesi et al. GenBank

					DQ081564-DQ081607
Italian	Tuscany, Italy	61	11.21	43.64	Varesi et al. GenBank DQ081608-DQ081668
Andalusian	Spain	66	-4.37	39.69	Via et al. GenBank DQ081234-DQ081299
**Japan and Korea**		**2188**			
Japanese	Japan	50	136.92	35.23	(129)
Japanese	Japan	162	138.40	36.40	(130)
Japanese	Japan	211	139.30	36.90	(131)
Japanese	Japan	137	136.76	35.43	(132)
Japanese	Japan	150	138.16	36.74	(133)
Japanese	Japan	100	131.00	33.00	(134)
Japanese	Japan	124	137.40	36.08	(135)
Japanese	Japan	62	138.38	35.04	(136)
Ainu	Japan	50	141.38	43.21	(136)
Pyukyuan	Japan	50	127.73	26.47	(136)
Japanese	Japan	82	138.00	36.00	(137)
Japanese	Japan	104	131.00	33.00	(137)
Japanese	Japan	45	127.72	26.43	(137)
Koryak	Koryak	110	166.73	62.26	(137)
Nivkhi	Nivkhi	57	142.71	53.47	(137)
Korean	South Korea	694	127.76	35.90	(138)
**Total**		**48725**			

References

1. Gayden T, *et al.* (2013) The Himalayas: Barrier and conduit for gene flow. *Am J Phys Anthropol* 151(2):169-182.
2. Ji F, *et al.* (2012) Mitochondrial DNA variant associated with Leber hereditary optic neuropathy and high-altitude Tibetans. *Proc Natl Acad Sci U S A* 109(19):7391-7396.
3. Qi X, *et al.* (2013) Genetic evidence of paleolithic colonization and neolithic expansion of modern humans on the tibetan plateau. *Molecular Biology & Evolution* 30(8):1761-1778.
4. Qin Z, *et al.* (2010) A mitochondrial revelation of early human migrations to the Tibetan Plateau before and after the last glacial maximum. *Am J Phys Anthropol* 143(4):555-569.
5. Zhao M, *et al.* (2009) Mitochondrial genome evidence reveals successful Late Paleolithic settlement on the Tibetan Plateau. *Proc Natl Acad Sci* 106(50):21230-21235.
6. Liu X & Li S (2003) Polymorphism of mitochondrial DNA D-loop region in Chinese Baoan ethnic group. *J Fourth Mil Med Univ* 20:004.
7. Liu X & Li S (2004) Mitochondrial DNA Polymorphism in control region from Chinese Yugu population. *J Xi'an Jiaotong Univ* 16(174-177).
8. Liu X, Chen T, & Li S (2004) Sequence polymorphism of human mitochondrial DNA control region in Chinese Dongxiang unrelated individuals. *J Med Coll PLA* 19(5).
9. Liu X & Li S (2004) Study on polymorphisms of mitochondrial DNA D-loop region in the Sala population in China. *J Xi'an Jiaotong Univ* 25(213-216).
10. Oota H, *et al.* (2002) Extreme mtDNA homogeneity in continental Asian populations. *Am J Phys Anthropol* 118(2):146-153.
11. Wang W, Wise C, Baric T, Black ML, & Bittles AH (2003) The origins and genetic structure of three co-resident Chinese Muslim populations: the Salar, Bo'an and Dongxiang. *Hum Genet* 113(3):244-252.
12. Wen B, *et al.* (2004) Analyses of genetic structure of Tibeto-Burman populations reveals sex-biased admixture in southern Tibeto-Burmans. *Am J Hum Genet* 74(5):856-865.
13. Wen B, *et al.* (2004) Genetic evidence supports demic diffusion of Han culture. *Nature* 431(7006):302-305.
14. Yao Y-G, Kong Q-P, Wang C-Y, Zhu C-L, & Zhang Y-P (2004) Different matrilineal contributions to genetic structure of ethnic groups in the Silk Road region in China. *Mol Biol Evol* 21(12):2265-2280.
15. Yao Y-G, Kong Q-P, Bandelt H-J, Kivisild T, & Zhang Y-P (2002) Phylogeographic differentiation of mitochondrial DNA in Han Chinese. *Am J Hum Genet* 70(3):635-651.
16. Cheng B, *et al.* (2008) Genetic imprint of the Mongol: signal from phylogeographic analysis of mitochondrial DNA. *J Hum Genet* 53(10):905-913.

17. Derenko M, *et al.* (2012) Complete mitochondrial DNA analysis of eastern Eurasian haplogroups rarely found in populations of northern Asia and eastern Europe. *PLoS One* 7(2):e32179.
18. Kong Q-P, *et al.* (2003) Mitochondrial DNA sequence polymorphisms of five ethnic populations from northern China. *Hum Genet* 113(5):391-405.
19. Tajima A, *et al.* (2004) Genetic background of people in the Dominican Republic with or without obese type 2 diabetes revealed by mitochondrial DNA polymorphism. *J Hum Genet* 49(9).
20. Yao Y-G, Kong Q-P, Man X-Y, Bandelt H-J, & Zhang Y-P (2003) Reconstructing the evolutionary history of China: a caveat about inferences drawn from ancient DNA. *Mol Biol Evol* 20(2):214-219.
21. Zhang YJ, Xu QS, Zheng ZJ, Lin HY, & Lee JB (2005) Haplotype diversity in mitochondrial DNA hypervariable region I, II and III in northeast China Han. *Forensic Sci Int* 149(2):267-269.
22. Chen F, *et al.* (2008) Analysis of mitochondrial DNA polymorphisms in Guangdong Han Chinese. *Forensic Sci Int Genet* 2(2):150-153.
23. 陈雪 (2011) <广西壮族与海南黎族人群线粒体 DNA 群体遗传关系的研究.pdf>.
24. Hill C, *et al.* (2007) A mitochondrial stratigraphy for island southeast Asia. *Am J Hum Genet* 80(1):29-43.
25. Kivisild T, *et al.* (2002) The emerging limbs and twigs of the East Asian mtDNA tree. *Mol Biol Evol* 19(10):1737-1751.
26. Li H, *et al.* (2007) Mitochondrial DNA diversity and population differentiation in southern East Asia. *Am J Phys Anthropol* 134(4):481-488.
27. Nishimaki Y, *et al.* (1999) Sequence polymorphism in the mtDNA HV1 region in Japanese and Chinese. *Leg Med* 1(4):238-249.
28. Gan R-J, *et al.* (2008) Pinghua population as an exception of Han Chinese's coherent genetic structure. *J Hum Genet* 53(4):303-313.
29. Tabbada KA, *et al.* (2010) Philippine mitochondrial DNA diversity: a populated viaduct between Taiwan and Indonesia? *Mol Biol Evol* 27(1):21-31.
30. Tajima A, *et al.* (2003) Mitochondrial DNA polymorphisms in nine aboriginal groups of Taiwan: implications for the population history of aboriginal Taiwanese. *Hum Genet* 113(1):24-33.
31. Tsai L, *et al.* (2001) Sequence polymorphism of mitochondrial D-loop DNA in the Taiwanese Han population. *Forensic Sci Int* 119(2):239-247.
32. Wang XQ, Wang CC, Deng QY, & Li H (2013) [Genetic analysis of Y chromosome and mitochondrial DNA poly-morphism of Mulam ethnic group in Guangxi, China]. *Hereditas* 35(2):168-174.
33. Wang WZ, *et al.* (2010) Tracing the origins of Hakka and Chaoshanese by mitochondrial DNA analysis. *Am J Phys Anthropol* 141(1):124-130.
34. Wen B, *et al.* (2005) Genetic structure of Hmong-Mien speaking populations in East Asia as revealed by mtDNA lineages. *Mol Biol Evol* 22(3):725-734.
35. Li Y-C, Huang W, Tian J-Y, Chen X-Q, & Kong Q-P (2016) Exploring the maternal history of the Tai people. *J Hum Genet* 61(8):721-729.
36. Qian YP, *et al.* (2001) Mitochondrial DNA polymorphisms in Yunnan nationalities in China. *J Hum Genet* 46(4):211-220.

37. Wang C-C, *et al.* (2014) Genetic structure of Qiangic populations residing in the western Sichuan corridor. *PLoS One* 9(8):e103772.
38. Wang D, *et al.* (2012) Mitochondrial DNA copy number, but not haplogroup, confers a genetic susceptibility to leprosy in Han Chinese from Southwest China. *PLoS One* 7(6):e38848.
39. Yao Y-G, *et al.* (2002) Genetic relationship of Chinese ethnic populations revealed by mtDNA sequence diversity. *Am J Phys Anthropol* 118(1):63-76.
40. Yao Y-G & Zhang Y-P (2002) Phylogeographic analysis of mtDNA variation in four ethnic populations from Yunnan Province: new data and a reappraisal. *J Hum Genet* 47(6):311-318.
41. Bermisheva M, Tambets K, Villems R, & Khusnutdinova E (2001) [Diversity of mitochondrial DNA haplotypes in ethnic populations of the Volga-Ural region of Russia]. *Mol Biol (Mosk)* 36(6):990-1001.
42. Derbeneva OA, Starikovskaya EB, Volodko NV, Wallace DC, & Sukernik RI (2002) Mitochondrial DNA Variation in the Kets and Nganasans and Its Implications for the Initial Peopling of Northern Eurasia. *Rus J Genet* 38(11):1316-1321.
43. Derbeneva OA, *et al.* (2002) Analysis of Mitochondrial DNA Diversity in the Aleuts of the Commander Islands and Its Implications for the Genetic History of Beringia. *Am J Hum Genet* 71(2):415-421.
44. Derenko M & Shields G (1997) Diversity of mitochondrial DNA nucleotide sequences in three groups of aboriginal inhabitants of Northern Asia]. *Mol Biol (Mosk)* 31(5):784.
45. Derenko MV, *et al.* (2000) Mitochondrial DNA variation in two South Siberian Aboriginal populations: implications for the genetic history of North Asia. *Hum Biol*:945-973.
46. Derenko M, *et al.* (2003) Diversity of mitochondrial DNA lineages in South Siberia. *Ann Hum Genet* 67(5):391-411.
47. Fedorova S, Bermisheva M, Villems R, Maksimova N, & Khusnutdinova E (2003) Analysis of mitochondrial DNA haplotypes in yakut population]. *Mol Biol (Mosk)* 37(4):643.
48. Keyser-Tracqui C, Crubézy E, & Ludes B (2003) Nuclear and Mitochondrial DNA Analysis of a 2,000-Year-Old Necropolis in the Egyin Gol Valley of Mongolia. *Am J Hum Genet* 73(2):247-260.
49. Keyser‐Tracqui C, Crubezy E, Pamzsav H, Varga T, & Ludes B (2006) Population origins in Mongolia: genetic structure analysis of ancient and modern DNA. *Am J Phys Anthropol* 131(2):272-281.
50. Kolman CJ, Sambuughin N, & Bermingham E (1996) Mitochondrial DNA analysis of Mongolian populations and implications for the origin of New World founders. *Genetics* 142(4):1321-1334.
51. Malyarchuk B & Derenko M (2001) Mitochondrial DNA variability in Russians and Ukrainians: Implication to the origin of the Eastern Slavs. *Ann Hum Genet*

65(1):63-78.

52. Pakendorf B, *et al.* (2003) Mitochondrial DNA evidence for admixed origins of central Siberian populations. *Am J Phys Anthropol* 120(3):211-224.

53. Pimenoff VN, *et al.* (2008) Northwest Siberian Khanty and Mansi in the junction of West and East Eurasian gene pools as revealed by uniparental markers. *Eur J Hum Genet* 16(10):1254-1264.

54. Starikovskaya EB, *et al.* (2005) Mitochondrial DNA diversity in indigenous populations of the southern extent of Siberia, and the origins of Native American haplogroups. *Ann Hum Genet* 69(1):67-89.

55. Sukernik RI, *et al.* (2012) Mitochondrial genome diversity in the tubalar, even, and ulchi: Contribution to prehistory of native siberians and their affinities to native americans. *Am J Phys Anthropol* 148(1):123-138.

56. Volodko NV, *et al.* (2008) Mitochondrial genome diversity in arctic Siberians, with particular reference to the evolutionary history of Beringia and Pleistocenic peopling of the Americas. *Am J Hum Genet* 82(5):1084-1100.

57. Li Y-C, *et al.* (2015) Ancient inland human dispersals from Myanmar into interior East Asia since the Late Pleistocene. *Sci Rep* 5.

58. Oota H, Settheetham-Ishida W, Tiwawech D, Ishida T, & Stoneking M (2001) Human mtDNA and Y-chromosome variation is correlated with matrilocal versus patrilocal residence. *Nat Genet* 29(1):20-21.

59. Irwin JA, *et al.* (2008) Mitochondrial control region sequences from a Vietnamese population sample. *Int J Legal Med* 122(3):257-259.

60. Peng MS, *et al.* (2010) Tracing the Austronesian footprint in Mainland Southeast Asia: a perspective from mitochondrial DNA. *Mol Biol Evol* 27(10):2417-2430.

61. Zimmermann B, *et al.* (2009) Forensic and phylogeographic characterization of mtDNA lineages from northern Thailand (Chiang Mai). *Int J Legal Med* 123(6):495-501.

62. Black M, Dufall K, Wise C, Sullivan S, & Bittles A (2006) Genetic ancestries in northwest Cambodia. *Ann Hum Biol* 33(5-6):620-627.

63. Fucharoen G, Fucharoen S, & Horai S (2001) Mitochondrial DNA polymorphisms in Thailand. *J Hum Genet* 46(3):115-125.

64. Lertrit P, *et al.* (2008) Genetic history of Southeast Asian populations as revealed by ancient and modern human mitochondrial DNA analysis. *Am J Phys Anthropol* 137(4):425-440.

65. Hill C, *et al.* (2006) Phylogeography and ethnogenesis of aboriginal Southeast Asians. *Mol Biol Evol* 23(12):2480-2491.

66. Maruyama S, Nohira-Koike C, Minaguchi K, & Nambiar P (2010) MtDNA control region sequence polymorphisms and phylogenetic analysis of Malay population living in or around Kuala Lumpur in Malaysia. *Int J Legal Med* 124(2):165-170.

67. Friedlaender JS, *et al.* (2007) Melanesian mtDNA complexity. *PLoS One* 2(2):e248.

68. Wong HY, *et al.* (2007) Sequence polymorphism of the mitochondrial DNA hypervariable regions I and II in 205 Singapore Malays. *Leg Med* 9(1):33-37.

69. Haslindawaty ARN, Panneerchelvam S, Edinur HA, Norazmi MN, & Zafarina Z (2010) Sequence polymorphisms of mtDNA HV1, HV2, and HV3 regions in the Malay population of Peninsular Malaysia. *Int J Legal Med* 124(5):415-426.
70. Metspalu M, *et al.* (2004) Most of the extant mtDNA boundaries in south and southwest Asia were likely shaped during the initial settlement of Eurasia by anatomically modern humans. *BMC Genet* 5(1):26.
71. Bodner M, *et al.* (2011) Southeast Asian diversity: first insights into the complex mtDNA structure of Laos. *BMC Evol Biol* 11(1):49.
72. Zhang X, *et al.* (2013) Analysis of mitochondrial genome diversity identifies new and ancient maternal lineages in Cambodian aborigines. *Nat Commun* 4.
73. Summerer M, *et al.* (2014) Large-scale mitochondrial DNA analysis in Southeast Asia reveals evolutionary effects of cultural isolation in the multi-ethnic population of Myanmar. *BMC Evol Biol* 14(1):17.
74. Scholes C, *et al.* (2011) Genetic diversity and evidence for population admixture in Batak Negritos from Palawan. *Am J Phys Anthropol* 146(1):62-72.
75. Cordaux R, *et al.* (2003) Mitochondrial DNA analysis reveals diverse histories of tribal populations from India. *Eur J Hum Genet* 11(3):253-264.
76. Roychoudhury S, *et al.* (2001) Genomic structures and population histories of linguistically distinct tribal groups of India. *Hum Genet* 109(3):339-350.
77. Reddy BM, *et al.* (2007) Austro-Asiatic tribes of Northeast India provide hitherto missing genetic link between South and Southeast Asia. *PLoS One* 2(11):e1141.
78. Quintana-Murci L, *et al.* (2004) Where west meets east: the complex mtDNA landscape of the southwest and Central Asian corridor. *Am J Hum Genet* 74(5):827-845.
79. Sharma S, Saha A, Rai E, Bhat A, & Bamezai R (2005) Human mtDNA hypervariable regions, HVR I and II, hint at deep common maternal founder and subsequent maternal gene flow in Indian population groups. *J Hum Genet* 50(10):497-506.
80. Barnabas S, Shouche Y, & Suresh C (2006) High‐Resolution mtDNA Studies of the Indian Population: Implications for Palaeolithic Settlement of the Indian Subcontinent. *Ann Hum Genet* 70(1):42-58.
81. Thangaraj K, *et al.* (2005) Different population histories of the Mundari-and Mon-Khmer-speaking Austro-Asiatic tribes inferred from the mtDNA 9-bp deletion/insertion polymorphism in Indian populations. *Hum Genet* 116(6):507-517.
82. Mountain JL, *et al.* (1995) Demographic history of India and mtDNA-sequence diversity. *Am J Hum Genet* 56:979-992.
83. Rajkumar R & Kashyap V (2003) Haplotype diversity in mitochondrial DNA hypervariable regions I and II in three communities of Southern India. *Forensic Sci Int* 136(1):79-82.
84. Rajkumar R & Kashyap VK (2003) Mitochondrial DNA hypervariable region I and II sequence polymorphism in the Dravidian linguistic group of India. *J Forensic Sci* 48(1):227-237.
85. Thangaraj K, Ramana GV, & Singh L (1999) Y‐chromosome and mitochondrial DNA polymorphisms in Indian populations. *Electrophoresis* 20(8):1743-1747.

86. Kivisild T, *et al.* (1999) Deep common ancestry of Indian and western-Eurasian mitochondrial DNA lineages. *Curr Biol* 9(22):1331-1334.
87. Quintana-Murci L, *et al.* (1999) Genetic evidence of an early exit of Homo sapiens sapiens from Africa through eastern Africa. *Nat Genet* 23(4):437-441.
88. Thanseem I, *et al.* (2006) Genetic affinities among the lower castes and tribal groups of India: inference from Y chromosome and mitochondrial DNA. *BMC Genet* 7(1):42.
89. Kumar V, *et al.* (2006) Global patterns in human mitochondrial DNA and Y-chromosome variation caused by spatial instability of the local cultural processes. *PLoS Genet* 2(4):e53.
90. Kivisild T, *et al.* (2003) The genetic heritage of the earliest settlers persists both in Indian tribal and caste populations. *Am J Hum Genet* 72(2):313-332.
91. Watkins W, *et al.* (1999) Multiple origins of the mtDNA 9-bp deletion in populations of South India. *Am J Phys Anthropol* 109(2):147-158.
92. Mittal B, *et al.* (2008) Mitochondrial DNA variation and substructure among the tribal populations of Andhra Pradesh, India. *Am J Hum Biol* 20(6):683-692.
93. Gaikwad S & Kashyap V (2005) Molecular insight into the genesis of ranked caste populations of western India based upon polymorphisms across non-recombinant and recombinant regions in genome. *Genome Biol* 6(8):P10.
94. Roy S, Thakur C, & Majumder PP (2003) Mitochondrial DNA variation in ranked caste groups of Maharashtra (India) and its implication on genetic relationships and origins. *Ann Hum Biol* 30(4):443-454.
95. Baig M, Khan A, & Kulkarni K (2004) Mitochondrial DNA diversity in tribal and caste groups of Maharashtra (India) and its implication on their genetic origins. *Ann Hum Genet* 68(5):453-460.
96. Kaur I, *et al.* (2002) Genomic diversities and affinities among four endogamous groups of Punjab (India) based on autosomal and mitochondrial DNA polymorphisms. *Hum Biol*:819-836.
97. Banerjee J, Trivedi R, & Kashyap V (2005) Mitochondrial DNA control region sequence polymorphism in four indigenous tribes of Chotanagpur plateau, India. *Forensic Sci Int* 149(2):271-274.
98. Sahoo S & Kashyap V (2006) Phylogeography of mitochondrial DNA and Y‐Chromosome haplogroups reveal asymmetric gene flow in populations of Eastern India. *Am J Phys Anthropol* 131(1):84-97.
99. Thangaraj K, *et al.* (2003) Genetic affinities of the Andaman Islanders, a vanishing human population. *Curr Biol* 13(2):86-93.
100. Prasad BV, *et al.* (2001) Mitochondrial DNA variation in Nicobarese Islanders. *Human Biology* 73(5):715-725.
101. Wirth T, *et al.* (2004) Distinguishing human ethnic groups by means of sequences from Helicobacter pylori: Lessons from Ladakh. *Proc Natl Acad Sci* 101(14):4746-4751.
102. Eaaswarkhanth M, *et al.* (2010) Traces of sub-Saharan and Middle Eastern lineages in Indian Muslim populations. *Eur J Hum Genet* 18(3):354-363.

103. Fornarino S, *et al.* (2009) Mitochondrial and Y-chromosome diversity of the Tharus (Nepal): a reservoir of genetic variation. *BMC Evol Biol* 9(1):154.
104. Wang H-W, *et al.* (2012) Revisiting the role of the Himalayas in peopling Nepal: insights from mitochondrial genomes. *J Hum Genet* 57(4):228-234.
105. Gazi NN, *et al.* (2013) Genetic Structure of Tibeto-Burman Populations of Bangladesh: Evaluating the Gene Flow along the Sides of Bay-of-Bengal. *PLoS One* 8(10):e75064.
106. Li YC, *et al.* (2017) Cultural diffusion of Indo-Aryan languages into Bangladesh: A perspective from mitochondrial DNA. *Mitochondrion* 38.
107. Comas D, *et al.* (2004) Admixture, migrations, and dispersals in Central Asia: evidence from maternal DNA lineages. *Eur J Hum Genet* 12(6):495-504.
108. Comas D, *et al.* (1998) Trading genes along the silk road: mtDNA sequences and the origin of central Asian populations. *Am J Hum Genet* 63(6):1824-1838.
109. Irwin JA, *et al.* (2010) The mtDNA composition of Uzbekistan: a microcosm of Central Asian patterns. *Int J Legal Med* 124(3):195-204.
110. Chaix R, *et al.* (2007) From social to genetic structures in central Asia. *Curr Biol* 17(1):43-48.
111. Lalueza-Fox C, *et al.* (2004) Unravelling migrations in the steppe: mitochondrial DNA sequences from ancient Central Asians. *Proc R Soc Lond B Biol Sci* 271(1542):941-948.
112. Dulik Matthew C, *et al.* (2012) Mitochondrial DNA and Y Chromosome Variation Provides Evidence for a Recent Common Ancestry between Native Americans and Indigenous Altaians. *Am J Hum Genet* 90(2):229-246.
113. Derenko M, *et al.* (2007) Phylogeographic Analysis of Mitochondrial DNA in Northern Asian Populations. *Am J Hum Genet* 81(5):1025-1041.
114. Malyarchuk B, Derenko M, Denisova G, & Kravtsova O (2010) Mitogenomic diversity in Tatars from the Volga-Ural region of Russia. *Mol Biol Evol* 27(10):2220-2226.
115. Malyarchuk B, *et al.* (2002) Mitochondrial DNA variability in Poles and Russians. *Ann Hum Genet* 66(4):261-283.
116. Achilli A, *et al.* (2007) Mitochondrial DNA variation of modern Tuscans supports the near eastern origin of Etruscans. *Am J Hum Genet* 80(4):759-768.
117. Achilli A, *et al.* (2011) Mitochondrial DNA backgrounds might modulate diabetes complications rather than T2DM as a whole. *PLoS One* 6(6):e21029.
118. Álvarez-Cubero MJ, *et al.* (2012) Mitochondrial Haplogroups and Polymorphisms Reveal No Association with Sporadic Prostate Cancer in a Southern European Population. *PLoS One* 7(7):e41201.
119. Bertolin C, *et al.* (2011) Analysis of complete mitochondrial genomes of patients with schizophrenia and bipolar disorder. *J Hum Genet* 56(12):869-872.
120. Bogacsi-Szabo E, *et al.* (2005) Mitochondrial DNA of ancient Cumanians: culturally Asian steppe nomadic immigrants with substantially more western Eurasian mitochondrial DNA lineages. *Hum Biol*:639-662.
121. Cardoso S, *et al.* (2010) Variability of the entire mitochondrial DNA control region in a human isolate from the Pas Valley (northern Spain). *J Forensic Sci* 55(5):1196-1201.

122. Cardoso S, *et al.* (2011) The maternal legacy of Basques in northern navarre: New insights into the mitochondrial DNA diversity of the Franco - Cantabrian area. *Am J Phys Anthropol* 145(3):480-488.
123. Cardoso S, *et al.* (2013) The Expanded mtDNA Phylogeny of the Franco-Cantabrian Region Upholds the Pre-Neolithic Genetic Substrate of Basques. *PLoS One* 8(7):e67835.
124. Di Benedetto G, *et al.* (2000) Mitochondrial DNA sequences in prehistoric human remains from the Alps. *Eur J Hum Genet* 8(9):669-677.
125. Helgason A, *et al.* (2001) mtDNA and the islands of the North Atlantic: estimating the proportions of Norse and Gaelic ancestry. *Am J Hum Genet* 68(3):723-737.
126. Irwin J, *et al.* (2008) Mitochondrial control region sequences from northern Greece and Greek Cypriots. *Int J Legal Med* 122(1):87-89.
127. Mogentale-Profizi N, *et al.* (2001) Mitochondrial DNA sequence diversity in two groups of Italian Veneto speakers from Veneto. *Ann Hum Genet* 65(2):153-166.
128. Pierron D, *et al.* (2008) New evidence of a mitochondrial genetic background paradox: impact of the J haplogroup on the A3243G mutation. *BMC Med Genet* 9(1):41.
129. Koyama H, *et al.* (2002) Mitochondrial sequence haplotype in the Japanese population. *Forensic Sci Int* 125(1):93-96.
130. Imaizumi K, Parsons TJ, Yoshino M, & Holland M (2002) A new database of mitochondrial DNA hypervariable regions I and II sequences from 162 Japanese individuals. *Int J Legal Med* 116(2):68-73.
131. Maruyama S, Minaguchi K, & Saitou N (2003) Sequence polymorphisms of the mitochondrial DNA control region and phylogenetic analysis of mtDNA lineages in the Japanese population. *Int J Legal Med* 117(4):218-225.
132. Nagai A, Nakamura I, Shiraki F, Bunai Y, & Ohya I (2003) Sequence polymorphism of mitochondrial DNA in Japanese individuals from Gifu Prefecture. *Leg Med* 5:S210-S213.
133. Nishimaki Y, *et al.* (1999) Sequence polymorphism in the mtDNA HV1 region in Japanese and Chinese. *Legal Med* 1(4):238-249.
134. Seo Y, Stradmann-Bellinghausen B, Rittner C, Takahama K, & Schneider PM (1998) Sequence polymorphism of mitochondrial DNA control region in Japanese. *Forensic Sci Int* 97(2):155-164.
135. Mabuchi T, Susukida R, Kido A, & Oya M (2007) Typing the 1.1 kb control region of human mitochondrial DNA in Japanese individuals. *J Forensic Sci* 52(2):355-363.
136. Horai S, *et al.* (1996) mtDNA polymorphism in East Asian Populations, with special reference to the peopling of Japan. *Am J Hum Genet* 59(3):579.
137. Tajima A, *et al.* (2004) Genetic origins of the Ainu inferred from combined DNA analyses of maternal and paternal lineages. *J Hum Genet* 49(4):187-193.
138. Lee HY, *et al.* (2006) East Asian mtDNA haplogroup determination in Koreans: Haplogroup-level coding region SNP analysis and subhaplogroup-level control region sequence analysis. *Electrophoresis* 27(22):4408-4418.

Table S4. Radiocarbon dates of archaeological sites with millet (n=120) in the TP and north China.

Site	Elevation (m.asl)	^{14}C age (BP)	Calibrated age 2σ (BP)	Reference
Yuezhuang	45	6900 ±35	7745±78	1
Beiqian	10	4690 ±35	5448±129	2
Beiqian	10	4470 ±40	5132±160	2
Beiqian	10	4695 ±35	5448±129	2
Beiqian	10	4660 ±35	5440±128	2
Beiqian	10	4570 ±40	5248±196	2
Beiqian	10	4640 ±30	5386±78	2
Beiqian	10	4555 ±35	5243±190	2
Xinglonggou	354	—	7640±30	3
Xianglushan	668	3340 ±30	3579±100	4
Xianglushan	668	3360 ±30	3588±103	4
Baoguluxi	630	3450 ±30	3733±96	4
Xianglushan	668	2880 ±30	3014±126	4
Guangdegongnanshan	1118	2410 ±30	2518±168	4
Huamuzhazi	1529	2690 ±30	2800±48	4
Nanjiaokou	477	5220±35	6042±132	5
Nanjiaokou	477	5550±40	6347±59	5
Nanjiaokou	477	5195±40	6033±140	5
Nanjiaokou	477	4480±45	5136±164	5
Nanjiaokou	477	4690±40	5447±130	5
Nanjiaokou	477	4655±40	5439±131	5
Nanjiaokou	477	4685±35	5447±129	5
Nanjiaokou	477	4535±35	5181±130	5
Nanjiaokou	477	4645±40	5435±133	5

Nanjiaokou	477	4505±35	5172±128	5
Nanjiaokou	477	5550±35	6347±55	5
Nanjiaokou	477	4530±35	5180±130	5
Huizui	209	3590±40	3896±168	5
Huizui	209	3455±55	3715±135	6
Huizui	209	3415±35	3698±124	6
Huizui	209	3425±35	3703±122	6
Huizui	209	3600±70	3896±194	6
Huizui	209	4380±30	4950±88	6
Huizui	209	3680±30	4025±112	7
Huizui	209	3595±30	3906±70	7
Huizui	209	3180±35	3407±63	7
Nanshi	128	3740±70	4136±244	7
Sigou	113	4260±50	4795±167	8
Weizhuang	192	4330±70	4965±310	8
Fengyangzhai	248	2940±40	3088±120	8
Tianposhuiku	253	3060±50	3231±150	8
Feiyao	183	3510±40	3769±120	8
Gaoya	126	2665±35	2795±49	7
Gaoya	126	4420±30	5069±199	7
Jinzhongshi	126	3655±30	3988±95	7
Jingyanggang	126	2575±30	2638±120	7
Jingyanggang	126	3175±25	3405±43	7

Matun	223	3590±60	3898±183	8
Matun	223	3550±25	3816±92	7
Matun	223	3465±35	3736±95	7
Matun	223	3390±35	3685±124	7
Shaochai	130	3125±35	3340±102	7
Tunzhai	130	3630±30	3965±111	7
Tunzhai	130	3060±40	3266±99	7
zhaiwan	127	3605±35	3948±116	7
Luokoudong	127	3190±45	3413±143	7
Baligang	126	2935±35	3086±119	9
Yingyang	454	4950±85	5696±209	10
Mawangcun	414	2850±50	2995±147	11
Mawangcun	414	2900±50	3027±146	11
Mawangcun	414	2860±33	2972±99	12
Xishanping	1330	4490±35	5140±158	13
Guanzizui	2126	3630±30	3965±111	14
Sumiaoyuantou	1740	3600±25	3908±64	14
Laohuzui	1358	3870±30	4287±126	14
Xihetan	1608	3795±50	4199±206	15
Mozuizi	1722	3750±35	4109±124	15
Buziping	2134	4300±25	4893±63	16
Buziping	2134	3610±20	3918±58	16
Linjia	2320	4110±95	4632±217	17

Jinchankou	2309	3555±25	3840±115	18
Jinchankou	2309	3705±25	4060±84	18
Jinchankou	2309	3705±25	4060±84	18
Jinchankou	2309	3585±20	3899±63	18
Benbakou	2415	4135±25	4695±126	14
Pingandongcun	2100	3980±25	4466±53	14
Xinjia	1815	3690±30	4035±109	14
Wayaotai	1697	3410±30	3694±118	14
Andaqiha	2015	4340±40	4937±98	14
Shangduoba	2065	4035±30	4600±177	14
Hurere	1930	4530±60	5208±234	19
Luowalinchang	2326	4470±25	5141±164	19
Gayixiangjing	2255	4410±40	5068±206	19
Hongtjiaozi	2254	4395±30	4956±90	19
Gayixiangjing	2255	4370±25	4947±87	19
Andaqiha	2059	4340±40	4937±98	19
Zhangga	2210	4340±40	4937±98	19
Heibiya	2355	4245±30	4759±103	19
Hongyazhangjia	1941	4185±35	4712±127	19
Benbakou	2408	4185±25	4731±105	19
Benbakou	2408	4135±25	4695±126	19
Luowalinchang	2326	4110±30	4669±145	19
Shangduoba	2105	4035±30	4600±178	19

Dongcun	2280	3980±25	4467±54	19
Yaluhu	2002	3940±25	4402±109	19
Mijiawan	2233	3900±30	4332±86	19
Liuwanshagou	1901	3840±25	4279±127	19
Yangjiazhaipo	2468	3715±25	4064±83	19
Xinjia	1835	3690±30	3994±149	19
Xiasunjiazhai	2334	3680±25	4008±82	19
Nanshansi	1953	3680±25	4008±82	19
Ajiacun	2527	3640±30	3975±107	19
Zhongtan	2132	3640±30	3975±107	19
Zhaojiazhuang	2093	3595±25	3906±67	19
Qingshiya	2146	3755±25	4111±118	19
Wayaotai	1760	3410±30	3694±121	19
Karuo	3233	4115±25	4669±142	20
Karuo	3233	3995±25	4469±51	20
Karuo	3233	3980±40	4431±135	20
Karuo	3233	3910±25	4337±83	20
Guojiacun	54	3990±90	4486±327	21
Yuanqushangcheng	262	3550±90	3850±236	17
Nanshantou	785	4685±30	5446±127	22
Nanshantou	785	4340±30	4935±90	22
Nanshantou	785	4265±25	4844±18	22
Gaomuxudi	1850	4150±30	4701±124	23

Xihetan	1587	3675±35	4020±120	23
Xitai	2029	3770±25	4039±105	23
Duojialiang	1820	3730±20	40699±80	23
Xichengyi	1462	3745±25	4110±122	23

Reference：

1. Crawford CW, Chen XS, Luan FS, Wang JH. A preliminary analysis on plant remains of the Yuezhuang site in Changqing district, Jinan city, Shandong province. Jianghan Archaeology **127**, 107-116 (2013). (InChinese)

2. Jin GY, Wagner M, Tarasov PE, Wang F, Liu YC. Archaeobotanical records of Middle and Late Neolithic agriculture from Shandong Province, East China, and a major change in regional subsistence during the Dawenkou Culture. The Holocene **26**, 1605-1615 (2016).

3. Zhao ZJ. New Archaeobotanic Data for the Study of the Origins of Agriculture in China. Current Anthropology **52**(4), S295-S306 (2011).

4. Jia X, *et al.* The transition of human subsistence strategies in relation to climate change during the Bronze Age in the West Liao River Basin, Northeast China. The Holocene **26**, 781-789 (2016).

5. Henan Provincial Institute of Cultural Relics. Nanjiaokou site in Sanmenxia city. Beijing: Science Press, (2009). (In Chinese)

6. Lee GA, Bestel S. Contextual analysis of plant remains at the Erlito-period Huizui site, Henan, China. Bulletin of the Indo-Pacific Prehistory Association **27**, 49-60 (2007).

7. Zhang JN, Xia ZK, Zhang XH. Research on charred plant remains from the Neolithic to the Bronze Age in Luoyang Basin . Chin Sci Bull **59**, 3388–3397 (2014). (in Chinese)

8. Lee GA, Crawford GW, Liu L, Chen XC. Plants and people from the Early Neolithic to Shang periods in North China. Proceedings of the National Academy of Sciences **104**, 1087-1092 (2007).

9. Deng ZH, Qin L, Gao Y, Weisskopf AR, Zhang C, Fuller DQ. From early domesticated rice of the Middle Yangtze Basin to Millet, rice and wheat agriculture:

Archaeobotanical macro-remains from Baligang, Nanyang Basin, Central China (6700–500 BC). PLoS One **10**, e0139885 (2015).

10. Radiocarbon laboratory of Peking University. Radiocarbon dating report (one). Cultural Relics, (1996). (In Chinese)

11. Experts of Xia-Shang-Zhou Chronology Project. Panel of Xia-Shang-Zhou Chronology Project. Beijing: World Book Inc, (2000). (In Chinese)

12. Institute of Archaeology, Chinese Academy of Social Sciences. Radiocarbon dating report, (2002). (In Chinese)

13. Li XQ, Zhou XY, Zhou J, Dodson J, Zhang HB, Shang X. The earliest archaeobiological evidence of the broadening agriculture in China recorded at Xishanping site in Gansu Province. Science in China Series D: Earth Sciences **50**, 1707-1714 (2007).

14. Dong GH, *et al.* A comparative study of radiocarbon dating charcoal and charred seeds from the same flotation samples in the Late Neolithic and Bronze Age sites in the Gansu and Qinghai Provinces, Northwest China. Radiocarbon **56**, 157-163 (2014).

15. Zhou XY, Li XQ, Dodson J, Zhao KL. Rapid agricultural transformation in the prehistoric Hexi corridor, China. Quaternary International **426**, 33-41 (2016).

16. Jia X, *et al.* The development of agriculture and its impact on cultural expansion during the late Neolithic in the Western Loess Plateau, China. The Holocene **23**, 85-92 (2013).

17. Institute of Archaeology Chinese Academy of Social Sciences. Radiocarbon dating report (seven). Cultural Relics, (1987). (In Chinese)

18. Yang Y. The analysis of charred plant seeds at Jinchankou site and Lijiaping site during Qijia culture period in the Hehuang region, China. Master’s Thesis. Lanzhou: Lanzhou University, (2014).

19. Chen FH, *et al.* Agriculture facilitated permanent human occupation of the Tibetan Plateau after 3600 BP. Science **347**. 248-250 (2015).

20. D'Alpoim Guedes, J. Adaptation and invention during the spread of agriculture to southwest China. Doctoral dissertation. Cambridge: Harvard University, (2013).

21. Institute of Archaeology Chinese Academy of Social Sciences. Radiocarbon dating report (six). Cultural Relics, (1990). (In Chinese).

22. Wang X, Shang X, Jiang HE, Zhang CP, Wang WL, Wang CS. A preliminary study of flotation results of two ancient sites in the Baishui River region, Shaanxi province. Archaeology and Cultural Relics **2**, 100-104 (2015).

23. Dong GH, *et al.* Prehistoric trans-continental cultural exchange in the Hexi Corridor, northwest China. The Holocene **28**(4), 621-628 (2018).

Table S5. Radiocarbon dates of archaeological sites with wheat and barley remains (n=131) in the TP and north China.

Site	Elevation (m.asl)	^{14}C age (BP)	Calibrated age 2σ(BP)	Reference
Feiyaonan	213	2840±25	2950±83	1
Feiyaonan	213	3000±30	3200±125	1
Zhaojiashuimo	1468	2600±40	2647±139	2
Yingwoshu	1179	3150±30	3356±91	2
Yingwoshu	1179	3245±30	3478±82	2
Ganguya	1468	3025±35	3212±132	2
Guojiashan	1534	3370±35	3591±106	2
Guojiashan	1534	3380±30	3629±64	2
Ganggangwa	1216	3560±50	3842±132	2
Huoshiliang	1208	3635±45	3959±125	2
Mozuizi	1799	2115±50	2126±177	3
Diaoyutai	34	2370±90	2440±281	4
Xindian	1862	2805±35	2812±16	9
Donghuishan	1782	3215±50	3460±104	5
Donghuishan	1782	3195±50	3415±144	5
Donghuishan	1782	3215±40	3460±96	5
Donghuishan	1782	3195±35	3449±93	5
Donghuishan	1782	3235±35	3471±88	5
Donghuishan	1782	3225±35	3467±91	5
Donghuishan	1782	3175±35	3369±95	5
Donghuishan	1782	3235±35	3471±88	5
Donghuishan	1782	3260±35	3484±85	5
Donghuishan	1782	3250±35	3479±83	5
Donghuishan	1782	3265±35	3486±85	5

Donghuishan	1782	3240±35	3474±86	5
Donghuishan	1782	3260±45	3481±96	3
Donghuishan	1782	3405±50	3671±157	3
Donghuishan	1782	3425±40	3702±124	3
Donghuishan	1782	3410±50	3695±135	3
Xichengyi	1476	3391±26	3635±58	6
Xichengyi	1476	3602±22	3910±62	6
Xichengyi	1476	3391±24	3635±57	6
Lijiaping	2508	3370±35	3591±106	7
Lijiaping	2508	3380±35	3609±99	7
Lijiaping	2508	3240±30	3474±85	7
Jinchankou	2419	3565±20	3867±90	7
Jinchankou	2419	3605±25	3911±65	7
Jinchankou	2419	3515±25	3782±78	7
Jinchankou	2419	3545±25	3812±89	7
Jinchankou	2419	3535±25	3806±85	7
Jinchankou	2419	3565±25	3846±116	7
Jinchankou	2419	3595±20	3906±65	8
Jinchankou	2419	3440±30	3730±99	8
Huangniangniangtai	1794	3570±60	3867±208	3
Huoshaogou	1751	2495±45	2560±180	3
Huoshaogou	1751	3430±50	3703±106	3
Shaguoliang	1283	3390±50	3653±171	3
Shaguoliang	1283	3450±60	3718±146	3
Nansha	437	3260±35	3484±85	3
Nansha	437	3275±30	3493±80	3

Nansha	437	3300±30	3522±69	3
Dongfengxinan	2028	3010±40	3206±134	9
Shuangerdongping	2017	2770±25	2865±76	9
Yantai	40	2695±25	2802±45	10
Zhaojiazhuang	10	3905±50	4514±4	10
Jiaochangpu	33	2710±20	2807±43	10
Jiaochangpu	33	2210±50	2208±135	10
Liujiazhuang	18	2841±26	2961±93	10
Daxinzhuang	51	3114±30	3314±75	10
Dongpan	59	2503±29	2611±122	10
Dongpan	59	2511±28	2614±124	10
Zhaogezhuang	15	2746±26	2847±72	10
Wangchenggang	248	3155±30	3359±90	10
Wangchenggang	248	3205±25	3421±46	10
Wangchenggang	248	3210±40	3458±97	10
Wangchenggang	248	3180±25	3407±42	10
Yanshishangcheng	124	2475±20	2589±123	10
zhouyuan	682	3140±20	3047±91	10
Donggao	357	2895±20	3047±91	10
changguogou	3659	3122±29	3340±94	10
changguogou	3659	3070±30	3273±89	10
changguogou	3659	3100±30	3306±73	10
changguogou	3659	3120±30	3316±73	10
Bangga	3562	2260±24	2252±93	10
Bangga	3562	2280±30	2255±95	10
Bangga	3562	2450±30	2531±171	10

Bangga	3562	2820±30	2925±76	10
Karuo	3233	2913±27	3061±95	10
Karuo	3233	2877±24	2974±81	10
Karuo	3233	3317±27	3540±73	10
Fengtai	2642	2615±20	2750±13	10
Xishanping	1451	2450±20	2530±168	10
Xishanping	1451	2460±20	2551±154	10
Xishanping	1451	2510±25	2614±123	10
Xishanping	1451	2500±20	2642±151	10
Guojiashan	1726	3560±25	3843±115	11
Maolinshan	1785	2900±30	3054±101	11
Lijiageleng	1804	3415±25	3699±111	11
Huoshiliang	1198	3495±20	3768±65	11
Shaguoliang	1266	3250±30	3480± 81	11
Dadunwan	1789	3155±20	3395±49	11
Xihuishan	1654	3445±20	3730±92	11
Ganguya	1821	3435±20	3727±94	11
Sanjiaocheng	1440	2500±25	2607±118	11
Huoshitan	1306	2505±20	2609±117	11
Zhaojiashuimo	1451	2630±20	2756±14	11
Guohuitai	2300	2505±20	2609±117	11
Huoshaogou	1774	2770±25	2865±76	11
Lucheng	946	2140±25	2145±145	11
Gudongtan	1267	2520±35	2616±127	11
Xiasunjiazhai	2247	3340±40	3578±108	8
Xiasunjiazhai	2247	3665±25	3997±88	8

Gongshijia	2074	3620±30	3955±112	8
Gongshijia	2074	3165±35	3398±63	8
Jiaoridang	2258	2486±68	2546±190	8
Talitaliha	2802	2840±30	2961±95	8
Talitaliha	2802	2770±30	2866±78	8
Xiariyamakebu	3050	3100±30	3306±73	8
Hongshanzuinanpo	3086	3075±30	3288±78	8
Huidui	2384	3060±35	3266±100	8
Lagalamaerma	3341	3060±30	3285±75	8
Luowalinchang	2326	3075±40	3277±98	8
Luowalinchang	2326	3050±30	3262±95	8
Longshan	2585	3030±25	3250±90	8
Tawendaliha	2861	3110±52	3324±141	8
Aiqingya	3221	3160±30	3361±88	8
Kalashishuwan	2642	3020±25	3210±126	8
Weijiabao	2423	2905±30	3075±122	8
Tuanjie	2836	2930±35	3088±121	8
Erfang	1772	2910±30	3078±121	8
Wenjia	1813	2890±30	3041±115	8
Bayan	2815	2860±20	2977±88	8
Longshan	2585	2790±20	2882±73	8
Yingpandi	2343	2760±25	2854±72	8
Xiawatai	2660	2750±30	2848±71	8
Lalongwa	2811	2685±30	2799±48	8
Gagai	2610	2550±30	2624±126	8
Keer	3190	2550±30	2624±126	8

Lamuzui	2314	2520±40	2606±140	8
Yangou	1878	2460±30	2534±171	8
Shawuang	2697	2325±30	2317±133	8

Reference:

1. Zhang JN, Xia ZK, Zhang XH. Research on charred plant remains from the Neolithic to the Bronze Age in Luoyang Basin . Chin Sci Bull **59**, 3388–3397 (2014). (in Chinese)

2. Zhou XY, Li XQ, Dodson J, Zhao KL. Rapid agricultural transformation in the prehistoric Hexi corridor, China. Quaternary International **426**, 33-41 (2016).

3. Dodson J, Li XQ, Zhou XY, Zhao KL, Sun N, Atahan P. Origin and spread of wheat in China. Quaternary Science Reviews **72**, 108-111 (2013).

4. Institute of Archaeology, Chinese Academy of Social Sciences. Radiocarbon dates report. Beijing: Cultural Relics Press, (1991).

5. Flad R, Li SC, Wu XH, Zhao ZJ. Early wheat in China: Results from new studies at Donghuishan in the Hexi Corridor. The Holocene **20**, 955-965 (2010).

6. Institute of Archaeology, Chinese Academy of Social Sciences. Radiocarbon dates report (forty one). Archaeology, (1991).

7. Yang Y. The analysis of charred plant seeds at Jinchankou site and Lijiaping site during Qijia culture period in the Hehuang region, China. Master's Thesis. Lanzhou: Lanzhou University, (2014).

8. Chen FH, *et al.* Agriculture facilitated permanent human occupation of the Tibetan Plateau after 3600 BP. Science **347**, 248-250 (2015).

9. Dong GH, *et al.* A comparative study of radiocarbon dating charcoal and charred seeds from the same flotation samples in the Late Neolithic and Bronze Age sites in the Gansu and Qinghai Provinces, Northwest China. Radiocarbon **56**, 157-163 (2014).

10. Liu XY, *et al.* The virtues of small grain size: Potential pathways to a distinguishing feature of Asian wheats. Quaternary International **426**, 107-119 (2016).

11. Dong GH, Y *et al.* Prehistoric trans-continental cultural exchange in the Hexi Corridor, northwest China. The Holocene **28**(4), 621-628 (2018).

Table S6. Radiocarbon dates of sites with human bones and the stable isotope signals in the TP and north China.

Site	Elevation (m.asl)	^{14}C dates (BP)	Calibrated age 2σ (BP)	Archaeological age	Stable isotope signals	Reference
Dadiwan	1456	5010±35	5773±119	—	C_4	1
Dadiwan	1456	4950±30	5669±46	—	C_4	1
Xinglonggou I	548	6485±35	7389±70	—	C_4	5
Xinglonggou I	548	6695±40	7567±83	—	C_4	5
Baiyinchanghan	937	5290±55	6068±139	—	C_4	5
Caomaoshan	515	4550±35	5185±135	—	C_4	5
Caomaoshan	515	4570±40	5248±196	—	C_4	5
Caomaoshan	515	4605±40	5268±197	—	C_4	5
Xishan-Chifeng	572	4020±50	4651±242	—	C_4	5
Xishan-Chifeng	572	4045±40	4608±189	—	C_4	5
Xishan-Chifeng	572	4035±40	4601±183	—	C_4	5
Xinglongwa 3	548	3165±35	3361±94	—	C_4	5
Xinglongwa 3	548	3120±40	3335±108	—	C_4	5
Xinglongwa 3	548	3130±40	3342±102	—	C_4	5
huoshiliang	1208	3515±45	3774±129	—	C_4	6
huoshiliang	1208	3590±45	3898±173	—	C_4	6
Liuzhuang	104	—	—	3750-3600 BP	C_4	7
Qijiaping	1940	3050±50	3228±148	—	C_4	8
Qijiaping	1940	3215±40	3460±96	—	C_4	8
liangchengzhen	37	—	—	4600-4000 BP	C_4	10
Dongying	3	—	—	5900-5500 BP	C_4	11
Dongying	3	—	—	4600-4000 BP	C_4	11
Hupo	2380	3785±35	4143±143	—	C_4	13

Xiahaishi	1800	3680±30	4025±112	—	C_4	13
Xiahaishi	1800	3630±30	3965±111	—	C_4	13
Sanheyi	2400	3595±25	3905±65	—	C_4	13
Xin'anzhuang	82	—	—	3600-3046 BP	C_4	14
Beiliu	551	—	—	8000-5500 BP	C_4	15
Jiangzhai	405	—	—	6900-6000 BP	C_4	16
Shijia	478	—	—	6300-6000 BP	C_4	16
Yinxu	80	—	—	3250-3046 BP	C_4	17
Mengjiawa	111	—	—	2770-2200 BP	C_4	18
Zongri	2820	—	—	5200-4100 BP	C_4	19
Mozuizi	1779	—	—	4350-4000 BP	C_4	20
Wuba	2310	—	—	4450-3950 BP	C_4	20
Taosi	582	—	—	4600-4000 BP	C_4	21
Xujianian	1650	2745±130	2858±367	—	C_4	21
Liulihe Tombs	51	—	—	3046-2771 BP	C_4	21
Yanshishangcheng	124	—	—	3500 BP	C_4	21
Nanzhai	321	—	—	3750-3530 BP	C_4	21
Guzhendu	163	—	—	6650-5600 BP	C_4	21
Beizhuang	13	—	—	5400-5100 BP	C_4	21
Qingliangsi Tombs	504	—	—	5500 BP	C_4	23
Xinzhai	123	—	—	3870-3720 BP	C_4	24
Erlitou	120	—	—	3735-3530 BP	C_4	24
Qianzhangda	45	—	—	3100 BP	C_4	24
Wadian	128	—	—	4200-3900 BP	C_4	25
Beiquan	6	—	—	6000-5000 BP	C_4	27
Nancheng tombs	55	—	—	4000-3600 BP	C_4	29

Shengedaliang	986	3180±30	3406±49	—	C_4	30
Yuhuazhai	406	—	—	5000 BP	C_4	31
Jianhe Tombs	963	—	—	2475-2221 BP	C_4	32
DabaoshanTombs	1160	—	—	2410-2180 BP	C_4	35
Lajia	1786	3580±20	3897±63	—	C_4	36
Xichengyi	1476	—	—	3880-3680 BP	C_4	37
Jiahu	70	—	—	9000-7800 BP	C_3	2
Xiaojingshan	117	—	—	8000 BP	C_3	3
lingyanghe	111	3630±145	3999±405	—	C_3	21
Baishicun	40	5840±110	6665±260	—	C_3	21
Hemudu	5	—	—	7500-5000 BP	C_3	22
Songze	2	—	—	6400-4850 BP	C_3	22
Sanxingcun	5	—	—	6500-5500 BP	C_3	26
Shilinggang	842	—	—	2700-2300 BP	C_3	33
Jinlianshan Tombs	1752	—	—	2500-2200 BP	C_3	33
Baijia	344	6710±40	7582±75	—	C_3/C_4	4
Qinglongquan	161	—	—	5000-4200 BP	C_3/C_4	9
Gouwan	171	—	—	7000-6600 BP	C_3/C_4	12
Gouwan	171	—	—	6600-6000 BP	C_3/C_4	12
Gouwan	171	—	—	5000-4600 BP	C_3/C_4	12
Mogou	2200	3190±30	3410±49	—	C_3/C_4	13
Lajigai	2100	3010±60	3183±177	—	C_3/C_4	13
Banpo	417	—	—	6800-6300 BP	C_3/C_4	16
Xiaozhujiacun	19	—	—	6650-5600 BP	C_3/C_4	18
Zhanqi	2317	—	—	3100-2950 BP	C_3/C_4	20
Mogou	2200	—	—	3750-3100 BP	C_3/C_4	20

Beishouling	601	—	—	7500-5750 BP	C_3/C_4	21
Huxizhuang	462	—	—	4600-4000 BP	C_3/C_4	21
Shangsunjiazhai	2399	—	—	3600-2600 BP	C_3/C_4	22
Wadian	128	—	—	4200-3900 BP	C_3/C_4	25
Shenmingpu	952	—	—	2475-2221 BP	C_3/C_4	28
Xigongqiao	70	—	—	5000-4500 BP	C_3/C_4	34

C_3, a pure C_3 diet of human would correspond to a collagen $\delta^{13}C$ from -22.7‰—-19.4‰.
C_4, a pure C_4 diet would result in a value between -10.7‰ and -6.8‰.
C_3/C_4, the $\delta^{13}C$ value of human bone collagen ranges between -19.4‰ and -10.7‰.

Reference

1. Barton L, Newsome SD, Chen FH, Wang H, Guilderson TP, Bettinger RL. Agricultural origins and the isotopic identity of domestication in northern China. Proceedings of the National Academy of Sciences **106**, 5523-5528 (2009).

2. Hu YW, Ambrose S H, Wang C. Stable isotopic analysis of human bones from Jiahu site, Henan, China: implications for the transition to agriculture. Journal of Archaeological Science **33**, 1319-1330 (2006).

3. Hu YW, Wang SG, Luan FS, Wang CS, Richards MP. 2008. Stable isotope analysis of humans from Xiaojingshan site: implications for understanding the origin of millet agriculture in China. Journal of Archaeological Science **35**, 2960-2965 (2008).

4. Atahan P, *et al.* Early Neolithic diets at Baijia, Wei River valley, China: stable carbon and nitrogen isotope analysis of human and faunal remains. Journal of Archaeological Science **38**, 2811-2817 (2011).

5. Liu XY, Jones MK, Zhao ZJ, Liu GX, O'Connell TC. The earliest evidence of millet as a staple crop: new light on Neolithic foodways in North China. American Journal of Physical Anthropology **149**, 283-290 (2012).

6. Atahan P, *et al.* Subsistence and the isotopic signature of herding in the Bronze Age Hexi Corridor, NW Gansu, China. Journal of Archaeological Science **38**, 1747-1753 (2011).

7. Hou LL, *et al.* Human subsistence strategy at Liuzhuang site, Henan, China during the proto-Shang culture (~ 2000–1600 BC) by stable isotopic analysis. Journal of Archaeological Science **40**, 2344-2351 (2013).

8. Ma MM, *et al.* Stable isotope analysis of human and animal remains at the Qijiaping site in middle Gansu, China. International Journal of Osteoarchaeology **25**, 923-934 (2015).

9. Guo Y, Fan Y, Hu Y, Zhu J, Richards MP. Diet transition or human migration in the Chinese Neolithic? Dietary and migration evidence from the stable isotope analysis of humans and animals from the Qinglongquan site, China. International Journal of Osteoarchaeology **28**, 85-94 (2018).

10. Lanehart RE, *et al.* Dietary adaptation during the Longshan period in China: stable isotope analyses at Liangchengzhen (southeastern Shandong). Journal of Archaeological Science **38**, 2171-2181 (2011).

11. Chen X L, *et al.* Raising practices of Neolithic livestock evidenced by stable isotope analysis in the Wei River valley, North China. International Journal of Osteoarchaeology **26**, 42-52 (2016).

12. Fu QM, Jin SA, Hu YW, Ma Z, Pan JC, Wang CS. Agricultural development and human diets in Gouwan site, Xichuan, Henan. Chinese Science Bulletin **55**, 614-620 (2010).

13. Ma MM, Dong GH, Jia X, Wang H, Cui YF, Chen FH. Dietary shift after 3600 cal yr BP and its influencing factors in northwestern China: Evidence from stable isotopes. Quaternary Science Reviews **145**, 57-70 (2016).

14. Cheung C, Jing ZC, Tang JG, Yue ZW, Richards MP. Examining social and cultural differentiation in early Bronze Age China using stable isotope analysis and mortuary patterning of human remains at Xin'anzhuang, Yinxu. Archaeological and Anthropological Sciences **9**, 799-816 (2017).

15. Guo Y, *et al.* A preliminary study of the nature of prehistoric society: with the stable isotope analysis of the Beiliu people's diet composition as an example. Huaxia Archaeology **1**, 009 (2017) (In Chinese)

16. Pechenkina E A, Ambrose S H, Ma XL, Benfer Jr, RA. Reconstructing northern Chinese Neolithic subsistence practices by isotopic analysis. Journal of Archaeological Science **32**, 1176-1189 (2005).

17. Cheung C, Jing ZC, Tang JG, Weston DA, Richards MP. Diets, social roles, and geographical origins of sacrificial victims at the royal cemetery at Yinxu, Shang China: new evidence from stable carbon, nitrogen, and sulfur isotope analysis. Journal of Anthropological Archaeology **48**, 28-45 (2017).

18. Qi WY, *et al.* On the diet of the people represented by the human bones unearthed from the upper Shuhe River valley in Shandong. Huaxi Archaeology **2**, 005 (2004) (In Chinese)

19. Cui YP, *et al.* Stable isotope analysis on human bones from Zongri site. Quaternary Sciences, 604-611 (2006).

20. Liu X, *et al.* From necessity to choice: dietary revolutions in west China in the second millennium BC. World Archaeology **46**, 661-680 (2014).

21. Cai LZ, Qiu SH. The study of 13C and ancient diets. Archaeology **10**, 449-455(1984). (In Chinese)

22. Zhang XL, Wang JX, Xian ZQ, Qiu SH. The study of ancient human diets. Archaeology **2**, 62-75 (2003). (In Chinese)

23. Ling X, Chen L, Xue XM, Zhao CC. Stable isotopic analysis of human bones from the Qingliang temple graveyard, Ruicheng county, Shanxi province. Quaternary Sciences **2**, 415-421 (2010). (In Chinese)

24. Zhou LG, Garvie-Lok, SJ. Isotopic evidence for the expansion of wheat consumption in northern China. Archaeological Research in Asia **4**, 25-35 (2015).

25. Chen XL, *et al.* Isotopic Reconstruction of the Late Longshan Period (ca. 4200–3900 BP) Dietary Complexity before the Onset of State - Level Societies at the Wadian Site in the Ying River Valley, Central Plains, China. International Journal of Osteoarchaeology **26**(5), 808-817 (2016).

26. Hu YW, Wang GF, Cui YP, Dong Y, Guan L, Wang CS. Palaeodietary study of Sanxingcun Site, Jintan, Jiangsu. Chinese Science Bulletin **52**, 660-664 (2007).

27. Wang F, *et al.* Reconstructing the food structure of ancient coastal inhabitants from Beiqian village: Stable isotopic analysis of fossil human bone. Chinese science bulletin **57**, 2148-2154 (2012).

28. Hou LL, Wang N, Lü P, Hu YW, Song GD, Wang CS. Transition of human diets and agricultural economy in Shenmingpu Site, Henan, from the Warring States to Han Dynasties. Science China Earth Sciences **55**, 975-982 (2012).

29. Ma Y, *et al.* Isotopic perspectives (δ13C, δ15N, δ34S) of diet, social complexity, and animal husbandry during the proto - Shang period (ca. 2000–1600 BC) of China. American journal of physical anthropology **160**, 433-445 (2016).

30. Chen XL, *et al.* The subsistence patterns of the Shengedaliang site (~4,000 yr BP) revealed by stable carbon and nitrogen isotopes in northern Shaanxi, China. Science China Earth Sciences **60**: 268–276 (2017).

31. Zhang XL, *et al.* Studies on diet of the ancient people of the Yangshao cultural sites in the Central Plain. ACTA ANTHROPOLOGICAL SINICA **2**, 197-207 (2010). (In Chinese)

32. Ling X, Wang WS, Chen L, Sun LJ, Hu YW. Stable isotope analysis of Qin human bones from Jianhe cemetery in Baoji city, Shaanxi province. Archaeology and Cultural Relics **1**, 95-98 (2010).

33. Ren LL, *et al.* Human paleodiet and animal utilization strategies during the Bronze Age in northwest Yunnan Province, southwest China. PloS one **12**(5), e0177867 (2017).

34. Hu YW, He DL, Dong Y, Wang CS, Gao MK, Lan YF. Stable isotopic analysis on human bones from the Xigongqiao site, Tengzhou, Shandong. Quaternary Sciences **5**, 561-567 (2005). (In Chinese)

35. Zhang XY, Zhang X, Suo MJ, Wei D, Hu YW. The influence of agriculture in the process of population integration and cultural interaction during the Eastern Zhou Period in central-south, Inner Mongolia: Carbon and nitrogen stable isotope analysis of human bones from the Dabaoshan cemetery, Helingeer County. Science China Earth Sciences **61**, 205-214 (2018).

36. Zhang XL, Ye ML. A preliminary study on ancient diets in Lajia site—Carbon and nitrogen stable isotope analysis on human bones from disaster ruins in Lajia site. Relics from South, 197-202 (2016). (In Chinese)

37. Zhang XL, *et al.* A preliminary analysis on Paleo-diets of ancient humans in Xichengyi site, Zhangye Gansu. Archaeology, 110-120 (2015). (In Chinese)

Table S7. Distribution of haplogroups in different geographic regions based on HVS and whole mitogenome datasets.

	Tibet			Northwest China		North China		South China		Southwestern China		North Asia		Southeast Asia		South Asia		Central and West Asia		Western Eurasia		Japan and Korea	
	671 (This study	7481[a]	201[b]	1195[a]	53[b]	975[a]	232[b]	3975[a]	97[b]	3530[a]	98[b]	4688[a]	832[b]	8040[a]	1266[b]	6832[a]	688[b]	4543[a]	997[b]	1042[a]	4535[b]	2188[a]	790[b]
A	11	369	1	84	1	55	3	104	3	89	0	68	3	35	1	50	0	42	2	2	0	47	0
A8a	1	0	1	0	0	1	0	0	0	0	0	16	7	0	0	0	0	23	0	0	0	6	0
A11a1a†	29	292	7	7	0	1	0	2	0	2	0	0	0	0	0	0	0	0	0	0	0	0	0
A11b	3	64	3	3	0	0	0	0	0	29	0	0	0	0	0	0	0	0	0	0	0	0	0
A14	1	29	0	0	0	7	0	4	0	14	0	0	1	30	4	8	0	9	2	0	0	3	0
A14a†	6	0	6	0	0	0	0	0	0	0	0	0	0	0	0	0	0	0	0	0	0	0	0
A14b1†	3	0	3	0	0	0	0	0	0	0	0	0	0	0	0	0	0	0	0	0	0	0	0
A15a†	6	NA	1	NA	1	NA	2	NA	0	NA	0	NA	0	NA	0	NA	0	NA	0	NA	0	NA	0
A15c†	0	NA	0	NA	0	NA	0	NA	0	NA	0	NA	0	NA	0	NA	0	NA	0	NA	0	NA	0
A15c1†	1	NA	14	NA	0	NA	1	NA	0	NA	0	NA	0	NA	0	NA	0	NA	0	NA	0	NA	0
A15c2†	4	NA	0	NA	0	NA	0	NA	0	NA	0	NA	0	NA	0	NA	0	NA	0	NA	0	NA	0
A17	2	NA	0	NA	0	NA	1	NA	0	NA	0	NA	0	NA	10	NA	0	NA	1	NA	0	NA	0
A17a2†	3	NA	0	NA	0	NA	0	NA	0	NA	0	NA	0	NA	0	NA	0	NA	0	NA	0	NA	0
A21†	14	NA	3	NA	0	NA	0	NA	0	NA	0	NA	0	NA	0	NA	1	NA	0	NA	0	NA	0
A6	2	NA	4	NA	0	NA	0	NA	1	NA	0	NA	1	NA	0	NA	1	NA	0	NA	0	NA	0
A6b†	1	0	4	0	0	0	0	0	0	0	0	0	0	0	0	0	1	0	0	0	0	0	0
N9a1	1	13	1	5	0	7	0	21	3	15	0	6	2	3	3	0	0	16	1	0	0	7	2
N9a5	1	0	0	1	0	1	0	0	0	0	0	0	0	0	0	0	0	1	0	0	0	7	3
N11a1	1	13	0	3	0	0	0	1	0	7	0	0	0	0	0	0	0	2	0	0	0	0	0
X2p	1	NA	0	NA	0	NA	0	NA	0	NA	0	NA	0	NA	0	NA	6	NA	1	NA	0	NA	0
B4a	1	NA	0	NA	0	NA	0	NA	0	NA	0	NA	0	NA	0	NA	0	NA	0	NA	0	NA	0
B4a1	1	0	0	0	1	0	3	7	1	14	0	0	0	84	18	1	0	6	3	0	0	0	13
B4a4	4	57	0	8	0	2	3	19	1	19	0	4	4	3	0	5	0	1	0	0	0	4	2
B4h1	2	1	0	0	0	0	1	4	1	2	0	0	0	0	2	0	0	7	0	0	0	0	0
B4b1a2	1	NA	0	NA	0	NA	0	NA	0	NA	2	NA	0	NA	14	NA	0	NA	0	NA	0	NA	0
B4d1'2'3	7	NA	0	NA	0	NA	0	NA	1	NA	0	NA	0	NA	0	NA	0	NA	0	NA	0	NA	0
B4b4†	4	NA	0	NA	0	NA	0	NA	0	NA	0	NA	0	NA	0	NA	0	NA	0	NA	0	NA	0
B4c1b2c2	1	5	0	1	0	4	3	0	1	5	0	0	0	3	2	0	1	0	0	0	0	1	1
B5a1c2	1	NA	0	NA	0	NA	0	NA	0	NA	0	NA	0	NA	0	NA	0	NA	0	NA	0	NA	0
B5b	2	60	0	6	0	8	1	0	1	19	0	6	0	69	1	19	0	9	0	0	0	14	0
B5b1	1	NA	0	NA	0	NA	0	NA	0	NA	0	NA	0	NA	0	NA	0	NA	0	NA	0	NA	0
B5b4	1	1	0	0	0	1	0	0	0	0	0	5	1	0	0	1	0	3	0	0	0	3	0
B6a1a	1	0	0	0	0	0	0	0	0	0	0	0	0	27	9	0	0	0	0	0	0	0	0
U1a1c1a	1	0	1	0	0	0	0	0	0	0	0	0	0	0	0	1	0	0	0	0	0	0	0
U2c1	1	NA	0	NA	0	NA	0	NA	0	NA	0	NA	0	NA	0	NA	1	NA	0	NA	0	NA	0
U2e1h	3	3	0	4	0	0	0	0	0	0	0	5	0	0	0	9	4	6	8	1	1	0	0
U4a1	1	0	0	2	0	0	0	0	0	0	0	52	1	0	0	3	7	16	4	9	21	0	0
U7a	1	31	0	4	0	1	0	0	0	0	0	23	0	4	0	79	2	60	8	2	0	0	0
U7a3b	1	0	0	0	0	0	0	0	0	0	0	0	0	4	0	5	3	2	4	0	0	0	0
H15a1	1	NA	0	NA	0	NA	0	NA	0	NA	0	NA	0	NA	0	NA	0	NA	2	NA	2	NA	0
H2	2	NA	0	NA	0	NA	0	NA	0	NA	0	NA	0	NA	0	NA	7	NA	35	NA	0	NA	0
H7b2	2	NA	0	NA	0	NA	0	NA	0	NA	0	NA	0	NA	0	NA	0	NA	0	NA	7	NA	0
H101	1	0	0	0	0	0	1	0	0	0	0	6	0	0	0	0	2	14	4	0	0	0	0
H14	1	0	0	0	0	0	0	0	0	0	0	0	0	0	4	0	1	6	8	1	8	0	0
HV19	1	4	0	0	0	0	0	0	0	0	0	0	0	0	0	0	0	2	0	0	0	0	0
F1a1a1	1	NA	0	NA	0	NA	0	NA	0	NA	0	NA	0	NA	72	NA	0	NA	0	NA	0	NA	0
F1a1c	1	NA	0	NA	1	NA	3	NA	0	NA	1	NA	0	NA	0	NA	1	NA	0	NA	0	NA	1

	Tibet			Northwest China		North China		South China		Southwestern China		North Asia		Southeast Asia		South Asia		Central and West Asia		Western Eurasia		Japan and Korea	
	671 (This study)	7481[a]	201[b]	1195[a]	53[b]	975[a]	232[b]	3975[a]	97[b]	3530[a]	98[b]	4688[a]	832[b]	8040[a]	1266[b]	6832[a]	688[b]	4543[a]	997[b]	1042[a]	4535[b]	2188[a]	790[b]
F1c1a	3	138	1	14	0	7	0	0	1	6	0	1	0	3	4	3	2	1	0	0	0	2	0
F1c1a1a†	18	NA	2	NA	0	NA	0	NA	0	NA	0	NA	0	NA	0	NA	0	NA	0	NA	0	NA	0
F1b1	7	NA	1	NA	0	NA	1	NA	0	NA	0	NA	0	NA	0	NA	0	NA	4	NA	0	NA	2
F1b1c	4	35	0	4	0	3	0	0	0	17	0	14	0	2	0	6	1	4	0	0	0	1	0
F1d	7	NA	1	NA	0	NA	3	NA	1	NA	0	NA	0	NA	1	NA	0	NA	1	NA	0	NA	1
F1d1	11	NA	0	NA	0	NA	1	NA	0	NA	0	NA	0	NA	1	NA	0	NA	0	NA	0	NA	1
F1d1a1	3	4	0	0	0	0	0	0	0	0	0	0	0	0	0	7	0	1	0	0	0	0	0
F1d1a2†	8	5	0	0	0	0	0	0	0	0	0	0	0	0	0	0	0	0	0	0	0	0	0
F1d2†	7	NA	1	NA	0	NA	0	NA	0	NA	0	NA	0	NA	0	NA	0	NA	0	NA	0	NA	0
F1g†	21	NA	0	NA	0	NA	1	NA	1	NA	0	NA	0	NA	5	NA	1	NA	1	NA	0	NA	0
F1g1†	2	NA	1	NA	0	NA	0	NA	0	NA	1	NA	0	NA	0	NA	0	NA	0	NA	0	NA	0
F1g2†	3	NA	0	NA	0	NA	0	NA	0	NA	0	NA	0	NA	0	NA	0	NA	0	NA	0	NA	0
F2a1	1	0	0	4	0	2	1	0	0	29	0	0	0	8	0	0	0	0	0	0	0	0	0
F2b1	1	0	0	1	1	3	4	25	0	7	0	9	1	8	4	3	0	9	0	0	0	2	0
F2g	0	NA	0	NA	0	NA	0	NA	0	NA	0	NA	0	NA	0	NA	1	NA	0	NA	0	NA	0
F2g1a	3	3	0	5	0	0	0	0	0	10	0	0	0	15	0	0	0	0	0	0	0	0	0
F2c1	1	1	0	0	0	0	1	0	0	0	0	0	0	0	0	0	0	0	0	0	0	0	0
K1a4	1	NA	0	NA	0	NA	0	NA	0	NA	0	NA	0	NA	1	NA	0	NA	11	NA	54	NA	0
T1a1	1	NA	0	NA	0	NA	0	NA	0	NA	0	NA	0	NA	0	NA	3	NA	7	NA	5	NA	0
T1a1b1	1	NA	0	NA	0	NA	0	NA	0	NA	0	NA	0	NA	0	NA	0	NA	8	NA	5	NA	0
M8a1	2	NA	0	NA	0	NA	0	NA	0	NA	0	NA	4	NA	0	NA	0	NA	0	NA	0	NA	0
M8a2a1	1	0	0	1	0	1	2	0	1	11	0	0	0	5	5	0	0	2	0	0	0	0	1
C4a1a1a	12	NA	0	NA	0	NA	0	NA	0	NA	0	NA	0	NA	0	NA	2	NA	6	NA	9	NA	0
C4a2b†	5	NA	1	NA	0	NA	0	NA	0	NA	0	NA	0	NA	0	NA	0	NA	0	NA	0	NA	0
C4a2b2†	2	39	15	2	0	0	1	0	0	11	0	1	0	10	0	10	0	0	0	0	0	0	0
C4a2c	2	NA	0	NA	0	NA	0	NA	0	NA	0	NA	1	NA	0	NA	0	NA	2	NA	0	NA	0
C4a2c1	1	1	0	0	0	1	0	0	0	0	0	7	0	0	0	9	11	9	1	0	0	1	0
C4a2c2	2	5	0	2	0	1	0	4	0	0	0	2	0	1	0	1	6	1	0	0	0	0	0
C4a2c3†	3	NA	0	NA	0	NA	0	NA	0	NA	0	NA	0	NA	0	NA	0	NA	0	NA	0	NA	0
C4b1	1	NA	0	NA	0	NA	0	NA	0	NA	0	NA	37	NA	0	NA	0	NA	5	NA	0	NA	0
C4d	6	NA	2	NA	0	NA	1	NA	0	NA	0	NA	0	NA	2	NA	0	NA	0	NA	0	NA	0
C7	1	NA	0	NA	0	NA	0	NA	0	NA	0	NA	2	NA	8	NA	0	NA	0	NA	0	NA	0
C7a	2	NA	1	NA	0	NA	1	NA	0	NA	4	NA	0	NA	7	NA	0	NA	0	NA	0	NA	0
C7a1	1	NA	0	NA	0	NA	0	NA	1	NA	1	NA	2	NA	8	NA	0	NA	0	NA	0	NA	0
C7b	1	NA	0	NA	0	NA	0	NA	0	NA	0	NA	0	NA	0	NA	2	NA	0	NA	0	NA	0
Z3a	5	NA	0	NA	1	NA	0	NA	0	NA	0	NA	0	NA	2	NA	0	NA	0	NA	0	NA	0
Z3a1a	3	NA	0	NA	1	NA	1	NA	0	NA	0	NA	0	NA	1	NA	0	NA	0	NA	0	NA	0
Z4a	1	26	1	4	1	3	1	3	2	13	0	0	0	1	0	0	0	3	0	0	0	4	1
Z7	2	NA	1	NA	0	NA	0	NA	0	NA	0	NA	0	NA	0	NA	5	NA	0	NA	0	NA	0
D4	3	NA	1	NA	2	NA	0	NA	1	NA	0	NA	3	NA	9	NA	1	NA	4	NA	0	NA	3
D4b2b	2	NA	0	NA	0	NA	1	NA	0	NA	1	NA	2	NA	1	NA	0	NA	1	NA	0	NA	0
D4b2b8	9	NA	0	NA	0	NA	0	NA	0	NA	0	NA	0	NA	0	NA	0	NA	0	NA	0	NA	1
D4b2b9	4	NA	0	NA	0	NA	0	NA	0	NA	0	NA	0	NA	0	NA	0	NA	0	NA	0	NA	0
D4b2b10	4	NA	0	NA	0	NA	0	NA	0	NA	0	NA	0	NA	0	NA	0	NA	0	NA	0	NA	0
D4g2a1c1†	3	NA	0	NA	0	NA	1	NA	1	NA	2	NA	0	NA	6	NA	0	NA	0	NA	0	NA	0
D4h1c2a†	5	24	0	2	0	2	0	1	0	2	0	0	0	0	0	1	0	1	0	0	0	4	0
D4h4	1	0	0	0	0	1	0	0	0	0	0	2	2	0	0	0	0	0	2	0	0	0	1
D4i	1	25	0	7	1	5	1	6	1	8	0	10	0	0	0	0	0	9	0	0	0	5	2
D4j1a1	1	NA	1	NA	0	NA	1	NA	0	NA	0	NA	4	NA	8	NA	0	NA	1	NA	0	NA	0

	Tibet			Northwest China		North China		South China		Southwestern China		North Asia		Southeast Asia		South Asia		Central and West Asia		Western Eurasia		Japan and Korea	
	671 (This study)	7481[a]	201[b]	1195[a]	53[b]	975[a]	232[b]	3975[a]	97[b]	3530[a]	98[b]	4688[a]	832[b]	8040[a]	1266[b]	6832[a]	688[b]	4543[a]	997[b]	1042[a]	4535[b]	2188[a]	790[b]
D4j1a1c†	2	NA	1	NA	0	NA	0	NA	0	NA	0	NA	0	NA	0	NA	0	NA	0	NA	0	NA	0
D4j1a1d†	5	1	1	0	0	0	0	0	0	3	0	1	0	8	0	0	0	0	1	0	0	0	0
D4j1a2e†	5	0	0	0	0	0	0	0	0	0	0	0	0	0	0	0	0	0	0	0	0	0	0
D4j1a2f†	6	NA	2	NA	0	NA	0	NA	0	NA	0	NA	1	NA	0	NA	0	NA	1	NA	0	NA	0
D4j1b	3	NA	0	NA	0	NA	0	NA	0	NA	1	NA	0	NA	3	NA	0	NA	2	NA	0	NA	0
D4j3	14	100	0	17	0	9	2	1	0	17	0	4	0	0	1	0	0	2	0	0	0	7	0
D4o2a	2	7	0	0	0	3	3	0	0	1	0	61	13	0	0	0	15	11	0	0	0	4	0
D4q	3	35	0	2	0	0	0	5	0	13	0	0	0	5	0	20	15	1	0	0	0	0	0
D4s	4	NA	1	NA	0	NA	0	NA	0	NA	0	NA	0	NA	0	NA	0	NA	2	NA	0	NA	0
D5a2a1	12	102	3	18	0	16	3	28	2	28	0	20	1	20	0	17	0	27	0	0	0	17	8
D6a1	2	NA	0	NA	0	NA	0	NA	0	NA	0	NA	0	NA	0	NA	0	NA	0	NA	0	NA	1
G1	2	NA	NA	NA	NA	NA	NA	NA	NA	NA	NA	NA	NA	NA	NA	NA	NA	NA	NA	NA	NA	NA	NA
G1a	2	NA	0	NA	0	NA	0	NA	0	NA	0	NA	0	NA	0	NA	0	NA	0	NA	0	NA	0
G1a1	1	2	0	3	0	2	0	2	0	1	0	1	0	1	0	1	0	14	5	1	0	28	0
G1a2	1	11	0	1	0	1	1	1	0	1	0	0	0	2	0	0	0	0	0	0	0	0	0
G1c	1	NA	1	NA	0	NA	0	NA	0	NA	0	NA	1	NA	0	NA	0	NA	0	NA	0	NA	0
G2a1	13	NA	2	NA	1	NA	0	NA	0	NA	0	NA	4	NA	0	NA	0	NA	0	NA	12	NA	2
G2a1i	5	8	1	3	0	1	0	2	0	17	0	0	0	2	1	0	3	0	0	0	0	0	0
G2a1f	0	0	0	1	0	0	1	3	0	0	0	0	0	6	0	0	0	0	0	0	0	0	0
G2a1f2†	4	4	0	2	0	0	0	0	0	1	0	0	0	0	0	0	0	0	0	0	0	0	0
G2a1h	0	2	0	0	0	1	0	0	0	0	0	0	0	1	0	5	0	3	0	0	0	2	0
G2a1h1†	6	NA	0	NA	0	NA	0	NA	0	NA	0	NA	0	NA	0	NA	0	NA	0	NA	0	NA	0
G2a'c	1	NA	1	NA	0	NA	0	NA	0	NA	0	NA	0	NA	0	NA	0	NA	0	NA	0	NA	0
G2b1	3	NA	1	NA	0	NA	0	NA	0	NA	0	NA	0	NA	0	NA	0	NA	0	NA	0	NA	0
G2b1a	1	2	0	0	0	1	0	0	0	0	0	0	0	1	1	5	0	3	0	0	0	2	0
G2b1b†	10	20	1	3	0	0	0	0	0	22	0	0	0	0	0	0	0	2	0	0	0	0	3
G2b2a	2	NA	0	NA	0	NA	0	NA	0	NA	0	NA	0	NA	0	NA	0	NA	0	NA	0	NA	0
G3a1†	1	132	1	10	0	0	0	0	0	18	0	1	0	0	0	0	0	3	1	0	0	0	0
G3a1a†	11	NA	2	NA	0	NA	0	NA	0	NA	0	NA	0	NA	0	NA	0	NA	0	NA	0	NA	0
G3a2	2	NA	0	NA	0	NA	0	NA	0	NA	0	NA	0	NA	0	NA	0	NA	0	NA	0	NA	0
G3b†	5	NA	1	NA	0	NA	0	NA	0	NA	0	NA	0	NA	0	NA	0	NA	0	NA	0	NA	0
G3b1†	6	NA	1	NA	0	NA	0	NA	0	NA	0	NA	0	NA	0	NA	2	NA	0	NA	0	NA	0
M12a1b	1	1	0	2	0	2	0	3	0	14	0	0	0	2	11	2	0	0	0	0	0	0	0
M5a	1	NA	0	NA	0	NA	0	NA	0	NA	0	NA	0	NA	4	NA	13	NA	2	NA	0	NA	0
M10a1b	5	NA	0	NA	0	NA	2	NA	1	NA	0	NA	0	NA	0	NA	0	NA	0	NA	0	NA	0
M11a	2	NA	0	NA	0	NA	0	NA	0	NA	0	NA	1	NA	0	NA	0	NA	0	NA	0	NA	0
M11a2†	6	32	4	1	0	0	0	1	0	9	0	0	0	2	0	0	0	0	0	0	0	0	0
M13a1b†	17	92	3	4	0	2	0	0	0	32	0	8	4	2	0	1	0	5	0	0	0	0	0
M13a2†	20	197	8	5	0	0	0	0	0	10	0	0	0	4	0	0	0	0	0	0	0	0	0
M61	1	0	0	1	0	0	0	8	0	12	2	0	0	3	8	7	0	0	0	0	0	0	0
M61a	2	51	0	1	0	1	0	0	0	8	0	1	0	1	0	1	0	0	0	0	0	0	0
M33a1a	1	NA	0	NA	0	NA	0	NA	0	NA	0	NA	0	NA	0	NA	0	NA	0	NA	0	NA	0
M62a†	6	16	1	0	0	0	0	0	2	0	0	0	0	0	0	0	0	0	0	0	0	0	0
M62b1†	7	60	2	0	0	0	0	1	0	7	0	0	0	0	0	0	0	0	0	0	0	0	0
M62b1a1†	6	31	7	6	0	0	0	0	0	1	0	0	0	0	0	0	0	0	0	0	0	0	0
M62b2†	4	NA	4	NA	0	NA	0	NA	0	NA	0	NA	0	NA	0	NA	0	NA	0	NA	0	NA	0
M7b1a1a3	2	NA	0	NA	0	NA	1	NA	2	NA	3	NA	0	NA	31	NA	0	NA	0	NA	0	NA	1
M7b1a1b	1	0	0	0	0	0	0	0	0	0	2	0	0	0	18	0	0	0	1	0	0	0	0
M7c1a1a1	1	NA	0	NA	0	NA	1	NA	1	NA	0	NA	0	NA	0	NA	0	NA	0	NA	0	NA	0

	Tibet			Northwest China		North China		South China		Southwestern China		North Asia		Southeast Asia		South Asia		Central and West Asia		Western Eurasia		Japan and Korea	
	671 (This study)	7481[a]	201[b]	1195[a]	53[b]	975[a]	232[b]	3975[a]	97[b]	3530[a]	98[b]	4688[a]	832[b]	8040[a]	1266[b]	6832[a]	688[b]	4543[a]	997[b]	1042[a]	4535[b]	2188[a]	790[b]
M9a1a1c1b1a†	100	NA	25	NA	0	NA	0	NA	0	NA	0	NA	1	NA	0	NA	10	NA	0	NA	0	NA	0
M9a1a1c1b1a1	2	NA	3	NA	0	NA	0	NA	0	NA	0	NA	0	NA	0	NA	1	NA	0	NA	0	NA	0
M9a1a1c1b1a2	9	NA	1	NA	0	NA	0	NA	0	NA	0	NA	0	NA	0	NA	3	NA	0	NA	0	NA	0
M9a1a2†	5	38	1	3	0	1	0	0	0	5	0	0	0	3	0	24	0	0	0	0	0	0	0
M9a1b	3	NA	1	NA	0	NA	0	NA	0	NA	0	NA	0	NA	0	NA	0	NA	0	NA	0	NA	0
M9a1b1	14	359	3	17	0	0	0	1	0	28	0	2	1	29	1	63	1	2	0	0	0	0	0
M9a1b1c†	15	NA	4	NA	0	NA	0	NA	0	NA	0	NA	0	NA	0	NA	0	NA	0	NA	0	NA	0
M9a1b1d	6	NA	1	NA	0	NA	0	NA	0	NA	0	NA	0	NA	0	NA	0	NA	0	NA	0	NA	0
M9a1b1e	8	NA	0	NA	0	NA	0	NA	0	NA	0	NA	0	NA	0	NA	7	NA	0	NA	0	NA	0
M9a1b2	3	NA	0	NA	0	NA	0	NA	0	NA	0	NA	2	NA	0	NA	0	NA	0	NA	0	NA	0

Note: [a]HVS dataset

[b]Complete mitogenome dataset.

[c]NA means this haplogroup is defined by coding-region variants, thus cannot be searched in the HVS dataset.

Table S8. Coalescent ages of haplogroups with *de novo* differentiations in Tibetans.

	Number of samples for calculation	Number of Tibetan samples (including Sherpa)	Number of Tibetan samples in this study	Coding Region (Soares et al. 2009)			Synonymous Positions (Soares et al. 200			Complete Genome (Soares et al. 2009			Period†
				**Age (lower; upper)**	**ρ (σ)**	**$\rho/n\sigma^2$**	**Age (lower; upper)**	**ρ (σ)**	**$\rho/n\sigma^2$**	**Age (lower; upper)**	**ρ (σ)**	**$\rho/n\sigma^2$**	
A6b	5	5	1	4.24 (2.51; 5.97)	1.2(0.49)	1.00	7.87 (4.33; 11.41)	1(0.45)	0.99	6.20 (4.14; 8.27)	2.4(0.8)	0.75	Paleolithic
A11a1a‡	47	42	29	5.11 (4.37; 5.86)	1.45(0.21)	0.73	4.25 (3.23; 5.27)	0.54(0.13)	0.67	8.03 (5.82; 10.24)	3.11 (0.85)	0.09	Neolithic
A11a1a1	3	1	1	5.90 (2.37; 9.43)	1.67(1.00)	0.56	5.27 (0.00; 10.55)	0.67(0.67)	0.50	6.02 (3.15; 8.89)	2.33(1.11)	0.63	Neolithic
A11a1a2	3	3	0	4.70 (1.80; 7.60)	1.33(0.82)	0.66	2.60 (0.00; 5.20)	0.33(0.33)	1.01	6.90 (3.93; 9.87)	2.67(1.15)	0.67	Neolithic
A11a1a3	3	3	3	4.70 (3.53; 5.86)	1.33(0.33)	4.07	0.00 (0.00; 0.00)	0.00(0.00)	-	3.44 (1.71; 5.17)	1.33(0.67)	0.99	Neolithic
A11a1a4	3	3	3	2.37 (0.71; 4.03)	0.67(0.47)	1.01	2.60 (0.00; 5.20)	0.33(0.33)	1.01	3.44 (1.32; 5.56)	1.33(0.82)	0.66	Neolithic
A14a	6	6	6	8.83 (4.88; 12.79)	2.5(1.12)	0.33	14.41 (7.32; 21.49)	1.83(0.9)	0.38	9.05 (5.58; 12.51)	3.5(1.34)	0.32	Paleolithic
A14a1	4	4	4	0.88 (0.00; 1.77)	0.25(0.25)	1.00	1.97 (0.00; 3.94)	0.25(0.25)	1.00	1.94 (0.49; 3.39)	0.75(0.56)	0.60	Neolithic
A14b1	3	3	3	0.00 (0.00; 0.00)	0.00(0.00)	-	0.00 (0.00; 0.00)	0.00(0.00)	-	0.00 (0.00; 0.00)	0.00(0.00)	-	Neolithic
A15a‡	9	8	7	4.71(2.33;7.07)	1.33(0.67)	0.33	8.76 (3.78; 13.70)	1.11(0.63)	0.31	5.17 (2.92; 7.42)	2(0.87)	0.29	Neolithic
A15c	20	20	5	14.66 (8.55; 20.77)	4.15(1.73)	0.07	9.84 (2.28; 17.40)	1.25(0.96)	0.07	14.48 (9.54; 19.41)	5.6(1.91)	0.08	Paleolithic
A15c1	15	15	1	2.37 (1.20; 3.53)	0.67(0.33)	0.41	2.13 (0.87; 3.38)	0.27(0.16)	0.70	4.99 (2.25; 7.73)	1.93(1.06)	0.11	Neolithic
A15c2	4	4	4	2.65 (1.13; 4.17)	0.75(0.43)	1.01	1.97 (0.00; 3.94)	0.25(0.25)	1.00	4.52 (2.20; 6.85)	1.75(0.9)	0.54	Neolithic
A17a2	3	3	3	3.53 (0.88; 6.18)	1(0.75)	0.59	0.00 (0.00; 0.00)	0(0)	-	7.76 (4.21; 11.30)	3(1.37)	0.53	Neolithic
A21‡	20	17	14	2.82(1.97; 3.68)	0.80(0.24)	1.70	4.40 (3.16; 5.64)	1.70(0.48)	0.75	5.91 (4.04; 7.78)	0.75(0.23)	0.71	Neolithic
A21a	7	5	3	2.02(0.78;3.25)	0.57(0.35)	0.71	1.85 (0.87; 2.83)	0.71(0.38)	0.43	3.78 (0.86; 5.97)	0.43(0.32)	0.60	Neolithic
A21b	10	9	9	2.82(1.59;4.06)	0.8(0.35)	2.40	6.20 (3.90; 8.50)	2.4(0.89)	0.50	3.94 (1.89; 5.99)	0.5(0.26)	0.74	Neolithic
A21b1	7	7	7	3.03(1.62;4.45)	0.86(0.4)	2.00	5.17 (3.28; 7.06)	2(0.73)	0.43	3.38 (1.42; 5.36)	0.43(0.25)	0.98	Neolithic
A21b1a	3	3	3	2.35(0;4.73)	0.67(0.67)	2.00	5.17 (2.19; 8.14)	2(1.15)	0.00	0 (0; 0)	0(0)	-	Neolithic
A21c	3	3	2	4.71(2.33;7.07)	1.33(0.67)	1.67	4.32 (2.38; 6.26)	1.67(0.75)	0.33	2.60 (0; 5.20)	0.33(0.33)	1.01	Neolithic
B4d4	4	4	4	4.42 (2.44; 6.39)	1.25(0.56)	1.00	5.90 (2.52; 9.29)	0.75(0.43)	1.01	6.46 (4.21; 8.71)	2.5(0.87)	0.83	Neolithic
C4a1a1a	17	12	12	4.77 (3.39; 6.15)	1.35(0.39)	0.52	5.59 (3.07; 8.11)	0.71(0.32)	0.41	6.85 (5.17; 8.53)	2.65(0.65)	0.37	Neolithic
C4a1a1a1	6	4	4	5.30 (2.86; 7.74)	1.5(0.69)	0.53	6.53 (2.60; 10.47)	0.83(0.5)	0.55	3.88 (2.09; 5.66)	1.5(0.69)	0.53	Neolithic
C4a1a1a2	4	4	4	2.65 (1.13; 4.17)	0.75(0.43)	1.01	0.00 (0.00; 0.00)	0(0)	-	3.88 (2.30; 5.45)	1.5(0.61)	1.01	Neolithic
C4a1a1a3	5	3	3	3.53 (1.66; 5.41)	1(0.53)	0.71	3.15 (0.00; 6.30)	0.4(0.4)	0.50	5.69 (3.57; 7.81)	2.2(0.82)	0.65	Neolithic
C4a2b	26	19	5	13.18 (8.48; 17.88)	3.73(1.33)	0.08	18.74 (10.86; 26.61)	2.38(1)	0.09	13.31 (9.31; 17.32)	5.15(1.55)	0.08	Paleolithic
C4a2b2	20	15	1	8.13 (4.66; 11.59)	2.3(0.98)	0.12	13.78 (7.40; 20.15)	1.75(0.81)	0.13	7.37 (4.78; 9.95)	2.85(1)	0.14	Paleolithic
C4a2b2a	11	11	0	0.32 (0.00; 0.64)	0.09(0.09)	1.01	0.71 (0.00; 1.42)	0.09(0.09)	1.01	1.16 (0.65; 1.68)	0.45(0.2)	1.02	Neolithic
C4a2c3‡	3	3	3	4.70 (1.80; 7.60)	1.33(0.82)	0.66	0.00 (0.00; 0.00)	0.00(0.00)	-	4.32 (2.04; 6.59)	1.67(0.88)	0.72	Neolithic
C4d2	8	8	6	3.96 (2.65; 5.26)	1.12(0.37)	1.02	5.90 (3.46; 8.34)	0.75(0.31)	0.98	4.52 (3.08; 5.97)	1.75(0.56)	0.70	Neolithic

	Number of samples for calculation	Number of Tibetan samples (including Sherpa)	Number of Tibetan samples in this study	Coding Region (Soares et al. 2009)			Synonymous Positions (Soares et al. 200[illegible]			Complete Genome (Soares et al. 200[illegible]			Period†
				**Age (lower; upper)**	**ρ (σ)**	**$ρ/nσ^2$**	**Age (lower; upper)**	**ρ (σ)**	**$ρ/nσ^2$**	**Age (lower; upper)**	**ρ (σ)**	**$ρ/nσ^2$**	
D4b2b8	9	9	9	5.51 (3.74; 7.28)	1.56(0.50)	0.69	10.47 (6.77; 14.17)	1.33(0.47)	0.67	7.76 (5.84; 9.67)	3.00(0.74)	0.61	Paleolithic
D4b2b8a	4	4	4	8.83 (5.51; 12.15)	2.50(0.94)	0.71	17.71 (10.63; 24.80)	2.25(0.90)	0.69	7.76 (5.17; 10.34)	3.00(1.00)	0.75	Paleolithic
D4b2b9	4	4	4	3.53 (1.77; 5.30)	1(0.5)	1.00	3.94 (1.18; 6.69)	0.5(0.35)	1.02	2.59 (1.29; 3.88)	1(0.5)	1.00	Neolithic
D4b2b10	4	4	4	4.42 (2.08; 6.75)	1.25(0.66)	0.72	7.87 (3.07; 12.67)	1(0.61)	0.67	5.82 (3.67; 7.96)	2.25(0.83)	0.82	Neolithic
D4g2a1c1‡	3	3	3	4.70 (2.33; 7.07)	1.33(0.67)	0.99	2.60 (0.00; 5.20)	0.33(0.33)	1.01	4.32 (2.38; 6.26)	1.67(0.75)	0.99	Neolithic
D4h1c2a	5	5	5	9.89 (6.15; 13.64)	2.80(1.06)	0.50	14.17 (7.71; 20.62)	1.80(0.82)	0.54	10.86 (7.32; 14.40)	4.20(1.37)	0.45	Paleolithic
D4h1c2a1	3	3	3	1.17 (0.00; 2.33)	0.33(0.33)	1.01	2.60 (0.00; 5.20)	0.33(0.33)	1.01	0.85 (0.00; 1.71)	0.33(0.33)	1.01	Neolithic
D4j1a1c	3	3	2	2.37 (0.71; 4.03)	0.67(0.47)	1.01	0.00 (0.00; 0.00)	0(0)	-	1.73 (0.52; 2.95)	0.67(0.47)	1.01	Neolithic
D4j1a1d	8	7	5	4.03 (2.01; 6.04)	1.14(0.57)	0.50	6.77 (2.60; 10.94)	0.86(0.53)	0.44	4.06 (2.38; 5.74)	1.57(0.65)	0.53	Neolithic
D4j1a1d1	3	2	2	2.37 (0.00; 4.73)	0.67(0.67)	0.50	5.27 (0.00; 10.55)	0.67(0.67)	0.50	1.73 (0.00; 3.46)	0.67(0.67)	0.50	Neolithic
D4j1a1e	5	5	5	0.00 (0.00; 0.00)	0(0)	-	0.00 (0.00; 0.00)	0(0)	-	0.52 (0.00; 1.03)	0.2(0.2)	1.00	Neolithic
D4j1a1f‡	7	6	6	4.56 (3.04; 6.08)	1.29(0.43)	1.00	5.59 (3.07; 8.11)	0.71(0.32)	0.99	4.42 (3.05; 5.79)	1.71(0.53)	0.87	Neolithic
D4j3b	14	14	14	3.04 (1.91; 4.17)	0.86(0.32)	0.60	1.65 (0.39; 2.91)	0.21(0.16)	0.59	4.42 (3.23; 5.61)	1.71(0.46)	0.58	Neolithic
D4j3b1	3	3	3	2.37 (0.00; 4.73)	0.67(0.67)	0.50	0.00 (0.00; 0.00)	0(0)	-	2.59 (0.65; 4.52)	1(0.75)	0.59	Neolithic
D4s	8	5	4	8.83 (5.19; 12.47)	2.5(1.03)	0.29	6.85 (4.25; 9.45)	0.87(0.33)	1.00	9.69 (6.67; 12.72)	3.75(1.17)	0.34	Paleolithic
D4s1‡	7	4	4	4.56 (2.61; 6.50)	1.29(0.55)	0.61	4.49 (2.20; 6.77)	0.57(0.29)	0.97	6.28 (4.16; 8.40)	2.43(0.82)	0.52	Neolithic
D4s1a	3	3	3	1.17 (0.00; 2.33)	0.33(0.33)	1.01	2.60 (0.00; 5.20)	0.33(0.33)	1.01	0.85 (0.00; 1.71)	0.33(0.33)	1.01	Neolithic
D4s2	3	1	1	4.70 (2.33; 7.07)	1.33(0.67)	0.99	7.87 (3.31; 12.44)	1(0.58)	0.99	6.90 (3.93; 9.87)	2.67(1.15)	0.67	Paleolithic
D5a2a1c	5	5	4	4.24 (2.23; 6.25)	1.2(0.57)	0.74	1.57 (0.00; 3.15)	0.2(0.2)	1.00	5.69 (3.70; 7.68)	2.2(0.77)	0.74	Neolithic
D5a2a1d	3	3	3	5.88(2.79;9.01)	1.67(0.88)	0.72	2.63 (0.00; 5.20)	0.33(0.33)	1.01	7.75 (4.65; 10.86)	3(1.2)	0.69	Neolithic
F1b1c	6	4	4	6.47 (4.03; 8.90)	1.83(0.69)	0.64	11.81 (6.69; 16.92)	1.50(0.65)	0.59	8.61 (6.18; 11.04)	3.33(0.94)	0.63	Paleolithic
F1c1a1a	20	22	21	3.53 (2.51; 4.56)	1.00(0.29)	0.59	9.92 (7.24; 12.60)	1.26(0.34)	0.54	5.95 (4.81; 7.08)	2.30(0.44)	0.59	Neolithic
F1c1a1a1	3	3	3	3.53 (0.88; 6.18)	1.00 (0.75)	0.59	10.47 (4.01; 16.92)	1.33(0.82)	0.66	2.59 (0.65; 4.52)	1.00(0.75)	0.59	Neolithic
F1c1a1a2	4	4	4	3.53 (1.77; 5.30)	1.00 (0.50)	1.00	15.74 (9.53; 21.96)	2.00(0.79)	0.80	4.52 (2.59; 6.46)	1.75(0.75)	0.78	Neolithic
F1c1a1a3	3	3	3	0.00 (0.00; 0.00)	0.00 (0.00)	-	0.00 (0.00; 0.00)	0.00(0.00)	-	0.00 (0.00; 0.00)	0.00(0.00)	-	Neolithic
F1d1a2	8	8	8	5.76 (3.32; 8.20)	1.63(0.69)	0.43	22.59 (18.34; 26.84)	2.87(0.54)	1.23	8.07 (5.38; 10.75)	3.12(1.04)	0.36	Paleolithic
F1d1a2a	4	4	4	8.83 (4.17; 13.50)	2.50(1.32)	0.36	11.81 (3.46; 20.15)	1.50(1.06)	0.33	7.11 (-0.10; 14.32)	2.75(2.79)	0.09	Paleolithic
F1d1a2a1	3	3	3	0.00 (0.00; 0.00)	0.00(0.00)	-	0.00 (0.00; 0.00)	0.00(0.00)	-	0.85 (0.00; 1.71)	0.33(0.33)	1.01	Neolithic
F1d2	8	8	7	8.37 (6.18; 10.56)	2.37(0.62)	0.77	9.84 (6.46; 13.22)	1.25(0.43)	0.85	9.69 (7.70; 11.68)	3.75(0.77)	0.79	Paleolithic

	Number of samples for calculation	Number of Tibetan samples (including Sherpa)	Number of Tibetan samples in this study	Coding Region (Soares et al. 2009)			Synonymous Positions (Soares et al. 200			Complete Genome (Soares et al. 2009			Period†
				Age (lower; upper)	ρ (σ)	$\rho/n\sigma^2$	Age (lower; upper)	ρ (σ)	$\rho/n\sigma^2$	Age (lower; upper)	ρ (σ)	$\rho/n\sigma^2$	
F1g	33	26	26	9.12 (7.53; 10.70)	2.58(0.45)	0.39	12.44 (9.68; 15.19)	1.58(0.35)	0.39	8.12 (6.88; 9.36)	3.14(0.48)	0.41	Paleolithic
F1g1	4	3	2	19.43 (14.59; 24.27)	5.5(1.37)	0.73	27.55 (18.74; 36.37)	3.5(1.12)	0.70	17.45 (13.31; 21.58)	6.75(1.6)	0.66	Paleolithic
F1g2	3	3	3	1.17 (0.00; 2.33)	0.33(0.33)	1.01	2.60 (0.00; 5.20)	0.33(0.33)	1.01	0.85 (0.00; 1.71)	0.33(0.33)	1.01	Neolithic
G2a1f2	5	4	4	4.95 (2.40; 7.49)	1.4(0.72)	0.54	9.45 (4.01; 14.88)	1.2(0.69)	0.50	6.20 (3.36; 9.05)	2.4(1.1)	0.40	Paleolithic
G2a1h1	6	6	6	4.13 (2.19; 6.08)	1.17(0.55)	0.64	0.00 (0.00; 0.00)	0(0)	-	6.02 (3.83; 8.22)	2.33(0.85)	0.54	Neolithic
G2a1h1a	3	3	3	4.70 (1.80; 7.60)	1.33(0.82)	0.66	0.00 (0.00; 0.00)	0(0)	-	5.17 (2.74; 7.60)	2(0.94)	0.75	Neolithic
G2b1b	17	17	14	7.70 (5.62; 9.79)	2.18(0.59)	0.37	15.27 (10.47; 20.07)	1.94(0.61)	0.31	8.66 (6.54; 10.78)	3.35(0.82)	0.29	Paleolithic
G2b1b1	7	4	3	8.09 (5.16; 11.02)	2.29(0.83)	0.47	12.36 (6.93; 17.79)	1.57(0.69)	0.47	7.01 (4.76; 9.25)	2.71(0.87)	0.51	Paleolithic
G2b1b2	5	5	5	2.83 (1.41; 4.24)	0.8(0.4)	1.00	3.15 (0.94; 5.35)	0.4(0.28)	1.02	4.65 (2.27; 7.03)	1.8(0.92)	0.43	Neolithic
G2b1b2a	4	4	4	3.53 (1.77; 5.30)	1(0.5)	1.00	3.94 (1.18; 6.69)	0.5(0.35)	1.02	3.23 (1.78; 4.68)	1.25(0.56)	1.00	Neolithic
G3a1	15	14	11	8.23 (4.59; 11.87)	2.33(1.03)	0.15	4.17 (2.05; 6.30)	0.53(0.27)	0.48	8.61 (5.71; 11.50)	3.33(1.12)	0.18	Paleolithic
G3a1a	14	13	11	5.05 (3.39; 6.71)	1.43(0.47)	0.46	3.94 (1.73; 6.14)	0.5(0.28)	0.46	6.28 (4.60; 7.96)	2.43(0.65)	0.41	Neolithic
G3a1a1	4	4	4	4.42 (1.48; 7.35)	1.25(0.83)	0.45	5.90 (0.00; 11.81)	0.75(0.75)	0.33	3.88 (1.63; 6.13)	1.5(0.87)	0.50	Neolithic
G3a1a1a	3	3	3	2.37 (0.71; 4.03)	0.67(0.47)	1.01	0.00 (0.00; 0.00)	0(0)	-	2.59 (1.09; 4.08)	1(0.58)	0.99	Neolithic
G3a1a2	4	4	4	5.30 (2.51; 8.09)	1.5(0.79)	0.60	3.94 (0.00; 7.87)	0.5(0.5)	0.50	3.88 (1.84; 5.92)	1.5(0.79)	0.60	Neolithic
G3b	15	13	11	7.53 (4.80; 10.25)	2.13(0.77)	0.24	12.04 (6.69; 17.40)	1.53(0.68)	0.22	12.07 (9.07; 15.07)	4.67(1.16)	0.23	Paleolithic
G3b1	9	7	6	3.14 (1.59; 4.70)	0.89(0.44)	0.51	7.01 (3.54; 10.47)	0.89(0.44)	0.51	6.02 (4.14; 7.91)	2.33(0.73)	0.49	Paleolithic
G3b1a	4	4	4	5.30 (1.98; 8.62)	1.5(0.94)	0.42	11.81 (4.41; 19.21)	1.5(0.94)	0.42	3.88 (1.45; 6.31)	1.5(0.94)	0.42	Neolithic
G3b1a1	3	3	3	2.37 (0.00; 4.73)	0.67(0.67)	0.50	5.27 (0.00; 10.55)	0.67(0.67)	0.50	1.73 (0.00; 3.46)	0.67(0.67)	0.50	Neolithic
G3b3	5	5	5	4.24 (2.51; 5.97)	1.2(0.49)	1.00	6.30 (3.15; 9.45)	0.8(0.4)	1.00	6.20 (4.01; 8.40)	2.4(0.85)	0.66	Paleolithic
G3b3a	3	3	3	1.17 (0.00; 2.33)	0.33(0.33)	1.01	2.60 (0.00; 5.20)	0.33(0.33)	1.01	3.44 (1.71; 5.17)	1.33(0.67)	0.99	Neolithic
M11a2	10	10	6	12.37 (9.26; 15.47)	3.5(0.88)	0.45	11.81 (6.38; 17.24)	1.5(0.69)	0.32	14.48 (11.63; 17.32)	5.6(1.1)	0.46	Paleolithic
M11a2a	6	6	5	4.70 (3.04; 6.36)	1.33(0.47)	1.00	3.94 (1.65; 6.22)	0.5(0.29)	0.99	8.61 (5.95; 11.27)	3.33(1.03)	0.52	Neolithic
M11a2a1	4	4	3	4.42 (2.44; 6.39)	1.25(0.56)	1.00	1.97 (0.00; 3.94)	0.25(0.25)	1.00	7.11 (4.45; 9.77)	2.75(1.03)	0.65	Neolithic
M11a2a1a	3	3	2	1.17 (0.00; 2.33)	0.33(0.33)	1.01	0.00 (0.00; 0.00)	0(0)	-	0.85 (0.00; 1.71)	0.33(0.33)	1.01	Neolithic
M13a	56	48	40	15.72 (11.80; 19.64)	4.45(1.11)	0.06	15.43 (9.92; 20.94)	1.96(0.7)	0.07	21.56 (17.24; 25.88)	8.34(1.67)	0.05	Paleolithic
M13a1	28	20	17	6.82 (5.48; 8.16)	1.93(0.38)	0.48	8.74 (6.38; 11.10)	1.11(0.3)	0.44	8.76 (6.28; 11.24)	3.39(0.96)	0.13	Paleolithic
M13a1b	24	20	17	6.32 (5.02; 7.63)	1.79(0.37)	0.54	8.50 (6.14; 10.86)	1.08(0.3)	0.50	5.71 (4.65; 6.77)	2.21(0.41)	0.55	Paleolithic
M13a1b1	3	3	0	1.17 (0.00; 2.33)	0.33(0.33)	1.01	2.60 (0.00; 5.20)	0.33(0.33)	1.01	1.73 (0.52; 2.95)	0.67(0.47)	1.01	Neolithic
M13a2	28	28	23	13.46 (8.44; 18.48)	3.81(1.42)	0.07	14.56 (6.77; 22.36)	1.85(0.99)	0.07	13.31 (9.02; 17.60)	5.15(1.66)	0.07	Paleolithic
M13a2a	27	27	22	6.78 (5.30; 8.27)	1.92(0.42)	0.40	7.24 (5.20; 9.29)	0.92(0.26)	0.50	8.38 (5.82; 10.93)	3.24(0.99)	0.12	Paleolithic

	Number of samples for calculation	Number of Tibetan samples (including Sherpa)	Number of Tibetan samples in this study	Coding Region (Soares et al. 2009)			Synonymous Positions (Soares et al. 200[illegible]			Complete Genome (Soares et al. 2009			Period†
				Age (lower; upper)	ρ (σ)	$\rho/n\sigma^2$	Age (lower; upper)	ρ (σ)	$\rho/n\sigma^2$	Age (lower; upper)	ρ (σ)	$\rho/n\sigma^2$	
M13a2a1	23	23	20	7.24 (5.58; 8.90)	2.05(0.47)	0.40	7.87 (5.59; 10.15)	1(0.29)	0.52	6.46 (5.17; 7.76)	2.5(0.5)	0.43	Paleolithic
M13a2a1a	3	3	3	1.17 (0.00; 2.33)	0.33(0.33)	1.01	2.60 (0.00; 5.20)	0.33(0.33)	1.01	1.73 (0.52; 2.95)	0.67(0.47)	1.01	Neolithic
M13a2a1b	3	3	3	2.37 (0.71; 4.03)	0.67(0.47)	1.01	2.60 (0.00; 5.20)	0.33(0.33)	1.01	2.59 (1.09; 4.08)	1(0.58)	0.99	Neolithic
M13a2a1c	3	3	2	7.07 (3.74; 10.39)	2(0.94)	0.75	0.00 (0.00; 0.00)	0(0)	-	6.02 (3.44; 8.61)	2.33(1)	0.78	Paleolithic
M13a2a1d	3	3	3	5.90 (2.37; 9.43)	1.67(1)	0.56	13.15 (5.27; 21.02)	1.67(1)	0.56	4.32 (1.73; 6.90)	1.67(1)	0.56	Neolithic
M13a2a1e	4	4	3	2.83 (1.41; 4.24)	0.8(0.4)	1.25	4.72 (1.97; 7.48)	0.6(0.35)	1.22	3.10 (1.84; 4.37)	1.2(0.49)	1.25	Neolithic
M62	42	38	23	13.71 (10.70; 16.71)	3.88(0.85)	0.13	20.07 (14.72; 25.43)	2.55(0.68)	0.13	23.45 (19.31; 27.58)	9.07(1.6)	0.08	Paleolithic
M62a	9	7	6	5.51 (3.60; 7.42)	1.56(0.54)	0.59	5.27 (2.52; 8.03)	0.67(0.35)	0.61	12.93 (9.38; 16.47)	5(1.37)	0.30	Neolithic
M62a1	7	7	6	2.51 (0.99; 4.03)	0.71(0.43)	0.55	0.00 (0.00; 0.00)	0(0)	-	10.70 (7.26; 14.14)	4.14(1.33)	0.33	Neolithic
M62a1a	6	6	5	2.93 (1.17; 4.70)	0.83(0.5)	0.55	0.00 (0.00; 0.00)	0(0)	-	9.49 (6.44; 12.54)	3.67(1.18)	0.44	Neolithic
M62a1a1	4	4	3	4.42 (1.77; 7.07)	1.25(0.75)	0.56	0.00 (0.00; 0.00)	0(0)	-	6.46 (3.72; 9.20)	2.5(1.06)	0.56	Neolithic
M62b	33	31	17	14.03 (10.49; 17.56)	3.97(1)	0.12	19.84 (13.78; 25.90)	2.52(0.77)	0.13	20.91 (16.65; 25.18)	8.09(1.65)	0.09	Paleolithic
M62b1	23	21	13	14.45 (9.75; 19.15)	4.09(1.33)	0.10	18.11 (10.55; 25.66)	2.3(0.96)	0.11	16.62 (12.74; 20.50)	6.43(1.5)	0.12	Paleolithic
M62b1a	15	13	4	7.77 (4.49; 11.06)	2.2(0.93)	0.17	8.42 (6.22; 10.63)	1.07(0.28)	0.91	12.74 (9.18; 16.31)	4.93(1.38)	0.17	Paleolithic
M62b1a1	13	12	4	4.35 (3.18; 5.51)	1.23(0.33)	0.87	8.50 (6.06; 10.94)	1.08(0.31)	0.86	7.37 (5.69; 9.05)	2.85(0.65)	0.52	Paleolithic
M62b1a1a	3	3	1	9.43 (5.72; 13.14)	2.67(1.05)	0.81	15.74 (8.34; 23.14)	2(0.94)	0.75	7.76 (4.89; 10.62)	3(1.11)	0.81	Paleolithic
M62b1a1b	8	7	3	2.83 (1.84; 3.82)	0.8(0.28)	1.28	6.30 (4.09; 8.50)	0.8(0.28)	1.28	6.46 (4.63; 8.30)	2.5(0.71)	0.62	Paleolithic
M62b1a1b1	3	3	0	2.83 (1.41; 4.24)	0.8(0.4)	1.67	6.30 (3.15; 9.45)	0.8(0.4)	1.67	6.20 (4.14; 8.27)	2.4(0.8)	1.25	Paleolithic
M62b1b	6	6	6	4.13 (1.45; 6.82)	1.17(0.76)	0.34	6.53 (1.10; 11.97)	0.83(0.69)	0.29	6.90 (4.03; 9.77)	2.67(1.11)	0.36	Paleolithic
M62b1b1	4	4	4	0.88 (0.00; 1.77)	0.25(0.25)	1.00	1.97 (0.00; 3.94)	0.25(0.25)	1.00	5.17 (2.27; 8.07)	2(1.12)	0.40	Neolithic
M62b2	8	8	5	7.49 (4.80; 10.18)	2.12(0.76)	0.46	9.84 (4.49; 15.19)	1.25(0.68)	0.34	12.93 (9.85; 16.00)	5(1.19)	0.44	Paleolithic
M62b2a	5	5	3	7.07 (4.84; 9.29)	2(0.63)	1.01	7.87 (4.33; 11.41)	1(0.45)	0.99	10.34 (7.60; 13.08)	4(1.06)	0.71	Paleolithic
M9a1a1c1b1a‡	154	138	111	4.24 (2.40; 6.08)	1.20 (0.52)	0.03	4.96 (4.45; 5.48)	1.92 (0.20)	0.31	5.11 (4.36; 5.86)	0.64 (0.0.0[illegible]	-	Neolithic
M9a1a1c1b1a1	5	4	1	4.24 (2.26; 6.22)	1.20(0.56)	0.77	4.72 (1.97; 7.48)	0.60(0.35)	0.98	6.20 (4.14; 8.27)	2.40(0.80)	0.75	Neolithic
M9a1a1c1b1a10	3	0	0	0.00 (0.00; 0.00)	0.00(0.00)	-	0.00 (0.00; 0.00)	0.00(0.00)	-	1.73 (0.00; 3.46)	0.67(0.67)	0.50	Neolithic
M9a1a1c1b1a11	4	4	3	3.53 (1.77; 5.30)	1.00(0.50)	1.00	1.97 (0.00; 3.94)	0.25(0.25)	1.00	3.23 (1.78; 4.68)	1.25(0.56)	1.00	Neolithic
M9a1a1c1b1a12	5	5	4	3.53 (1.66; 5.41)	1.00(0.53)	0.71	6.30 (2.44; 10.15)	0.80(0.49)	0.67	3.10 (1.63; 4.58)	1.20(0.57)	0.74	Neolithic
M9a1a1c1b1a13	7	7	6	4.52 (2.86; 6.18)	1.28(0.47)	0.83	5.98 (3.23; 8.74)	0.76(0.35)	0.89	7.01 (4.96; 9.05)	2.71(0.79)	0.62	Neolithic
M9a1a1c1b1a14	3	3	2	1.17 (0.00; 2.33)	0.33(0.33)	1.01	0.00 (0.00; 0.00)	0.00(0.00)	-	3.44 (1.32; 5.56)	1.33(0.82)	0.66	Neolithic
M9a1a1c1b1a2a	8	9	8	2.65 (1.55; 3.74)	0.75(0.31)	0.98	3.94 (1.97; 5.90)	0.50(0.25)	1.00	3.23 (2.02; 4.45)	1.25(0.47)	0.71	Neolithic
M9a1a1c1b1a3	3	1	2	1.17 (0.00; 2.33)	0.33(0.33)	1.01	2.60 (0.00; 5.20)	0.33(0.33)	1.01	0.85 (0.00; 1.71)	0.33(0.33)	1.01	Neolithic

	**Number of samples for calculation**	**Number of Tibetan samples (including Sherpa)**	**Number of Tibetan samples in this study**	**Coding Region (Soares et al. 2009)**			**Synonymous Positions (Soares et al. 200**			**Complete Genome (Soares et al. 2009**			Period†
				**Age (lower; upper)**	**ρ (σ)**	**$\rho/n\sigma^2$**	**Age (lower; upper)**	**ρ (σ)**	**$\rho/n\sigma^2$**	**Age (lower; upper)**	**ρ (σ)**	**$\rho/n\sigma^2$**	
M9a1a1c1b1a4	3	0	3	0.00 (0.00; 0.00)	0.00(0.00)	-	0.00 (0.00; 0.00)	0.00(0.00)	-	0.00 (0.00; 0.00)	0.00(0.00)	-	Neolithic
M9a1a1c1b1a5	3	1	2	3.53 (0.88; 6.18)	1.00(0.75)	0.59	5.27 (0.00; 10.55)	0.67(0.67)	0.50	4.32 (2.04; 6.59)	1.67(0.88)	0.72	Neolithic
M9a1a1c1b1a6	3	3	3	4.70 (1.38; 8.02)	1.33(0.94)	0.50	5.27 (0.00; 10.55)	0.67(0.67)	0.50	3.44 (1.01; 5.87)	1.33(0.94)	0.50	Neolithic
M9a1a1c1b1a7	3	3	3	2.37 (0.71; 4.03)	0.67(0.47)	1.01	2.60 (0.00; 5.20)	0.33(0.33)	1.01	3.44 (1.71; 5.17)	1.33(0.67)	0.99	Neolithic
M9a1a1c1b1a8	8	7	4	4.84 (3.39; 6.29)	1.37(0.41)	1.02	5.90 (3.46; 8.34)	0.75(0.31)	0.98	4.19 (3.02; 5.35)	1.62(0.45)	1.00	Neolithic
M9a1a1c1b1a9	4	2	0	0.00 (0.00; 0.00)	0.00(0.00)	-	0.00 (0.00; 0.00)	0.00(0.00)	-	0.65 (0.00; 1.29)	0.25(0.25)	1.00	Neolithic
M9a1a2	18	16	5	3.53 (1.91; 5.16)	1(0.46)	0.26	5.27 (2.36; 8.19)	0.67(0.37)	0.27	9.05 (5.84; 12.25)	3.5(1.24)	0.13	Neolithic
M9a1a2a	17	14	5	3.32 (1.59; 5.05)	0.94(0.49)	0.23	5.12 (2.05; 8.19)	0.65(0.39)	0.25	6.70 (4.52; 8.87)	2.59(0.84)	0.22	Neolithic
M9a1a2a1	10	8	5	3.53 (1.24; 5.83)	1(0.65)	0.24	7.87 (2.76; 12.99)	1(0.65)	0.24	6.20 (3.98; 8.43)	2.4(0.86)	0.32	Neolithic
M9a1a2a1a	6	4	3	2.37 (0.92; 3.82)	0.67(0.41)	0.66	5.27 (2.05; 8.50)	0.67(0.41)	0.66	3.44 (2.07; 4.81)	1.33(0.53)	0.79	Neolithic
M9a1a2a1b	4	4	2	0.00 (0.00; 0.00)	0(0)	-	0.00 (0.00; 0.00)	0(0)	-	3.88 (1.63; 6.13)	1.5(0.87)	0.50	Neolithic
M9a1a2a1b1	3	3	1	0.00 (0.00; 0.00)	0(0)	-	0.00 (0.00; 0.00)	0(0)	-	1.73 (0.52; 2.95)	0.67(0.47)	1.01	Neolithic
M9a1a2a2	5	5	0	0.00 (0.00; 0.00)	0(0)	-	0.00 (0.00; 0.00)	0(0)	-	1.55 (0.39; 2.71)	0.6(0.45)	0.59	Neolithic
M9a1b1c	18	17	14	3.14 (2.26; 4.03)	0.89(0.25)	0.79	3.94 (2.52; 5.35)	0.50(0.18)	0.86	5.45 (4.37; 6.54)	2.11(0.42)	0.66	Neolithic
M9a1b1e	16	8	7	3.74 (2.79; 4.70)	1.06(0.27)	0.91	5.43 (3.62; 7.24)	0.69(0.23)	0.82	5.51 (4.16; 6.85)	2.13(0.52)	0.49	Neolithic
M9a1b1e1	5	4	4	4.24 (2.23; 6.25)	1.20(0.57)	0.74	9.45 (5.04; 13.85)	1.20(0.56)	0.77	3.10 (1.63; 4.58)	1.20(0.57)	0.74	Neolithic
M9a1b1d	7	6	6	2.51 (1.17; 3.85)	0.71(0.38)	0.70	2.28 (0.71; 3.86)	0.29(0.2)	1.04	2.22 (1.19; 3.26)	0.86(0.4)	0.77	Neolithic
Z3a3	4	4	4	9.72 (5.86; 13.57)	2.75(1.09)	0.58	9.84 (4.64; 15.04)	1.25(0.66)	0.72	13.57 (9.64; 17.50)	5.25(1.52)	0.57	Paleolithic
Z7a	3	3	2	4.70 (1.80; 7.60)	1.33(0.82)	0.66	2.60 (0.00; 5.20)	0.33(0.33)	1.01	6.90 (4.19; 9.62)	2.67(1.05)	0.81	Neolithic

Note: †The period of a haplogroup was determined if no less than two estimations were within this period.

‡ Haplogroups with ages from 5.2-4.0 kya

Table S9. Estimated proportions of different components in Tibetans since the Neolithic period.

	All Tibetans	A11a1a and M9a1a1c1b1a		Paleolithic lineages		Other Neolithic lineages	
Time (ka)	Effective population size (95% CI)	Effective population size (95% CI)	Proportions (%) (95% CI)	Effective population size (95% CI)	Proportions (%) (95% CI)	Effective population size (95% CI)	Proportions (%) (95% CI)
0.00	3452.10 (857.07-23076.84)	658.77 (127.35-4022.63)	19.08 (14.86-17.43)	1100.54 (216.81-9568.68)	31.88 (25.30-41.46)	686.09 (137.87-4288.62)	19.87 (16.09-18.58)
0.30	3279.65 (1174.51-19330.44)	723.57 (329.15-2411.54)	22.06 (28.02-12.48)	992.10 (381.92-5949.59)	30.25 (32.52-30.78)	675.60 (266.55-3165.78)	20.60 (22.69-16.38)
0.60	3060.01 (1242.33-15982.69)	778.15 (380.12-1917.62)	25.43 (30.60-12.00)	891.04 (374.49-3955.74)	29.12 (30.14-24.75)	668.19 (286.55-2450.72)	21.84 (23.07-15.33)
0.91	2872.22 (1206.36-13126.00)	821.77 (406.78-1810.85)	28.61 (33.72-13.80)	803.31 (320.08-2802.71)	27.97 (26.53-21.35)	651.39 (276.84-1966.34)	22.68 (22.95-14.98)
1.21	2672.07 (1154.17-10992.12)	854.82 (420.56-1833.28)	31.99 (36.44-16.68)	727.00 (277.84-2166.31)	27.21 (24.07-19.71)	625.46 (253.73-1756.94)	23.41 (21.98-15.98)
1.51	2484.53 (1046.98-9269.08)	880.98 (425.55-1865.96)	35.46 (40.65-20.13)	662.94 (237.50-1771.12)	26.68 (22.68-19.11)	592.89 (228.66-1567.90)	23.86 (21.84-16.92)
1.81	2290.48 (914.92-7719.55)	890.54 (409.22-1955.46)	38.88 (44.73-25.33)	603.27 (203.98-1567.74)	26.34 (22.29-20.31)	548.76 (189.31-1450.27)	23.96 (20.69-18.79)
2.12	2116.08 (781.28-6569.08)	887.08 (389.47-1987.02)	41.92 (49.85-30.25)	560.49 (178.82-1426.75)	26.49 (22.89-21.72)	496.59 (152.66-1357.36)	23.47 (19.54-20.66)
2.42	1950.34 (680.52-5639.98)	866.47 (349.50-2047.05)	44.43 (51.36-36.30)	528.37 (176.45-1330.37)	27.09 (25.93-23.59)	432.26 (106.99-1275.81)	22.16 (15.72-22.62)
2.72	1793.75 (607.32-4926.16)	835.07 (318.49-2102.90)	46.55 (52.44-42.69)	504.52 (176.33-1276.01)	28.13 (29.03-25.90)	363.76 (70.06-1216.14)	20.28 (11.54-24.69)
3.02	1641.83 (524.15-4461.01)	785.56 (269.19-2054.81)	47.85 (51.36-46.06)	485.98 (181.71-1205.97)	29.60 (34.67-27.03)	284.48 (40.38-1110.33)	17.33 (7.70-24.89)
3.32	1478.85 (441.76-4098.12)	721.85 (223.86-2039.35)	48.81 (50.68-49.76)	470.81 (187.54-1188.45)	31.84 (42.45-29.00)	202.90 (25.90-959.03)	13.72 (5.86-23.40)
3.63	1336.03 (365.04-3880.76)	636.34 (167.32-1863.34)	47.63 (45.84-48.01)	457.64 (181.59-1171.88)	34.25 (49.74-30.20)	132.48 (20.04-805.88)	9.92 (5.49-20.77)
3.93	1184.79 (322.60-3543.61)	536.85 (102.82-1754.30)	45.31 (31.87-49.51)	443.31 (173.02-1157.89)	37.42 (53.63-32.68)	82.70 (17.29-634.51)	6.98 (5.36-17.91)
4.23	1038.46 (285.20-3252.44)	416.05 (48.33-1585.25)	40.06 (16.95-48.74)	429.22 (165.91-1142.73)	41.33 (58.17-35.13)	60.85 (16.39-469.09)	5.86 (5.75-14.42)
4.53	902.31 (249.56-3019.70)	296.44 (19.13-1390.93)	32.85 (7.66-46.06)	415.41 (157.24-1125.01)	46.04 (63.01-37.26)	52.92 (16.33-342.76)	5.87 (6.54-11.35)
4.84	779.92 (209.33-2736.11)	182.78 (11.17-1123.84)	23.44 (5.34-41.07)	399.16 (149.63-1104.61)	51.18 (71.48-40.37)	50.37 (16.10-239.71)	6.46 (7.69-8.76)
5.14	672.17 (183.12-2401.86)	96.70 (7.96-858.28)	14.39 (4.35-35.73)	381.06 (140.37-1089.33)	56.69 (76.65-45.35)	50.40 (18.28-165.03)	7.50 (9.98-6.87)
5.44	591.40 (167.59-2127.73)	47.15 (6.21-634.47)	7.97 (3.71-29.82)	363.35 (132.85-1061.06)	61.44 (79.27-49.87)	51.60 (19.03-133.38)	8.72 (11.36-6.27)
5.74	530.01 (150.92-1864.11)	26.14 (5.41-456.49)	4.93 (3.59-24.49)	342.01 (121.14-1015.45)	64.53 (80.26-54.47)	53.98 (20.71-122.80)	10.19 (13.72-6.59)
6.04	496.95 (150.84-1622.32)	18.64 (4.71-291.55)	3.75 (3.12-17.97)	323.10 (115.87-985.07)	65.02 (76.82-60.72)	56.76 (22.87-121.99)	11.42 (15.16-7.52)
6.35	471.10 (154.82-1409.94)	15.22 (4.49-173.84)	3.23 (2.90-12.33)	305.27 (105.11-917.79)	64.80 (67.89-65.09)	60.09 (25.56-124.77)	12.75 (16.51-8.85)

Table S10. Complete mtDNA variants and haplogroup allocations of 682 samples in this study.

Sample ID	Population code	Location	Haplogroup	Variants
K38137	Tib_1	Ngari	D4b2b	73, 194, 263, 310, 489, 523-524d, 750, 1382C, 1438, 2706, 3010, 3106N, 3397, 4113, 4769, 4883, 5178A, 7028, 8020, 8414, 8701, 8860, 8964, 9296, 9540, 9824A, 10398, 10400, 10873, 11719, 12705, 14053, 14668, 14766, 14783, 15043, 15301, 15326, 16223, 16291, 16356, 16362, 16519
K38101	Tib_1	Ngari	A15a	73, 152, 204, 207, 235, 309+C, 315+C, 523-524d, 663, 750, 1438, 1736, 2706, 3106N, 4248, 4769, 4824, 7028, 8459, 8794, 8860, 11084, 11719, 12705, 14067, 14766, 15326, 16223, 16274, 16290, 16319, 16362
K38414	Tib_1	Ngari	D4q	73, 195, 200, 263, 309+C, 315+C, 489, 750, 1438, 2706, 3010, 3106N, 4769, 4883, 5178A, 7028, 8414, 8701, 8860, 9540, 10398, 10400, 10873, 11719, 12705, 14311, 14668, 14766, 14783, 15043, 15301, 15326, 16223, 16256, 16311, 16362, 16519
K38013	Tib_1	Ngari	M9a1a1c1b1a	73, 263, 309+CC, 315+C, 489, 711, 750, 1041, 1438, 2706, 3106N, 3394, 4491, 4769, 5899+XC, 7028, 7142, 7697, 8701, 8860, 9242, 9540, 10398, 10400, 10873, 11719, 12705, 14308, 14417, 14766, 14783, 15043, 15301, 15326, 16223, 16316, 16362
K38066	Tib_1	Ngari	M9a1a1c1b1a	73, 263, 309+C, 315+C, 489, 711, 750, 1041, 1438, 2706, 3106N, 3394, 4491, 4769, 5899+XC, 7028, 7142, 7697, 7870, 8701, 8860, 9242, 9540, 10398, 10400, 10873, 11719, 12705, 14308, 14417, 14766, 14783, 15043, 15301, 15326, 16223, 16234, 16244, 16316, 16362
K38842	Tib_1	Ngari	F1b1c	73, 152, 249d, 263, 309+CC, 315+C, 523-524d, 750, 1438, 2706, 3106N, 3345, 3970, 4732, 4769, 5147, 5508, 6392, 6962, 7028, 8860, 10235, 10310, 10609, 10976, 11719, 11732, 12406, 12882, 13095, 13928C, 14476, 14766, 15119, 15326, 16134, 16183C, 16189, 16232A, 16249, 16304, 16519
K37576	Tib_1	Ngari	M7b1a1b	73, 150, 152, 199, 204, 207, 263, 271, 309+CC, 315+C, 489, 750, 1438, 2706, 3106N, 3483, 4048, 4071, 4164, 4769, 5351, 5460, 6455, 6680, 7028, 7684, 7853, 8701, 8860, 9540, 9824, 10398, 10400, 10873, 11719, 12405, 12705, 12811, 13105, 14766, 14783, 15043, 15301, 15326, 16223, 16297
K38581	Tib_1	Ngari	G2a1i	73, 263, 310, 489, 709, 750, 1438, 2706, 3106N, 4769, 4833, 5108, 5601, 6266, 7028, 7600, 8701, 8860, 9377, 9540, 9575, 10398, 10400, 10873, 11719, 12705, 13563, 14200, 14569, 14766, 14783, 15043, 15301, 15326, 16129, 16223, 16278, 16362
K38844	Tib_1	Ngari	M9a1a1c1b1a	73, 263, 309+C, 315+C, 489, 711, 750, 1041, 1438, 2706, 3106N, 3394, 4491, 4769, 5899+XC, 7028, 7142, 7697, 8701, 8860, 9242, 9540, 10398, 10400, 10873, 11719, 12705, 14308, 14417, 14766, 14783, 15043, 15301, 15326, 16114G, 16223, 16234, 16316, 16362
K38724	Tib_1	Ngari	M9a1a1c1b1a8	73, 263, 309+CC, 315+C, 489, 711, 750, 1041, 1438, 2706, 3106N, 3394, 4491, 4769, 7028, 7142, 7697, 8701, 8860, 9242, 9540, 10398, 10400, 10873, 11719, 12705, 14308, 14417, 14766, 14783, 15043, 15301, 15326, 16223, 16234, 16316, 16362
K38671	Tib_1	Ngari	M9a1a1c1b1a	73, 263, 309+CC, 315+C, 489, 711, 750, 1041, 1438, 2706, 3106N, 3394, 4491, 4769, 5899+XC, 7028, 7142, 7697, 8701, 8860, 9242, 9540, 10398, 10400, 10873, 11719, 12705, 14185, 14308, 14417, 14766, 14783, 15043, 15301, 15326, 16223, 16234, 16316, 16362
K38418	Tib_1	Ngari	D4j3b	73, 263, 309+C, 315+C, 338, 489, 750, 1438, 2706, 3010, 3106N, 4769, 4883, 5178A, 7028, 8414, 8701, 8860, 9540, 10398, 10400, 10873, 11696, 11719, 12164, 12705, 14668, 14766, 14783, 15043, 15301, 15326, 15519, 16184, 16223, 16260, 16311, 16362, 16519
K38754	Tib_1	Ngari	U7a3b	73, 151, 152, 263, 310, 523-524d, 750, 980, 1438, 1453, 1811, 2706, 3106N, 3741, 3834, 4769, 5360, 6452, 7028, 8137, 8684, 8860, 10142, 10899, 11467, 11719, 12308, 12372, 12618, 13135, 13500, 14569, 14766, 15326, 16140, 16207, 16309, 16318T, 16362, 16519
K38733	Tib_1	Ngari	F1d	73, 146, 249d, 263, 309+CC, 315+C, 523-524d, 750, 1438, 1734, 2706, 3106N, 3970, 4769, 5460, 5628, 5794, 6392, 6962, 7028, 7738, 8410, 8860, 10310, 10609, 11092, 11719, 12406, 12882, 13928C, 14766, 15326, 15402, 16304, 16519
K38506	Tib_1	Ngari	A11a1a1	73, 152, 235, 263, 309+CC, 315+C, 460, 523-524d, 663, 750, 1005, 1438, 1736, 2706, 3106N, 4248, 4769, 4824, 5162, 5899+XC, 6755, 7028, 8794, 8843, 8860, 9650, 11719, 12705, 14766, 15326, 15930, 16212, 16223, 16290, 16293C, 16319, 16519
K38501	Tib_1	Ngari	Z3a1a	73, 146, 152, 207, 249d, 263, 309+C, 315+C, 489, 750, 1438, 2706, 3106N, 4715, 4769, 5806, 6752, 7028, 7196A, 8584, 8701, 8860, 8931, 9090, 9540, 9713, 10208, 10398, 10400, 10873, 11075, 11719, 12705, 13620, 14766, 14783, 15043, 15301, 15487T, 15784, 15928, 16185, 16223, 16260, 16298
K38180	Tib_2	Chamdo	B4d4	73, 188, 263, 309+CC, 315+C, 750, 827, 1438, 2706, 2755, 3106N, 4769, 5372, 6324C, 7028, 8281-8289d, 8860, 11719, 11914, 13942, 14766, 15326, 15535, 15930, 16183C, 16189, 16217, 16234, 16519
K38117	Tib_2	Chamdo	G2a1h1a	73, 146, 263, 283, 309+C, 315+C, 489, 709, 750, 1438, 2706, 3106N, 3203, 3316, 4158, 4769, 4833, 5108, 5601, 7028, 7600, 8701, 8860, 9377, 9540, 9575, 10398, 10400, 10873, 11719, 12705, 13194, 13563, 14200, 14569, 14766, 14783, 15043, 15301, 15326, 16092, 16215, 16227, 16278, 16362
K38845	Tib_2	Chamdo	A	73, 152, 182, 235, 263, 309+C, 315+C, 663, 750, 1438, 1736, 2706, 3106N, 4248, 4769, 4824, 7028, 8794, 8860, 8908, 9038, 11719, 12705, 13488, 14766, 15326, 16223, 16248, 16290, 16319, 16362

K38194	Tib_2	Chamdo	M9a1b1d	73, 150, 152, 153, 263, 310, 489, 750, 1041, 1438, 2706, 3106N, 3394, 4491, 4769, 7028, 8701, 8860, 9540, 10398, 10400, 10604, 10873, 11719, 12362, 12705, 14308, 14766, 14783, 15043, 15119, 15301, 15326, 15671, 16158, 16223, 16234, 16362, 16519
K3162	Tib_2	Chamdo	M9a1a1c1b1a11	73, 263, 309+C, 315+C, 489, 711, 750, 1041, 1438, 2706, 3106N, 3394, 4491, 4769, 5492, 5899+XC, 7028, 7142, 7697, 8701, 8860, 9242, 9540, 10398, 10400, 10873, 11719, 12705, 13602, 14308, 14417, 14766, 14783, 15043, 15301, 15326, 16223, 16234, 16316, 16362, 16519
K3163	Tib_2	Chamdo	M8a1	73, 263, 310, 489, 750, 1438, 2706, 3106N, 4314d, 4715, 4769, 6179, 7028, 7196A, 8453, 8584, 8684, 8701, 8860, 9540, 10398, 10400, 10873, 11719, 12705, 14470, 14766, 14783, 15043, 15047, 15301, 15326, 15487T, 15697, 15787, 16223, 16298, 16319
K38220	Tib_2	Chamdo	A17a2	73, 152, 235, 263, 309+C, 315+C, 523-524d, 538, 663, 750, 1438, 1736, 2218, 2706, 3106N, 4113, 4248, 4769, 4824, 5514, 7028, 8794, 8860, 9126, 11719, 12705, 14766, 15217, 15326, 16086, 16223, 16290, 16319, 16362
K38635	Tib_2	Chamdo	B4d1'2'3	73, 263, 309+C, 315+C, 750, 827, 1438, 2625, 2706, 3106N, 4769, 7028, 8281-8289d, 8860, 11719, 11914, 13942, 14766, 15326, 15535, 15930, 16183C, 16189, 16217, 16234, 16519
K38176	Tib_2	Chamdo	B5b	73, 103, 263, 309+CC, 315+C, 523-524d, 709, 750, 960+XC, 1438, 1598, 2706, 3106N, 4418, 4769, 4947, 6101, 7028, 8281-8289d, 8584, 8784, 8829, 8860, 9950, 10398, 11719, 12361, 14384, 14766, 15077, 15223, 15326, 15508, 15662, 15791C, 15851, 15927, 16140, 16182C, 16183C, 16189, 16243
K38679	Tib_2	Chamdo	D4b2b10	41, 73, 194, 207, 263, 310, 489, 523-524d, 750, 1382C, 1438, 2706, 3010, 3106N, 4769, 4883, 5178A, 7028, 8020, 8414, 8701, 8860, 8964, 9296, 9540, 9824A, 10398, 10400, 10873, 11719, 12705, 14668, 14766, 14783, 15043, 15301, 15326, 16223, 16362, 16519
K38130	Tib_2	Chamdo	D4b2b8	73, 183, 194, 263, 310, 390, 489, 523-524d, 750, 1382C, 1438, 2706, 3010, 3106N, 3398, 4769, 4883, 5178A, 7028, 8020, 8414, 8701, 8860, 8964, 9296, 9540, 9824A, 10398, 10400, 10873, 11719, 12705, 13500, 14668, 14766, 14783, 15043, 15301, 15326, 15613, 16223, 16362, 16519
K38195	Tib_2	Chamdo	D4j3b	73, 263, 309+CC, 315+C, 338, 489, 750, 1438, 2706, 3010, 3106N, 4769, 4883, 5178A, 7028, 8414, 8701, 8860, 9540, 10398, 10400, 10873, 11696, 11719, 12705, 14668, 14766, 14783, 15043, 15301, 15326, 16184, 16223, 16311, 16362
K38065	Tib_2	Chamdo	F1b1c	73, 152, 249d, 263, 309+CC, 315+C, 523-524d, 750, 1438, 2706, 3106N, 3970, 4732, 4769, 5147, 5508, 6392, 6962, 7028, 8860, 8955, 9932, 10235, 10310, 10609, 10976, 11719, 12406, 12882, 13928C, 14476, 14766, 15119, 15326, 16182C, 16183C, 16189, 16209, 16232A, 16249, 16304, 16519
K38419	Tib_2	Chamdo	F1g	73, 249d, 263, 310, 523-524d, 750, 1438, 2389, 2706, 3106N, 3398, 3970, 4769, 6392, 6962, 7028, 8837, 8860, 10310, 10609, 11719, 12406, 12882, 13928C, 14766, 15326, 16183C, 16189, 16304, 16519
K38576	Tib_2	Chamdo	G1a	73, 263, 310, 489, 709, 750, 1438, 2706, 3106N, 4769, 4833, 5108, 6719, 7028, 7867, 8200, 8289+CCCCCTCTA, 8701, 8860, 9540, 10398, 10400, 10873, 11719, 12070, 12705, 14569, 14766, 14783, 15043, 15301, 15323, 15326, 15497, 16223, 16311, 16362, 16519
K38155	Tib_2	Chamdo	G2a1h1a	73, 146, 263, 283, 309+C, 315+C, 489, 709, 750, 1438, 2706, 3106N, 4158, 4769, 4833, 5108, 5601, 7028, 7600, 8701, 8860, 9377, 9540, 9575, 10398, 10400, 10873, 11084, 11719, 12705, 13194, 13563, 14200, 14569, 14766, 14783, 15043, 15301, 15326, 16227, 16278, 16362
K38170	Tib_2	Chamdo	G3a1	16T, 73, 143, 150, 263, 310, 489, 709, 750, 1438, 2706, 3106N, 4769, 4833, 5108, 7028, 8701, 8860, 9540, 10398, 10400, 10873, 11719, 11914, 12705, 14569, 14766, 14783, 15043, 15301, 15326, 15746, 16215, 16223, 16225, 16274
K38848	Tib_2	Chamdo	M13a1b	73, 152, 263, 309+C, 315+C, 489, 750, 1438, 2706, 3106N, 3644, 4769, 5773, 6023, 6253, 6620, 7028, 8701, 8860, 9540, 10398, 10400, 10411, 10790, 10873, 11719, 12705, 13135, 14766, 14783, 15043, 15301, 15326, 15924, 16145, 16148, 16188, 16189, 16223, 16381
K38898	Tib_2	Chamdo	M9a1a1c1b1a4	73, 263, 310, 489, 711, 750, 1041, 1438, 2706, 3106N, 3394, 4491, 4769, 5899+XC, 7028, 7142, 7697, 8701, 8860, 9242, 9540, 10398, 10400, 10873, 11719, 12705, 14308, 14417, 14766, 14783, 15043, 15301, 15326, 15470, 16223, 16234, 16316, 16362, 16519
K38039	Tib_2	Chamdo	A	73, 152, 182, 235, 263, 309+C, 315+C, 663, 750, 1438, 1736, 2706, 3106N, 4248, 4769, 4824, 7028, 8794, 8860, 11719, 12705, 13488, 14766, 15326, 16223, 16248, 16290, 16319, 16362
K38095	Tib_2	Chamdo	C4a1a1a1	73, 249d, 263, 310, 489, 523-524d, 750, 1438, 1715, 2232+AA, 2706, 3106N, 3552A, 3576, 4715, 4769, 4884, 4958, 6026, 7028, 7196A, 7999, 8508, 8584, 8701, 8860, 9540, 9545, 10398, 10400, 10873, 11719, 11914, 11969, 12672, 12705, 13263, 14318, 14766, 14783, 15043, 15204, 15301, 15326, 15487T, 15968, 16093, 16129, 16223, 16224, 16298, 16327, 16519
K38182	Tib_2	Chamdo	D4b2b9	73, 194, 263, 310, 489, 523-524d, 750, 1382C, 1438, 2706, 3010, 3106N, 4769, 4883, 5178A, 7028, 8020, 8414, 8545, 8701, 8860, 8964, 9296, 9540, 9824A, 10398, 10400, 10873, 11719, 12705, 14668, 14751, 14766, 14783, 15043, 15301, 15326, 16111, 16223, 16362, 16519
K38183	Tib_2	Chamdo	F1g	73, 249d, 263, 309+CC, 315+C, 523-524d, 750, 1438, 2389, 2706, 3106N, 3398, 3970, 4769, 6260, 6392, 6962, 7028, 8860, 10310, 10609, 11719, 12406, 12882, 13928C, 14766, 15326, 16183C, 16189, 16193+CC, 16304, 16519

K38249	Tib_2	Chamdo	G3a1a	16T, 73, 150, 152, 263, 309+C, 315+C, 489, 709, 750, 1438, 2706, 3106N, 4769, 4833, 5108, 7028, 8701, 8860, 8861, 9540, 10398, 10400, 10873, 11719, 11914, 12705, 13279, 14569, 14766, 14783, 15043, 15301, 15326, 15746, 16215, 16223, 16274
K38986	Tib_2	Chamdo	M11a2	73, 146, 198, 200, 215, 263, 309+C, 315+C, 318, 326, 489, 750, 1095, 1438, 2706, 2850, 3106N, 4769, 6531, 7028, 7642, 8108, 8701, 8860, 9540, 9950, 10398, 10400, 10873, 11404, 11719, 11969, 12705, 13074, 13563, 14340, 14766, 14783, 15043, 15301, 15326, 16173, 16223, 16301
K38097	Tib_2	Chamdo	M9a1a1c1b1a	73, 143, 263, 309+C, 315+C, 489, 711, 750, 1041, 1438, 2706, 3106N, 3394, 4491, 4769, 5894C, 7028, 7142, 7697, 8425, 8701, 8860, 9242, 9540, 10398, 10400, 10873, 11719, 12681, 12705, 14308, 14417, 14766, 14783, 15043, 15301, 15326, 16223, 16234, 16362
K37106	Tib_2	Chamdo	N9a1	73, 150, 263, 309+CC, 315+C, 750, 1438, 2706, 3106N, 3846A, 4386, 4769, 5231, 5417, 6366, 7028, 7424, 7711, 8860, 11279, 11719, 12007, 12358, 12372, 12705, 14766, 15326, 16111, 16129, 16223, 16257A, 16261
K38363	Tib_2	Chamdo	A11b	73, 152, 235, 263, 309+C, 315+C, 523-524d, 663, 750, 1438, 1736, 2706, 3106N, 3290, 4248, 4769, 4824, 7028, 8794, 8860, 9338, 9650, 11719, 12705, 14766, 15326, 16223, 16234, 16290, 16293C, 16319, 16357, 16519, 16527
K38226	Tib_2	Chamdo	B4h1	73, 195, 263, 309d, 315+C, 750, 1438, 2706, 3106N, 3398, 4703, 4769, 5093, 7028, 7080, 8158, 8281-8289d, 8790, 8860, 11719, 13269, 13710, 14766, 15326, 16129, 16189, 16261
K38272	Tib_2	Chamdo	F2c1	73, 249d, 263, 310, 750, 1005, 1438, 1824, 2706, 3106N, 3970, 4769, 6392, 7028, 7828, 8860, 10310, 10535, 10586, 11150, 11719, 12338, 13708, 13722, 13928C, 14766, 15326, 15714, 16037, 16183C, 16189, 16304, 16355, 16519
K38248	Tib_2	Chamdo	G3a1a1a	16T, 73, 143, 150, 152, 263, 310, 489, 709, 750, 1438, 2706, 3106N, 4674, 4769, 4833, 5108, 7028, 8347, 8701, 8860, 8861, 9540, 10398, 10400, 10873, 11719, 11914, 12030, 12612, 12705, 14569, 14766, 14783, 15043, 15301, 15326, 15746, 16215, 16223, 16274
K38392	Tib_2	Chamdo	M11a2	73, 146, 198, 215, 263, 310, 318, 326, 489, 739, 750, 1095, 1438, 2706, 3106N, 3523, 4769, 6531, 7028, 7642, 8108, 8230, 8701, 8860, 9540, 9950, 10398, 10400, 10873, 10993, 11719, 11969, 12141, 12705, 13074, 14340, 14766, 14783, 15043, 15204, 15301, 15326, 16051, 16173, 16223
K38367	Tib_2	Chamdo	M11a2a1	73, 146, 200, 215, 263, 309+C, 315+C, 318, 326, 489, 523-524d, 750, 1095, 1438, 1822, 2706, 3106N, 4769, 6531, 7028, 7055, 7642, 8108, 8277, 8288-8289d, 8477, 8701, 8860, 9540, 9950, 10398, 10400, 10873, 11404, 11719, 11969, 12705, 13074, 14340, 14766, 14783, 15043, 15301, 15326, 16173, 16223, 16257, 16362
K38218	Tib_2	Chamdo	M13a1b	73, 152, 263, 310, 489, 750, 1438, 2706, 3106N, 3644, 4769, 5773, 6023, 6253, 6620, 7028, 8701, 8860, 9540, 10398, 10400, 10411, 10790, 10873, 11719, 12705, 13135, 14766, 14783, 15043, 15301, 15326, 15924, 16145, 16148, 16188, 16189, 16223, 16381, 16497
K38278	Tib_2	Chamdo	M61a	73, 152, 263, 309+C, 315+C, 489, 750, 980, 1193, 1342, 1438, 2706, 3106N, 3438, 4769, 5582C, 6253, 7028, 8269, 8701, 8790, 8860, 9540, 10398, 10400, 10873, 11719, 11810, 12705, 12732, 14766, 14783, 15043, 15301, 15326, 16093, 16223, 16270, 16362, 16381, 16519
K38330	Tib_2	Chamdo	M8a1	73, 263, 310, 489, 750, 1438, 2706, 3106N, 4314d, 4715, 4769, 6179, 7028, 7196A, 7861, 8453, 8584, 8684, 8701, 8860, 9540, 10398, 10400, 10873, 11719, 12705, 14470, 14766, 14783, 15043, 15047, 15301, 15326, 15487T, 15697, 16223, 16298, 16319
K38232	Tib_2	Chamdo	M9a1a1c1b1a6	73, 263, 309+C, 315+C, 489, 711, 750, 1041, 1438, 2706, 3106N, 3394, 4491, 4769, 7028, 7142, 7697, 8701, 8860, 9242, 9540, 9632, 10398, 10400, 10873, 11719, 12705, 14308, 14417, 14766, 14783, 15043, 15301, 15326, 16223, 16234, 16316, 16362
K38230	Tib_2	Chamdo	M9a1a1c1b1a	73, 263, 309+CC, 315+C, 489, 711, 750, 1041, 1438, 2667, 2706, 3106N, 3394, 4491, 4769, 5899+XC, 7028, 7142, 7697, 8701, 8860, 9242, 9540, 10398, 10400, 10873, 11719, 12705, 14308, 14417, 14766, 14783, 15043, 15301, 15310, 15326, 16223, 16234, 16316, 16362
K38280	Tib_2	Chamdo	M9a1a1c1b1a2	73, 146, 263, 297, 309+CC, 315+C, 489, 711, 750, 1041, 1438, 2706, 3106N, 3394, 3438, 4491, 4769, 5899+XC, 6446, 7028, 7142, 7697, 8701, 8860, 9242, 9540, 10398, 10400, 10873, 11719, 12705, 14308, 14417, 14766, 14783, 15043, 15301, 15326, 16223, 16234, 16316, 16362
K38275	Tib_2	Chamdo	M13a1b	73, 152, 263, 310, 489, 750, , 1438, , 2706, 3106N, 3644, 4769, 5773, 6023, 6253, 6620, 7028, 8155, 8701, 8860, 9540, 10398, 10400, 10411, 10790, 10873, 11719, 12705, 13135, 14766, 14783, 15043, 15301, 15326, 15924, 16145, 16148, 16188, 16189, 16223, 16381
K39041	Tib_2	Chamdo	G3a1a	16T, 73, 143, 150, 263, 315+C, 489, 709, 750, 825A, 1106, 1151A, 1173, 1185, 1211, 1227, 1230, 1231T, 1249, 1303, 1315, 1320, 1438, 2523, 2557, 2625, 2667, 2706, 3106N, 4769, 4833, 5108, 5703, 7028, 8701, 8860, 8861, 9540, 10172, 10398, 10400, 10873, 11719, 11914, 12705, 14569, 14766, 14783, 15043, 15226, 15301, 15326, 15746, 16215, 16223, 16274
K38703	Tib_2	Chamdo	M13a2a1c	73, 152, 263, 315+C, 489, 513, 750, 825A, 1211, 1438, 1883, 1888, 1977, 2523, 2557, 2625, 2667, 2706, 2786, 3106N, 3644, 4596, 4769, 5773, 6023, 6253, 6620, 7028, 8405, 8563, 8701, 8860, 9540, 10373, 10398, 10400, 10411, 10790, 10873, 11204, 11719, 12705, 13395, 14766, 14783, 15043, 15236, 15301, 15326, 15924, 15944d, 16145, 16168, 16188, 16223, 16257, 16311, 16362, 16519

K38421	Tib_2	Chamdo	G2a1h	73, 263, 310, 403, 489, 709, 750, 1438, 2706, 3106N, 3777, 4769, 5108, 5601, 7028, 7600, 8307, 8701, 8860, 9377, 9540, 9575, 10398, 10400, 10873, 11719, 12705, 13194, 13563, 14200, 14569, 14766, 14783, 15043, 15301, 15326, 16223, 16227, 16278, 16362
K38790	Tib_2	Chamdo	G3a1a	16T, 73, 143, 150, 263, 309+CC, 315+C, 489, 524+AC, 709, 750, 1438, 2706, 3106N, 4769, 4833, 5108, 7028, 8701, 8860, 8861, 9540, 10398, 10400, 10873, 11719, 11914, 12705, 14569, 14766, 14783, 15043, 15301, 15326, 15746, 16215, 16223, 16274
K39038	Tib_2	Chamdo	M9a1a1c1b1a	73, 263, 309+CC, 315+C, 489, 711, 750, 1041, 1438, 2706, 3106N, 3394, 4491, 4769, 5899+XC, 6680, 7028, 7142, 7697, 8701, 8860, 9242, 9540, 10398, 10400, 10873, 11719, 12477, 12705, 13681, 14308, 14417, 14766, 14783, 15043, 15301, 15326, 16223, 16234, 16316, 16355, 16362
K38184	Tib_2	Chamdo	D4j1a1f	73, 263, 310, 489, 750, 1438, 2706, 3010, 3106N, 4769, 4883, 5178A, 5262, 6383, 7028, 7375, 7581, 7783, 8414, 8701, 8860, 9053, 9540, 10398, 10400, 10873, 10933, 11696, 11719, 11963, 12130, 12358, 12705, 14668, 14766, 14783, 15043, 15295, 15301, 15326, 16086, 16223, 16362
K38634	Tib_2	Chamdo	F1g1	73, 249d, 263, 309+C, 315+C, 523-524d, 750, 1438, 2389, 2706, 3106N, 3203, 3398, 3621, 3970, 4769, 6351, 6392, 6962, 7028, 8648, 8860, 10310, 10609, 11335, 11719, 12406, 12882, 13928C, 14766, 14798, 15326, 15499, 16093, 16183C, 16189, 16193+C, 16304, 16519
K38285	Tib_2	Chamdo	G3b1a1	73, 195, 263, 309+C, 315+C, 489, 523-524d, 709, 750, 1438, , 2706, 3106N, 4769, 4833, 5108, 7028, 8701, 8860, 9540, 9599, 10398, 10400, 10511, 10873, 11719, 12705, 13477, 14569, 14605, 14766, 14783, 15043, 15301, 15326, 15927, 16223, 16249, 16274, 16362
K38714	Tib_2	Chamdo	A	73, 152, 235, 263, 310, 663, 750, 1438, 1736, 2706, 3106N, 4248, 4769, 4824, 6040, 7028, 8794, 8860, 11719, 12705, 14766, 15326, 16092, 16223, 16290, 16319, 16362
K38493	Tib_2	Chamdo	A11a1a3	73, 152, 235, 263, 309+C, 315+C, 663, 750, 1005, 1438, 1736, 2706, 3106N, 4248, 4769, 4824, 5899+XC, 6755, 7028, 8794, 8843, 8860, 9650, 11719, 12705, 13708, 14766, 15326, 16094, 16223, 16290, 16293C, 16319, 16519
K38420	Tib_2	Chamdo	A11a1a4	73, 152, 182CT, 235, 263, 309+CC, 315+C, 523-524d, 663, 750, 1005, 1438, 1736, 2706, 3106N, 4248, 4769, 4824, 5899+XC, 6755, 7028, 8794, 8843, 8860, 9055, 9650, 11719, 12705, 14766, 15326, 16223, 16290, 16293C, 16319
K38233	Tib_2	Chamdo	A21c	73, 151, 152, 235, 263, 310, 663, 750, 1438, 1736, 1888, 2706, 3106N, 4248, 4769, 4824, 7028, 8506, 8794, 8860, 11719, 12705, 13145, 14364, 14766, 14962, 15326, 16223, 16290, 16319, 16362
K38960	Tib_2	Chamdo	C4a2c2	73, 249d, 263, 309+C, 315+C, 489, 750, 1438, 2232+A, 2706, 3106N, 3394, 3552A, 4715, 4769, 6026, 7028, 7196A, 8584, 8701, 8860, 9540, 9545, 10398, 10400, 10873, 11719, 11914, 11969, 12672, 12705, 13263, 13967, 14318, 14766, 14783, 15043, 15204, 15301, 15326, 15487T, 16086, 16223, 16298, 16327, 16357, 16519
K38992	Tib_2	Chamdo	D4b2b8	73, 152, 194, 263, 309+C, 315+C, 390, 489, 523-524d, 750, 1382C, 1438, 2706, 3010, 3106N, 3398, 4769, 4883, 5178A, 7028, 8020, 8414, 8701, 8754, 8860, 8964, 9296, 9540, 9824A, 10398, 10400, 10873, 11719, 12705, 13500, 14668, 14766, 14783, 15043, 15301, 15326, 15613, 16223, 16362, 16519
K38792	Tib_2	Chamdo	D4b2b9	73, 194, 263, 310, 489, 523-524d, 750, 1382C, 1438, 2706, 3010, 3106N, 3316, 4491, 4769, 4883, 5178A, 7028, 7849, 8020, 8414, 8545, 8701, 8805, 8860, 8964, 9296, 9540, 9824A, 10398, 10400, 10873, 11719, 12705, 14668, 14751, 14766, 14783, 15043, 15301, 15326, 16111, 16223, 16362, 16519
K39009	Tib_2	Chamdo	D4j1a2	73, 263, 309+C, 315+C, 489, 750, 1438, 2706, 3010, 3106N, 4769, 4883, 5178A, 5262, 7028, 7783, 7984GA, 8414, 8701, 8860, 9540, 10398, 10400, 10873, 11696, 11719, 12130, 12358, 12705, 13635, 14668, 14766, 14783, 15043, 15301, 15326, 16086, 16223, 16271, 16362
K38525	Tib_2	Chamdo	D5a2a1c	73, 150, 263, 310, 489, 523-524d, 750, 752, 1107, 2706, 2833, 3106N, 4769, 4883, 5178A, 5301, 7028, 8701, 8860, 9180, 9540, 10397, 10398, 10400, 10873, 11719, 11944, 12026, 12705, 14766, 14783, 15043, 15301, 15326, 16164, 16172, 16182C, 16183C, 16189, 16223, 16243, 16266, 16362
K38621	Tib_2	Chamdo	F1c1a1a	73, 152, 249d, 263, 309+C, 315+C, 523-524d, 573+XC, 709, 750, 1438, 2706, 3106N, 3768, 3970, 4769, 6392, 6599, 6962, 7028, 8860, 9053, 9647, 10310, 10454, 10609, 11719, 12406, 12882, 13759, 13928C, 14766, 15326, 16111, 16129, 16266, 16304, 16519
K38263	Tib_2	Chamdo	F1c1a1a2	73, 143, 152, 249d, 263, 309+C, 315+C, 523-524d, 573+XC, 709, 750, 1438, 2706, 3106N, 3970, 4769, 6392, 6599, 6962, 7028, 8412, 8701, 8860, 9053, 9647, 10310, 10454, 10609, 10739, 11719, 12406, 12882, 13759, 13928C, 14766, 15326, 16111, 16129, 16266, 16304, 16519
K38231	Tib_2	Chamdo	F1d	73, 146, 185, 235, 249d, 263, 309+C, 315+C, 523-524d, 750, 1438, 1734, 2706, 3106N, 3849, 3864, 3970, 4769, 5460, 5628, 6392, 6962, 7028, 7424, 7738, 8020, 8860, 10310, 10609, 11092, 11719, 12406, 12882, 13928C, 14766, 15326, 15402, 16304, 16519
K38181	Tib_2	Chamdo	F1d	73, 146, 249d, 263, 309+C, 315+C, 523-524d, 750, 1438, 1734, 2706, 3106N, 3970, 4769, 5460, 5628, 6392, 6962, 7028, 7738, 8860, 10310, 10609, 11092, 11719, 12406, 12882, 13928C, 14766, 15326, 15402, 16304, 16519
K39045	Tib_2	Chamdo	F1d1a1	73, 146, 249d, 263, 310, 523-524d, 750, 1438, 1734, 2706, 3106N, 3970, 4769, 5628, 6392, 6962, 7028, 7738, 8860, 10310, 10609, 11719, 12406, 12882, 13135, 13928C, 14766, 14782, 15326, 15402, 16240, 16284, 16304, 16519

K38409	Tib_2	Chamdo	G3a1a2	16T, 73, 143, 150, 263, 309+C, 315+C, 489, 709, 750, 1438, 2706, 3106N, 4769, 4833, 5108, 6962, 7028, 8701, 8860, 8861, 9540, 10398, 10400, 10873, 11719, 11914, 12705, 14569, 14766, 14783, 15043, 15301, 15326, 15465, 15497, 15746, 16148, 16153, 16215, 16223, 16274
K38894	Tib_2	Chamdo	M13a2a1b	73, , 263, 309+C, 315+C, 489, 513, 523-524d, 750, 1438, 2706, 2786, 3106N, 3644, 4769, 5773, 6023, 6253, 6620, 7028, 8405, 8701, 8860, 9540, 10235, 10373, 10398, 10400, 10411, 10790, 10873, 11719, 12705, 13395, 14766, 14783, 15043, 15301, 15326, 15924, 15944d, 16145, 16168, 16188, 16223, 16257, 16311, 16519
K38492	Tib_2	Chamdo	M62b1b	73, 150, 203, 204, 263, 309+C, 315+C, 489, 750, 1438, 2706, 2735, 3030, 3106N, 3511, 4561, 4769, 7028, 7664, 8149, 8701, 8860, 9540, 10398, 10400, 10410, 10873, 11719, 12705, 13708, 14766, 14783, 15043, 15301, 15326, 15510, 15520, 15629, 15721, 16183C, 16189, 16193+C, 16223, 16260, 16295, 16519
K39036	Tib_2	Chamdo	M62b1a1b	73, 150, 203, 204, 263, 309+C, 315+C, 489, 750, 1438, 2706, 2735, 3106N, 3511, 4561, 4769, 7028, 7664, 7844, 8149, 8281-8289d, 8701, 8860, 9540, 10398, 10400, 10873, 10978, 11431, 11719, 12705, 13708, 14766, 14783, 15043, 15301, 15326, 15510, 15520, 15629, 15721, 16169, 16223, 16260, 16519
K38846	Tib_2	Chamdo	M62b1a1b	73, 150, 203, 204, 263, 309+C, 315+C, 489, 750, 1438, 2706, 2735, 3106N, 3511, 4561, 4769, 7028, 7664, 7844, 7930, 8149, 8281-8289d, 8701, 8860, 9540, 10398, 10400, 10873, 10978, 11431, 11719, 12705, 13708, 14766, 14783, 15043, 15301, 15326, 15510, 15520, 15629, 15721, 16169, 16223, 16260, 16519
K38441	Tib_2	Chamdo	M9a1a1c1b1a8	73, 263, 309+CC, 315+C, 489, 711, 750, 1041, 1438, 2706, 3106N, 3394, 4491, 4769, 7028, 7142, 7697, 8701, 8860, 9242, 9540, 10398, 10400, 10873, 11719, 12705, 13419, 14308, 14417, 14766, 14783, 15043, 15301, 15326, 16223, 16234, 16316, 16362
K38500	Tib_2	Chamdo	M9a1b1d	73, 150, 152, 153, 263, 310, 489, 750, 1041, 1438, 2706, 3106N, 3394, 4491, 4769, 7028, 8701, 8860, 9540, 10398, 10400, 10873, 11719, 12362, 12705, 14308, 14766, 14783, 15043, 15119, 15301, 15326, 15671, 16158, 16223, 16234, 16362, 16519
K38885	Tib_2	Chamdo	Z3a	73, 249d, 263, 279, 309+C, 315+C, 489, 750, 1438, 1700, 2706, 3106N, 4491, 4715, 4769, 5399, 6752, 7028, 7196A, 8584, 8701, 8860, 9090, 9128, 9540, 9713, 10208, 10286, 10398, 10400, 10873, 11719, 12705, 12810, 13620, 13942, 14766, 14783, 15043, 15301, 15326, 15487T, 15784, 15928, 16137, 16185, 16215, 16223, 16260, 16298, 16355
K38385	Tib_2	Chamdo	C4a2c2	73, 249d, 263, 310, 489, 750, 1438, 2232+A, 2706, 3106N, 3394, 3552A, 4715, 4769, 6026, 7028, 7196A, 8584, 8701, 8860, 9540, 9545, 10398, 10400, 10873, 11719, 11914, 11969, 12672, 12705, 13263, 13967, 14318, 14488, 14766, 14783, 15043, 15204, 15301, 15487T, 16086, 16223, 16298, 16327, 16357, 16519
K39028	Tib_2	Chamdo	D5a2a1d	73, 150, 225T, 263, 309+C, 315+C, 489, 523-524d, 650, 750, 752, 1107, 2706, 3106N, 3402, 4769, 4883, 5178A, 5301, 7028, 8701, 8860, 9180, 9540, 10397, 10398, 10400, 10873, 11152, 11719, 11944, 12026, 12705, 14766, 14783, 15043, 15301, 15326, 16093?, 16164, 16172, 16182C, 16183C, 16189, 16223, 16266, 16362
K38847	Tib_2	Chamdo	M9a1a1c1b1a	73, 263, 310, 489, 711, 750, 1041, 1438, 2706, 3106N, 3394, 4491, 4769, 5899+XC, 7028, 7142, 7697, 8701, 8860, 9242, 9540, 10398, 10400, 10604, 10873, 11719, 12705, 14308, 14417, 14766, 14783, 15043, 15301, 15326, 16223, 16234, 16316, 16362
K39031	Tib_2	Chamdo	G2a1f	73, 263, 310, 489, 709, 750, 1438, 2706, 3106N, 3243AG, 4769, 4833, 5108, 5601, 7028, 7600, 8701, 8860, 9377, 9540, 9575, 10398, 10400, 10873, 11719, 12705, 13563, 13884, 14200, 14569, 14766, 14783, 15043, 15301, 15326, 16051, 16114, 16223, 16227, 16278, 16291, 16362
K39017	Tib_2	Chamdo	A14a1	73, 151, 152, 200, 235, 263, 310, 523-524d, 663, 735, 750, 1438, 1736, 2706, 3106N, 4248, 4769, 4824, 7028, 8334, 8794, 8860, 8862, 11719, 12004, 12705, 12990, 14766, 14803, 15326, 16093, 16172, 16223, 16290, 16319, 16362
K38935	Tib_2	Chamdo	D5a2a1d	73, 150, 263, 309+C, 315+C, 489, 523-524d, 650, 750, 752, 1107, 2706, 3106N, 3402, 4769, 4883, 5178A, 5301, 7028, 8701, 8860, 9180, 9540, 10397, 10398, 10400, 10873, 11152, 11719, 11944, 12026, 12358, 12705, 14766, 14783, 15043, 15301, 15326, 16164, 16172, 16182C, 16183C, 16189, 16209, 16223, 16266, 16362
K38928	Tib_2	Chamdo	M9a1b2	73, 150, 152, 263, 309+CC, 315+C, 489, 750, 1041, 1438, 2706, 3106N, 3394, 4491, 4721, 4769, 7028, 8701, 8860, 9540, 10398, 10400, 10873, 10951A, 11719, 12362, 12705, 13563, 14308, 14766, 14783, 15043, 15301, 15326, 15671, 16223, 16234, 16362, 16519
K38902	Tib_2	Chamdo	D4h4	73, 195, 225, 263, 309+CC, 315+C, 335, 489, 593TC, 750, 1438, 2706, 3010, 3106N, 3336, 3644, 4769, 4883, 5048, 5074, 5178A, 7028, 7502, 8414, 8701, 8860, 9540, 10398, 10400, 10873, 11719, 12705, 14668, 14766, 14783, 15022, 15043, 15301, 15326, 16051, 16223, 16311, 16362
K38852	Tib_2	Chamdo	B4a1	73, 263, 309+CC, 315+C, 523-524d, 750, 1438, 2706, 3106N, 4769, 5465, 7028, 7340, 8281-8289d, 8860, 8886, 9123, 9233, 10238, 11719, 14562, 14766, 15326, 15670, 16182C, 16183C, 16189, 16217, 16261, 16348, 16519
K38764	Tib_2	Chamdo	F1c1a1a1	73, 152, 249d, 263, 310, 523-524d, 709, 750, 1438, 2706, 3106N, 3970, 4769, 6392, 6599, 6962, 7028, 8860, 9053, 9647, 10310, 10454, 10609, 11719, 12406, 12882, 13759, 13928C, 14766, 15244, 15326, 16111, 16129, 16304, 16519
K38739	Tib_2	Chamdo	D4b2b9	73, 194, 263, 310, 489, 523-524d, 750, 1382C, 1438, 2706, 3010, 3106N, 4769, 4883, 5178A, 7028, 8020, 8414, 8545, 8701, 8860, 8964, 9296, 9540, 9824A, 10398, 10400, 10873, 11719, 12705, 14668, 14751, 14766, 14783, 15043, 15301, 15326, 16111, 16223, 16362, 16519

K38629	Tib_2	Chamdo	M9a1a1c1b1a	73, 263, 309+C, 315+C, 489, 711, 750, 1041, 1438, 2706, 3106N, 3394, 4491, 4769, 5899+XC, 7028, 7142, 7697, 8701, 8860, 9242, 9540, 10398, 10400, 10873, 11719, 12705, 14308, 14417, 14766, 14783, 15043, 15301, 15326, 16092, 16174, 16223, 16234, 16316, 16344, 16362
K38598	Tib_2	Chamdo	G2b1b	73, 263, 309+C, 315+C, 489, 709, 750, 1438, 2706, 3106N, 4769, 4833, 4853, 5108, 5601, 7028, 8701, 8860, 8877, 9540, 10398, 10400, 10873, 11719, 12375, 12705, 13563, 14569, 14766, 14783, 15043, 15301, 15326, 16223, 16362
K38499	Tib_2	Chamdo	M9a1b1d	73, 150, 152, 153, 263, 310, 489, 750, 1041, 1438, 2706, 3106N, 3394, 4491, 4769, 7028, 8701, 8860, 9540, 10398, 10400, 10873, 11719, 12362, 12705, 14308, 14766, 14783, 15043, 15119, 15301, 15326, 15671, 16158, 16223, 16234, 16362, 16519
K38494	Tib_2	Chamdo	D4h	73, 263, 310, 489, 571G, 750, 1438, 2706, 3010, 3106N, 3336, 3644, 3818, 4580, 4769, 4883, 5048, 5178A, 6663, 7028, 8414, 8701, 8860, 9424, 9540, 10192, 10398, 10400, 10873, 11719, 12705, 13879, 14668, 14766, 14783, 15043, 15301, 15326, 16223, 16362, 16519
K38453	Tib_3	Lhasa	D4s	73, 199, 263, 309+CC, 315+C, 489, 750, 813, 1438, 2706, 3010, 3106N, 4769, 4883, 5178A, 5483, 7028, 8414, 8701, 8860, 9540, 9944, 10321, 10398, 10400, 10873, 11719, 11986, 12705, 14668, 14766, 14783, 15043, 15301, 15326, 15734, 16223, 16362, 16519
K38148	Tib_3	Lhasa	M9a1a1c1b1a11	73, 263, 309+C, 315+C, 489, 711, 750, 1041, 1438, 2706, 3106N, 3221, 3394, 4491, 4769, 5492, 5582, 5899+XC, 7028, 7142, 7697, 8701, 8860, 9139, 9242, 9540, 10398, 10400, 10873, 11719, 12705, 14308, 14417, 14766, 14783, 15043, 15301, 15326, 16223, 16234, 16316, 16362
K38144	Tib_3	Lhasa	U7a	73, 151, 152, 263, 309+C, 315+C, 523-524d, 750, 980, 1438, 1811, 2706, 3106N, 3741, 4769, 5360, 7028, 8137, 8684, 8860, 10142, 11399, 11467, 11719, 12308, 12372, 13500, 14569, 14766, 15326, 16309, 16318T, 16519
K38200	Tib_3	Lhasa	G2b1b1	73, 263, 309+C, 315+C, 489, 709, 750, 1438, 2706, 3106N, 3221, 4769, 4833, 4853, 5108, 5601, 7028, 8701, 8860, 8877, 9540, 10398, 10400, 10873, 11719, 12375, 12705, 13563, 14569, 14766, 14783, 15043, 15301, 15326, 16223, 16269, 16362
K38188	Tib_3	Lhasa	M9a1a1c1b1a	73, 189, 263, 309+C, 315+C, 489, 711, 750, 1041, 1438, 2706, 3106N, 3394, 4491, 4769, 5899+XC, 7028, 7142, 7697, 8701, 8860, 9242, 9540, 10398, 10400, 10873, 11719, 12705, 14308, 14417, 14766, 14783, 15043, 15301, 15326, 16223, 16234, 16316, 16362, 16519
K38171	Tib_3	Lhasa	G2a1h1	73, 146, 263, 283, 309+CC, 315+C, 489, 709, 750, 1438, 2706, 3106N, 4158, 4769, 4833, 5108, 5601, 7028, 7600, 8701, 8860, 9377, 9540, 9575, 10398, 10400, 10873, 11719, 12705, 13194, 13563, 14200, 14569, 14766, 14783, 15043, 15301, 15326, 16223, 16227, 16278, 16362
K38169	Tib_3	Lhasa	M9a1a1c1b1a6	73, 263, 309+C, 315+C, 489, 711, 750, 1041, 1438, 2706, 3106N, 3394, 4491, 4769, 7028, 7142, 7697, 7754, 8597, 8701, 8860, 9242, 9540, 9632, 10398, 10400, 10873, 11719, 12705, 14308, 14417, 14422, 14766, 14783, 15043, 15301, 15326, 16223, 16234, 16316, 16362
K38165	Tib_3	Lhasa	C4a1a1a1	73, 249d, 263, 310, 489, 523-524d, 750, 1438, 1715, 2232+AA, 2706, 3106N, 3327, 3552A, 3576, 4715, 4769, 4884, 4958, 6026, 7028, 7196A, 7999, 8508, 8584, 8701, 8860, 9540, 9545, 10398, 10400, 10873, 11719, 11914, 11969, 12672, 12705, 13263, 14318, 14766, 14783, 15043, 15204, 15301, 15326, 15487T, 15968, 16093, 16129, 16223, 16224, 16298, 16327, 16519
K38088	Tib_3	Lhasa	M13a2	73, 152, 195, 263, 310, 489, 513, 749, 750, 1438, 2706, 2786, 3106N, 3644, 4769, 5773, 6023, 6253, 6620, 7028, 8405, 8701, 8860, 9540, 10373, 10398, 10400, 10411, 10790, 10873, 11719, 12705, 13395, 14766, 14783, 15043, 15301, 15326, 15924, 15944d, 16145, 16168, 16188, 16223, 16257, 16311, 16519
K38053	Tib_3	Lhasa	G2b1b2a	73, 263, 309+C, 315+C, 489, 709, 750, 1438, 2706, 3106N, 3975, 4769, 4833, 4853, 5108, 5601, 7028, 8701, 8764, 8860, 8877, 9540, 10398, 10400, 10873, 11719, 12285, 12375, 12705, 12795, 13563, 14563G, 14569, 14766, 14783, 15043, 15301, 15326, 16037, 16223, 16324, 16362
K38028	Tib_3	Lhasa	G2a1h1	73, 146, 263, 283, 309+C, 315+C, 489, 709, 750, 1438, 2706, 3106N, 4158, 4769, 4833, 4917, 5108, 5601, 7028, 7600, 8701, 8860, 9377, 9540, 9575, 10398, 10400, 10873, 11719, 12705, 13194, 13563, 14200, 14569, 14766, 14783, 15043, 15301, 15326, 15773, 16080, 16223, 16227, 16278, 16362
K38199	Tib_3	Lhasa	F1g	73, 249d, 263, 309+CC, 315+C, 523-524d, 750, 1438, 2389, 2706, 3106N, 3398, 3970, 4769, 6392, 6962, 7028, 8860, 10310, 10609, 11719, 12406, 12882, 13928C, 14766, 15326, 16183C, 16189, 16304, 16519
K38444	Tib_3	Lhasa	M10a1b	73, 263, 309+C, 315+C, 489, 567C, 709, 750, 1438, 1719, 2706, 3106N, 3172+C, 4140, 4769, 7028, 7250, 8701, 8793, 8856, 8860, 9540, 10398, 10400, 10646, 10873, 11719, 12549, 12705, 13152, 13466, 13768, 14502, 14766, 14783, 15040, 15043, 15071, 15218, 15301, 15326, 16066, 16175C, 16223, 16311, 16519
K38179	Tib_3	Lhasa	M9a1a1c1b1a5	73, 263, 309+C, 315+C, 489, 711, 750, 1041, 1438, 2706, 3106N, 3394, 4491, 4769, 5899+XC, 7028, 7142, 7697, 8701, 8860, 9242, 9540, 10398, 10400, 10873, 11719, 12705, 14308, 14766, 14783, 15043, 15301, 15326, 15766, 16223, 16234, 16316, 16362
K38246	Tib_3	Lhasa	M9a1b1e	73, 150, 152, 153, 263, 310, 489, 709, 750, 1041, 1438, 2706, 3106N, 3394, 4491, 4769, 7028, 8701, 8860, 9540, 10398, 10400, 10873, 11719, 12362, 12705, 14308, 14766, 14783, 15043, 15301, 15326, 15671, 16158, 16223, 16234, 16362, 16519
K38294	Tib_3	Lhasa	Z3a	73, 152, 207, 249d, 263, 309+CC, 315+C, 489, 750, 1393, 1438, 2706, 3106N, 4715, 4769, 6752, 7028, 7196A, 7775, 8292, 8584, 8701, 8860, 9090, 9540, 9713, 10208, 10398, 10400, 10527, 10873, 11719, 12705, 13620, 14766, 14783, 15043, 15301, 15326, 15487T, 15784, 15928, 16185, 16223, 16260, 16298

K38198	Tib_3	Lhasa	A11a1a	73, 152, 235, 263, 309+C, 315+C, 523-524d, 663, 750, 1005, 1438, 1736, 2706, 3106N, 4248, 4769, 4824, 5899+XC, 6755, 7028, 8794, 8843, 8860, 9650, 11719, 12705, 14766, 15326, 16093, 16223, 16290, 16293C, 16319, 16519, 16527
K38173	Tib_3	Lhasa	A11a1a	73, 152, 235, 263, 309+C, 315+C, 523-524d, 663, 750, 1005, 1438, 1736, 2706, 3106N, 4248, 4769, 4824, 5899+XC, 6755, 7028, 8794, 8843, 8860, 9650, 11719, 12705, 14766, 15326, 16223, 16290, 16293C, 16319
K38187	Tib_3	Lhasa	F1a1c	73, 249d, 263, 310, 523-524d, 548, 750, 1438, 2706, 3106N, 3970, 4086, 4769, 6392, 6962, 7028, 7804, 8410, 8860, 9053, 9548, 9554, 10211, 10310, 10609, 11719, 12406, 12882, 13149, 13759, 13928C, 14766, 15326, 16129, 16162, 16172, 16304, 16519
K38445	Tib_3	Lhasa	F1b1c	73, 152, 249d, 263, 309+C, 315+C, 523-524d, 750, 1438, 2706, 3106N, 3970, 4732, 4769, 5147, 5508, 6392, 6962, 7028, 8860, 10235, 10310, 10609, 10976, 11719, 11732, 12406, 12882, 13928C, 14476, 14766, 15119, 15326, 16134, 16183C, 16189, 16232A, 16249, 16304, 16317, 16519
K38063	Tib_3	Lhasa	F1c1a1a	73, 152, 249d, 263, 309+CC, 315+C, 523-524d, 573+XC, 709, 750, 1438, 2706, 3106N, 3970, 4769, 6392, 6599, 6962, 7028, 8860, 9053, 9647, 10310, 10454, 10609, 11719, 11731C, 12406, 12501, 12882, 13759, 13928C, 14766, 15326, 16111, 16129, 16259, 16266, 16304, 16519
K38029	Tib_3	Lhasa	G2a1i	73, 263, 310, 489, 709, 750, 1438, 2706, 3106N, 4769, 5108, 5601, 7028, 7600, 8400, 8701, 8860, 9377, 9540, 9575, 10398, 10400, 10873, 11719, 12705, 13563, 14200, 14569, 14766, 14783, 15043, 15301, 15326, 16129, 16201, 16223, 16278, 16360, 16362, 16555
K38084	Tib_3	Lhasa	M11a2a	73, 146, 198, 200, 215, 263, 310, 318, 326, 489, 750, 1095, 1438, 2706, 3106N, 4769, 5964, 6531, 7028, 7543, 7642, 8108, 8701, 8860, 9540, 9950, 10398, 10400, 10873, 11404, 11719, 11969, 12705, 13074, 14340, 14766, 14783, 15043, 15301, 15326, 15944d, 16093, 16173, 16223, 16311
K38086	Tib_3	Lhasa	M13a2a1b	73, 152, 263, 309+C, 315+C, 489, 513, 523-524d, 750, 1438, 2706, 2786, 3106N, 3644, 4769, 5773, 6023, 6253, 6620, 7028, 8405, 8701, 8860, 9540, 10235, 10373, 10398, 10400, 10411, 10790, 10873, 11719, 12705, 13395, 14766, 14783, 15043, 15301, 15326, 15773, 15924, 15944d, 16145, 16168, 16188, 16223, 16257, 16311, 16519
K38147	Tib_3	Lhasa	M62b1b1	73, 150, 203, 204, 263, 309+C, 315+C, 457, 489, 524+AC, 750, 1438, 2706, 2735, 3030, 3106N, 3511, 4561, 4769, 7028, 7609, 7664, 7807, 8149, 8701, 8860, 9540, 10398, 10400, 10873, 11719, 12705, 13708, 14766, 14783, 15043, 15301, 15326, 15510, 15520, 15629, 15721, 16223, 16260, 16295, 16519
K38416	Tib_3	Lhasa	M9a1a1c1b1a	73, 263, 310, 489, 711, 750, 1041, 1438, 2706, 3106N, 3394, 4491, 4769, 5899+XC, 7028, 7142, 7697, 8701, 8860, 9242, 9540, 10398, 10400, 10873, 11719, 12705, 14308, 14417, 14766, 14783, 15043, 15301, 15326, 16223, 16234, 16316, 16362
K38160	Tib_3	Lhasa	M9a1a1c1b1a	73, 263, 309+C, 315+C, 489, 711, 750, 1041, 1438, 2706, 3106N, 3394, 4491, 4769, 5894C, 7028, 7142, 7697, 8701, 8860, 9242, 9540, 9632, 10398, 10400, 10873, 11719, 12705, 14308, 14417, 14766, 14783, 15043, 15301, 15326, 16223, 16234, 16316, 16356, 16362
K38456	Tib_3	Lhasa	M9a1a1c1b1a2	73, 146, 263, 297, 309+C, 315+C, 489, 711, 750, 1041, 1438, 2625, 2706, 3106N, 3394, 3438, 4491, 4769, 5899+XC, 6446, 7028, 7142, 7697, 8701, 8860, 9242, 9540, 10398, 10400, 10873, 11719, 12705, 14308, 14417, 14766, 14783, 15043, 15301, 15326, 16051, 16129, 16223, 16234, 16316, 16362
K38067	Tib_3	Lhasa	M9a1b	73, 150, 152, 153, 195, 263, 310, 489, 523-524d, 709, 750, 1041, 1438, 2706, 3106N, 3394, 4491, 4769, 7028, 8701, 8860, 9540, 10398, 10400, 10873, 11719, 12362, 12705, 14308, 14766, 14783, 15043, 15301, 15326, 15671, 16223, 16234, 16362, 16519
K38175	Tib_3	Lhasa	A11a1a	64, 73, 152, 235, 263, 309+CC, 315+C, 523-524d, 663, 750, 1005, 1438, 1736, 2706, 3106N, 4248, 4769, 4824, 5899+XC, 6755, 7028, 8794, 8843, 8860, 9650, 11719, 12705, 14766, 15326, 15773, 16223, 16290, 16293C, 16319
K38162	Tib_3	Lhasa	A11a1a	73, 152, 235, 263, 309+C, 315+C, 523-524d, 663, 750, 1005, 1438, 1736, 2706, 3106N, 4248, 4769, 4824, 5899+XC, 6755, 7028, 8794, 8843, 8860, 9650, 10007, 11719, 12528, 12705, 14766, 15326, 16093, 16223, 16290, 16293C, 16319, 16519
K38164	Tib_3	Lhasa	B4b1a2	73, 263, 309+CCC, 315+C, 499, 750, 827, 1438, 2706, 3106N, 4769, 4820, 6023, 6216, 6413, 7028, 8266, 8281-8289d, 8860, 11719, 13092, 13590, 14766, 15326, 15535, 16136, 16182C, 16183C, 16189, 16217, 16256, 16519
K38099	Tib_3	Lhasa	C4a2c3	73, 249d, 263, 309+C, 315+C, 489, 750, 1438, 1628, 2232+A, 2706, 3106N, 3394, 3552A, 4715, 4769, 6026, 7028, 7196A, 8584, 8701, 8860, 9540, 9545, 9843, 10398, 10400, 10427, 10873, 11719, 11914, 11969, 12672, 12705, 13263, 13967, 14318, 14766, 14783, 15043, 15204, 15301, 15326, 15487T, 16223, 16298, 16327, 16357, 16519
K38138	Tib_3	Lhasa	F1d1a2a1	73, 146, 234, 249d, 263, 309+CC, 315+C, 523-524d, 750, 1438, 1734, 1888, 2706, 3106N, 3970, 4769, 5628, 6392, 6962, 7028, 7738, 8167, 8860, 10310, 10609, 11719, 12372, 12406, 12882, 13135, 13928C, 14766, 15326, 15402, 16145, 16188+CC, 16255, 16284, 16304, 16519
K38161	Tib_3	Lhasa	G1c	73, 263, 310, 489, 593, 709, 750, 1438, 1719, 2706, 3106N, 4353, 4769, 4833, 5108, 7028, 8200, 8701, 8860, 9540, 9682, 9824, 9966, 10398, 10400, 10873, 11008, 11719, 12361, 12705, 14569, 14766, 14783, 15043, 15301, 15323, 15326, 15497, 16223, 16293, 16362, 16519

K38142	Tib_3	Lhasa	M9a1b	73, 150, 152, 263, 310, 489, 750, 1041, 1438, 2706, 3106N, 3394, 4491, 4769, 7028, 8701, 8860, 9478, 9540, 10398, 10400, 10873, 11719, 12362, 12705, 14308, 14766, 14783, 15043, 15301, 15326, 15671, 16223, 16234, 16362, 16519
K38140	Tib_3	Lhasa	B4d4	73, 150, 263, 309+CC, 315+C, 750, 827, 1438, 2706, 2755, 3106N, 4769, 5372, 6324C, 7028, 8281-8289d, 8860, 11719, 11914, 13942, 14766, 15326, 15535, 15930, 16183C, 16189, 16193+CC, 16234, 16519
K38207	Tib_3	Lhasa	D4j1	73, 146, 263, 309+C, 315+C, 489, 750, 1438, 2706, 3010, 3106N, 4769, 4883, 5178A, 5262, 7028, 8414, 8701, 8860, 9540, 10398, 10400, 10873, 11696, 11719, 12705, 14668, 14766, 14783, 15043, 15301, 15326, 16223, 16362, 16526
K38304	Tib_3	Lhasa	D4j3b	73, 263, 309+C, 315+C, 338, 489, 750, 1438, 2706, 3010, 3106N, 4769, 4883, 5178A, 7028, 8414, 8701, 8860, 9540, 10398, 10400, 10873, 11696, 11719, 12705, 14668, 14766, 14783, 14851, 15043, 15301, 15326, 16184, 16223, 16311, 16362, 16519
K38261	Tib_3	Lhasa	F1c1a1a	73, 152, 249d, 263, 309+CC, 315+C, 523-524d, 573+XC, 709, 750, 1438, 2706, 3106N, 3970, 4769, 6340, 6392, 6599, 6962, 7028, 7702, 7775, 8860, 9053, 9647, 10310, 10398, 10454, 10609, 11719, 12406, 12882, 13759, 13928C, 14766, 15326, 16111, 16129, 16188+CC, 16266, 16304, 16519
K38301	Tib_3	Lhasa	M13a1b	73, 152, 263, 310, 489, 750, 1438, 1709, 2706, 3106N, 3644, 4769, 5773, 6023, 6253, 6620, 7028, 7711, 8701, 8860, 9540, 10398, 10400, 10411, 10790, 10873, 11719, 12705, 13135, 14766, 14783, 15043, 15301, 15326, 15924, 16145, 16148, 16188, 16189, 16223, 16381
K38228	Tib_3	Lhasa	M13a2a1a	73, 152, 263, 309+C, 315+C, 489, 513, 750, 1438, 2706, 2786, 3106N, 3644, 4769, 5773, 6023, 6253, 6620, 7028, 8405, 8701, 8860, 9540, 10373, 10398, 10400, 10411, 10790, 10873, 11719, 12705, 13395, 14766, 14783, 15043, 15301, 15326, 15924, 15944d, 16145, 16168, 16223, 16257, 16311, 16519
K38247	Tib_3	Lhasa	M9a1a1c1b1a	73, 263, 309+CC, 315+C, 489, 711, 750, 1041, 1438, 2706, 3106N, 3394, 4491, 4769, 5899+XC, 7028, 7142, 7697, 8701, 8860, 9242, 9540, 10398, 10400, 10873, 11719, 12705, 14308, 14417, 14766, 14783, 15043, 15301, 15326, 16223, 16234, 16316, 16362
K38317	Tib_3	Lhasa	M13a2	73, 152, 204, 263, 309+C, 315+C, 489, 513, 750, 1438, 2706, 2786, 3106N, 3644, 4769, 5773, 6023, 6253, 6620, 7028, 8405, 8701, 8860, 9540, 10373, 10398, 10400, 10411, 10790, 10873, 11719, 12705, 13395, 14766, 14783, 15043, 15301, 15326, 15924, 15944d, 16145, 16168, 16188, 16223, 16257, 16311, 16519
K38455	Tib_3	Lhasa	M9a1a1c1b1a	73, 263, 309+C, 315+C, 489, 711, 750, 1041, 1438, 2706, 3106N, 3394, 4491, 4769, 5899+XC, 7028, 7142, 7697, 8701, 8860, 9242, 9540, 10398, 10400, 10873, 11719, 12705, 14308, 14417, 14766, 14783, 15043, 15301, 15326, 16223, 16234, 16316, 16362
K38123	Tib_3	Lhasa	M9a1a1c1b1a	73, 263, 309+C, 315+C, 489, 711, 750, 1041, 1438, 2706, 3106N, 3221, 3394, 4491, 4769, 5492, 5582, 5899+XC, 7028, 7142, 7697, 8701, 8860, 9242, 9540, 10398, 10400, 10873, 11719, 12705, 14308, 14417, 14766, 14783, 15043, 15301, 15326, 16223, 16234, 16316, 16362
K38260	Tib_3	Lhasa	D4	73, 263, 309+C, 315+C, 489, 750, 1438, 2706, 2789A, 3010, 3106N, 3253, 4769, 4883, 5178A, 7028, 8414, 8701, 8860, 9540, 9725, 10398, 10400, 10873, 11719, 11746, 12432, 12705, 14668, 14766, 14783, 15043, 15301, 15326, 16093, 16188+CC, 16223, 16362
K38027	Tib_3	Lhasa	M9a1a1c1b1a3	73, 263, 309+C, 315+C, 489, 711, 750, 1041, 1438, 2706, 3106N, 3394, 4491, 4769, 7028, 7142, 7697, 8701, 8860, 9242, 9540, 10398, 10400, 10873, 11719, 12705, 13419, 14308, 14417, 14766, 14783, 15043, 15301, 15326, 16223, 16234, 16316, 16362
K38286	Tib_3	Lhasa	C4a1a1a3	73, 249d, 263, 310, 489, 523-524d, 750, , 1438, 1715, 2232+AA, 2706, 3106N, 3552A, 3576, 4715, 4769, 4884, 4958, 5484, 6026, 7028, 7196A, 7999, 8508, 8584, 8701, 8860, 9540, 9545, 10398, 10400, 10873, 11719, 11914, 11969, 12672, 12705, 13263, 14318, 14766, 14783, 15043, 15204, 15301, 15326, 15487T, 15968, 16129, 16223, 16224, 16298, 16327, 16519
K38202	Tib_3	Lhasa	C4a1a1a2	73, 234, 249d, 263, 310, 489, 750, 1438, 1715, 2232+AA, 2706, 3106N, 3552A, 3576, 4715, 4769, 4884, 4958, 5918, 6026, 7028, 7196A, 7999, 8405, 8508, 8584, 8701, 8860, 9540, 9545, 10398, 10400, 10873, 11719, 11914, 11969, 12672, 12705, 13263, 14318, 14766, 14783, 15043, 15204, 15301, 15326, 15487T, 15968, 16092, 16093, 16129, 16223, 16298, 16327, 16519
K38288	Tib_3	Lhasa	F1d1a2	73, 146, 249d, 263, 309+CCC, 315+C, 523-524d, 750, 1438, 1734, 2706, 3106N, 3970, 4769, 5628, 6392, 6962, 7028, 7738, 8860, 10310, 10609, 11719, 12406, 12882, 13135, 13928C, 14766, 15326, 15402, 16129, 16145, 16183C, 16189, 16284, 16304, 16519
K38153	Tib_3	Lhasa	G2a1h1	73, 146, 263, 283, 309+C, 315+C, 489, 709, 750, 1438, 2706, 3106N, 4158, 4769, 4833, 5108, 5601, 7028, 7600, 8701, 8860, 9377, 9540, 9575, 10398, 10400, 10873, 11719, 12705, 13194, 13563, 14200, 14569, 14766, 14783, 15043, 15301, 15326, 15773, 16080, 16223, 16227, 16278, 16362
K38186	Tib_3	Lhasa	M9a1a1c1b1a	73, 263, 309+C, 315+C, 489, 711, 750, 1041, 1438, 2706, 3106N, 3394, 4491, 4769, 5899+XC, 5978, 7028, 7142, 7697, 8701, 8860, 9242, 9540, 10398, 10400, 10873, 11719, 12705, 14308, 14417, 14766, 14783, 15043, 15301, 15326, 16223, 16234, 16316, 16362
K38449	Tib_3	Lhasa	C4a2	47, 73, 249d, 263, 309+C, 315+C, 489, 523-524d, 750, 1438, 2232+A, 2706, 3106N, 3552A, 4715, 4769, 6026, 6710, 7028, 7196A, 8584, 8654, 8701, 8802, 8860, 9540, 9545, 10398, 10400, 10873, 11719, 11914, 11969, 12131, 12672, 12705, 13263, 14318, 14766, 14783, 15043, 15204, 15301, 15326, 15487T, 16093, 16126, 16223, 16269, 16298, 16327, 16354, 16357, 16519

K38201	Tib_3	Lhasa	D4j3b	73, 263, 310, 338, 489, 750, 1438, 2706, 3010, 3106N, 4769, 4883, 5178A, 7028, 8414, 8496-8501d, 8581, 8701, 8860, 9540, 10398, 10400, 10873, 11696, 11719, 12705, 14668, 14766, 14783, 15043, 15301, 15326, 16184, 16223, 16311, 16362
K38408	Tib_3	Lhasa	F1c1a1a	73, 152, 249d, 263, 309+C, 315+C, 523-524d, 573+XC, 709, 750, 1438, 2706, 3106N, 3970, 4769, 6392, 6599, 6962, 7028, 8860, 9053, 9647, 10310, 10454, 10609, 11719, 12406, 12501, 12882, 13759, 13928C, 14766, 14830, 15326, 16111, 16129, 16266, 16304, 16519
K38446	Tib_3	Lhasa	F1g	73, 249d, 263, 309+CC, 315+C, 523-524d, 750, 1438, 2389, 2706, 3106N, 3398, 3630, 3970, 4769, 6392, 6962, 7028, 8860, 10310, 10609, 11719, 12406, 12882, 13840, 13928C, 14766, 15326, 16182C, 16183C, 16189, 16304, 16519
K38204	Tib_3	Lhasa	F1g	73, 249d, 263, 309+CC, 315+C, 523-524d, 750, 1438, 2389, 2706, 3106N, 3398, 3970, 4769, 6260, 6392, 6962, 7028, 8860, 10310, 10609, 11719, 12406, 12882, 13928C, 14766, 15326, 16183C, 16189, 16304, 16519
K38314	Tib_3	Lhasa	G3b3a	73, 152, 189, 263, 310, 489, 524+AC, 709, 750, 1438, 2706, 3106N, 3394, 4769, 4833, 5108, 7028, 8701, 8860, 9540, 10398, 10400, 10873, 10972, 11719, 12705, 13477, 14569, 14605, 14766, 14783, 15043, 15301, 15326, 15927, 16209, 16223, 16274, 16362
K38146	Tib_3	Lhasa	G3b1a1	73, 195, 263, 309+C, 315+C, 489, 523-524d, 709, 750, 1438, 2706, 3106N, 4769, 4833, 5108, 7028, 8701, 8860, 9540, 9599, 10398, 10400, 10511, 10873, 11719, 12616, 12705, 13477, 14569, 14605, 14766, 14783, 15043, 15301, 15326, 15927, 16223, 16249, 16274, 16362
K38460	Tib_3	Lhasa	M13a1b	73, 152, 263, 310, 489, 750, 1438, 2706, 3106N, 3644, 4769, 5773, 6023, 6253, 6599, 6620, 7028, 8701, 8860, 9540, 10398, 10400, 10411, 10790, 10873, 11719, 12705, 13135, 14766, 14783, 15043, 15301, 15326, 15924, 16145, 16148, 16188, 16189, 16223, 16381
K38442	Tib_3	Lhasa	M13a1b	73, 152, 263, 310, 489, 750, 1438, 1709, 2706, 3106N, 3644, 4769, 5773, 6023, 6253, 6620, 7028, 7711, 8701, 8860, 9540, 10398, 10400, 10411, 10790, 10873, 11719, 12705, 13135, 14766, 14783, 15043, 15301, 15326, 15924, 16145, 16148, 16188, 16189, 16223, 16381
K38300	Tib_3	Lhasa	M13a1b	73, 152, 263, 310, 489, 750, 1438, 1709, 2706, 3106N, 3644, 4769, 5773, 6023, 6253, 6620, 7028, 7711, 8701, 8860, 9540, 10398, 10400, 10411, 10790, 10873, 11719, 12705, 13135, 14766, 14783, 15043, 15301, 15326, 15924, 16145, 16148, 16188, 16189, 16223, 16381
K38440	Tib_3	Lhasa	M62b1a1b	73, 150, 203, 204, 263, 309+C, 315+C, 489, 750, 1438, 2706, 2735, 3106N, 3511, 4561, 4769, 7028, 7664, 7844, 8149, 8281-8289d, 8701, 8860, 9540, 10398, 10400, 10873, 10978, 11431, 11719, 12705, 13708, 14766, 14783, 15043, 15301, 15326, 15510, 15520, 15629, 15721, 16092, 16169, 16223, 16260, 16295, 16519
K38346	Tib_3	Lhasa	M8a	73, 263, 310, 489, 750, 1438, 2706, 3106N, 4314d, 4715, 4769, 6179, 7028, 7196A, 7861TC, 8453, 8584, 8684, 8701, 8860, 9540, 10398, 10400, 10873, 11719, 12705, 14470, 14766, 14783, 15043, 15047, 15301, 15326, 15487T, 15697, 16223, 16298, 16319
K38439	Tib_3	Lhasa	M9a1a1c1b1a	73, 263, 309+CC, 315+C, 489, 711, 750, 1041, 1438, 2706, 3106N, 3394, 4491, 4769, 5471, 5899+XC, 7028, 7142, 7697, 8701, 8860, 9242, 9540, 10398, 10400, 10873, 11719, 12705, 14308, 14417, 14766, 14783, 15043, 15301, 15326, 16223, 16234, 16292, 16305, 16316, 16362, 16519
K38253	Tib_3	Lhasa	M9a1b1c	73, 150, 152, 153, 263, 310, 489, 750, 1041, 1438, 2706, 3106N, 3394, 4491, 4769, 7028, 8701, 8860, 9540, 10398, 10400, 10454, 10873, 11719, 12362, 12705, 14308, 14766, 14783, 15043, 15301, 15326, 15671, 16223, 16234, 16256, 16288, 16362, 16519
K38454	Tib_3	Lhasa	G3a1a1	16T, 73, 143, 150, 263, 309+C, 315+C, 489, 709, 750, 1438, 2706, 3106N, 4674, 4769, 4833, 5108, 7028, 8701, 8860, 8861, 9540, 10398, 10400, 10873, 11719, 11914, 12705, 14569, 14766, 14783, 15043, 15301, 15326, 15746, 16215, 16223, 16274
K38820	Tib_4	Nyingchi	A	73, 152, 235, 263, 309+C, 315+C, 523-524d, 663, 750, 1438, 1736, 2706, 3106N, 3644, 4248, 4769, 4824, 7028, 7331A, 8794, 8860, 9063, 10275, 11719, 12705, 14766, 15326, 16223, 16271, 16274, 16290, 16319, 16362, 16527
K38108	Tib_4	Nyingchi	G3b3a	73, 152, 189, 263, 310, 489, 524+ACAC, 709, 750, 1438, 2706, 3106N, 3394, 4769, 4833, 5108, 7028, 8701, 8860, 9540, 10398, 10400, 10873, 11719, 12705, 13477, 14569, 14605, 14766, 14783, 15043, 15301, 15326, 15927, 16209, 16223, 16274, 16362
K38020	Tib_4	Nyingchi	M62b1b1	73, 150, 203, 204, 263, 309+CC, 315+C, 489, 524+ACAC, 750, 1438, 2706, 2735, 3030, 3106N, 3511, 4561, 4769, 7028, 7664, 7807, 8149, 8701, 8860, 9540, 10398, 10400, 10873, 11719, 12705, 13708, 14766, 14783, 15043, 15301, 15326, 15510, 15520, 15629, 15721, 16093, 16223, 16260, 16295, 16362, 16519
K38113	Tib_4	Nyingchi	M9a1b1	73, 150, 152, 153, 263, 310, 489, 750, 1041, 1438, 2706, 3106N, 3394, 4491, 4769, 7028, 8701, 8860, 9478, 9540, 10398, 10400, 10873, 11719, 12362, 12705, 13326, 14308, 14766, 14783, 15043, 15301, 15326, 15671, 16158, 16223, 16234, 16362, 16519
K38771	Tib_4	Nyingchi	A15c2	73, 152, 153, 235, 310, 523-524d, 663, 750, 1438, 1736, 2706, 3106N, 4248, 4769, 4824, 6458, 7028, 8459, 8794, 8860, 9052, 11719, 12705, 13111, 14067, 14766, 15262, 15326, 16223, 16290, 16319, 16362
K38206	Tib_4	Nyingchi	F1g	73, 249d, 263, 309+C, 315+C, 414G, 523-524d, 750, 1438, 2389, 2706, 3106N, 3398, 3970, 4769, 6392, 6962, 7028, 8860, 10310, 10609, 11719, 12406, 12882, 13928C, 14766, 15326, 16183C, 16189, 16304, 16519

K38174	Tib_4	Nyingchi	M9a1a1c1b1a	73, 263, 309+C, 315+C, 489, 711, 750, 1041, 1438, 2706, 3106N, 3394, 4491, 4769, 5899+XC, 7028, 7142, 7697, 8701, 8860, 9242, 9540, 10398, 10400, 10873, 11719, 12705, 13098, 14308, 14417, 14766, 14783, 15043, 15301, 15326, 16092, 16223, 16234, 16316, 16362
K38111	Tib_4	Nyingchi	A6	73, 152, 235, 263, 310, 523-524d, 654, 663, 750, 1438, 1736, 2706, 3106N, 3687, 4248, 4769, 4824, 7028, 8794, 8860, 9705, 11719, 12705, 13287, 14766, 15326, 16223, 16234, 16290, 16319, 16362
K38110	Tib_4	Nyingchi	B5b4	73, 103, 146, 204, 263, 309+C, 315+C, 523-524d, 709, 750, 1438, 1598, 2706, 3106N, 4703, 4769, 6852, 7028, 7170, 8281-8289d, 8349, 8584, 8829, 8860, 9950, 10398, 11719, 12361, 14766, 15223, 15326, 15508, 15662, 15851, 15927, 16140, 16182C, 16183C, 16189, 16243, 16288, 16519
K3151	Tib_4	Nyingchi	M9a1a1c1b1a	73, 263, 309+C, 315+C, 489, 711, 750, 1041, 1438, 2706, 3106N, 3394, 4077, 4491, 4769, 5899+XC, 7028, 7142, 7697, 8701, 8860, 9242, 9540, 10398, 10400, 10873, 11719, 12192, 12705, 14308, 14417, 14766, 14783, 15043, 15301, 15326, 16223, 16234, 16316, 16362
K36966	Tib_4	Nyingchi	C7	73, 195, 249d, 263, 310, 489, 523-524d, 750, 1438, 1719, 2706, 3106N, 3552A, 4715, 4769, 5821, 6338, 7028, 7196A, 7424, 8584, 8701, 8860, 9540, 9545, 9641, 10398, 10400, 10873, 11719, 11914, 12705, 12732, 13263, 14124, 14318, 14766, 14783, 15043, 15301, 15326, 15487T, 16093, 16223, 16266, 16298, 16327, 16356, 16519
K38546	Tib_4	Nyingchi	G2b1b	73, 263, 309+CC, 315+C, 489, 709, 750, 1438, 2706, 3106N, 4769, 4833, 4853, 5108, 5601, 5951, 7028, 8701, 8860, 8877, 9540, 10398, 10400, 10873, 11719, 12375, 12705, 13563, 14569, 14766, 14783, 15043, 15301, 15326, 16223, 16362
K38640	Tib_4	Nyingchi	G3b1a	73, 195, 263, 309+C, 315+C, 489, 523-524d, 709, 750, 1438, 2706, 3106N, 4769, 4833, 5108, 7028, 8065, 8701, 8860, 9540, 9599, 10398, 10400, 10873, 11719, 12705, 13477, 14569, 14605, 14766, 14783, 15043, 15301, 15326, 15927, 16223, 16249, 16274, 16362
K38428	Tib_4	Nyingchi	A8a	64, 73, 146, 151, 235, 263, 309+CC, 315+C, 523-524d, 663, 750, 1438, 1736, 2706, 3106N, 3144, 4248, 4508, 4769, 4824, 5046, 7028, 7711, 8794, 8860, 9300, 9531, 11719, 12705, 14766, 15326, 15758, 16223, 16264, 16290, 16319
K38967	Tib_4	Nyingchi	C7a	73, 249d, 263, 309+CC, 315+C, 489, 523-524d, 750, 1438, 2706, 3106N, 3552A, 4715, 4742, 4769, 5821, 6338, 7028, 7196A, 7853, 8584, 8701, 8860, 9073, 9540, 9545, 10398, 10400, 10873, 11719, 11914, 12705, 13263, 14318, 14766, 14783, 15043, 15301, 15326, 15487T, 16223, 16298, 16327
K38821	Tib_4	Nyingchi	D4h1c2a1	73, 151, 152, 263, 310, 489, 709, 750, 1438, 2706, 3010, 3106N, 3336, 3644, 4182, 4679, 4769, 4883, 5048, 5178A, 7028, 7181, 7673, 8179, 8414, 8701, 8860, 9540, 10398, 10400, 10873, 11719, 12477, 12705, 13914A, 14178, 14668, 14766, 14783, 15043, 15301, 15326, 16174, 16223, 16311, 16319, 16362, 16519
K38120	Tib_4	Nyingchi	F1d1a1	73, 146, 249d, 263, 309+C, 315+C, 523-524d, 750, 1438, 1734, 2706, 2833, 3106N, 3970, 4769, 5628, 6392, 6962, 7028, 7738, 8860, 10310, 10609, 11719, 12406, 12882, 13135, 13928C, 14766, 15326, 15402, 16145, 16284, 16304, 16474C, 16519
K38040	Tib_4	Nyingchi	M12a1b	73, 125G, 127, 128, 146, 195, 263, 309+CC, 315+C, 489, 750, 979, 1438, 2706, 3106N, 3621, 4170, 4562, 4769, 5492, 5580, 6446, 7028, 8701, 8860, 9540, 9755, 10398, 10400, 10873, 11016, 11025, 11353, 11719, 12030, 12358, 12372, 12616, 12705, 14569, 14727, 14766, 14783, 15010, 15043, 15301, 15326, 15424, 15894, 16148, 16172, 16223, 16234, 16261, 16290, 16519
K38427	Tib_4	Nyingchi	M7c1a1a1	64, 73, 146, 199, 263, 310, 489, 523-524d, 750, 1438, 2706, 3106N, 3882, 4071, 4769, 4850, 5442, 6455, 7028, 8701, 8860, 9266, 9540, 9824, 10398, 10400, 10873, 11665, 11719, 12091, 12372, 12705, 12810, 13759, 13942, 14766, 14783, 15043, 15301, 15326, 16134, 16169, 16223, 16295, 16519
K36969	Tib_4	Nyingchi	A17	73, 152, 234, 235, 263, 310, 523-524d, 663, 750, 1438, 1736, 2706, 3106N, 4113, 4248, 4769, 4824, 5514, 7028, 7805, 8794, 8860, 9126, 11252, 11719, 12408, 12705, 14766, 15217, 15326, 16223, 16235, 16274, 16290, 16299, 16311, 16319, 16362, 16519
K38096	Tib_4	Nyingchi	B4a4	73, 189, 193, 263, 309+CC, 315+C, 523-524d, 709, 750, 1438, 2706, 3106N, 4769, 5465, 5498, 7028, 8281-8289d, 8860, 9123, 11719, 13834, 14751, 14766, 15326, 16182C, 16183C, 16189, 16217, 16299, 16357, 16390, 16519
K38483	Tib_4	Nyingchi	C4d2	73, 152, 195, 249d, 263, 310, 489, 750, 1438, 2706, 3106N, 3552A, 4715, 4769, 6026, 7028, 7100, 7196A, 8584, 8701, 8860, 9540, 9545, 10398, 10400, 10873, 11719, 11914, 11969, 12705, 12780, 13263, 14318, 14766, 14783, 15043, 15204, 15236, 15301, 15326, 15487T, 16093, 16298, 16327, 16519
K38093	Tib_4	Nyingchi	G2a1	73, 263, 309+CC, 315+C, 489, 709, 750, 1438, 2706, 3106N, 4769, 4833, 5108, 5601, 7028, 7298, 7600, 8701, 8860, 9377, 9540, 9575, 10398, 10400, 10873, 11719, 12705, 13563, 14200, 14569, 14766, 14783, 15043, 15301, 15326, 16223, 16227, 16278, 16362
K38011	Tib_4	Nyingchi	M9a1b1c	73, 150, 152, 153, 263, 310, 489, 750, 1041, 1438, 2706, 3106N, 3394, 4491, 4769, 7028, 8701, 8860, 9540, 10398, 10400, 10454, 10873, 11719, 12362, 12705, 14308, 14766, 14783, 15043, 15301, 15326, 15671, 16158, 16223, 16234, 16355, 16362, 16519
K38378	Tib_4	Nyingchi	A11a1a	73, 152, 235, 263, 309+C, 315+C, 523-524d, 663, 750, 1005, 1438, 1736, 2706, 3106N, 3918, 4248, 4769, 4824, 5768, 5899+XC, 6755, 7028, 8794, 8843, 8860, 9650, 11719, 12705, 14766, 15326, 16223, 16290, 16293C, 16319

K38395	Tib_4	Nyingchi	A14b1	73, 151, 152, 200, 235, 263, 310, 523-524d, 663, 735, 750, 1438, 1598, 1736, 2706, 3106N, 4248, 4769, 4824, 7028, 7805, 8794, 8860, 11719, 11914, 12705, 14766, 15326, 16223, 16290, 16319, 16362
K38225	Tib_4	Nyingchi	A21a	73, 152, 235, 263, 310, 663, 750, 1438, 1736, 2706, 3106N, 4248, 4769, 4824, 7028, 8794, 8860, 11719, 12705, 14364, 14766, 15326, 16092, 16223, 16290, 16319, 16362
K38268	Tib_4	Nyingchi	F1c1a1a	73, 152, 249d, 263, 309+C, 315+C, 523-524d, 573+XC, 709, 750, 1438, 2706, 3106N, 3970, 4769, 6392, 6599, 6962, 7028, 8860, 9053, 9647, 10310, 10454, 10609, 11719, 12406, 12882, 13759, 13928C, 14110, 14766, 15326, 16111, 16129, 16266, 16304, 16519
K38396	Tib_4	Nyingchi	G2b1b2a	73, 263, 309+C, 315+C, 489, 523-524d, 709, 750, 1438, 2706, 3106N, 3975, 4769, 4833, 4853, 5108, 5601, 7028, 8701, 8860, 8877, 9540, 10398, 10400, 10873, 11719, 12375, 12705, 12795, 13563, 14209, 14563G, 14569, 14766, 14783, 15043, 15301, 15326, 16223, 16324, 16362
K38333	Tib_4	Nyingchi	G3b3	73, 152, 263, 310, 489, 709, 750, 1438, 2706, 3106N, 3394, 4769, 4833, 5108, 7028, 8701, 8860, 9540, 10398, 10400, 10873, 11620, 11719, 11908, 12705, 13477, 14564, 14569, 14605, 14766, 14783, 15043, 15301, 15326, 15927, 16209, 16223, 16274, 16362
K38388	Tib_4	Nyingchi	G3b1a1	73, 195, 263, 309+C, 315+C, 489, 523-524d, 709, 750, 1438, 2706, 3106N, 4769, 4833, 5108, 7028, 8701, 8860, 9540, 9599, 10398, 10400, 10511, 10873, 11719, 12616, 12705, 13477, 14569, 14605, 14766, 14783, 15043, 15301, 15326, 15927, 16223, 16249, 16274, 16362
K38397	Tib_4	Nyingchi	M5a	73, 263, 310, 489, 709, 750, 1438, 1888, 2706, 3106N, 3921, 4769, 7028, 8701, 8860, 9540, 9773, 9947, 10398, 10400, 10790, 10873, 11719, 12477, 12705, 12727, 13708, 14323, 14766, 14783, 15043, 15301, 15326, 15927, 16129, 16183C, 16189, 16193+C, 16209, 16223, 16362, 16519
K38400	Tib_4	Nyingchi	M9a1b1e	73, 150, 152, 153, 263, 309+CC, 315+C, 489, 709, 750, 1041, 1438, 2706, 3106N, 3394, 4491, 4769, 7028, 8701, 8860, 9540, 10398, 10400, 10873, 11719, 12362, 12705, 14308, 14766, 14783, 15043, 15301, 15326, 15671, 16158, 16223, 16234, 16343, 16356, 16362, 16519
K38221	Tib_4	Nyingchi	A14b1	73, 152, 200, 235, 263, 310, 523-524d, 663, 735, 750, 1438, 1598, 1736, 2706, 3106N, 4248, 4769, 4824, 7028, 7705, 7805, 8794, 8860, 11719, 12705, 14766, 15326, 16223, 16290, 16319, 16362
K37573	Tib_4	Nyingchi	B5a1c2	73, 146, 210, 263, 310, 523-524d, 709, 750, 1438, 2706, 3106N, 3537, 4562, 4769, 6960, 7028, 8281-8289d, 8584, 8860, 9950, 10325, 10398, 11719, 14766, 15235, 15326, 15514, 16086, 16111, 16140, 16183C, 16189, 16193+CC, 16266A, 16465, 16519
K38628	Tib_4	Nyingchi	F1b1c	73, 152, 249d, 263, 309+CC, 315+C, 523-524d, 750, 1438, 2706, 3106N, 3970, 4732, 4769, 5147, 5508, 6392, 6962, 7028, 8860, 8955, 9932, 10235, 10310, 10609, 10976, 11719, 12406, 12882, 13928C, 14476, 14766, 15119, 15326, 16182C, 16183C, 16189, 16209, 16232A, 16249, 16304, 16519
K38877	Tib_4	Nyingchi	A11a1a4	73, 152, 235, 263, 309+CC, 315+C, 523-524d, 663, 750, 1005, 1438, 1736, 2706, 3106N, 4248, 4769, 4824, 5899+XC, 6755, 7028, 8794, 8843, 8860, 9055, 9650, 11719, 12705, 14766, 15326, 16223, 16290, 16293C, 16319
K38358	Tib_4	Nyingchi	A11a1a	73, 152, 235, 263, 309+C, 315+C, 523-524d, 663, 750, 1005, 1438, 1736, 1810, 2706, 3106N, 4248, 4316, 4769, 4824, 5899+XC, 6755, 7028, 8794, 8843, 8860, 9650, 11719, 12705, 14766, 15326, 16223, 16290, 16293C, 16319
K38405	Tib_4	Nyingchi	A17	73, 152, 234, 235, 263, 310, 523-524d, 663, 750, 1438, 1736, 2706, 3106N, 4113, 4248, 4769, 4824, 5514, 7028, 7805, 8794, 8860, 9126, 11252, 11719, 12408, 12705, 14766, 15217, 15326, 16223, 16235, 16274, 16290, 16299, 16311, 16319, 16362, 16519
K38973	Tib_4	Nyingchi	C4a1a1a1	73, 249d, 263, 310, 489, 523-524d, 750, 1438, 1715, 2232+AA, 2706, 3106N, 3327, 3552A, 3576, 4715, 4769, 4884, 4958, 6026, 7028, 7196A, 7999, 8508, 8584, 8701, 8860, 9540, 9545, 10398, 10400, 10873, 11719, 11914, 11969, 12672, 12705, 13263, 14318, 14766, 14783, 15043, 15204, 15301, 15326, 15487T, 15968, 16093, 16129, 16223, 16224, 16298, 16327, 16519
K38878	Tib_4	Nyingchi	C4a2c1	47, 73, 207, 249d, 263, 309+C, 315+C, 489, 750, 1438, 2232+A, 2706, 3106N, 3394, 3552A, 4715, 4769, 6026, 6929, 7028, 7196A, 8584, 8701, 8860, 9540, 9545, 10398, 10400, 10873, 11719, 11914, 11969, 12672, 12705, 13263, 13967, 14318, 14766, 14783, 15043, 15204, 15301, 15326, 15487T, 16223, 16239, 16327, 16357, 16519
K38873	Tib_4	Nyingchi	C4d2	73, 152, 249d, 263, 309+C, 315+C, 489, 750, 1438, 2706, 3106N, 3552A, 4715, 4769, 6026, 7028, 7100, 7196A, 8584, 8701, 8860, 9540, 9545, 10398, 10400, 10873, 11719, 11914, 11969, 12705, 12780, 13263, 14318, 14766, 14783, 15043, 15204, 15236, 15301, 15326, 15487T, 16093, 16298, 16327, 16519
K38786	Tib_4	Nyingchi	D4b2b	73, 263, 310, 489, 523-524d, 750, 1382C, 1438, 2706, 3010, 3106N, 4769, 4883, 5178A, 6701, 7028, 8020, 8414, 8701, 8860, 8964, 9296, 9540, 9824A, 10398, 10400, 10873, 11719, 12705, 14002, 14668, 14766, 14783, 15043, 15301, 15326, 16223, 16362, 16519
K38743	Tib_4	Nyingchi	D4b2b9	73, 194, 263, 310, 489, 523-524d, 750, 1382C, 1438, 2706, 3010, 3106N, 4769, 4883, 5178A, 7028, 8020, 8414, 8545, 8701, 8860, 8964, 9296, 9540, 9824A, 10398, 10400, 10873, 11719, 12705, 14668, 14751, 14766, 14783, 15043, 15301, 15326, 16111, 16223, 16362, 16519

K38892	Tib_4	Nyingchi	D4g2a1c1	73, 263, 298, 310, 489, 750, 827, 1438, 2706, 3010, 3106N, 4394, 4769, 4883, 5178A, 5231, 7028, 8414, 8701, 8860, 9540, 10398, 10400, 10873, 11059, 11719, 12231, 12705, 12966A, 13104, 14668, 14766, 14783, 15043, 15301, 15326, 16223, 16240, 16264, 16274, 16362, 16519
K38484	Tib_4	Nyingchi	D4h1c2a1	73, 151, 152, 263, 309+C, 315+C, 489, 709, 750, 1438, 2706, 3010, 3106N, 3336, 3644, 4182, 4769, 4883, 5048, 5178A, 7028, 7181, 7673, 8179, 8414, 8701, 8860, 9540, 10398, 10400, 10873, 11719, 12477, 12705, 13914A, 14178, 14668, 14766, 14783, 15043, 15301, 15326, 16174, 16223, 16311, 16319, 16362, 16519
K38478	Tib_4	Nyingchi	D4h1c2a1	73, 151, 152, 263, 309+C, 315+C, 489, 709, 750, 1438, 2706, 3010, 3106N, 3336, 3644, 4182, 4769, 4883, 5048, 5178A, 7028, 7181, 7673, 8179, 8414, 8701, 8860, 9540, 10398, 10400, 10873, 11719, 12477, 12705, 13914A, 14178, 14668, 14766, 14783, 15043, 15301, 15326, 16174, 16223, 16311, 16319, 16362, 16519
K38959	Tib_4	Nyingchi	D4j1b	73, 263, 310, 489, 750, 1438, 1734, 2706, 3010, 3106N, 4769, 4883, 5178A, 5262, 6286, 7028, 7055, 8194, 8414, 8701, 8860, 9540, 10253, 10398, 10400, 10873, 11696, 11719, 12705, 12993, 14668, 14766, 14783, 15043, 15301, 15326, 16223, 16362
K38876	Tib_4	Nyingchi	D4j3b	73, 263, 309+C, 315+C, 338, 489, 750, 1438, 2706, 3010, 3106N, 4769, 4883, 5178A, 7028, 8414, 8701, 8860, 9540, 10398, 10400, 10873, 11696, 11719, 12164, 12705, 14668, 14766, 14783, 15043, 15301, 15326, 15519, 16184, 16223, 16260, 16311, 16362, 16519
K38208	Tib_4	Nyingchi	D4j3b	73, 195, 263, 309+C, 315+C, 338, 489, 750, 789, 1438, 2706, 3010, 3106N, 4769, 4883, 5178A, 7028, 8414, 8701, 8860, 9540, 10398, 10400, 10873, 11696, 11719, 12705, 14668, 14766, 14783, 15043, 15301, 15326, 16157, 16184, 16223, 16311, 16362
K38675	Tib_4	Nyingchi	D5a2a1c	73, 150, 263, 310, 489, 523-524d, 750, 752, 1107, 2706, 3106N, 4769, 4883, 5178A, 5301, 7028, 8701, 8860, 9180, 9540, 10397, 10398, 10400, 10873, 11719, 11944, 12026, 12705, 14766, 14783, 15043, 15301, 15326, 16092, 16164, 16172, 16182C, 16183C, 16189, 16223, 16243, 16266, 16362
K38823	Tib_4	Nyingchi	F1a1a1	73, 249d, 263, 309+C, 315+C, 523-524d, 750, 1438, 2706, 3106N, 3970, 4086, 4769, 6392, 6962, 7028, 8149, 8479, 8860, 9053, 9548, 10310, 10609, 11215, 11719, 11914, 12406, 12882, 13759, 13928C, 14766, 15326, 16108, 16129, 16162, 16172, 16304, 16317, 16519
K38406	Tib_4	Nyingchi	F1c1a1a	73, 151, 152, 249d, 263, 309+CC, 315+C, 523-524d, 573+XC, 709, 750, 1438, 2706, 3106N, 3970, 4769, 6392, 6599, 6962, 7028, 8860, 9053, 9647, 10310, 10454, 10609, 11719, 12406, 12882, 13759, 13928C, 14766, 15326, 16111, 16129, 16188+CC, 16266, 16304, 16519
K38477	Tib_4	Nyingchi	F1d	73, 146, 249d, 263, 309+C, 315+C, 523-524d, 750, 1438, 1734, 2706, 3106N, 3970, 4769, 5460, 5628, 6050, 6392, 6962, 7028, 7193, 7738, 8860, 10306, 10310, 10609, 11092, 11719, 12406, 12882, 13928C, 14766, 15326, 15402, 15930, 16042GA, 16093, 16304, 16519
K38583	Tib_4	Nyingchi	F1g1	73, 249d, 263, 309+CC, 315+C, 523-524d, 750, 1438, 2389, 2706, 3106N, 3398, 3621, 3970, 4769, 6351, 6392, 6962, 7028, 8860, 9180, 10310, 10609, 11335, 11719, 12406, 12882, 13928C, 14766, 14798, 15326, 15499, 16093, 16182C, 16183C, 16189, 16304, 16519
K38748	Tib_4	Nyingchi	G2a'c	73, 151, 152, 263, 310, 489, 709, 750, 1438, 2706, 3106N, 4769, 4833, 5108, 5493, 5601, 5783, 7028, , 8701, 8860, 9230, 9540, 9575, 10398, 10400, 10873, 11084, 11719, 12705, 13542C, 13563, 14569, 14766, 14783, 15043, 15262, 15301, 15326, 16223, 16291, 16311, 16362
K38349	Tib_4	Nyingchi	H101	152, 263, 309+CC, 315+C, 721, 750, 1438, 3106N, 4769, 8860, 9230, 15326, 16184
K38995	Tib_4	Nyingchi	M13a2a1e	73, 152, 263, 310, 489, 513, 750, 1438, 2706, 2786, 3106N, 3644, 4769, 5773, 6023, 6253, 6620, 7028, 8405, 8701, 8860, 9540, 10373, 10398, 10400, 10411, 10790, 10873, 11719, 12705, 13395, 13981, 14766, 14783, 14824, 15043, 15301, 15326, 15924, 15944d, 16145, 16168, 16188, 16223, 16257, 16311, 16519
K38895	Tib_4	Nyingchi	M9a1a1c1b1a14	73, 152, 263, 309+CC, 315+C, 489, 711, 750, 1041, 1438, 2706, 3106N, 3394, 4491, 4769, 5899+XC, 7028, 7142, 7697, 8701, 8860, 9242, 9540, 10398, 10400, 10873, 11719, 12705, 14308, 14417, 14766, 14783, 15043, 15301, 15326, 16223, 16234, 16316, 16362
K38860	Tib_4	Nyingchi	M9a1a1c1b1a	73, 263, 309+C, 315+C, 453, 489, 711, 750, 1041, 1438, 2706, 3106N, 3394, 4491, 4769, 5899+XC, 5911, 7028, 7142, 7697, 8701, 8860, 9242, 9540, 10398, 10400, 10873, 11719, 12705, 14308, 14417, 14766, 14783, 15043, 15301, 15326, 16223, 16234, 16316, 16362
K38721	Tib_4	Nyingchi	M9a1a1c1b1a	73, 263, 291+A, 309+CC, 315+C, 489, 711, 750, 1041, 1438, 2706, 3106N, 3394, 4491, 4769, 5899+XC, 7028, 7142, 7697, 8701, 8860, 9242, 9540, 10398, 10400, 10873, 11719, 12705, 14308, 14417, 14766, 14783, 15043, 15301, 15326, 16223, 16234, 16316, 16362
K38295	Tib_4	Nyingchi	M9a1a1c1b1a	73, 263, 309+C, 315+C, 489, 711, 750, 1041, 1438, 2706, 3106N, 3394, 4491, 4769, 5899+XC, 7028, 7142, 7697, 8701, 8860, 9242, 9540, 10205, 10398, 10400, 10873, 11719, 12705, 14308, 14417, 14766, 14783, 15043, 15301, 15326, 16223, 16234, 16241, 16316, 16362
K38437	Tib_4	Nyingchi	M9a1a1c1b1a	73, 263, 309+CC, 315+C, 489, 711, 750, 1041, 1438, 2706, 3106N, 3394, 4491, 4769, 5471, 5899+XC, 7028, 7142, 7697, 8701, 8860, 9242, 9540, 10398, 10400, 10873, 11719, 12705, 14308, 14417, 14766, 14783, 15043, 15301, 15326, 16223, 16234, 16292, 16305, 16316, 16362, 16519
K39022	Tib_4	Nyingchi	C4a1a1a3	73, 179, 249d, 263, 310, 489, 523-524d, 750, 1438, 1715, 2232+AA, 2706, 3106N, 3552A, 3576, 4715, 4769, 4884, 4958, 6026, 7028, 7196A, 7999, 8508, 8584, 8701, 8860, 9540, 9545, 10398, 10400, 10873, 11719, 11914, 11969, 12672, 12705, 13263, 14318, 14766, 14783, 15043, 15204, 15301, 15326, 15487T, 15968, 16129, 16223, 16298, 16327, 16519

K38720	Tib_4	Nyingchi	M9a1a1c1b1a	73, 263, 291+A, 309+CC, 315+C, 489, 711, 750, 1041, 1438, 2706, 3106N, 3394, 4491, 4769, 5899+XC, 7028, 7142, 7697, 8701, 8860, 9242, 9540, 10398, 10400, 10873, 11719, 12705, 14308, 14417, 14766, 14783, 15043, 15301, 15326, 16223, 16234, 16316, 16362
K38643	Tib_4	Nyingchi	G2b1b2a	73, 263, 309+C, 315+C, 489, 709, 750, 1438, 2706, 3106N, 3975, 4769, 4833, 4853, 5108, 5601, 7028, 7202, 8701, 8860, 8877, 9540, 10398, 10400, 10873, , 11719, 12375, 12705, 12795, 13563, 14563G, 14569, 14766, 14783, 15043, 15301, 15326, 16223, 16324, 16362
K38637	Tib_4	Nyingchi	D5a2a1d	73, 150, 263, 309+C, 315+C, 489, 523-524d, 650, 750, 752, 1107, 2706, 3106N, 3402, 4083, 4769, 4883, 5178A, 5301, 5581, 7028, 8701, 8860, 9180, 9540, 10397, 10398, 10400, 10873, 11152, 11719, 11944, 12026, 12358, 12705, 13711, 13985-13987d, 14766, 14783, 15043, 15301, 15326, 16164, 16172, 16182C, 16183C, 16189, 16209, 16223, 16266, 16362
K38548	Tib_4	Nyingchi	F1g	73, 249d, 263, 309+CC, 315+C, 523-524d, 750, 1438, 2389, 2706, 3106N, 3398, 3421, 3970, 4769, 6023, 6392, 6962, 7028, 8860, 10310, 10609, 11719, 12406, 12882, 13928C, 14766, 15326, 16182C, 16183C, 16189, 16304, 16519
K38511	Tib_4	Nyingchi	D5a2a1c	73, 150, 263, 310, 489, 523-524d, 750, 752, 1107, 2706, 2833, 3106N, 4769, 4883, 5178A, 5301, 7028, 8701, 8860, 9180, 9540, 10397, 10398, 10400, 10873, 11719, 11944, 12026, 12705, 14766, 14783, 15043, 15301, 15326, 16164, 16172, 16182C, 16183C, 16189, 16223, 16243, 16266, 16362
K38443	Tib_4	Nyingchi	F1g	73, 249d, 263, 309+CC, 315+C, 523-524d, 750, 1438, 2389, 2706, 3106N, 3398, 3630, 3970, 4769, 6392, 6962, 7028, 8860, 10310, 10609, 11719, 12406, 12882, 13840, 13928C, 14766, 15326, 16182C, 16183C, 16189, 16304, 16519
K38062	Tib_4	Nyingchi	D4b2b10	41, 73, 194, 263, 310, 489, 523-524d, 750, 1382C, 1438, 2706, 3010, 3106N, 4769, 4883, 5178A, 7028, 8020, 8065, 8291, 8414, 8701, 8784, 8860, 8964, 9296, 9540, 9824A, 10398, 10400, 10873, 11719, 12705, 14668, 14766, 14783, 15043, 15217, 15301, 15326, 16223, 16362, 16519
K1998	Tib_5	Anduo, Naqu	D4j1a1f	73, 263, 309+C, 315+C, 489, 523-524d, 750, 1438, 2706, 3010, 3106N, 4769, 4883, 5178A, 5262, 7028, 7581, 7783, 8414, 8701, 8860, 9053, 9540, 10398, 10400, 10873, 11696, 11719, 12130, 12358, 12705, 14668, 14766, 14783, 15043, 15295, 15301, 15326, 16086, 16223, 16362
K1823	Tib_5	Anduo, Naqu	M9a1a1c1b1a	73, 263, 310, 489, 711, 750, 1041, 1438, 2706, 3106N, 3394, 4491, 4769, 5899+XC, 7028, 7142, 7697, 8701, 8860, 9242, 9540, 10398, 10400, 10873, 11719, 12705, 14308, 14417, 14766, 14783, 15043, 15301, 15326, 16223, 16234, 16316, 16362
K2007	Tib_5	Anduo, Naqu	B4a4	73, 193, 263, 309+C, 315+C, 523-524d, 709, 750, 1438, 2706, 3106N, 4769, 5465, 5498, 7028, 7830, 8281-8289d, 8860, 9123, 11719, 13834, 14751, 14766, 15262, 15326, 16182C, 16183C, 16189, 16217, 16299, 16519
K1816	Tib_5	Anduo, Naqu	D4j3b	73, 263, 309+C, 315+C, 338, 489, 750, 1438, 2706, 3010, 3106N, 4769, 4883, 5178A, 7028, 8414, 8701, 8860, 9540, 10398, 10400, 10873, 11696, 11719, 12705, 14668, 14766, 14783, 15043, 15301, 15326, 16184, 16223, 16278, 16311, 16362
K1808	Tib_5	Anduo, Naqu	F1g	73, 249d, 263, 309+CC, 315+C, 523-524d, 750, 1438, 2389, 2706, 3106N, 3398, 3970, 4769, 6392, 6962, 7028, 8860, 10310, 10321, 10609, 11719, 12406, 12699, 12882, 12930, 13928C, 14766, 15326, 16182C, 16183C, 16189, 16304, 16519
K1862	Tib_5	Anduo, Naqu	D5a2a1c	73, 150, 263, 272, 310, 489, 523-524d, 750, 752, 1107, 2706, 3106N, 4769, 4883, 5178A, 5301, 7028, 8573, 8701, 8860, 9180, 9540, 10397, 10398, 10400, 10873, 11719, 11944, 12026, 12705, 13203, 14766, 14783, 15043, 15301, 15326, 16092, 16164, 16172, 16182C, 16183C, 16189, 16223, 16243, 16266, 16362
K1812	Tib_5	Anduo, Naqu	F1g	73, 249d, 263, 309+CC, 315+C, 523-524d, 750, 1438, 2389, 2706, 3106N, 3398, 3970, 4769, 6392, 6962, 7028, 8860, 10310, 10321, 10609, 11719, 12406, 12699, 12882, 12930, 13928C, 14766, 15326, 16182C, 16183C, 16189, 16304, 16519
K1844	Tib_5	Anduo, Naqu	M13a1b	73, 152, 263, 310, 489, 750, 1438, 2706, 3106N, 3644, 4769, 5773, 6023, 6253, 6620, 7028, 8392, 8701, 8860, 9540, 10398, 10400, 10411, 10790, 10873, 11719, 12705, 13135, 14766, 14783, 15043, 15301, 15326, 15924, 16145, 16148, 16188, 16189, 16223, 16381
K1864	Tib_5	Anduo, Naqu	M9a1a1c1b1a2	73, 146, 263, 309+C, 315+C, 489, 711, 750, 1041, 1438, 2706, 3106N, 3394, 4491, 4769, 5899+XC, 6446, 7028, 7142, 7598, 7697, 8701, 8860, 9242, 9540, 10398, 10400, 10873, 11719, 12705, 14308, 14417, 14766, 14783, 15043, 15301, 15326, 16223, 16234, 16311, 16316, 16362
K1468	Tib_5	Anduo, Naqu	M9a1b1c	73, 150, 152, 153, 263, 310, 489, 709, 750, 1041, 1438, 2706, 3106N, 3394, 4491, 4769, 7028, 8701, 8860, 9000, 9540, 10398, 10400, 10454, 10873, 11719, 12362, 12705, 14308, 14766, 14783, 15043, 15301, 15326, 15671, 16158, 16223, 16234, 16288, 16362, 16519
K1859	Tib_5	Anduo, Naqu	A	73, 152, 182, 235, 263, 309+CC, 315+C, 663, 750, 1438, 1736, 2706, 3106N, 4248, 4769, 4824, 7028, 8794, 8860, 11719, 12705, 13488, 14766, 15326, 16223, 16248, 16290, 16319, 16362
K1861	Tib_5	Anduo, Naqu	A11a1a	73, 152, 235, 263, 272, 309+C, 315+C, 523-524d, 663, 750, 1005, 1438, 1736, 2706, 3106N, 4248, 4769, 4824, 5503, 5899+XC, 6755, 7028, 8794, 8843, 8860, 9650, 11719, 12705, 14207, 14766, 15326, 16093, 16223, 16290, 16293C, 16319, 16519
K1677	Tib_5	Anduo, Naqu	D4b2b8	73, 194, 199, 227T, 263, 309+C, 315+C, 390, 489, 523-524d, 750, 1382C, 1438, 2706, 3010, 3106N, 3398, 4769, 4883, 5178A, 7028, 8020, 8414, 8701, 8860, 8964, 9296, 9540, 9824A, 10398, 10400, 10873, 11719, 12705, 14668, 14766, 14783, 15043, 15301, 15326, 15613, 16173, 16223, 16362, 16519

K1852	Tib_5	Anduo, Naqu	D4b2b10	40, 41, 73, 194, 199, 263, 309+CC, 315+C, 489, 523-524d, 750, 1382C, 1438, 2706, 3010, 3106N, 4769, 4883, 5178A, 7028, 8020, 8414, 8701, 8784, 8860, 8964, 9296, 9540, 9824A, 10398, 10400, 10873, 11719, 12705, 14668, 14766, 14783, 15043, 15301, 15326, 16223, 16243, 16362, 16519
K1867	Tib_5	Anduo, Naqu	D4g2a1c1	73, 263, 298, 309+C, 315+C, 489, 750, 1438, 2706, 3010, 3106N, 4394, 4769, 4883, 5178A, 5231, 7028, 8414, 8701, 8860, 9540, 10398, 10400, 10873, 11059, 11719, 12705, 12966A, 13104, 13215, 14668, 14766, 14783, 15043, 15301, 15326, 16223, 16240, 16274, 16362, 16519
K1828	Tib_5	Anduo, Naqu	D4j1a1e	73, 263, 310, 489, 750, 1438, 2706, 3010, 3106N, 4769, 4883, 5178A, 5262, 7028, 7581, 7783, 8414, 8701, 8860, 9540, 10398, 10400, 10873, 11696, 11719, 12130, 12358, 12705, 14668, 14766, 14783, 15043, 15295, 15301, 15326, 16086, 16223, 16362, 16399, 16519
K1989	Tib_5	Anduo, Naqu	D4j1a1f	73, 263, 310, 489, 750, 1438, 2706, 3010, 3106N, 4769, 4883, 5178A, 5262, 5563, 7028, 7581, 7783, 8414, 8701, 8860, 9053, 9540, 10398, 10400, 10873, 11696, 11719, 12130, 12358, 12705, 14668, 14766, 14783, 15043, 15295, 15301, 15326, 16086, 16223, 16362
K1865	Tib_5	Anduo, Naqu	D4j1a1d1	73, 263, 310, 489, 750, 1438, 2706, 3010, 3106N, 4769, 4883, 5178A, 5262, 7028, 7581, 7783, 8414, 8701, 8860, 9540, 10398, 10400, 10810, 10873, 11696, 11719, 12130, 12358, 12705, 14587, 14668, 14766, 14783, 15043, 15295, 15301, 15326, 16086, 16223, 16274, 16362, 16519
K1853	Tib_5	Anduo, Naqu	F1b1	73, 249d, 263, 310, 481, 523-524d, 750, 1438, 2706, 3106N, 3970, 4732, 4769, 5147, 6392, 6962, 7028, 8860, 10310, 10609, 10976, 11719, 12406, 12633, 12882, 13928C, 14476, 14766, 15326, 15725, 16183C, 16189, 16232A, 16249, 16304, 16311, 16362, 16519
K1821	Tib_5	Anduo, Naqu	F1d	73, 249d, 263, 310, 523-524d, 750, 1189, 1438, 1734, 2706, 3106N, 3970, 4769, 5460, 5628, 6392, 6962, 7028, 7738, 8860, 10310, 10609, 10653, 11092, 11719, 12406, 12882, 13928C, 14766, 15326, 15402, 16189A, 16304, 16519
K1857	Tib_5	Anduo, Naqu	F1d	73, 153, 195, 249d, 263, 309+C, 315+C, 523-524d, 750, 1189, 1438, 1734, 2706, 3106N, 3970, 4769, 5460, 5628, 6392, 6962, 7028, 7738, 8860, 10310, 10609, 11092, 11719, 12406, 12882, 13928C, 14750, 14766, 15326, 15402, 16140, 16188+CC, 16304, 16519
K1825	Tib_5	Anduo, Naqu	F1g	73, 249d, 263, 310, 523-524d, 750, 1040, 1438, 2389, 2706, 3106N, 3398, 3970, 4769, 6392, 6962, 7028, 8658, 8860, 10310, 10609, 11719, 12406, 12882, 13928C, 14766, 15326, 16183C, 16189, 16304, 16519
K1326	Tib_5	Anduo, Naqu	G1a	73, 263, 310, 489, 709, 750, 1438, 2706, 2772, 3106N, 4769, 4833, 5108, 7028, 7867, 8200, 8701, 8860, 9540, 10398, 10400, 10873, 11719, 12705, 14180, 14569, 14766, 14783, 15043, 15301, 15323, 15326, 15497, 16223, 16362, 16519
K1842	Tib_5	Anduo, Naqu	H7b2	263, 309+C, 315+C, 750, 1438, 3106N, 4769, 4793, 5348, 8860, 13095, 13323, 15326, 16519
K2004	Tib_5	Anduo, Naqu	M11a2a1a	73, 146, 200, 263, 309+C, 315+C, 318, 326, 489, 750, 1095, 1438, 2706, 3106N, 4769, 6531, 7028, 7642, 8108, 8701, 8860, 9540, 9950, 10398, 10400, 10873, 11404, 11719, 11969, 12705, 13074, 14340, 14766, 14783, 15043, 15301, 15326, 16173, 16223
K1557	Tib_5	Anduo, Naqu	M13a2a1e	73, 152, 263, 310, 489, 513, 750, 1438, 2706, 2786, 3106N, 3644, 4769, 5773, 6023, 6253, 6620, 7028, 8405, 8701, 8860, 9540, 10373, 10398, 10400, 10411, 10790, 10873, 11719, 12705, 13395, 13981, 14766, 14783, 15043, 15301, 15326, 15924, 15944d, 16145, 16168, 16188, 16223, 16257, 16311, 16519
K1822	Tib_5	Anduo, Naqu	M61	73, 146, 152, 195, 263, 310, 489, 710, 750, 1193, 1342, 1438, 2706, 3106N, 4769, 5582C, 6253, 7028, 8269, 8701, 8860, 9540, 10398, 10400, 10873, 11719, 11810, 12705, 12732, 13356, 14766, 14783, 15043, 15301, 15326, 16176, 16223, 16260, 16270, 16362, 16381, 16519
K1804	Tib_5	Anduo, Naqu	M62a1a1	73, 143, 146, 150, 263, 489, 750, 1438, 2706, 2735, 3106N, 3511, 4561, 4763, 4769, 7028, 7664, 8149, 8701, 8860, 9540, 9935, 10398, 10400, 10873, 11719, 12223, 12245, 12705, 13708, 14766, 14783, 15043, 15301, 15326, 15510, 15520, 15629, 15721, 16147, 16189, 16193+CC, 16223, 16295, 16452, 16487, 16519
K1811	Tib_5	Anduo, Naqu	M7b1a1a3	73, 150, 199, 204, 207, 263, 310, 489, 750, 1438, 2706, 3106N, 4048, 4071, 4164, 4679, 4769, 5351, 5460, 6455, 6680, 7028, 7684, 7853, 8701, 8860, 9540, 9824, 10398, 10400, 10873, 11719, 12405, 12705, 12811, 14766, 14783, 14978, 15043, 15301, 15326, 16129, 16223, 16248, 16297
K1799	Tib_5	Anduo, Naqu	M9a1a1c1b1a	73, 263, 309+C, 315+C, 489, 711, 750, 1041, 1438, 2404, 2706, 3106N, 3394, 4491, 4769, 5306, 5899+XC, 7028, 7142, 7697, 8701, 8860, 9242, 9540, 10398, 10400, 10873, 11719, 12705, 14308, 14417, 14766, 14783, 15043, 15301, 15326, 16223, 16234, 16316, 16362
K1802	Tib_5	Anduo, Naqu	M9a1a2a1a	73, 153, 263, 310, 489, 513, 523-524d, 750, 1041, 1438, 2706, 3106N, 3394, 4491, 4769, 6512, 7028, 7256, 8485, 8701, 8860, 9540, 10398, 10400, 10873, 11719, 12705, 14308, 14766, 14783, 15043, 15301, 15326, 16145, 16223, 16234, 16316
K1834	Tib_5	Anduo, Naqu	M9a1b1e	73, 150, 152, 153, 263, 310, 489, 709, 750, 1041, 1438, 2706, 3106N, 3394, 4491, 4682, 4769, 7028, 8701, 8860, 9540, 10398, 10400, 10873, 11719, 12362, 12705, 14308, 14766, 14783, 15043, 15301, 15326, 15671, 16093, 16158, 16223, 16234, 16362, 16519
K1836	Tib_5	Anduo, Naqu	U2e1h	73, 217, 228, 263, 309+CC, 315+C, 340, 508, 750, 1438, 1811, 2706, 3106N, 3720, 4769, 5390, 5426, 6045, 6152, 7028, 8860, 10876, 11467, 11719, 12308, 12372, 13020, 13734, 14766, 15326, 15907, 16051, 16129C, 16183C, 16193+C, 16362, 16519

K1800	Tib_5	Anduo, Naqu	D4j1a1e	73, 263, 310, 489, 750, 1438, 2706, 3010, 3106N, 4769, 4883, 5178A, 5262, 7028, 7581, 7783, 8414, 8701, 8860, 9540, 10398, 10400, 10873, 11696, 11719, 12130, 12358, 12705, 14668, 14766, 14783, 15043, 15295, 15301, 15326, 16086, 16223, 16362, 16399, 16519
K1796	Tib_5	Anduo, Naqu	F1g	73, 249d, 263, 309+CC, 315+C, 523-524d, 750, 1438, 2389, 2706, 3106N, 3398, 3970, 4769, 6392, 6962, 7028, 8860, 10310, 10321, 10609, 11719, 12406, 12699, 12882, 12930, 13928C, 14766, 15326, 16182C, 16183C, 16189, 16304, 16519
K1863	Tib_5	Anduo, Naqu	A	73, 152, 235, 263, 309+CC, 315+C, 523-524d, 576, 663, 750, 1438, 1736, 2706, 3106N, 4232, 4248, 4769, 4824, 7028, 8794, 8860, 11719, 12705, 14384, 14766, 15326, 16125, 16223, 16290, 16311, 16319, 16362
K1833	Tib_5	Anduo, Naqu	A11a1a	73, 152, 235, 263, 309+C, 315+C, 523-524d, 663, 750, 1005, 1438, 1736, 1810, 2706, 3106N, 4248, 4769, 4824, 5899+XC, 6755, 7028, 8794, 8843, 8860, 9650, 11719, 12705, 14766, 15326, 16093, 16223, 16290, 16293C, 16319
K1817	Tib_5	Anduo, Naqu	D4j3b	73, 263, 309+C, 315+C, 338, 489, 750, 1438, 2706, 3010, 3106N, 4769, 4883, 5178A, 7028, 8414, 8701, 8860, 9540, 10398, 10400, 10873, 11696, 11719, 12705, 14668, 14766, 14783, 15043, 15301, 15326, 16184, 16223, 16278, 16311, 16362
K1846	Tib_5	Anduo, Naqu	F1g	73, 249d, 263, 309+C, 315+C, 523-524d, 750, 1438, 2389, 2706, 3106N, 3398, 3970, 4769, 6392, 6962, 7028, 8658, 8860, 10310, 10609, 11719, 12406, 12882, 13928C, 14766, 15326, 16183C, 16189, 16304, 16519
K1813	Tib_5	Anduo, Naqu	F1g	73, 249d, 263, 309+CC, 315+C, 523-524d, 750, 1438, 2389, 2706, 3106N, 3398, 3970, 4769, 6392, 6962, 7028, 8860, 10310, 10321, 10609, 11719, 12406, 12699, 12882, 12930, 13928C, 14766, 15326, 16182C, 16183C, 16189, 16304, 16519
K1809	Tib_5	Anduo, Naqu	F1g	73, 249d, 263, 310, 523-524d, 750, 1040, 1438, 2389, 2706, 3106N, 3398, 3970, 4769, 6392, 6962, 7028, 8658, 8860, 10310, 10609, 11719, 12406, 12882, 13928C, 14766, 15326, 16183C, 16189, 16304, 16519
K2011	Tib_5	Anduo, Naqu	G3a2	73, 143, 152, 195, 309+CC, 315+C, 489, 709, 750, 1438, 2706, 3106N, 4769, 4833, 5108, 7028, 7621, 8701, 8860, 9540, 10398, 10400, 10873, 11719, 12705, 14569, 14766, 14783, 15043, 15301, 15326, 15479, 15746, 16186, 16223, 16274, 16362
K1845	Tib_5	Anduo, Naqu	M11a2a1a	73, 146, 200, 263, 310, 318, 326, 489, 750, 1095, 1438, 2706, 3106N, 4769, 6531, 7028, 7642, 8108, 8701, 8860, 9540, 9950, 10398, 10400, 10873, 11404, 11719, 11969, 12705, 13074, 14340, 14766, 14783, 15043, 15301, 15326, 16173, 16223
K1843	Tib_5	Anduo, Naqu	M9a1a1c1b1a7	73, 263, 309+CC, 315+C, 489, 711, 750, 1041, 1438, 2706, 3106N, 3394, 4491, 4769, 5899+XC, 7028, 7142, 7697, 8701, 8860, 9242, 9540, 10398, 10400, 10873, 11719, 11887, 12705, 14308, 14417, 14766, 14783, 15043, 15301, 15326, 16223, 16234, 16316, 16362
K1798	Tib_5	Anduo, Naqu	M9a1a1c1b1a	73, 150, 195, 263, 309+C, 315+C, 489, 711, 750, 1041, 1438, 2706, 3106N, 3394, 4491, 4769, 5899+XC, 7028, 7142, 7604, 7697, 8152, 8701, 8860, 8998, 9242, 9540, 10398, 10400, 10873, 11719, 12705, 14308, 14417, 14766, 14783, 15043, 15301, 15326, 16223, 16234, 16243, 16311, 16316, 16362
K1797	Tib_5	Anduo, Naqu	M9a1a1c1b1a	73, 263, 309+C, 315+C, 489, 711, 750, 1041, 1438, 2706, 3106N, 3394, 4491, 4769, 5899+XC, 6293, 7028, 7142, 7697, 8701, 8860, 9242, 9540, 10398, 10400, 10873, 11719, 12705, 14308, 14417, 14766, 14783, 15043, 15301, 15326, 16223, 16234, 16316, 16362
K1801	Tib_5	Anduo, Naqu	M9a1a2a1a	73, 153, 263, 310, 489, 513, 523-524d, 750, 1041, 1438, 2706, 3106N, 3394, 4491, 4769, 6512, 7028, 7256, 8485, 8701, 8860, 9540, 10398, 10400, 10873, 11719, 12705, 14308, 14766, 14783, 15043, 15301, 15326, 16145, 16223, 16234, 16316
K1826	Tib_5	Anduo, Naqu	N11a1	73, 195, 240, 263, 310, 523-524d, 750, 813, 1438, 2706, 3106N, 4769, 6375, 6674, 7028, 7975, 8618, 8860, 10289, 10908, 11581A, 11719, 12634, 12705, 14311, 14431, 14488, 14502, 14766, 15326, 15514, 15900, 16145, 16183C, 16189, 16193+C, 16223, 16355, 16519
K1849	Tib_5	Anduo, Naqu	U2e1h	73, 217, 228, 263, 309+CC, 315+C, 340, 508, 750, 1438, 1811, 2706, 3106N, 3720, 4769, 5390, 5426, 6045, 6152, 7028, 8860, 10876, 11467, 11719, 12308, 12372, 13020, 13734, 14766, 15326, 15907, 16051, 16129C, 16183C, 16193+C, 16362, 16519
K38150	Tib_5	Anduo, Naqu	A15c2	73, 152, 153, 235, 310, 523-524d, 663, 750, 771, 1438, 1736, 2706, 3106N, 4248, 4769, 4824, 7028, 8459, 8794, 8860, 9052, 11719, 12705, 13111, 14067, 14766, 15262, 15326, 16223, 16290, 16319, 16362
K1803	Tib_5	Anduo, Naqu	D6a1	73, 263, 310, 489, 709, 750, 1438, 1719, 2706, 3106N, 3714, 4769, 4883, 4985, 5178A, 6701, 7028, 7424, 7879, 8701, 8860, 9540, 10398, 10400, 10873, 11719, 12654, 12705, 13194, 14766, 14783, 15043, 15218, 15301, 15326, 15388, 16223, 16274, 16362
K1840	Tib_5	Anduo, Naqu	A14a1	73, 151, 152, 200, 235, 263, 309+C, 315+C, 523-524d, 663, 735, 750, 1438, 1736, 2706, 3106N, 4248, 4769, 4824, 7028, 8334, 8794, 8860, 8862, 11719, 12004, 12705, 12990, 14766, 14803, 14905, 15326, 16093, 16172, 16223, 16278, 16290, 16319, 16362
K1805	Tib_5	Anduo, Naqu	F1g	73, 249d, 263, 310, 523-524d, 750, 1438, 2389, 2706, 3106N, 3398, 3970, 4769, 6392, 6962, 7028, 8860, 9389T, 10310, 10609, 11719, 12406, 12882, 13759, 13928C, 14766, 15326, 16183C, 16189, 16304, 16519

K1866	Tib_5	Anduo, Naqu	F2g1a	73, 249d, 263, 309+C, 315+C, 747, 750, 1005, 1438, 1824, 2706, 3106N, 3970, 4769, 6392, 6683, 7028, 7649, 7828, 8860, 10310, 10535, 10586, 11719, 12338, 12354, 13566, 13708, 13928C, 14766, 15326, 16185, 16266G, 16291, 16304, 16390, 16519
K1819	Tib_5	Anduo, Naqu	M13a2	73, 152, 263, 309+C, 315+C, 489, 513, 750, 1438, 2706, 2786, 3106N, 3644, 4769, 5773, 6023, 6253, 6620, 7028, 8405, 8563, 8701, 8860, 9540, 10373, 10398, 10400, 10411, 10790, 10873, 10920, 11204, 11719, 12705, 13395, 14766, 14769, 14783, 15043, 15236, 15301, 15326, 15924, 15944d, 16145, 16168, 16188, 16223, 16311, 16519
K1810	Tib_5	Anduo, Naqu	M62a1a1	73, 143, 146, 150, 263, 489, 750, 1438, 2706, 2735, 3106N, 3511, 4561, 4763, 4769, 7028, 7664, 8149, 8701, 8860, 9540, 9935, 10398, 10400, 10873, 11719, 12223, 12245, 12705, 13708, 14766, 14783, 15043, 15301, 15326, 15510, 15520, 15629, 15721, 16147, 16188+CC, 16223, 16295, 16452, 16487, 16519
K1856	Tib_5	Anduo, Naqu	M8a	73, 263, 309+C, 315+C, 489, 750, 1438, 2706, 3106N, 4314d, 4715, 4769, 6081T, 6179, 7028, 7196A, , 8453, 8584, 8684, 8701, 8860, 9540, 10398, 10400, 10873, 11719, 12705, 13647, 14470, 14766, 14783, 15043, 15047, 15301, 15326, 15487T, 15697, 16223, 16298, 16319
K1847	Tib_5	Anduo, Naqu	M8a	73, 263, 309+C, 315+C, 489, 750, 1438, 2706, 3106N, 4314d, 4715, 4769, 6081T, 6179, 7028, 7196A, , 8453, 8584, 8684, 8701, 8860, 9540, 10398, 10400, 10873, 11719, 12705, 13647, 14470, 14766, 14783, 15043, 15047, 15301, 15326, 15487T, 15697, 16223, 16298, 16319
K1851	Tib_5	Anduo, Naqu	M9a1a1c1b1a8	73, 198, 263, 309+CC, 315+C, 489, 711, 750, 1041, 1438, 2706, 3106N, 3338, 3394, 3438, 3992, 4491, 4769, 5894C, 7028, 7142, 7697, 8701, 8860, 9098, 9242, 9540, 9632, 9921, 10398, 10400, 10873, 11719, 12112, 12705, 14308, 14417, 14766, 14783, 15043, 15301, 15326, 16223, 16234, 16316, 16362
K1832	Tib_5	Anduo, Naqu	M9a1a1c1b1a	73, 263, 310, 489, 711, 750, 1041, 1438, 2706, 3106N, 3394, 4491, 4769, 5899+XC, 7028, 7142, 7697, 8701, 8860, 9242, 9540, 10398, 10400, 10873, 11719, 12705, 14308, 14417, 14766, 14783, 15043, 15301, 15326, 16223, 16234, 16316, 16362
K1835	Tib_5	Anduo, Naqu	M9a1a1c1b1a	73, 146TC, 152TC, 263, 309+C, 315+C, 489, 711, 750, 1041, 1438, 2706, 3106N, 3394, 4491, 4769, 5899+XC, 6719, 7028, 7142, 7697, 8701, 8860, 9242, 9540, 10398, 10400, 10873, 11719, 12705, 14308, 14417, 14766, 14783, 15043, 15301, 15326, 16223, 16234, 16316, 16362
K1831	Tib_5	Anduo, Naqu	M9a1b1e	73, 150, 152, 153, 263, 310, 489, 709, 750, 1041, 1438, 2706, 3106N, 3394, 4491, 4682, 4769, 7028, 8701, 8860, 9540, 10398, 10400, 10873, 11719, 12362, 12705, 14308, 14766, 14783, 15043, 15301, 15326, 15671, 16093, 16158, 16223, 16234, 16362, 16519
K2006	Tib_5	Anduo, Naqu	U4a1	73, 143, 152, 195, 263, 309+C, 315+C, 499, 750, 1438, 1811, 2706, 3106N, 4646, 4769, 5999, 6047, 7028, 7382, 8533GA, 8818, 8860, 9518, 11332, 11467, 11719, 12308, 12372, 12937, 14620, 14766, 15326, 15346, 15693, 16134, 16274, 16356, 16519
K1900	Tib_6	Baqing, Naqu	A	73, 152, 235, 263, 309+CC, 315+C, 523-524d, 663, 750, 1438, 1736, 2706, 3106N, 3828, 4248, 4760, 4769, 4824, 5704, 7028, 7337, 7765, 8794, 8860, 10724, 11719, 12705, 14766, 15142, 15326, 16223, 16290, 16319, 16362
K1877	Tib_6	Baqing, Naqu	G2b1b1	73, 263, 309+C, 315+C, 489, 709, 750, 1438, 2706, 3106N, 3975, 4769, 4833, 4853, 5108, 5601, 7028, 8701, 8860, 8877, 9540, 10398, 10400, 10873, 11719, 12375, 12705, 12795, 12892, 13563, 14563G, 14569, 14766, 14783, 15043, 15301, 15326, 16223, 16269, 16324, 16362
K1583	Tib_6	Baqing, Naqu	D4b2b8	73, 152, 263, 310, 390, 489, 523-524d, 750, 1382C, 1438, 2706, 3010, 3106N, 3398, 4769, 4883, 5178A, 7028, 8020, 8414, 8701, 8860, 8964, 9296, 9540, 9824A, 10398, 10400, 10873, 11719, 12705, 13500, 14668, 14764, 14766, 14783, 15043, 15301, 15326, 15328, 15613, 16223, 16326, 16362, 16519
K1881	Tib_6	Baqing, Naqu	M9a1a1c1b1a	73, 263, 309+C, 315+C, 489, 711, 750, 1041, 1438, 2706, 3106N, 3338, 3394, 4491, 4769, 5899+XC, 7028, 7142, 7697, 8701, 8860, 9242, 9540, 10398, 10400, 10873, 11719, 12705, 14308, 14417, 14766, 14783, 15043, 15301, 15326, 16223, 16234, 16316, 16362
K1871	Tib_6	Baqing, Naqu	D4	73, 263, 309+C, 315+C, 489, 524+AC, 750, 1438, 2706, 3010, 3106N, 4136, 4769, 4883, 4928, 5178A, 6194, 7028, 8414, 8701, 8860, 9540, 9856, 10084, 10398, 10400, 10825, 10873, 11719, 12705, 14569, 14668, 14766, 14783, 14996, 15043, 15301, 15326, 15364, 16093, 16192, 16223, 16316, 16362
K1910	Tib_6	Baqing, Naqu	A21c	73, 151, 152, 235, 263, 310, 663, 750, 1438, 1736, 3106N, 4248, 4769, 4824, 7028, 8506, 8794, 8860, 11719, 12705, 13145, 14364, 14766, 14962, 15326, 16223, 16290, 16311, 16319, 16362
K1909	Tib_6	Baqing, Naqu	M13a1b	73, 152, 263, 310, 489, 750, 1438, 2706, 3106N, 3644, 4616, 4769, 5021, 5773, 6023, 6253, 6620, 7028, 8701, 8860, 9540, 10398, 10400, 10411, 10790, 10873, 11719, 12011, 12705, 13135, 14766, 14783, 15043, 15301, 15326, 15924, 16145, 16148, 16188, 16189, 16223, 16381
K1889	Tib_6	Baqing, Naqu	M9a1a2a1a	73, 153, 263, 310, 489, 513, 523-524d, 750, 1041, 1438, 2706, 3106N, 3394, 4491, 4769, 6512, 7028, 7256, 8701, 8860, 9540, 10398, 10400, 10873, 11719, 12705, 13203, 14308, 14766, 14783, 15043, 15301, 15326, 16145, 16223, 16234, 16316, 16344
K1884	Tib_6	Baqing, Naqu	M13a2a1a	73, 152, 263, 309+C, 315+C, 489, 513, 750, 1438, 2706, 2786, 3106N, 3644, 4769, 5435, 5773, 6023, 6253, 6620, 7028, 8405, 8701, 8860, 9540, 10373, 10398, 10400, 10411, 10790, 10873, 11719, 12705, 13395, 14766, 14783, 15043, 15301, 15326, 15924, 15944d, 16145, 16168, 16223, 16257, 16311, 16519
K1887	Tib_6	Baqing, Naqu	A21b1	73, 152, 235, 263, 309+C, 315+C, 663, 750, 1438, 1736, 2706, 3106N, 4248, 4769, 4824, 6221, 7028, 8794, 8860, 9860, 11719, 12705, 13934, 14364, 14766, 15326, 16051, 16092, 16223, 16290, 16319, 16362

K1875	Tib_6	Baqing, Naqu	A21b	73, 152, 235, 263, 309+C, 315+C, 663, 750, 1438, 1736, 2706, 3106N, 4248, 4769, 4824, 6260, 7028, 8794, 8860, 11719, 12705, 13934, 14364, 14766, 15326, 16223, 16290, 16319, 16362
K1872	Tib_6	Baqing, Naqu	M9a1a1c1b1a7	73, 93, 228, 263, 309+C, 315+C, 489, 711, 750, 1041, 1438, 2706, 3106N, 3204, 3394, 4491, 4769, 5899+XC, 6680, 7028, 7142, 7697, 8701, 8860, 9242, 9540, 9698Y, 10398, 10400, 10873, 11719, 11887, 12705, 14308, 14417, 14766, 14783, 15043, 15301, 15326, 16223, 16234, 16316, 16362
K1911	Tib_6	Baqing, Naqu	D4j1a1d1	73, 263, 310, 489, 750, 1438, 2706, 3010, 3106N, 4769, 4883, 5178A, 5262, 7028, 7581, 7783, 8414, 8701, 8860, 9540, 10398, 10400, 10810, 10873, 11696, 11719, 12130, 12358, 12705, 14587, 14668, 14766, 14783, 15043, 15295, 15301, 15326, 16086, 16223, 16274, 16362, 16519
K1893	Tib_6	Baqing, Naqu	D5a2a1	73, 150, 199, 263, 309+C, 315+C, 489, 523-524d, 573+XC, 750, 752, 1107, 2706, 3106N, 3172+A, 4769, 4883, 5178A, 5301, 7028, 8701, 8860, 9180, 9540, 10397, 10398, 10400, 10873, 11719, 11944, 12026, 12705, 13278, 13590, 14180, 14766, 14783, 15043, 15301, 15326, 16129, 16164, 16182C, 16183C, 16189, 16223, 16266, 16295, 16362
K1906	Tib_6	Baqing, Naqu	M62b1b1	73, 150, 203, 204, 263, 309+CC, 315+C, 489, 524+ACAC, 750, 1438, 2706, 2735, 3030, 3106N, 3511, 4561, 4769, 7028, 7664, 7807, 8149, 8701, 8860, 9540, 10398, 10400, 10873, 11719, 12705, 13708, 14766, 14783, 15043, 15301, 15326, 15510, 15520, 15629, 15721, 16093, 16223, 16260, 16295, 16362, 16519
K1879	Tib_6	Baqing, Naqu	U2e1h	73, 217, 228, 263, 309+CC, 315+C, 340, 508, 750, 1438, 1811, 2706, 3106N, 3720, 4769, 5390, 5426, 6045, 6152, 7028, 8860, 10876, 11467, 11719, 12308, 12372, 13020, 13734, 14766, 15326, 15907, 16051, 16129C, 16183C, 16193+C, 16270, 16362, 16519
K1895	Tib_6	Baqing, Naqu	B4a4	73, 152, 193, 263, 309+CC, 315+C, 523-524d, 709, 750, 1438, 2706, 3106N, 4769, 5095, 5465, 7028, 8281-8289d, 8860, 9123, 11719, 14751, 14766, 15271, 15326, 15758, 16092, 16182C, 16183C, 16189, 16217, 16261, 16299, 16519
K1564	Tib_6	Baqing, Naqu	C4a1a1a1	73, 249d, 263, 310, 489, 523-524d, 750, 1438, 1715, 2232+AA, 2706, 3106N, 3552A, 3576, 4715, 4769, 4884, 4958, 6026, 7028, 7196A, 7999, 8508, 8584, 8701, 8860, 9540, 9545, 10398, 10400, 10873, 11719, 11914, 11969, 12672, 12705, 13263, 14318, 14766, 14783, 15043, 15204, 15301, 15326, 15487T, 15968, 16093, 16129, 16223, 16224, 16298, 16327, 16519
K1758	Tib_6	Baqing, Naqu	D4i	64, 65A, 73, 195, 237, 263, 310, 489, 501, 750, 1187, 1438, 2706, 3010, 3106N, 3644, 4769, 4883, 5178A, 7028, 8414, 8701, 8860, 9540, 10398, 10400, 10873, 11719, 12705, 14180, 14668, 14766, 14783, 15043, 15301, 15326, 15884C, 16093, 16223, 16294, 16362
K1323	Tib_6	Baqing, Naqu	D4j1a1d	73, 195, 263, 310, 489, 750, 1438, 2706, 3010, 3106N, 4769, 4883, 5178A, 5262, 7028, 7581, 7783, 8414, 8701, 8860, 9540, 10398, 10400, 10873, 11696, 11719, 12130, 12358, 12705, 14668, 14766, 14783, 15043, 15295, 15301, 15326, 16086, 16223, 16274, 16293, 16362, 16519
K1870	Tib_6	Baqing, Naqu	D4j1a1e	73, 263, 310, 489, 750, 1438, 2706, 3010, 3106N, 4769, 4883, 5178A, 5262, 7028, 7581, 7783, 8414, 8701, 8860, 9540, 10398, 10400, 10873, 11696, 11719, 12130, 12358, 12705, 14668, 14766, 14783, 15043, 15295, 15301, 15326, 16086, 16223, 16362, 16399, 16519
K1904	Tib_6	Baqing, Naqu	F2a1	73, 249d, 263, 309+C, 315+C, 408A, 750, 1005, 1438, 1824, 2706, 3106N, 3970, 4769, 6392, 7028, 7828, 8281-8289d, 8860, 10310, 10535, 10586, 10810, 11719, 12338, 13708, 13928C, 14100, 14560, 14766, 15326, 16203, 16304, 16519
K1874	Tib_6	Baqing, Naqu	G1a2	73, 263, 309+C, 315+C, 489, 709, 750, 1438, 2706, 3106N, 4769, 4833, 5108, 7028, 7867, 8200, 8701, 8860, 9540, 10398, 10400, 10873, 11719, 12040, 12705, 14569, 14766, 14783, 14950, 15043, 15301, 15323, 15326, 15497, 16184, 16223, 16290, 16362, 16519
K1890	Tib_6	Baqing, Naqu	G2a1h1a	73, 146, 263, 283, 309+CC, 315+C, 489, 709, 750, 1438, 2706, 3106N, 4158, 4769, 4833, 5108, 5601, 7028, 7600, 8701, 8860, 9377, 9540, 9575, 10398, 10400, 10873, 11084, 11719, 12705, 13194, 13563, 14200, 14569, 14766, 14783, 15043, 15301, 15326, 16227, 16278, 16362
K1905	Tib_6	Baqing, Naqu	G2b1b1	73, 263, 309+CC, 315+C, 489, 709, 750, 1438, 2706, 3106N, 4769, 4833, 4853, 5108, 5601, 7028, 8701, 8860, 8877, 8896, 9540, 9585, 10398, 10400, 10873, 11719, 12375, 12705, 13563, 14569, 14766, 14783, 15043, 15301, 15326, 16223, 16269, 16362
K1914	Tib_6	Baqing, Naqu	G3b1	73, 195, 198, 263, 309+C, 315+C, 489, 709, 750, 1438, 2706, 3106N, 4769, 4833, 5108, 7028, 8701, 8860, 9540, 9599, 10398, 10400, 10873, 11719, 12705, 13477, 14569, 14605, 14766, 14783, 15043, 15301, 15326, 15927, 16093, 16104, 16223, 16274, 16362
K1883	Tib_6	Baqing, Naqu	M9a1a1c1b1a	73, 263, 309+C, 315+C, 489, 711, 750, 1041, 1438, 2706, 3106N, 3394, 4491, 4769, 5899+XC, 7028, 7142, 7697, 8701, 8860, 9242, 9540, 10398, 10400, 10873, 11719, 12705, 14308, 14417, 14766, 14783, 15043, 15301, 15326, 16223, 16234, 16316, 16362
K1896	Tib_6	Baqing, Naqu	M9a1a1c1b1a2	73, 146, 263, 297, 309+C, 315+C, 489, 711, 750, 1041, 1438, 2706, 3106N, 3394, 3438, 4491, 4769, 5899+XC, 6446, 7028, 7142, 7697, 8521, 8701, 8860, 9242, 9540, 10398, 10400, 10873, 11437, 11719, 12705, 14308, 14417, 14766, 14783, 15043, 15301, 15326, 16223, 16234, 16316, 16362
K1868	Tib_6	Baqing, Naqu	M9a1b1c	73, 150, 152, 153, 263, 309+C, 315+C, 489, 750, 1041, 1438, 2706, 3106N, 3394, 4491, 4769, 7028, 8701, 8860, 9214, 9540, 10398, 10400, 10454, 10873, 11719, 12362, 12705, 14308, 14766, 14783, 15043, 15301, 15326, 15671, 16158, 16223, 16234, 16362, 16519

K1897	Tib_6	Baqing, Naqu	X2p	73, 195, 263, 309+C, 315+C, 750, 1438, 1719, 2625, 2706, 3106N, 4769, 6221, 6371, 7028, 7109, 8227, 8860, 10143, 11719, 12705, 13966, 14470, 14766, 15326, 16223, 16249, 16278, 16362, 16519
K1902	Tib_6	Baqing, Naqu	A15a1	73, 152, 199, 204, 207, 235, 309+C, 315+C, 523-524d, 663, 750, 1438, 1736, 2706, 3106N, 4248, 4769, 4824, 6239, 7028, 8459, 8794, 8860, 11084, 11719, 12705, 14067, 14766, 15326, 16223, 16274, 16290, 16319, 16362
K1508	Tib_6	Baqing, Naqu	B4d4	73, 263, 309+CC, 315+C, 750, 827, 1438, 2706, 2755, 3106N, 4769, 5321, 5372, 6324C, 7028, 8281-8289d, 8860, 11084, 11719, 11914, 13942, 14766, 15326, 15448, 15535, 15930, 16183C, 16189, 16193+CC, 16217, 16234, 16519
K1892	Tib_6	Baqing, Naqu	D4j1a1e	73, 263, 310, 489, 750, 1438, 2706, 3010, 3106N, 4769, 4883, 5178A, 5262, 7028, 7581, 7783, 8414, 8701, 8860, 9540, 10398, 10400, 10873, 11696, 11719, 12130, 12358, 12705, 14668, 14766, 14783, 15043, 15295, 15301, 15326, 16086, 16223, 16362, 16399, 16519
K1869	Tib_6	Baqing, Naqu	D4j1a1c	73, 195, 263, 309+C, 315+C, 489, 709, 750, 1438, 2706, 3010, 3106N, 3397, 4769, 4883, 5178A, 5262, 6704, 7028, 7581, 7783, 8414, 8701, 8860, 9540, 10398, 10400, 10873, 11696, 11719, 12130, 12358, 12705, 14668, 14766, 14783, 15043, 15295, 15301, 15326, 16086, 16092, 16223, 16362
K1594	Tib_6	Baqing, Naqu	G3a1a2	16T, 73, 143, 150, 263, 309+C, 315+C, 489, 709, 750, 1438, 2706, 3106N, 4769, 4833, 5108, 6962, 7028, 8701, 8860, 8861, 9540, 10398, 10400, 10873, 11719, 11914, 12705, 14569, 14766, 14783, 15043, 15301, 15326, 15465, 15746, 16148, 16153, 16215, 16223, 16274
K1526	Tib_6	Baqing, Naqu	Z3a	73, 152, 249d, 263, 309+C, 315+C, 489, 750, 1438, 2706, 3106N, 4715, 4769, 5399, 6752, 7028, 7196A, 8020, 8584, 8701, 8860, 9090, 9540, 9713, 10208, 10398, 10400, 10873, 11719, 12705, 13620, 13942, 14766, 14783, 15043, 15301, 15326, 15487T, 15784, 15928, 16185, 16223, 16260, 16298
K1885	Tib_6	Baqing, Naqu	F1c1a1a	73, 152, 249d, 263, 309+C, 315+C, 523-524d, 573+XC, 709, 750, 1438, 2706, 3106N, 3970, 4769, 6392, 6599, 6962, 7028, 7684, 8860, 9053, 9647, 10310, 10454, 10609, 11719, 12406, 12882, 13759, 13928C, 14766, 15326, 16111, 16129, 16266, 16304, 16519
K1473	Tib_6	Baqing, Naqu	A14b1	73, 152, 200, 235, 263, 315+C, 523-524d, 663, 735, 750, 1438, 1598, 1736, 2706, 3106N, 4248, 4769, 4824, 7028, 7705, 7805, 8794, 8860, 11719, 12705, 14766, 15326, 16223, 16290, 16319, 16362
K1901	Tib_6	Baqing, Naqu	M9a1a2a1b1	73, 153, 263, 309+C, 315+C, 489, 513, 750, 1041, 1438, 2706, 3106N, 3394, 4491, 4769, 7028, 7256, 8701, 8860, 9540, 10398, 10400, 10873, 11719, 12705, 14142A, 14308, 14766, 14783, 15043, 15301, 15326, 16145, 16223, 16234, 16293, 16316
K1982	Tib_6	Baqing, Naqu	A11a1a	73, 152, 235, 263, 310, 523-524d, 663, 750, 1005, 1438, 1736, 2706, 3106N, 4248, 4769, 4812, 4824, 5899+XC, 6755, 7028, 8794, 8843, 8860, 9650, 11719, 12705, 12915A, 13135, 14110, 14766, 15326, 16093, 16223, 16290, 16293C, 16319
K1674	Tib_6	Baqing, Naqu	D4b2b8	73, 194, 263, 309+C, 315+C, 390, 456, 489, 523-524d, 750, 1382C, 1438, 2706, 3010, 3106N, 3398, 4769, 4883, 5178A, 7028, 7474, 8020, 8414, 8701, 8860, 8964, 9296, 9540, 9824A, 10398, 10400, 10873, 11719, 12705, 14668, 14766, 14783, 15043, 15301, 15326, 15613, 16223, 16362, 16519
K1425	Tib_6	Baqing, Naqu	D4j1a1	73, 263, 309+C, 315+C, 489, 523-524d, 750, 1438, 2706, 3010, 3106N, 4769, 4883, 5054, 5178A, 5262, 7028, 7581, 7783, 8414, 8701, 8860, 9540, 10191G, 10398, 10400, 10873, 10915, 11696, 11719, 12130, 12358, 12705, 14569, 14668, 14766, 14783, 15043, 15295, 15301, 15326, 16086, 16150, 16223, 16362, 16519
K1903	Tib_6	Baqing, Naqu	F1b1	73, 249d, 263, 310, 481, 523-524d, 750, 1438, 2706, 3106N, 3970, 4732, 4769, 5147, 6392, 6962, 7028, 8860, 10310, 10609, 10976, 11719, 12406, 12633, 12882, 13928C, 14476, 14766, 15326, 15725, 16183C, 16189, 16232A, 16249, 16304, 16311, 16519
K1888	Tib_6	Baqing, Naqu	F2b1	73, 146, 249d, 263, 310, 523-524d, 750, 1005, 1438, 1824, , 2706, 3106N, 3970, 4769, 4811, 5150, 6392, 7028, 7828, 8860, 10310, 10535, 10586, 11719, 12338, 13708, 13928C, 14766, 15326, 16092A, 16093, 16183C, 16189, 16193+C, 16291, 16304, 16311
K1913	Tib_6	Baqing, Naqu	M9a1a1c1b1a3	73, 263, 309+CC, 315+C, 489, 711, 750, 1041, 1438, 2706, 3106N, 3394, 4491, 4769, 5899+XC, 7028, 7142, 7697, 8701, 8860, 9242, 9540, 10398, 10400, 10873, 11719, 12705, 13419, 14308, 14417, 14766, 14783, 15043, 15301, 15326, 16223, 16234, 16316, 16362
K1916	Tib_6	Baqing, Naqu	A14a1	73, 151, 152, 200, 235, 263, 310, 523-524d, 663, 735, 750, 1438, 1736, 2706, 3106N, 4248, 4769, 4824, 7028, 8334, 8794, 8860, 8862, 11719, 12004, 12705, 12990, 14766, 14803, 15326, 16093, 16172, 16223, 16290, 16319, 16362
K1894	Tib_6	Baqing, Naqu	M13a2a1e	73, 152, 263, 310, 489, 513, 750, , 1438, , 2706, 2786, 3106N, 3644, 4769, 5773, 6023, 6253, 6620, 7028, 8405, 8701, 8860, 9540, 10373, 10398, 10400, 10411, 10790, 10873, 11719, 12135C\A, M, 12705, 13395, 13981, 14766, 14783, 15043, 15301, 15326, 15924, 15944d, 16145, 16168, 16188, 16223, 16257, 16311, 16519
K1899	Tib_6	Baqing, Naqu	Z3a	73, 152, 249d, 263, 309+C, 315+C, 489, 750, 1438, 2706, 3106N, 4715, 4769, 5399, 6752, 7028, 7196A, 8584, 8701, 8860, 9090, 9540, 9626, 9713, 10208, 10398, 10400, 10873, 11719, 12705, 13620, 13942, 14766, 14783, 15043, 15301, 15326, 15487T, 15784, 15928, 16185, 16223, 16260, 16298
K1680	Tib_6	Baqing, Naqu	C4a1a1a3	73, 249d, 263, 310, 489, 523-524d, 750, 1438, 1715, 2232+AA, 2706, 3106N, 3552A, 3576, 4715, 4769, 4884, 4958, 6026, 7028, 7196A, 7999, 8508, 8584, 8701, 8860, 9540, 9545, 10398, 10400, 10873, 11719, 11914, 11969, 12672, 12705, 13263, 14318, 14766, 14783, 15043, 15204, 15301, 15326, 15487T, 15617, 15968, 16129, 16223, 16298, 16327, 16519

K1509	Tib_6	Baqing, Naqu	R31?	73, 146, 249d, 263, 310, 523-524d, 750, 1438, 1531, 2706, 3106N, 3963, 4769, 7028, 7295, 7981, 8860, 9380, 11719, 13557, 14553, 14766, 15326, 15458, 16172, 16265T, 16304, 16362, 16519
K1969	Tib_7	Jiali, Naqu	F2g1a	73, 249d, 263, 310, 747, 750, 1005, 1438, 1824, 2706, 3106N, 3834, 3970, 4769, 6267, 6392, 6485, 6683, 7028, 7754, 7828, 8860, 10310, 10535, 10586, 11719, 12338, 13708, 13928C, 14766, 15326, 16185, 16266A, 16291, 16304, 16519
K1475	Tib_7	Jiali, Naqu	D4j3b1	73, 94, 263, 309+C, 315+C, 338, 489, 750, 1438, 2706, 3010, 3106N, 4769, 4883, 5178A, 7028, 8414, 8701, 8860, 9540, 10398, 10400, 10873, 11696, 11719, 12705, 14668, 14766, 14783, 15043, 15301, 15326, 16184, 16223, 16311, 16362
K38192	Tib_7	Jiali, Naqu	M9a1a1c1b1a	73, 263, 309+CC, 315+C, 489, 524+AC, 711, 750, 1041, 1438, 2706, 3106N, 3394, 3736, 4491, 4769, 5899+XC, 7028, 7142, 7697, 8701, 8860, 9242, 9540, 10398, 10400, 10873, 11719, 12705, 14308, 14417, 14766, 14783, 15043, 15301, 15326, 16223, 16234, 16316, 16362
K1298	Tib_7	Jiali, Naqu	Z3a	73, 249d, 263, 279, 310, 489, 750, 1438, 1700, 2706, 3106N, 4491, 4715, 4769, 5399, 6752, 7028, 7196A, 8584, 8701, 8860, 9090, 9128, 9540, 9713, 10208, 10398, 10400, 10873, 11719, 12705, 12810, 13620, 13942, 14766, 14783, 15043, 15301, 15326, 15487T, 15784, 15928, 16111A, 16185, 16215, 16223, 16260, 16298, 16355
K1300	Tib_7	Jiali, Naqu	A14b1	73, 152, 200, 235, 263, 310, 523-524d, 663, 735, 750, 1438, 1598, 1736, 2706, 3106N, 4248, 4769, 4824, 7028, 7705, 7805, 8794, 8860, 11719, 12705, 14766, 15326, 16223, 16290, 16319, 16362
K1297	Tib_7	Jiali, Naqu	A15a1	73, 152, 199, 204, 207, 235, 309+CC, 315+C, 523-524d, 663, 750, 1438, 1736, 2706, 3106N, 4248, 4769, 4824, 6239, 7028, 8459, 8794, 8860, 11084, 11719, 12705, 14067, 14766, 15326, 16223, 16274, 16290, 16319, 16362, 16519
K1253	Tib_7	Jiali, Naqu	A15a1	73, 152, 199, 204, 207, 235, 309+CC, 315+C, 523-524d, 663, 750, 1438, 1736, 2706, 3106N, 4248, 4769, 4824, 6239, 7028, 8459, 8794, 8860, 11084, 11719, 12705, 14067, 14766, 15326, 16223, 16274, 16290, 16319, 16362, 16519
K1270	Tib_7	Jiali, Naqu	D4b2b8	73, 263, 310, 390, 489, 523-524d, 750, 1382C, 1438, 2706, 3010, 3106N, 3398, 4769, 4883, 5178A, 7028, 8020, 8414, 8701, 8860, 8964, 9296, 9540, 9824A, 10398, 10400, 10873, 11719, 12705, 12940, 13488, 13500, 14668, 14766, 14783, 15043, 15301, 15326, 15613, 16223, 16362, 16519
K1279	Tib_7	Jiali, Naqu	D4j3b	73, 263, 309+C, 315+C, 338, 489, 750, 1438, 2706, 3010, 3106N, 4769, 4883, 5178A, 7028, 8414, 8701, 8860, 9540, 10398, 10400, 10873, 11696, 11719, 12705, 13212, 14668, 14766, 14783, 15043, 15301, 15326, 16181, 16184, 16223, 16311, 16362
K1301	Tib_7	Jiali, Naqu	H7b2	263, 309+C, 315+C, 750, 1438, 3106N, 4769, 4793, 5348, 5460, 6266, 8860, 13323, 15326, 16519
K1258	Tib_7	Jiali, Naqu	M62b1b1	73, 150, 203, 204, 263, 309+C, 315+C, 489, 524+ACAC, 750, 1438, 2706, 2735, 3030, 3106N, 3511, 4561, 4769, 7028, 7664, 7807, 8149, 8701, 8860, 9540, 10398, 10400, 10873, 11719, 12705, 13708, 14766, 14783, 15043, 15301, 15326, 15510, 15520, 15629, 15721, 16093, 16223, 16260, 16295, 16362, 16519
K1294	Tib_7	Jiali, Naqu	M9a1b1d	73, 150, 152, 153, 263, 310, 489, 750, 1041, 1438, 2706, 3106N, 3394, 4491, 4769, 7028, 8701, 8860, 9010, 9540, 10398, 10400, 10873, 11719, 12362, 12705, 14308, 14766, 14783, 15043, 15119, 15301, 15326, 15671, 16158, 16223, 16234, 16362, 16519
K1286	Tib_7	Jiali, Naqu	A15a1	73, 152, 199, 204, 207, 235, 309+CC, 315+C, 523-524d, 663, 750, 1438, 1736, 2706, 3106N, 4248, 4769, 4824, 6239, 7028, 8459, 8794, 8860, 11084, 11719, 12705, 14067, 14766, 15326, 16223, 16274, 16290, 16319, 16362, 16519
K1274	Tib_7	Jiali, Naqu	A17a2	73, 152, 235, 263, 523-524d, 538, 663, 723, 750, 1438, 1736, 2706, 3106N, 4113, 4248, 4769, 4824, 5514, 7028, 8794, 8860, 9126, 11719, 12705, 14766, 15217, 15326, 16037, 16086, 16129, 16223, 16278, 16290, 16319, 16362
K1285	Tib_7	Jiali, Naqu	D4j3b	73, 263, 309+CC, 315+C, 338, 489, 750, 1438, 2706, 3010, 3106N, 4769, 4883, 5178A, 7028, 8414, 8701, 8860, 9540, 10398, 10400, 10873, 11696, 11719, 12705, 14668, 14766, 14783, 15043, 15301, 15326, 16184, 16223, 16311, 16362
K1283	Tib_7	Jiali, Naqu	F1b1	73, 249d, 263, 309+C, 315+C, 523-524d, 750, 2706, 3106N, 3970, 4732, 4769, 5147, 6392, 6962, 7028, 8860, 10310, 10609, 10976, 11719, 12406, 12633, 12882, 13928C, 14476, 14766, 15326, 16183C, 16189, 16193+CC, 16232A, 16249, 16304, 16311, 16360, 16519
K1256	Tib_7	Jiali, Naqu	F1d1a2	73, 146, 249d, 263, 309+C, 315+C, 523-524d, 750, 1438, 1734, 2706, 3106N, 3970, 4769, 5341T, 5628, 6392, 6962, 7028, 7738, 8860, 10310, 10609, 11152, 11719, 12406, 12882, 13135, 13928C, 14766, 15326, 15402, 16129, 16145, 16183C, 16189, 16284, 16304, 16519
K1271	Tib_7	Jiali, Naqu	F2g1a	73, 249d, 263, 310, 747, 750, 1005, 1438, 1824, 2706, 3106N, 3834, 3970, 4769, 6267, 6392, 6683, 7028, 7754, 7828, 8860, 10310, 10535, 10586, 11719, 12338, 13708, 13928C, 14766, 15326, 16185, 16266A, 16291, 16304, 16519
K1269	Tib_7	Jiali, Naqu	G3a1a1a	16T, 73, 143, 150, 263, 309+C, 315+C, 489, 709, 750, 1438, 2706, 3106N, 4674, 4769, 4833, 5108, 7028, 8701, 8860, 8861, 9540, 10398, 10400, 10873, 11719, 11914, 12612, 12705, 14569, 14766, 14783, 15043, 15301, 15326, 15746, 16215, 16223, 16274

K1559	Tib_7	Jiali, Naqu	M13a2a1a	73, 152, 263, 309+C, 315+C, 489, 513, 750, 1438, 2706, 2786, 3106N, 3644, 4769, 5773, 6023, 6253, 6620, 7028, 8405, 8701, 8860, 9540, 10373, 10398, 10400, 10411, 10790, 10873, 11719, 12705, 13395, 14766, 14783, 15043, 15301, 15326, 15924, 15944d, 16145, 16168, 16172, 16223, 16257, 16311, 16519
K1536	Tib_7	Jiali, Naqu	M9a1a1c1b1a5	73, 159, 263, 310, 489, 711, 750, 1041, 1438, 2706, 3106N, 3394, 4491, 4769, 5899+XC, 7028, 7142, 7697, 8701, 8860, 9242, 9540, 10398, 10400, 10873, 11719, 12705, 14308, 14766, 14783, 15043, 15301, 15326, 15766, 16223, 16234, 16316, 16362
K1290	Tib_7	Jiali, Naqu	M9a1a1c1b1a	73, 263, 309+C, 315+C, 489, 711, 750, 1041, 1438, 2706, 3106N, 3394, 4491, 4769, 5899+XC, 7028, 7142, 7697, 8701, 8860, 9242, 9540, 9782, 10398, 10400, 10873, 11719, 12705, 14308, 14417, 14766, 14783, 15043, 15301, 15326, 16223, 16234, 16316, 16362
K1284	Tib_7	Jiali, Naqu	M9a1a1c1b1a13	73, 263, 309+C, 315+C, 489, 711, 750, 1041, 1438, 2706, 3106N, 3394, 3866, 4021, 4491, 4769, 5474, 5899+XC, 7028, 7142, 7697, 8701, 8860, 9242, 9540, 10398, 10400, 10873, 11719, 12705, 14308, 14417, 14766, 14783, 15043, 15301, 15326, 16094, 16194, 16223, 16234, 16316, 16362
K1296	Tib_7	Jiali, Naqu	A14a1	73, 151, 152, 200, 235, 263, 309+C, 315+C, 523-524d, 663, 735, 750, 1438, 1736, 2706, 3106N, 4248, 4769, 4824, 7028, 8334, 8794, 8860, 8862, 11719, 12004, 12705, 12990, 14766, 14803, 15326, 16093, 16172, 16223, 16278, 16290, 16319, 16362
K1278	Tib_7	Jiali, Naqu	M13a1b	73, 152, 263, 310, 489, 710, 750, 1438, 2706, 3106N, 3644, 4769, 5773, 6023, 6253, 6620, 7028, 8020, 8701, 8860, 9540, 10398, 10400, 10411, 10790, 10873, 11719, 11818, 12705, 13135, 13827, 14766, 14783, 15043, 15301, 15326, 15924, 16145, 16148, 16188, 16189, 16223, 16381
K1293	Tib_7	Jiali, Naqu	M13a2	73, 152, 263, 309+C, 315+C, 489, 513, 709, 750, 1438, 2706, 2786, 3106N, 3644, 4769, 5773, 6023, 6253, 6620, 7028, 8405, 8701, 8860, 8939, 9540, 10373, 10398, 10400, 10411, 10790, 10873, 11719, 12705, 13395, 14198, 14766, 14783, 15043, 15301, 15326, 15924, 15944d, 16145, 16168, 16188, 16223, 16311, 16519
K1275	Tib_7	Jiali, Naqu	M9a1a1c1b1a13	73, 263, 309+C, 315+C, 489, 711, 750, 1041, 1438, 2706, 3106N, 3394, 3866, 4021, 4491, 4769, 5474, 5899+XC, 7028, 7142, 7697, 8701, 8860, 9242, 9540, 10398, 10400, 10873, 11719, 12705, 14308, 14417, 14766, 14783, 15043, 15301, 15326, 16094, 16194, 16223, 16234, 16316, 16362
K1260	Tib_7	Jiali, Naqu	M9a1a1c1b1a	73, 263, 309+C, 315+C, 489, 711, 750, 1041, 1438, 2706, 3106N, 3394, 4491, 4769, 5899+XC, 7028, 7142, 7697, 8701, 8860, 9242, 9540, 10398, 10400, 10873, 11719, 12705, 14308, 14417, 14766, 14783, 15043, 15301, 15326, 16223, 16234, 16316, 16362
K1259	Tib_7	Jiali, Naqu	M9a1a1c1b1a	73, 263, 309+C, 315+C, 489, 573+XC, 711, 750, 1041, 1438, 2706, 3106N, 3394, 4491, 4769, 5899+XC, 7028, 7142, 7697, 8701, 8860, 9242, 9540, 9932, 10398, 10400, 10873, 11719, 11770, 12705, 14308, 14417, 14766, 14783, 15043, 15301, 15326, 15497, 16223, 16234, 16316, 16362
K1266	Tib_7	Jiali, Naqu	T1a1b1	73, 152, 195, 263, 309+C, 315+C, 709, 750, 1438, 1888, 2706, 3106N, 4216, 4769, 4917, 7028, 8697, 8860, 9548, 9899, 10143, 10463, 11251, 11719, 12028, 12633A, 13368, 14281, 14766, 14905, 15326, 15452A, 15607, 15928, 16126, 16129, 16163, 16186, 16189, 16234A, 16294, 16519
K1272	Tib_7	Jiali, Naqu	F1d1a2	73, 146, 249d, 263, 309+C, 315+C, 523-524d, 750, 1438, 1734, 2706, 3106N, 3970, 4769, 5628, 6392, 6962, 7028, 7738, 8860, 9053, 10310, 10609, 11719, 12406, 12882, 13135, 13928C, 14766, 15326, 15402, 16145, 16189, 16193+CC, 16284, 16304, 16519
K1254	Tib_7	Jiali, Naqu	G2b1b2a	73, 263, 309+C, 315+C, 489, 709, 750, 1438, 2706, 3106N, 3975, 4769, 4833, 4853, 5108, 5601, 7028, 8701, 8860, 8877, 9540, 10398, 10400, 10873, 11719, 12375, 12705, 12795, 13563, 14563G, 14569, 14766, 14783, 15043, 15301, 15326, 16223, 16324, 16362
K1276	Tib_7	Jiali, Naqu	A	73, 152, 235, 263, 309+C, 315+C, 523-524d, 576, 663, 750, 1438, 1736, 2706, 3106N, 3229+A, 4232, 4248, 4769, 4824, 7028, 8794, 8860, 11719, 12705, 14766, 15326, 16125, 16223, 16290, 16311, 16319, 16362
K1325	Tib_7	Jiali, Naqu	A15a	73, 152, 204, 207, 235, 309+CC, 315+C, 523-524d, 663, 750, 1438, 1736, 2706, 3106N, 4053, 4248, 4769, 4824, 7028, 8200, 8459, 8794, 8860, 11084, 11719, 12705, 14067, 14533A, 14569, 14766, 14831, 15326, 16223, 16274, 16290, 16319, 16362
K1273	Tib_7	Jiali, Naqu	A17a2	73, 152, 235, 263, 523-524d, 538, 663, 723, 750, 1438, 1736, 2706, 3106N, 4113, 4248, 4769, 4824, 5514, 7028, 8794, 8860, 9126, 11719, 12705, 14766, 15217, 15326, 16037, 16086, 16129, 16223, 16278, 16290, 16319, 16362
K1265	Tib_7	Jiali, Naqu	F1b1	73, 249d, 263, 309+C, 315+C, 523-524d, 750, 2706, 3106N, 3764CT, 3970, 4732, 4769, 5147, 6392, 6962, 7028, 8860, 10310, 10609, 10976, 11719, 12406, 12633, 12882, 13928C, 14476, 14766, 15326, 16183C, 16189, 16193+CC, 16232A, 16249, 16304, 16311, 16360, 16519
K1291	Tib_7	Jiali, Naqu	F1g	73, 249d, 263, 310, 523-524d, 750, 1438, 2389, 2706, 3106N, 3398, 3970, 4769, 6392, 6962, 7028, 8860, 10310, 10609, 11719, 12406, 12882, 13928C, 14766, 15326, 16129, 16182C, 16183C, 16189, 16304, 16519
K1252	Tib_7	Jiali, Naqu	G1	73, 263, 310, 489, 709, 750, 1438, 2706, 3106N, 4769, 4833, 5108, 7028, 7867, 8200, 8701, 8860, 9540, 10398, 10400, 10873, 11305, 11719, 12705, 14569, 14766, 14783, 15043, 15301, 15323, 15326, 15497, 16223, 16311, 16362, 16519
K1299	Tib_7	Jiali, Naqu	G1	73, 263, 309+CC, 315+C, 489, 709, 750, 1438, 2706, 3106N, 4769, 4833, 5108, 7028, 7867, 8200, 8701, 8860, 9540, 10398, 10400, 10873, 11719, 12705, 14569, 14766, 14783, 15043, 15301, 15323, 15326, 15497, 16223, 16362, 16519

K1250	Tib_7	Jiali, Naqu	K1a4	73, 263, 310, 497, 573+XC, 750, 1189, 1438, 1811, 2706, 3106N, 3480, 4769, 7028, 8588, 8860, 9055, 9575, 9667, 9698, 10398, 10550, 10560, 11299, 11467, 11485, 11719, 12308, 12372, 14167, 14766, 14798, 15326, 15904, 16093, 16145, 16224, 16311, 16519
K1277	Tib_7	Jiali, Naqu	M13a1b	73, 152, 263, 310, 489, 710, 750, 1438, 2706, 3106N, 3644, 4769, 5773, 6023, 6253, 6620, 7028, 8020, 8701, 8860, 9540, 10398, 10400, 10411, 10790, 10873, 11719, 11818, 12705, 13135, 13827, 14766, 14783, 15043, 15301, 15326, 15924, 16145, 16148, 16188, 16189, 16223, 16381
K1268	Tib_7	Jiali, Naqu	M7b1a1a3	73, 150, 199, 204, 207, 263, 310, 489, 750, 1438, 2706, 3106N, 4048, 4071, 4164, 4679, 4769, 5351, 5460, 6455, 6680, 7028, 7684, 7853, 8701, 8860, 9540, 9824, 10398, 10400, 10873, 11719, 12405, 12705, 12811, 14766, 14783, 14978, 15043, 15301, 15326, 16129, 16223, 16248, 16297
K1267	Tib_7	Jiali, Naqu	M9a1a1c1b1a	73, 263, 309+C, 315+C, 489, 711, 750, 1041, 1438, 2706, 3106N, 3394, 4491, 4769, 5899+XC, 7028, 7142, 7697, 8701, 8860, 9242, 9540, 10398, 10400, 10873, 11719, 12705, 14308, 14417, 14766, 14783, 15043, 15301, 15326, 16223, 16234, 16316, 16362
K1289	Tib_7	Jiali, Naqu	M9a1b1d	73, 150, 152, 153, 263, 310, 489, 750, 1041, 1438, 2706, 3106N, 3394, 4491, 4769, 7028, 8701, 8860, 9010, 9540, 10398, 10400, 10873, 11719, 12362, 12705, 14308, 14766, 14783, 15043, 15119, 15301, 15326, 15671, 16158, 16223, 16234, 16362, 16519
K2278	Tib_8	Kangma, Shigatse	F1g2	73, 195, 249d, 263, 309+CC, 315+C, 523-524d, 750, 1438, 2389, 2706, 3106N, 3398, 3970, 4769, 6392, 6962, 7013, 7028, 8860, 10310, 10609, 11719, 12406, 12882, 13928C, 14766, 15326, 16182C, 16183C, 16189, 16304, 16519
K2277	Tib_8	Kangma, Shigatse	A11a1a	73, 152, 235, 263, 309+CC, 315+C, 523-524d, 663, 750, 1005, 1438, 1736, 2706, 3106N, 4248, 4769, 4824, 5432, 5899+XC, 6392, 6755, 7028, 8794, 8843, 8860, 9650, 11719, 12705, 14766, 15326, 16093, 16223, 16290, 16293C, 16319
K2240	Tib_8	Kangma, Shigatse	C4a2b	73, 249d, 263, 310, 489, 513, 750, 1438, 2232+A, 2706, 2887, 3106N, 3552A, 4696, 4715, 4769, 6026, 7028, 7196A, 7286, 8584, 8701, 8860, 9540, 9545, 10398, 10400, 10873, 11719, 11914, 11969, 12624, 12672, 12705, 13263, 14318, 14766, 14783, 15043, 15204, 15301, 15326, 15487T, 16223, 16298, 16327, 16357, 16519
K2191	Tib_8	Kangma, Shigatse	M62b2a	73, 150, 187T, 204, 263, 309+C, 315+C, 489, 750, 1438, 2706, 2735, 3106N, 3511, 3693, 4561, 4769, 6305, 7028, 7364, 7664, 8149, 8701, 8860, 9540, 10398, 10400, 10873, 11719, 12705, 13708, 14766, 14783, 15043, 15301, 15326, 15510, 15520, 15629, 15721, 16192, 16223, 16260, 16278, 16311, 16519
K2190	Tib_8	Kangma, Shigatse	A11a1a	73, 152, 235, 263, 309+CC, 315+C, 523-524d, 663, 750, 1005, 1438, 1736, 2706, 3106N, 4248, 4769, 4824, 5899+XC, 6755, 7028, 8794, 8843, 8860, 9650, 11719, 12705, 14766, 15326, 16093, 16223, 16290, 16293C, 16319, 16362, 16497
K2166	Tib_8	Kangma, Shigatse	M9a1a1c1b1a	73, 195, 263, 310, 318, 489, 711, 750, 1041, 1438, 2706, 3106N, 3394, 4491, 4769, 5899+XC, 7028, 7142, 7697, 8701, 8860, 9242, 9540, 10398, 10400, 10873, 11719, 12705, 14308, 14417, 14766, 14783, 15043, 15301, 15326, 16129, 16223, 16234, 16316, 16362, 16390
K2478	Tib_8	Kangma, Shigatse	A11a1a	73, 151, 152, 235, 263, 309+C, 315+C, 523-524d, 663, 750, 1005, 1438, 1736, 2706, 3106N, 4248, 4769, 4824, 5899+XC, 6755, 7028, 8794, 8843, 8860, 9650, 11719, 12705, 14766, 15326, 16223, 16290, 16293C, 16319
K2475	Tib_8	Kangma, Shigatse	G2b1b	73, 263, 309+C, 315+C, 489, 709, 750, 1438, 2706, 3106N, 4769, 4833, 4853, 5108, 5601, 7028, 8701, 8860, 8877, 8896, 9540, 9585, 10398, 10400, 10873, 11719, 12375, 12705, 13563, 14569, 14766, 14783, 15043, 15148, 15301, 15326, 16223, 16362
K2440	Tib_8	Kangma, Shigatse	A11a1a	73, 152, 235, 263, 309+C, 315+C, 523-524d, 663, 750, 1005, 1438, 1736, 2706, 3106N, 4248, 4769, 4824, 5899+XC, 6497, 6755, 7028, 8639, 8794, 8843, 8860, 9650, 11176, 11719, 12705, 14766, 15326, 16093, 16223, 16290, 16293C, 16319, 16519
K2433	Tib_8	Kangma, Shigatse	M9a1a1c1b1a	73, 263, 310, 489, 711, 750, 1041, 1438, 2706, 3106N, 3394, 4491, 4769, 5655, 5899+XC, 7028, 7142, 7697, 8701, 8860, 9138, 9242, 9540, 10398, 10400, 10873, 11719, 12425C, 12705, 14308, 14417, 14766, 14783, 15043, 15301, 15326, 15924, 16223, 16234, 16316, 16362
K2927	Tib_8	Kangma, Shigatse	M9a1a1c1b1a12	73, 263, 309+CC, 315+C, 489, 711, 750, 1041, 1438, 2706, 3106N, 3394, 4491, 4769, 5899+XC, 7028, 7142, 7697, 8701, 8860, 9242, 9540, 10398, 10400, 10873, 11719, 12705, 14308, 14417, 14766, 14783, 15043, 15301, 15326, 15440, 16114G, 16223, 16234, 16316, 16362
K2659	Tib_8	Kangma, Shigatse	F1c1a1a	73, 152, 249d, 263, 309+CC, 315+C, 523-524d, 573+XC, 709, 750, 1438, 2706, 3106N, 3970, 4769, 6392, 6599, 6962, 7028, 8860, 9053, 9647, 10310, 10454, 10609, 11719, 12397, 12406, 12882, 13759, 13928C, 14766, 15326, 16111, 16129, 16266, 16304, 16519
K2510	Tib_8	Kangma, Shigatse	M9a1a1c1b1a14	73, 263, 310, 489, 711, 750, 1041, 1438, 2706, 3106N, 3394, 4491, 4769, 5899+XC, 7028, 7142, 7697, 8701, 8860, 9242, 9540, 10398, 10400, 10873, 11719, 12705, 13528, 13681, 14308, 14417, 14766, 14783, 15043, 15301, 15326, 16223, 16234, 16256, 16316, 16362
K2311	Tib_8	Kangma, Shigatse	M9a1b1c	73, 150, 152, 153, 263, 310, 489, 750, 1041, 1438, 2706, 3106N, 3394, 4491, 4769, 7028, 7785, 8701, 8860, 9540, 10398, 10400, 10454, 10873, 11719, 12362, 12705, 14308, 14766, 14783, 15043, 15301, 15326, 15671, 16158, 16223, 16234, 16362, 16519
K3031	Tib_8	Kangma, Shigatse	Z4a	73, 151, 152, 249d, 263, 309+C, 315+C, 489, 750, 1438, 2706, 3106N, 4715, 4769, 6752, 7028, 7196A, 7822, 7859, 8584, 8701, 8860, 9090, 9540, 10398, 10400, 10873, 11719, 12705, 14514G, 14766, 14783, 15043, 15301, 15326, 15475, 15487T, 15727, 15784, 15944d, 16037, 16129, 16185, 16223, 16260, 16298

K2925	Tib_8	Kangma, Shigatse	F1g2	73, 195, 249d, 263, 309+CC, 315+C, 523-524d, 750, 1438, 2389, 2706, 3106N, 3398, 3970, 4769, 6392, 6962, 7028, 8860, 10310, 10609, 11719, 12406, 12882, 13928C, 14766, 15326, 16183C, 16189, 16193+C, 16304, 16519
K2869	Tib_8	Kangma, Shigatse	F1c1a1a2	73, 143, 152, 249d, 263, 309+C, 315+C, 523-524d, 573+XC, 709, 750, 1438, 2706, 3106N, 3970, 4769, 6392, 6599, 6962, 7028, 8860, 9053, 9647, 10310, 10454, 10609, 10739, 11719, 12406, 12882, 13759, 13928C, 14182, 14766, 15326, 16111, 16129, 16266, 16304, 16519
K2867	Tib_8	Kangma, Shigatse	M10a1b	73, 263, 310, 489, 573+XC, 709, 750, 1438, 2706, 3106N, 3172+C, 4140, 4769, 5393, 7028, 7250, 8701, 8793, 8856, 8860, 9540, 10398, 10400, 10646, 10873, 11719, 12549, 12705, 13152, 14502, 14766, 14783, 15040, 15043, 15071, 15218, 15301, 15326, 15461, 16066, 16223, 16311
K2864	Tib_8	Kangma, Shigatse	D4s1a	73, 199, 263, 309+C, 315+C, 489, 524+AC, 750, 813, 1438, 2706, 3010, 3106N, 4769, 4883, 5178A, 7028, 8414, 8701, 8860, 9540, 10398, 10400, 10873, 11719, 12662, 12705, 14668, 14766, 14783, 15043, 15301, 15326, 15734, 16223, 16319, 16362, 16519
K2861	Tib_8	Kangma, Shigatse	A	73, 152, 195, 235, 263, 310, 523-524d, 663, 750, 1438, 1736, 2163, 2706, 3106N, 3906, 4248, 4769, 4824, 6367, 6911, 7028, 8794, 8860, 11252, 11719, 12705, 14766, 15326, 16223, 16290, 16319, 16362
K2859	Tib_8	Kangma, Shigatse	B5b1	73, 263, 309+CC, 315+C, 523-524d, 709, 750, 960+XC, 1438, 1598, 2706, 3106N, 4418, 4769, 6101, 6719, 7028, 8281-8289d, 8584, 8784, 8829, 8860, 9950, 10398, 11719, 12361, 14384, 14527, 14766, 15077, 15223, 15326, 15508, 15662, 15851, 15927, 16140, 16182C, 16183C, 16189, 16243, 16519
K2693	Tib_8	Kangma, Shigatse	D5a2a1	51, 58, 73, 150, 199, 263, 310, 489, 523-524d, 750, 752, 930, 1107, 2706, 3106N, 4769, 4883, 5178A, 5301, 5483, 7028, 8613, 8701, 8860, 9180, 9540, 10397, 10398, 10400, 10873, 11719, 11944, 12026, 12705, 13278, 14766, 14783, 15043, 15301, 15326, 16092, 16129, 16164, 16182C, 16183C, 16189, 16223, 16266, 16362
K2687	Tib_8	Kangma, Shigatse	D5a2a	73, 150, 263, 310, 489, 523-524d, 750, 752, 1107, 2706, 3106N, 4769, 4883, 5178A, 5301, 7028, 8410, 8701, 8860, 9180, 9540, 10397, 10398, 10400, 10873, 11719, 11944, 12026, 12705, 14766, 14783, 15043, 15301, 15326, 16164, 16172, 16182C, 16183C, 16189, 16223, 16266, 16362
K2513	Tib_8	Kangma, Shigatse	G2b1a	73, 263, 310, 489, 709, 750, 1438, 2706, 3106N, 4769, 4833, 4853, 5108, 5601, 7028, 8701, 8860, 8877, 9540, 10398, 10400, 10873, 11151, 11719, 12705, 13563, 14569, 14687, 14766, 14783, 15043, 15301, 15326, 16223, 16362
K2509	Tib_8	Kangma, Shigatse	M13a2	73, 152, 263, 309+CC, 315+C, 489, 513, 750, 1438, 2706, 2786, 3106N, 3644, 4769, 5773, 6023, 6253, 6620, 6719, 7028, 8405, 8701, 8829, 8860, 9540, 10373, 10398, 10400, 10411, 10790, 10873, 11719, 12705, 13395, 14766, 14783, 15043, 15301, 15326, 15924, 15944d, 16145, 16168, 16188, 16223, 16257, 16311, 16519
K2479	Tib_8	Kangma, Shigatse	A11a1a	73, 151, 152, 235, 263, 309+C, 315+C, 523-524d, 663, 750, 1005, 1438, 1736, 2706, 3106N, 4248, 4769, 4824, 5899+XC, 6755, 7028, 8794, 8843, 8860, 9650, 11719, 12705, 14766, 14777, 15326, 16223, 16290, 16293C, 16319
K2436	Tib_8	Kangma, Shigatse	D4q	73, 200, 204, 263, 309+C, 315+C, 489, 750, 1438, 2706, 3010, 3106N, 4769, 4883, 5178A, 7028, 8414, 8701, 8860, 9540, 10398, 10400, 10873, 11719, 12705, 14668, 14766, 14783, 15043, 15301, 15326, 16223, 16256, 16261, 16311, 16362, 16519
K2435	Tib_8	Kangma, Shigatse	M13a2a1d	73, 152, 263, 309+C, 315+C, 489, 513, 750, 1393, 1438, 2706, 2786, 3106N, 3644, 4769, 4928, 5773, 6023, 6253, 6620, 7028, 8405, 8701, 8860, 9540, 10373, 10398, 10400, 10411, 10790, 10873, 11719, 12705, 13395, 14100, 14766, 14783, 15043, 15301, 15326, 15924, 15944d, 16145, 16168, 16188, 16223, 16257, 16311, 16519
K2241	Tib_8	Kangma, Shigatse	F1g	73, 150, 249d, 263, 309+CCC, 315+C, 750, 980, 1438, 2389, 2706, 3106N, 3398, 3970, 4769, 6392, 6962, 7028, 7754, 8860, 10310, 10609, 10792, 11719, 12406, 12882, 13928C, 14766, 15310, 15326, 16183C, 16189, 16193+CC, 16304, 16519
K2238	Tib_8	Kangma, Shigatse	D4h1c2a	73, 152, 195, 241, 263, 310, 489, 709, 750, 1438, 2706, 3010, 3106N, 3336, 3644, 3645, 3834C, 4182, 4769, 4883, 5048, 5178A, 6881, 7028, 7181, 7673, 8179, 8414, 8701, 8860, 9540, 10398, 10400, 10873, 11719, 12705, 13914A, 14668, 14766, 14783, 15043, 15301, 15326, 16174, 16223, 16311, 16362
K2186	Tib_8	Kangma, Shigatse	F1c1a1a	73, 152, 249d, 263, 309+CC, 315+C, 523-524d, 573+XC, 709, 750, 1438, 2706, 3106N, 3970, 4769, 6392, 6599, 6962, 7028, 8860, 9053, 9647, 10310, 10454, 10609, 11719, 12397, 12406, 12882, 13759, 13928C, 14766, 15080, 15326, 16111, 16129, 16266, 16304, 16519
K2170	Tib_8	Kangma, Shigatse	D4o2a	73, 195, 263, 309+C, 315+C, 489, 750, 1438, 2706, 3010, 3106N, 3789, 4769, 4883, 5178A, 7028, 8414, 8701, 8860, 9077, 9540, 10398, 10400, 10646, 10873, 11719, 12705, 13812, 14668, 14766, 14783, 15043, 15301, 15326, 16093, 16223, 16232, 16290, 16362, 16519
K2168	Tib_8	Kangma, Shigatse	M9a1a1c1b1a	73, 263, 309+CC, 315+C, 489, 711, 750, 1041, 1438, 2706, 3106N, 3394, 4491, 4769, 5899+XC, 7028, 7142, 7697, 8701, 8860, 9242, 9540, 10398, 10400, 10873, 11719, 12705, 14308, 14417, 14766, 14783, 15043, 15301, 15326, 16223, 16234, 16316, 16362
K2164	Tib_8	Kangma, Shigatse	M13a2a1d	73, 152, 263, 309+C, 315+C, 489, 513, 750, 1393, 1438, 2706, 2786, 3106N, 3644, 4769, 4928, 5773, 6023, 6253, 6620, 7028, 8405, 8701, 8860, 9540, 10373, 10398, 10400, 10411, 10790, 10873, 11719, 12705, 13395, 14100, 14766, 14783, 15043, 15301, 15326, 15924, 15944d, 16145, 16168, 16188, 16223, 16257, 16311, 16519

K2689	Tib_8	Kangma, Shigatse	M13a1b	73, 152, 263, 310, 489, 750, 1438, 1836, 2706, 3106N, 3644, 4769, 5773, 6023, 6253, 6620, 7028, 8701, 8860, 9540, 10398, 10400, 10411, 10790, 10873, 11719, 12705, 13135, 13708, 14766, 14783, 15043, 15301, 15326, 15924, 16145, 16148, 16188, 16189, 16223, 16381
K2658	Tib_8	Kangma, Shigatse	M13a2a1c	73, 152, 263, 309+C, 315+C, 489, 513, 745+T, 750, 1438, 2706, 2786, 3106N, 3644, 4769, 5773, 6023, 6253, 6620, 7028, 7147, 7679, 8405, 8563, 8701, 8860, 9540, 10373, 10398, 10400, 10411, 10790, 10873, 11204, 11719, 12705, 13395, 14766, 14783, 15043, 15236, 15301, 15326, 15891, 15924, 15944d, 16145, 16168, 16188, 16223, 16257, 16311, 16519
K2438	Tib_8	Kangma, Shigatse	D4	73, 263, 309+C, 315+C, 489, 750, 1438, 2706, 3010, 3106N, 4769, 4883, 5178A, 6497, 7028, 8414, 8701, 8860, 9540, 9812, 10398, 10400, 10601, 10873, 11719, 12705, 13153, 14668, 14766, 14783, 14883, 15043, 15301, 15326, 15884, 16223, 16362
K2872	Tib_8	Kangma, Shigatse	M8a2a1	73, 263, 309+C, 315+C, 489, 750, 827, 1438, 2706, 2835, 3106N, 4715, 4769, 6179, 7028, 7196A, 8245, 8584, 8684, 8701, 8860, 9540, 10398, 10400, 10873, 11176, 11719, 12705, 14208, 14470, 14766, 14783, 15043, 15301, 15326, 15487T, 16147G, 16184, 16189, 16223, 16298, 16319, 16468, 16470, 16471, 16473
K2860	Tib_8	Kangma, Shigatse	M9a1a1c1b1a	73, 263, 309+CC, 315+C, 489, 711, 750, 1041, 1438, 2706, 3106N, 3394, 4491, 4769, 5899+XC, 7028, 7142, 7697, 8701, 8860, 9242, 9540, 10398, 10400, 10873, 11719, 12705, 14308, 14417, 14766, 14783, 15043, 15301, 15326, 16223, 16234, 16316, 16362
K2439	Tib_8	Kangma, Shigatse	A11a1a	73, 152, 235, 263, 309+C, 315+C, 523-524d, 663, 750, 1005, 1438, 1736, 2706, 3106N, 4248, 4769, 4824, 5899+XC, 6755, 7028, 8794, 8843, 8860, 9650, 11719, 12528, 12705, 14766, 15326, 16093, 16223, 16290, 16293C, 16319, 16519
K2511	Tib_8	Kangma, Shigatse	A11b	73, 152, 235, 263, 309+C, 315+C, 523-524d, 663, 750, 1092, 1438, 1736, 2706, 3106N, 3290, 4248, 4769, 4824, 7028, 8794, 8860, 9650, 11719, 12705, 13676, 14180, 14766, 15326, 16223, 16234, 16290, 16293C, 16311, 16319, 16519, 16527
K2434	Tib_8	Kangma, Shigatse	A21b1a	63, 64, 66T, 73, 152, 235, 263, 309+C, 315+C, 663, 750, 1047, 1438, 1736, 2706, 3106N, 4248, 4769, 4824, 7028, 8794, 8860, 11719, 12705, 13934, 14364, 14766, 15326, 16092, 16223, 16290, 16319, 16362
K2236	Tib_8	Kangma, Shigatse	D4b2b8	73, 194, 263, 309+CC, 315+C, 390, 489, 523-524d, 750, 1382C, 1438, 2706, 3010, 3106N, 3398, 4769, 4883, 5178A, 7028, 8020, 8414, 8701, 8860, 8964, 9296, 9540, 9824A, 10398, 10400, 10873, 11719, 12705, 14668, 14766, 14783, 15043, 15301, 15326, 15613, 16223, 16292, 16362, 16519
K2314	Tib_8	Kangma, Shigatse	F1b1	73, 249d, 263, 310, 523-524d, 750, 1438, 2706, 2757, 3106N, 3970, 4732, 4769, 5147, 6392, 6962, 7028, 8860, 10310, 10609, 10976, 11719, 12406, 12633, 12882, 13444G, 13928C, 14476, 14766, 15326, 16183C, 16189, 16232A, 16249, 16311, 16519
K2239	Tib_8	Kangma, Shigatse	F1c1a1a	73, 152, 185, 249d, 263, 309+C, 315+C, 523-524d, 573+XC, 709, 750, 1438, 2706, 3106N, 3970, 4769, 6392, 6599, 6962, 7028, 8860, 9053, 9531, 9647, 10310, 10454, 10609, 11719, 12406, 12882, 13759, 13928C, 14766, 15326, 16111, 16129, 16266, 16304, 16519
K2579	Tib_8	Kangma, Shigatse	G2a1f	73, 263, 309+C, 315+C, 489, 709, 750, 1438, 2386A, 2706, 3106N, 4769, 4833, 5108, 5601, 7028, 7600, 8701, 8860, 9377, 9540, 9575, 9659, 10398, 10400, 10873, 11719, 12705, 13563, 13884, 14200, 14569, 14766, 14783, 15043, 15301, 15326, 16051, 16114, 16223, 16227, 16278, 16291, 16362
K2306	Tib_8	Kangma, Shigatse	G2b1b	73, 263, 309+C, 315+C, 489, 709, 750, 1438, 2706, 3106N, 4769, 4833, 4853, 5108, 5601, 7028, 8701, 8860, 8877, 8896, 9540, 9585, 10398, 10400, 10873, 11719, 12375, 12705, 13563, 14569, 14766, 14783, 15043, 15148, 15301, 15326, 16223, 16362
K2874	Tib_8	Kangma, Shigatse	M9a1a1c1b1a	73, 263, 310, 489, 711, 750, 1041, 1438, 2706, 3106N, 3394, 4491, 4769, 5899+XC, 7028, 7142, 7697, 8701, 8860, 9242, 9540, 10398, 10400, 10873, 11719, 12705, 14308, 14417, 14766, 14783, 15043, 15301, 15326, 16163, 16223, 16234, 16316, 16362
K2868	Tib_8	Kangma, Shigatse	M9a1a1c1b1a	73, 263, 309+CC, 315+C, 489, 711, 750, 1041, 1438, 2706, 3106N, 3394, 4491, 4769, 5618, 5899+XC, 7028, 7142, 7697, 8701, 8860, 9242, 9540, 10398, 10400, 10873, 11719, 12705, 14308, 14417, 14766, 14783, 15043, 15301, 15326, 16223, 16234, 16316, 16362
K2242	Tib_8	Kangma, Shigatse	M9a1a1c1b1a	73, 263, 309+CC, 315+C, 489, 711, 750, 1041, 1438, 2706, 3106N, 3394, 4077, 4491, 4769, 5899+XC, 7028, 7142, 7697, 8701, 8860, 9242, 9540, 10398, 10400, 10873, 11719, 12192, 12705, 14308, 14417, 14766, 14783, 15043, 15301, 15326, 16223, 16234, 16316, 16362
K2237	Tib_8	Kangma, Shigatse	M9a1a1c1b1a12	73, 263, 309+C, 315+C, 489, 711, 750, 1041, 1438, 2706, 3106N, 3394, 3397, 4491, 4769, 5899+XC, 7028, 7142, 7697, 8701, 8860, 9242, 9540, 10398, 10400, 10873, 11719, 12705, 13113, 14308, 14417, 14766, 14783, 15043, 15301, 15326, 16114G, 16223, 16234, 16311, 16316, 16362
K2688	Tib_8	Kangma, Shigatse	M9a1a1c1b1a1	73, 263, 309+C, 315+C, 489, 711, 750, 1041, 1438, 2706, 3106N, 3394, 4491, 4769, 5899+XC, 7028, 7142, 7697, 8567, 8701, 8860, 9242, 9540, 10398, 10400, 10873, 11719, 11908, 12705, 14308, 14417, 14514, 14766, 14783, 15043, 15301, 15326, 16223, 16234, 16316, 16362
K2305	Tib_8	Kangma, Shigatse	A11a1a	73, 152, 235, 263, 310, 523-524d, 663, 750, 1005, 1438, 1736, 2706, 3106N, 4248, 4769, 4824, 5414, 5899+XC, 6497, 6755, 7028, 7674, 8794, 8843, 8860, 9650, 11719, 12705, 14766, 15326, 16093, 16223, 16290, 16293C, 16319, 16519
K2312	Tib_8	Kangma, Shigatse	A15a	73, 152, 204, 207, 235, 309+CC, 315+C, 523-524d, 663, 750, 1438, 1736, 2706, 3106N, 4248, 4769, 4824, 7028, 8459, 8794, 8860, 11084, 11719, 12705, 14067, 14569, 14766, 14831, 15326, 16223, 16274, 16290, 16319, 16362

K2304	Tib_8	Kangma, Shigatse	C4a1a1a2	73, 146, 249d, 263, 310, 489, 750, 1438, 1715, 2232+AA, 2706, 3106N, 3397, 3552A, 3576, 4715, 4769, 4884, 4958, 5918, 6026, 7028, 7196A, 7999, 8508, 8584, 8701, 8860, 9540, 9545, 10398, 10400, 10873, 11719, 11914, 11969, 12672, 12705, 13263, 14318, 14766, 14783, 15043, 15204, 15301, 15326, 15487T, 15968, 16093, 16129, 16223, 16298, 16327, 16519
K2661	Tib_8	Kangma, Shigatse	C4d2	73, 152, 195, 249d, 263, 310, 489, 750, 1438, 2706, 3106N, 3552A, 4715, 4769, 4907, 6026, 7028, 7100, 7196A, 8584, 8701, 8860, 9540, 9545, 10398, 10400, 10873, 11719, 11914, 11969, 12705, 12780, 13263, 14318, 14766, 14783, 15043, 15204, 15236, 15301, 15326, 15487T, 16093, 16298, 16327, 16519
K2313	Tib_8	Kangma, Shigatse	D4j1b	73, 185, 263, 309+C, 315+C, 489, 750, 1438, 1734, 2706, 3010, 3106N, 4300, 4769, 4883, 5178A, 5262, 7028, 7055, 8414, 8701, 8860, 9540, 10398, 10400, 10873, 11696, 11719, 12705, 14668, 14766, 14783, 15043, 15301, 15326, 16223, 16362
K2581	Tib_8	Kangma, Shigatse	F1d1a2a1	73, 146, 234, 249d, 263, 309+CC, 315+C, 523-524d, 750, 1438, 1734, 1888, 2706, 3106N, 3970, 4769, 5628, 6392, 6962, 7028, 7738, 8167, 8860, 10310, 10609, 11719, 12372, 12406, 12882, 13135, 13928C, 14766, 15326, 15402, 16145, 16255, 16284, 16304, 16519
K2442	Tib_8	Kangma, Shigatse	G2a1i	73, 195, 198, 263, 278, 310, 489, 709, 750, 1438, 2706, 3106N, 4769, 4833, 5108, 5601, 7028, 7600, 8701, 8860, 9377, 9540, 9575, 10398, 10400, 10873, 11719, 12705, 13563, 14200, 14569, 14766, 14783, 15043, 15301, 15326, 16129, 16223, 16278, 16362
K2690	Tib_8	Kangma, Shigatse	H2	146, 263, 310, 750, 1438, 3106N, 4769, 8281-8289d, 8860, 13314, 14022, 15326, 16172, 16186, 16519
K2660	Tib_8	Kangma, Shigatse	H2	146, 263, 310, 750, 1438, 3106N, 4769, 8281-8289d, 8860, 13314, 14022, 15326, 16172, 16186, 16519
K2865	Tib_8	Kangma, Shigatse	M10a1b	73, 263, 310, 489, 573+XC, 709, 750, 1438, 2706, 3106N, 3172+C, 4140, 4769, 5393, 7028, 7250, 8701, 8793, 8856, 8860, 9540, 10398, 10400, 10646, 10873, 11719, 12549, 12705, 13152, 13759, 14502, 14766, 14783, 15040, 15043, 15071, 15218, 15301, 15326, 16066, 16223, 16311
K2512	Tib_8	Kangma, Shigatse	M11a	73, 198, 200, 215, 263, 309+C, 315+C, 318, 326, 489, 750, 1095, 1438, 2706, 3106N, 4069, 4769, 5993, 6531, 7028, 7642, 8108, 8701, 8860, 9540, 9950, 10398, 10400, 10873, 11719, 11969, 12705, 13074, 14340, 14766, 14783, 15043, 15301, 15326, 16223, 16320
K2441	Tib_8	Kangma, Shigatse	M9a1b1c	73, 150, 152, 153, 263, 310, 489, 750, 1041, 1438, 2706, 3106N, 3394, 4491, 4769, 7028, 8701, 8860, 9540, 10398, 10400, 10454, 10873, 11719, 12362, 12705, 14308, 14766, 14783, 15043, 15301, 15326, 15671, 16158, 16223, 16234, 16362, 16519
K2315	Tib_8	Kangma, Shigatse	M9a1b1c	73, 150, 152, 153, 183, 263, 309+C, 315+C, 489, 750, 1041, 1438, 2706, 3106N, 3394, 4491, 4769, 7028, 8701, 8860, 9540, 10398, 10400, 10454, 10873, 11719, 12362, 12705, 14308, 14766, 14783, 15043, 15301, 15326, 15671, 16158, 16223, 16234, 16362, 16519
K2508	Tib_8	Kangma, Shigatse	Z3a1a	73, 152, 189, 204, 207, 249d, 263, 309+C, 315+C, 489, 524+AC, 573+XC, 750, 1438, 2706, 3106N, 4715, 4769, 6752, 7028, 7196A, 7702, 8584, 8701, 8860, 8931, 9090, 9540, 9713, 10208, 10398, 10400, 10873, 11075, 11719, 12705, 13359, 13620, 14766, 14783, 14841, 15043, 15301, 15487T, 15784, 15928, 16185, 16223, 16260
K2167	Tib_8	Kangma, Shigatse	A21a	73, 152, 235, 263, 310, 663, 750, 1438, 1736, 2706, 3106N, 4248, 4769, 4824, 7028, 8794, 8860, 11719, 12705, 14364, 14766, 15326, 16092, 16167, 16223, 16290, 16319, 16362
K2188	Tib_8	Kangma, Shigatse	A	73, 152, 235, 263, 309+C, 315+C, 523-524d, 663, 750, 1438, 1736, 2706, 3106N, 4248, 4769, 4824, 7028, 7073, 8794, 8860, 11699, 11719, 12705, 14766, 15326, 16223, 16290, 16319, 16362
K2926	Tib_8	Kangma, Shigatse	A21b1a	66T, 73, 152, 235, 263, 309+C, 315+C, 663, 750, 1438, 1736, 2706, 3106N, 4248, 4769, 4824, 7028, 8794, 8860, 11719, 12705, 13934, 12990, 14766, 15326, 16092, 16223, 16290, 16319, 16362
K2862	Tib_8	Kangma, Shigatse	A21b	73, 152, 235, 263, 309+C, 315+C, 663, 750, 1438, 1736, 2706, 3106N, 4248, 4769, 4824, 6260, 7028, 8794, 8860, 11719, 12705, 13934, 14364, 14766, 15326, 16223, 16290, 16319, 16362
K2192	Tib_8	Kangma, Shigatse	B4a	73, 263, 309+CC, 315+C, 523-524d, 750, 1438, 2706, 3106N, 3396, 4769, 5465, 7028, 8281-8289d, 8860, 9123, 11719, 12634, 13810, 14766, 15326, 16182C, 16183C, 16189, 16217, 16219, 16261, 16286, 16519
K2477	Tib_8	Kangma, Shigatse	F1c1a1a2	73, 143, 152, 249d, 263, 309+C, 315+C, 523-524d, 573+XC, 709, 750, 1438, 2706, 3106N, 3970, 4769, 6392, 6599, 6962, 7028, 8860, 9053, 9647, 10310, 10454, 10609, 10739, 11719, 12406, 12882, 13759, 13928C, 14766, 15326, 16094, 16111, 16129, 16266, 16304, 16519
K2858	Tib_8	Kangma, Shigatse	M62a1a1	73, 146, 150, 263, 489, 524+AC, 709, 750, 1438, 2706, 2735, 3106N, 3511, 4561, 4763, 4769, 7028, 7664, 8149, 8701, 8860, 9540, 9935, 10398, 10400, 10873, 11719, 12705, 13708, 14766, 14783, 15043, 15301, 15326, 15510, 15520, 15629, 15721, 16147, 16183C, 16189, 16193+C, 16215, 16223, 16295, 16452, 16487, 16491, 16519

K2577	Tib_8	Kangma, Shigatse	M9a1a1c1b1a13	73, 263, 309+C, 315+C, 489, 711, 750, 1041, 1438, 2706, 3106N, 3394, 3866, 4491, 4769, 5899+XC, 7028, 7142, 7697, 8701, 8860, 9242, 9540, 10398, 10400, 10873, 11719, 12705, 13725, 14308, 14417, 14766, 14783, 15043, 15301, 15326, 15634, 16194, 16223, 16234, 16316, 16362
K2437	Tib_8	Kangma, Shigatse	M9a1b2	73, 150, 152, 153, 263, 309+C, 315+C, 489, 750, 1041, 1438, 2706, 3106N, 3394, , 4491, 4769, 5592, 5899+XC, 7028, 8701, 8860, 9540, 10398, 10400, 10873, 10951A, 11719, 12362, 12705, 14308, 14766, 14783, 15043, 15301, 15326, 15671, 16223, 16362, 16519
K2443	Tib_8	Kangma, Shigatse	F1b1	73, 249d, 263, 310, 523-524d, 750, 1438, 2706, 2757, 3106N, 3970, 4732, 4769, 5147, 6392, 6962, 7028, 8860, 10310, 10609, 10976, 11719, 12406, 12633, 12882, 13444G, 13928C, 14476, 14766, 15326, 16183C, 16189, 16232A, 16249, 16311, 16519
K2189	Tib_8	Kangma, Shigatse	M9a1a1c1b1a	73, 263, 309+CC, 315+C, 489, 711, 750, 1041, 1438, 2706, 3106N, 3394, 4491, 4769, 5894C, 5899+XC, 7028, 7142, 7697, 8701, 8860, 9242, 9540, 10398, 10400, 10873, 11719, 12705, 14308, 14417, 14766, 14783, 15043, 15301, 15326, 16223, 16234, 16316, 16362
K3592	Tib_9	Lazi, Shigatse	G2a1h	73, 263, 309+C, 315+C, 489, 593, 709, 750, 1438, 2404, 2706, 3106N, 3158+T, 3777, 4769, 5108, 5601, 7028, 7600, 8307, 8701, 8860, 9377, 9540, 9575, 10398, 10400, 10873, 11719, 12705, 13194, 13563, 14200, 14569, 14766, 14783, 15043, 15301, 15326, 16223, 16227, 16278, 16362
K3541	Tib_9	Lazi, Shigatse	A11a1a	73, 152, 235, 263, 310, 523-524d, 663, 750, 1005, 1438, 1736, 2706, 3106N, 4248, 4769, 4824, 5899+XC, 6755, 7028, 8281-8289d, 8794, 8843, 8860, 9145, 9650, 11719, 12705, 14766, 15326, 16093, 16223, 16290, 16293C, 16294, 16319, 16527
K3371	Tib_9	Lazi, Shigatse	C4a2c	73, 214, 249d, 263, 310, 489, 750, 1438, 2232+A, 2706, 3106N, 3552A, 3565, 4216, 4715, 4769, 6026, 6647, 7028, 7196A, 7664, 8062, 8584, 8701, 8860, 9540, 9545, 10398, 10400, 10873, 11719, 11914, 11969, 12672, 12705, 13263, 13967, 14318, 14766, 14783, 15043, 15204, 15301, 15326, 15487T, 16223, 16298, 16327, 16357, 16519
K3302	Tib_9	Lazi, Shigatse	A21b1	73, 152, 235, 263, 310, 663, 750, 1438, 1736, 2706, 3106N, 4248, 4769, 4824, 7028, 8794, 8860, 11719, 12705, 13934, 14364, 14766, 15326, 16092, 16223, 16290, 16319, 16362
K3296	Tib_9	Lazi, Shigatse	A15c2	73, 152, 153, 235, 310, 523-524d, 663, 750, 1438, 1736, 2706, 3106N, 4248, 4769, 4824, 7028, 8459, 8794, 8860, 9052, 11719, 12705, 13111, 14067, 14766, 15262, 15326, 16223, 16290, 16319, 16362
K3293	Tib_9	Lazi, Shigatse	A21b1	73, 152, 235, 263, 310, 663, 750, 1438, 1736, 2706, 3106N, 4248, 4769, 4824, 7028, 8794, 8860, 9142, 11719, 12705, 13934, 14364, 14766, 15326, 16092, 16223, 16290, 16319, 16362
K3111	Tib_9	Lazi, Shigatse	M9a1a1c1b1a	73, 263, 309+CC, 315+C, 489, 711, 750, 1041, 1438, 2706, 3106N, 3394, 4491, 4769, 5605, 5899+XC, 7028, 7142, 7352, 7697, 8701, 8860, 9242, 9540, 10398, 10400, 10873, 11719, 12705, 13943, 14308, 14417, 14766, 14783, 15043, 15301, 15326, 16223, 16234, 16316, 16362
K3110	Tib_9	Lazi, Shigatse	M9a1a1c1b1a12	73, 263, 309+C, 315+C, 489, 711, 750, 1041, 1438, 2706, 3106N, 3394, 4491, 4769, 5899+XC, 7028, 7142, 7697, 8701, 8860, 9242, 9540, 10398, 10400, 10873, 11719, 12705, 14308, 14417, 14766, 14783, 15043, 15301, 15326, 15440, 16114G, 16223, 16234, 16316, 16362
K2742	Tib_9	Lazi, Shigatse	T1a1	73, 152, 195, 263, 709, 750, 1438, 1888, 2706, 3106N, 4216, 4769, 4917, 7028, 8697, 8860, 9899, 10463, 11251, 11719, 12633A, 13368, 14766, 14905, 15326, 15452A, 15607, 15928, 16126, 16163, 16186, 16189, 16294, 16519
K2685	Tib_9	Lazi, Shigatse	M13a2	73, 152, 263, 309+C, 315+C, 489, 513, 750, 1438, 2706, 2786, 3105, 3106N, 3644, 4769, 5773, 6023, 6253, 6620, 7028, 8405, 8701, 8860, 9540, 10373, 10398, 10400, 10411, 10790, 10873, 11719, 12705, 13395, 14766, 14783, 15043, 15301, 15326, 15924, 15944d, 16145, 16168, 16188, 16189, 16223, 16257, 16311, 16519
K2684	Tib_9	Lazi, Shigatse	U2c1	73, 125, 127, 128, 152, 263, 310, 523-524d, 750, 1438, 1811, 2706, 3106N, 3796, 4769, 5790A, 7028, 7853, 8023, 8676, 8860, 9156, 9767, 10682T, 11467, 11719, 12308, 12372, 13488, 14766, 14935, 15061, 15326, 16051, 16093, 16220, 16234, 16247, 16311, 16519, 16527
K3607	Tib_9	Lazi, Shigatse	C4a2c3	47, 73, 214, 249d, 263, 309+C, 315+C, 489, 750, 1438, 2232+A, 2706, 3106N, 3552A, 3565, 4715, 4769, 6026, 6647, 7028, 7196A, 7664, 8062, 8584, 8701, 8860, 9540, 9545, 10398, 10400, 10873, 11719, 11914, 11969, 12672, 12705, 13263, 13967, 14318, 14693, 14766, 14783, 15043, 15204, 15301, 15326, 15487T, 16223, 16298, 16327, 16357, 16519
K3606	Tib_9	Lazi, Shigatse	D4o2a	73, 195, 263, 309+C, 315+C, 489, 750, 1438, 2706, 3010, 3106N, 3789, 4769, 4883, 5178A, 7028, 8414, 8701, 8860, 9077, 9540, 10398, 10400, 10646, 10873, 11719, 12705, 13812, 14668, 14766, 14783, 15043, 15301, 15326, 16093, 16223, 16232, 16290, 16362, 16519
K3602	Tib_9	Lazi, Shigatse	F1d1a1	73, 146, 249d, 263, 309+C, 315+C, 523-524d, 750, 1438, 1734, 2706, 3106N, 3970, 4769, 5628, 6392, 6962, 7028, 7738, 8860, 8994, 10310, 10609, 11719, 12007, 12406, 12882, 13135, 13928C, 14766, 15326, 15402, 16265C, 16284, 16304, 16519
K3600	Tib_9	Lazi, Shigatse	F1c1a1a	73, 151, 152, 249d, 263, 309+C, 315+C, 523-524d, 573+XC, 709, 750, 1438, 2706, 3106N, 3970, 4769, 6392, 6599, 6962, 7028, 8860, 9053, 9647, 10310, 10454, 10609, 11719, 12406, 12882, 13759, 13928C, 14766, 15326, 16111, 16129, 16266, 16304, 16519

K3599	Tib_9	Lazi, Shigatse	M9a1a1c1b1a13	54C, 73, 263, 310, 489, 711, 750, 1041, 1438, 2706, 3106N, 3394, 3866, 4491, 4769, 5899+XC, 7028, 7142, 7697, 8701, 8860, 9242, 9540, 10398, 10400, 10873, 11719, 12705, 14308, 14417, 14766, 14783, 15043, 15301, 15326, 16155, 16223, 16234, 16316, 16362
K3591	Tib_9	Lazi, Shigatse	M33a1a	73, 195, 263, 309+C, 315+C, 489, 573+XC, 750, 1438, 2361, 2706, 3106N, 4769, 7028, 8562, 8572, 8701, 8860, 9540, 10398, 10400, 10873, 11719, 12153, 12705, 14053, 14766, 14783, 15043, 15052, 15301, 15326, 15466, 15862, 15908, 16223, 16294, 16519
K3551	Tib_9	Lazi, Shigatse	C4a2b2	73, 194, 249d, 263, 489, 750, 1438, 1677, 2232+A, 2706, 2887, 3106N, 3552A, 4696, 4715, 4769, 6026, 7028, 7196A, 8584, 8701, 8860, 9540, 9545, 10304, 10398, 10400, 10873, 11719, 11914, 11969, 12624, 12672, 12705, 13263, 14318, 14766, 14783, 15043, 15204, 15301, 15326, 15487T, 16223, 16298, 16311, 16327, 16357, 16519
K3548	Tib_9	Lazi, Shigatse	C7a1	73, 146, 153, 249d, 263, 309+C, 315+C, 489, 750, 1438, 2706, 3106N, 3552A, 3918, 4715, 4769, 5790, 5821, 6338, 6524, 7028, 7196A, 7853, 8584, 8701, 8860, 9540, 9545, 10398, 10400, 10873, 11719, 11914, 12705, 12957, 13263, 14318, 14766, 14783, 14978, 15043, 15301, 15326, 15487T, 16223, 16239, 16298, 16327, 16519
K3545	Tib_9	Lazi, Shigatse	A21b1a	63, 64, 66T, 73, 152, 235, 263, 309+C, 315+C, 663, 750, 1047, 1438, 1736, 2706, 3106N, 4248, 4769, 4824, 7028, 8794, 8860, 11719, 12705, 13934, 14364, 14766, 15326, 16092, 16223, 16290, 16319, 16362
K3542	Tib_9	Lazi, Shigatse	A15c2	73, 152, 235, 309+CC, 315+C, 523-524d, 663, 750, 1438, 1736, 2706, 3106N, 4226, 4248, 4769, 4824, 7028, 8459, 8794, 8860, 9052, 11719, 12705, 13111, 14067, 14766, 15262, 15326, 16223, 16290, 16295, 16319, 16362
K3300	Tib_9	Lazi, Shigatse	M62a1a	73, 146, 150, 263, 489, 750, 1438, 2706, 2735, 3106N, 3511, 4561, 4763, 4769, 7028, 7664, 8149, 8701, 8860, 9540, 9935, 10398, 10400, 10873, 11719, 12705, 13708, 14766, 14783, 15043, 15301, 15326, 15510, 15520, 15629, 15721, 16147, 16223, 16284, 16295, 16368, 16452, 16484, 16487, 16491, 16519
K2973	Tib_9	Lazi, Shigatse	M13a2a1d	73, 152, 263, 310, 489, 513, 750, 1393, 1438, 2706, 2786, 3106N, 3644, 4769, 5773, 6023, 6253, 6620, 7028, 8405, 8701, 8860, 9540, 10373, 10398, 10400, 10411, 10790, 10873, 11719, 12705, 13395, 14766, 14783, 15043, 15301, 15326, 15334, 15924, 15944d, 16145, 16168, 16188, 16223, 16257, 16311, 16519
K2743	Tib_9	Lazi, Shigatse	D4j1a1c	73, 263, 310, 489, 750, 1438, 2706, 3010, 3106N, 3397, 4769, 4883, 5178A, 5262, 7028, 7581, 7783, 8414, 8701, 8860, 9540, 10398, 10400, 10873, 11696, 11719, 12130, 12358, 12705, 14668, 14766, 14783, 15043, 15295, 15301, 15326, 15884, 16086, 16223, 16362
K2748	Tib_9	Lazi, Shigatse	F1c1a1a3	73, 152, 249d, 263, 309+C, 315+C, 523-524d, 573+XC, 709, 750, 1438, 2706, 3106N, 3970, 4769, 6392, 6599, 6962, 7028, 8860, 9053, 9389, 9647, 10310, 10454, 10609, 11719, 12406, 12882, 13759, 13928C, 14766, 15326, 15758, 16111, 16129, 16266, 16304, 16362, 16519
K3108	Tib_9	Lazi, Shigatse	D6a1	73, 263, 309+C, 315+C, 489, 709, 750, 1438, 1719, 2706, 3106N, 3714, 4769, 4883, 5178A, 6701, 7028, 7424, 7879, 8701, 8860, 9540, 10398, 10400, 10873, 11719, 12654, 12705, 13194, 14766, 14783, 15043, 15301, 15326, 16189, 16193+TC, 16223, 16274, 16362
K2971	Tib_9	Lazi, Shigatse	M9a1a1c1b1a	73, 263, 309+C, 315+C, 489, 711, 750, 1041, 1438, 2706, 3106N, 3394, 4491, 4769, 5899+XC, 5978, 7028, 7142, 7697, 8701, 8860, 9242, 9540, 10398, 10400, 10873, 11719, 12705, 14308, 14417, 14766, 14783, 15043, 15301, 15326, 16223, 16234, 16316, 16362
K3543	Tib_9	Lazi, Shigatse	B4c1b2c2	73, 150, 263, 309+C, 315+C, 709, 750, 1119, 1438, 2706, 3106N, 3435, 3497, 3571, 4769, 7028, 8281-8289d, 8860, 9128, 9770, 11204, 11440, 11719, 14311, 14766, 14945, 15326, 15346, 16051, 16129, 16140, 16166, 16186+T, 16189, 16217, 16274, 16335, 16519
K3605	Tib_9	Lazi, Shigatse	C4a1a1a2	73, 249d, 263, 310, 489, 750, 1438, 1715, 2232+AA, 2706, 3106N, 3552A, 3576, 4715, 4769, 4884, 4958, 5918, 6026, 7028, 7196A, 7999, 8508, 8584, 8701, 8860, 9540, 9545, 10398, 10400, 10873, 11719, 11914, 11969, 12672, 12705, 13263, 14318, 14766, 14783, 15043, 15204, 15301, 15326, 15487T, 15968, 16093, 16129, 16223, 16298, 16327, 16519
K3109	Tib_9	Lazi, Shigatse	C4a2c3	47, 73, 214, 249d, 263, 309+C, 315+C, 489, 750, 1438, 2232+A, 2706, 3106N, 3552A, 3565, 4715, 4769, 6026, 6647, 7028, 7196A, 7664, 8062, 8584, 8701, 8860, 9540, 9545, 10398, 10400, 10873, 11719, 11914, 11969, 12672, 12705, 13263, 13967, 14318, 14693, 14766, 14783, 15043, 15204, 15301, 15326, 15487T, 16223, 16298, 16327, 16357, 16519
K3294	Tib_9	Lazi, Shigatse	C4d2	73, 152, 207, 249d, 263, 310, 489, 750, 1438, 2706, 3106N, 3552A, 4715, 4769, 6026, 6261, 7028, 7100, 7196A, 8584, 8701, 8860, 9540, 9545, 10398, 10400, 10873, 10915, 11719, 11914, 11969, 12705, 12780, 13263, 14318, 14766, 14783, 15043, 15204, 15236, 15301, 15326, 15487T, 16093, 16298, 16327, 16519
K3842	Tib_9	Lazi, Shigatse	D4j1a1	73, 263, 310, 489, 750, 1438, 2706, 3010, 3106N, 4769, 4883, 5178A, 5262, 6383, 7028, 7375, 7581, 7783, 8414, 8701, 8860, 9053, 9540, 10398, 10400, 10873, 10933, 11696, 11719, 11963, 12130, 12358, 12705, 14668, 14766, 14783, 15043, 15295, 15301, 15326, 16086, 16223, 16362
K3549	Tib_9	Lazi, Shigatse	F1d1a2a	73, 146, 234, 249d, 263, 309+C, 315+C, 523-524d, 750, 1438, 1734, 2706, 3106N, 3970, 4058A, 4769, 5628, 6392, 6962, 7028, 7738, 8860, 10310, 10609, 11719, 12406, 12882, 13135, 13928C, 14766, 15326, 15402, 16145, 16183C, 16189, 16255, 16284, 16304, 16519

K3597	Tib_9	Lazi, Shigatse	G2b1b2	73, 263, 309+CC, 315+C, 489, 709, 750, 1438, 2706, 3106N, 3975, 4769, 4833, 4853, 5108, 5601, 7028, 8701, 8860, 8877, 9540, 10398, 10400, 10873, 11719, 12375, 12705, 12795, 13563, 14563G, 14569, 14766, 14783, 15043, 15301, 15326, 16223, 16362
K3754	Tib_9	Lazi, Shigatse	M9a1a1c1b1a	73, 263, 309+C, 315+C, 489, 711, 750, 1041, 1438, 2706, 3106N, 3394, 4491, 4769, 5899+XC, 7028, 7142, 7371, 7697, 8701, 8860, 9242, 9540, 10398, 10400, 10873, 11719, 12705, 14308, 14417, 14766, 14783, 15043, 15301, 15326, 16148, 16223, 16234, 16274, 16316, 16362
K3753	Tib_9	Lazi, Shigatse	M9a1a1c1b1a	73, 263, 309+C, 315+C, 489, 711, 750, 1041, 1438, 2706, 3106N, 3394, 4491, 4769, 5899+XC, 7028, 7142, 7697, 8701, 8860, 9242, 9540, 10398, 10400, 10873, 11719, 12705, 14308, 14417, 14766, 14783, 15043, 15301, 15326, 16223, 16234, 16316, 16362
K3546	Tib_9	Lazi, Shigatse	M9a1a1c1b1a	73, 263, 309+C, 315+C, 489, 711, 750, 1041, 1438, 2706, 3106N, 3394, 4491, 4769, 5899+XC, 7028, 7142, 7697, 8701, 8860, 9242, 9540, 10398, 10400, 10873, 11719, 12705, 14308, 14417, 14766, 14783, 15043, 15301, 15326, 16129, 16223, 16234, 16316, 16362
K3595	Tib_9	Lazi, Shigatse	M9a1b2	73, 150, 152, 153, 263, 309+CC, 315+C, 489, 750, 1041, 1438, 2706, 3106N, 3394, 4491, 4769, 7028, 8701, 8860, 9540, 10398, 10400, 10873, 10951A, 11719, 12362, 12705, 14308, 14766, 14783, 15043, 15301, 15326, 15671, 16158, 16223, 16234, 16362, 16519
K3755	Tib_9	Lazi, Shigatse	M9a1b1c	73, 150, 152, 153, 183, 263, 310, 489, 750, 1041, 1438, 2706, 3106N, 3394, 4491, 4769, 7028, 8701, 8860, 9540, 10398, 10400, 10454, 10873, 11719, 12362, 12705, 14308, 14766, 14783, 15043, 15301, 15326, 15671, 16158, 16223, 16234, 16362, 16519
K3604	Tib_9	Lazi, Shigatse	A14a	73, 151, 152, 200, 235, 263, 310, 523-524d, 663, 735, 750, 1438, 1736, 2706, 3106N, 4248, 4769, 4824, 6272, 7028, 8334, 8640, 8794, 8860, 11719, 12004, 12468, 12705, 12990, 14766, 15326, 16093, 16223, 16290, 16319, 16362, 16519
K2456	Tib_9	Lazi, Shigatse	C4b1	73, 146, 249d, 263, 310, 489, 750, 1438, 2232+A, 2706, 3106N, 3552A, 3816, 4715, 4769, 6026, 7028, 7196A, 8251, 8584, 8701, 8860, 9540, 9545, 10398, 10400, 10873, 11719, 11914, 11969, 12705, 13263, 14318, 14766, 14783, 15043, 15204, 15301, 15326, 15487T, 16129, 16223, 16298, 16327, 16519
K3603	Tib_9	Lazi, Shigatse	C4d2	73, 152, 249d, 263, 310, 489, 750, 1438, 2706, 3106N, 3552A, 4715, 4769, 6026, 7028, 7100, 7196A, 8584, 8701, 8860, 9540, 9545, 10398, 10400, 10873, 11719, 11914, 11969, 12705, 12780, 13263, 14318, 14488, 14766, 14783, 15043, 15204, 15236, 15301, 15326, 15487T, 16093, 16298, 16327, 16519
K3608	Tib_9	Lazi, Shigatse	D4s1a	73, 199, 263, 309+C, 315+C, 489, 524+AC, 750, 813, 1438, 2706, 3010, 3106N, 4769, 4883, 5178A, 7028, 8414, 8701, 8860, 9540, 10398, 10400, 10873, 11719, 12662, 12705, 13707, 14668, 14766, 14783, 15043, 15301, 15326, 15734, 16223, 16319, 16362, 16519
K3370	Tib_9	Lazi, Shigatse	D4s1a	73, 199, 263, 309+C, 315+C, 489, 524+AC, 750, 813, 1438, 2706, 3010, 3106N, 4769, 4883, 5178A, 7028, 8414, 8701, 8860, 9540, 10398, 10400, 10873, 11719, 12662, 12705, 14668, 14766, 14783, 15043, 15301, 15326, 15734, 16223, 16319, 16362, 16519
K3756	Tib_9	Lazi, Shigatse	G2a1f	73, 263, 309+CC, 315+C, 489, 709, 750, 1438, 2706, 3106N, 4769, 4833, 5108, 5601, 7028, 7600, 8701, 8860, 9377, 9540, 9575, 9659, 10398, 10400, 10873, 11719, 12705, 13563, 13884, 14200, 14569, 14766, 14783, 15043, 15301, 15326, 16051, 16114, 16223, 16227, 16278, 16291, 16362
K3601	Tib_9	Lazi, Shigatse	G3a1a2	16T, 73, 143, 150, 263, 309+C, 315+C, 489, 709, 750, 1438, 2706, 3106N, 4769, 4833, 5108, 7028, 8255, 8701, 8860, 8861, 9540, 10398, 10400, 10873, 11719, 11914, 12705, 14569, 14766, 14783, 15043, 15301, 15326, 15746, 16148, 16153, 16215, 16223, 16274
K2680	Tib_9	Lazi, Shigatse	G3a1a	16T, 73, 143, 150, 182, 263, 310, 489, 709, 750, 1438, 2706, 3106N, 4769, 4833, 5108, 7028, 8701, 8860, 8861, 8887, 9540, 10398, 10400, 10873, 11719, 11914, 12705, 14569, 14766, 14783, 15043, 15301, 15326, 15746, 16215, 16223, 16274
K2679	Tib_9	Lazi, Shigatse	G3b1	73, 195, 200, 215, 263, 310, 489, 709, 750, 1438, 2706, 3106N, 4769, 4833, 5108, 7028, 7897, 8701, 8860, 9540, 9599, 10398, 10400, 10873, 11719, 12705, 13477, 14094, 14569, 14605, 14766, 14783, 15043, 15301, 15326, 15927, 16223, 16274, 16362
K3835	Tib_9	Lazi, Shigatse	HV19	263, 309+C, 315+C, 750, 1438, 2706, 3106N, 4769, 4823, 5585, 7028, 8860, 15326, 16260, 16399
K2455	Tib_9	Lazi, Shigatse	M62b1a1	73, 143, 150, 203, 204, 263, 309+C, 315+C, 489, 750, 1438, 2706, 2735, 3106N, 3511, 4561, 4769, 7028, 7664, 7844, 8149, 8281-8289d, 8701, 8860, 9540, 10398, 10400, 10873, 10978, 11431, 11719, 12705, 13708, 14766, 14783, 15043, 15301, 15326, 15510, 15520, 15629, 15721, 15751, 16086, 16169, 16223, 16260, 16519
K3149	Tib_9	Lazi, Shigatse	M9a1a1c1b1a	73, 263, 309+CC, 315+C, 489, 711, 750, 1041, 1438, 2706, 3106N, 3394, 4136, 4491, 4769, 5899+XC, 7028, 7142, 7697, 8701, 8860, 9242, 9540, 10398, 10400, 10873, 11719, 12705, 14308, 14417, 14766, 14783, 15043, 15301, 15326, 16223, 16234, 16316, 16362
K2683	Tib_9	Lazi, Shigatse	M9a1a1c1b1a	73, 263, 310, 489, 711, 750, 1041, 1438, 2706, 3106N, 3394, 4491, 4769, 5899+XC, 7028, 7142, 7697, 8701, 8860, 9242, 9540, 10398, 10400, 10873, 11719, 12705, 14308, 14417, 14766, 14783, 15043, 15301, 15326, 16051, 16223, 16234, 16316, 16362
K2745	Tib_9	Lazi, Shigatse	Z3a1a	73, 152, 189, 204, 207, 249d, 263, 309+C, 315+C, 489, 573+XC, 750, 1438, 2706, 3106N, 4715, 4769, 6752, 7028, 7196A, 7702, 8584, 8701, 8860, 8931, 9090, 9540, 9713, 10208, 10398, 10400, 10873, 11075, 11719, 12705, 13359, 13620, 14766, 14783, 14841, 15043, 15301, 15487T, 15784, 15928, 16185, 16223, 16260

K2744	Tib_9	Lazi, Shigatse	C7a	73, 249d, 263, 310, 489, 750, 1438, 2706, 3106N, 3552A, 4715, 4769, 5821, 6338, 7028, 7153, 7196A, 7853, 8584, 8701, 8860, 9540, 9545, 10398, 10400, 10873, 11719, 11914, 12705, 13263, 14318, 14766, 14783, 15043, 15301, 15326, 15487T, 16223, 16298, 16327, 16362, 16519
K2747	Tib_9	Lazi, Shigatse	F1g	73, 249d, 263, 309+CC, 315+C, 523-524d, 750, 1438, 2389, 2706, 2857, 3106N, 3398, 3483, 3970, 4769, 6392, 6962, 7028, 8854, 8860, 10310, 10609, 11719, 12406, 12882, 13928C, 14766, 15326, 16183C, 16189, 16193+CC, 16304, 16519
k3757	Tib_9	Lazi, Shigatse	M11a	73, 125, 198, 215, 263, 309+C, 315+C, 318, 326, 489, 573+XC, 750, 1095, 1438, 2706, 3106N, 4769, 5582, 6531, 7028, 7642, 8108, 8701, 8860, 9540, 9554, 9950, 10398, 10400, 10873, 10914, 11272, 11719, 11969, 12705, 13074, 14163, 14340, 14766, 14783, 15043, 15301, 15326, 16188, 16223
K3831	Tib_9	Lazi, Shigatse	M9a1a1c1b1a	73, 263, 309+CC, 315+C, 489, 711, 750, 1041, 1438, 2706, 3106N, 3394, 4491, 4769, 5899+XC, 7028, 7142, 7697, 8701, 8860, 9242, 9540, 10398, 10400, 10873, 11719, 12705, 14308, 14417, 14766, 14783, 15043, 15301, 15326, 16223, 16234, 16316, 16362
K3839	Tib_9	Lazi, Shigatse	M9a1b1c	73, 150, 152, 153, 263, 310, 489, 750, 1041, 1438, 2706, 3106N, 3394, 4491, 4769, 7028, 8701, 8860, 9540, 10398, 10400, 10454, 10873, 11719, 12362, 12705, 14308, 14766, 14783, 15043, 15301, 15326, 15671, 16223, 16234, 16256, 16288, 16362, 16519
K3540	Tib_9	Lazi, Shigatse	D4j1a1d	73, 263, 310, 489, 750, 1189, 1438, 1462, 2706, 3010, 3106N, 4769, 4883, 5178A, 5262, 7028, 7581, 7783, 8414, 8701, 8860, 9540, 10398, 10400, 10873, 11696, 11719, 12130, 12358, 12648, 12705, 14668, 14766, 14783, 15043, 15295, 15301, 15326, 16086, 16223, 16274, 16362, 16519
K3840	Tib_9	Lazi, Shigatse	F1g2	73, 195, 249d, 263, 309+CC, 315+C, 523-524d, 750, 1438, 2389, 2706, 3106N, 3398, 3970, 4769, 6392, 6962, 7028, 8860, 10310, 10609, 11719, 12406, 12882, 13928C, 14766, 15326, 16183C, 16189, 16304, 16519
K3825	Tib_9	Lazi, Shigatse	M13a2	73, 152, 263, 309+C, 315+C, 489, 513, 750, 1438, 2706, 2786, 3106N, 3644, 4769, 5773, 6023, 6253, 6620, 6719, 7028, 8405, 8701, 8829, 8860, 9540, 10373, 10398, 10400, 10411, 10790, 10873, 11719, 12705, 13395, 14766, 14783, 15043, 15301, 15326, 15924, 15944d, 16145, 16168, 16188, 16223, 16257, 16311, 16519
K3299	Tib_9	Lazi, Shigatse	M62a1a	73, 146, 150, 263, 489, 750, 1438, 2706, 2735, 3106N, 3511, 4561, 4763, 4769, 7028, 7664, 8149, 8701, 8860, 9540, 9935, 10398, 10400, 10873, 11719, 12705, 13708, 14766, 14783, 15043, 15301, 15326, 15510, 15520, 15629, 15721, 16147, 16223, 16284, 16295, 16368, 16452, 16484, 16487, 16491, 16519
K2682	Tib_9	Lazi, Shigatse	A21a	73, 152, 235, 263, 310, 663, 750, 1438, 1736, 2706, 3106N, 4248, 4769, 4824, 6354Y, 7028, 8794, 8860, 11719, 12705, 14142, 14364, 14766, 15326, 16092, 16223, 16290, 16319, 16362
K3593	Tib_9	Lazi, Shigatse	D4j3b1	73, 94, 263, 309+C, 315+C, 338, 489, 750, 1438, 2706, 2880, 3010, 3106N, 4769, 4883, 5178A, 7028, 8414, 8701, 8860, 9540, 9630T, 10398, 10400, 10873, 11696, 11719, 12705, 14668, 14766, 14783, 15043, 15301, 15326, 16184, 16223, 16311, 16362
K3594	Tib_9	Lazi, Shigatse	M9a1b1e	73, 150, 152, 153, 263, 309+CC, 315+C, 489, 709, 750, 1041, 1438, 2706, 3106N, 3394, 4491, 4769, 7028, 8701, 8860, 9540, 10398, 10400, 10873, 11719, 12362, 12705, 14308, 14766, 14783, 15043, 15301, 15326, 15671, 16051, 16158, 16223, 16234, 16311, 16362, 16519
K3550	Tib_9	Lazi, Shigatse	G3b3	73, 152, 263, 315+C, 489, 524+AC, 709, 750, 1438, 2706, 2967, 3106N, 3394, 4113, 4769, 4833, 5108, 7028, 8701, 8860, 9540, 10398, 10400, 10873, 11719, 12705, 13477, 14569, 14605, 14766, 14783, 15043, 15301, 15326, 15927, 16209, 16223, 16274, 16362
K2699	Tib_9	Lazi, Shigatse	M9a1a1c1b1a13	73, 182, 263, 309+C, 315+C, 489, 711, 750, 1041, 1438, 2706, 3106N, 3394, 3866, 4491, 4769, 5474, 5899+XC, 7028, 7142, 7697, 8701, 8860, 9242, 9540, 10398, 10400, 10873, 11719, 12705, 14308, 14417, 14766, 14783, 15043, 15301, 15326, 16223, 16234, 16316, 16362
K2972	Tib_9	Lazi, Shigatse	B4d1'2'3	73, 263, 310, 750, 827, 1438, 2706, 3106N, 4769, 7028, 8116, 8281-8289d, 8860, 11719, 11914, 13942, 14766, 15326, 15535, 15930, 16183C, 16189, 16217, 16234, 16519
K3827	Tib_9	Lazi, Shigatse	A11a1a	73, 151, 152, 235, 263, 309+C, 315+C, 523-524d, 663, 750, 1005, 1438, 1736, 2706, 3106N, 4248, 4769, 4824, 5899+XC, 6755, 7028, 8794, 8843, 8860, 9650, 11719, 12705, 14766, 15326, 16093, 16223, 16290, 16293C, 16319
K3301	Tib_9	Lazi, Shigatse	A11a1a	73, 152, 235, 263, 309+C, 315+C, 523-524d, 663, 750, 1005, 1438, 1736, 2706, 3106N, 3918, 4248, 4769, 4824, 5768, 5899+XC, 6755, 7028, 8794, 8843, 8860, 9650, 11719, 12705, 14766, 15326, 16223, 16290, 16293C, 16319
K3829	Tib_9	Lazi, Shigatse	A6b	73, 146, 152, 235, 263, 310, 523-524d, 654, 663, 750, 1438, 1736, 2706, 3106N, 3687, 4248, 4769, 4824, 6707, 7028, 8531, 8794, 8860, 10097, 10993, 11719, 12705, 13287, 14766, 15326, 15670, 16223, 16290, 16319, 16362
K2681	Tib_9	Lazi, Shigatse	M10a1b	73, 263, 310, 489, 573+XC, 709, 750, 1438, 2706, 3106N, 3172+C, 4140, 4769, 7028, 7250, 8701, 8793, 8856, 8860, 9540, 10398, 10400, 10646, 10873, 11719, 12549, 12705, 13152, 14502, 14766, 14783, 15040, 15043, 15071, 15218, 15301, 15326, 15913, 15927, 16066, 16150, 16223, 16311
K3843	Tib_9	Lazi, Shigatse	M9a1a1c1b1a2	73, 146, 263, 309+CC, 315+C, 489, 711, 750, 1041, 1438, 2706, 3106N, 3394, 4491, 4769, 5899+XC, 6446, 7028, 7142, 7697, 8701, 8860, 9242, 9540, 10398, 10400, 10873, 11719, 12705, 14308, 14417, 14766, 14783, 15043, 15301, 15326, 16223, 16234, 16316, 16362

K3298	Tib_9	Lazi, Shigatse	H14	263, 309+CC, 315+C, 750, 1438, 3106N, 3197, 4769, 6570T, 7645, 7990, 8860, 10217, 10685, 15326
K3834	Tib_9	Lazi, Shigatse	M9a1a1c1b1a	73, 263, 309+CC, 315+C, 489, 711, 750, 1041, 1438, 2706, 3106N, 3394, 4491, 4769, 5899+XC, 7028, 7142, 7697, 8701, 8860, 9242, 9540, 10398, 10400, 10873, 11719, 12705, 14308, 14417, 14766, 14783, 15043, 15301, 15326, 16223, 16234, 16316, 16362
K3508	Tib_10	Zhongba, Shigatse	M9a1b1c	73, 150, 152, 153, 263, 309+CC, 315+C, 489, 750, 1041, 1438, 2706, 3106N, 3394, 4491, 4769, 7028, 8701, 8860, 9540, 10398, 10400, 10454, 10873, 11719, 12362, 12705, 14308, 14766, 14783, 15043, 15301, 15326, 15671, 16158, 16234, 16362, 16519
K3498	Tib_10	Zhongba, Shigatse	G2a1f	73, 263, 309+C, 315+C, 489, 709, 750, 1438, 2706, 3106N, 4769, 4823, 4833, 5108, 5601, 7028, 7600, 8701, 8860, 9377, 9540, 9575, 9659, 10398, 10400, 10873, 11719, 12705, 13563, 13884, 14200, 14569, 14766, 14783, 15043, 15301, 15326, 16051, 16114, 16223, 16227, 16278, 16362
K3497	Tib_10	Zhongba, Shigatse	M62b1a1b1	73, 143, 150, 203, 204, 263, 309+C, 315+C, 489, 750, 1438, 2706, 2735, 3106N, 3511, 4561, 4769, 7028, 7471+C, 7664, 7844, 8149, 8281-8289d, 8701, 8860, 9540, 10398, 10400, 10873, 10978, 11431, 11719, 12705, 13708, 14766, 14783, 15043, 15301, 15326, 15510, 15520, 15629, 15721, 16086, 16169, 16223, 16260, 16519
K3494	Tib_10	Zhongba, Shigatse	M9a1a1c1b1a	73, 263, 309+C, 315+C, 489, 711, 750, 1041, 1438, 2706, 3106N, 3394, 4491, 4769, 5899+XC, 7028, 7142, 7697, 8701, 8860, 9242, 9540, 10398, 10400, 10873, 11719, 12705, 14308, 14417, 14766, 14783, 15043, 15301, 15326, 16223, 16234, 16316, 16362
K3488	Tib_10	Zhongba, Shigatse	C4d2	73, 152, 249d, 310, 489, 750, 1438, 2706, 3106N, 3552A, 4715, 4769, 5471, 6026, 7028, 7100, 7196A, 8584, 8701, 8860, 9540, 9545, 10398, 10400, 10873, 11719, 11914, 11969, 12705, 12780, 13263, 14318, 14766, 14783, 15043, 15204, 15236, 15301, 15326, 15487T, 16093, 16298, 16327, 16519
K3483	Tib_10	Zhongba, Shigatse	H15a1	199, 263, 309+C, 315+C, 750, 951, 1438, 2248, 3106N, 4769, 6173, 6253, 8860, 11410, 13095, 14953, 15326, 16354
K3478	Tib_10	Zhongba, Shigatse	M13a1b	73, 152, 263, 310, 489, 750, 1438, 1664, 2706, 3106N, 3644, 3918, 4769, 5773, 6023, 6253, 6620, 7028, 8701, 8860, 9064, 9540, 10192, 10398, 10400, 10411, 10790, 10873, 11719, 12705, 13135, 14766, 14783, 15043, 15301, 15326, 15586, 15924, 16145, 16148, 16188, 16189, 16223, 16381
K3476	Tib_10	Zhongba, Shigatse	C4a1a1a	64, 73, 249d, 263, 310, 489, 523-524d, 750, 1438, 1715, 2232+AA, 2706, 3106N, 3552A, 3576, 4715, 4769, 4884, 4958, 6026, 7028, 7196A, 7999, 8508, 8584, 8701, 8860, 9540, 9545, 10398, 10400, 10873, 11719, 11914, 11969, 12672, 12705, 13263, 14318, 14560, 14766, 14783, 15043, 15204, 15301, 15326, 15487T, 15968, 16093, 16129, 16223, 16298, 16327, 16519
K3477	Tib_10	Zhongba, Shigatse	D4j3b1	73, 94, 263, 309+C, 315+C, 338, 489, 750, 1438, 2706, 2880, 3010, 3106N, 4769, 4883, 5178A, 7028, 8414, 8701, 8860, 9540, 10398, 10400, 10873, 11696, 11719, 12705, 14668, 14766, 14783, 15043, 15301, 15326, 16184, 16223, 16311, 16362
K3806	Tib_10	Zhongba, Shigatse	D5a2a	73, 150, 263, 310, 489, 523-524d, 750, 752, 1107, 2706, 3106N, 4619, 4769, 4883, 5178A, 5301, 7028, 7270, 8701, 8860, 9180, 9540, 10101, 10397, 10398, 10400, 10873, 11719, 11944, 12026, 12705, 14766, 14783, 15043, 15226, 15301, 15326, 15346, 16164, 16172, 16182C, 16183C, 16189, 16223, 16266, 16362
K3512	Tib_10	Zhongba, Shigatse	M9a1b1e	73, 150, 152, 153, 263, 309+C, 315+C, 489, 709, 750, 1041, 1438, 2706, 3106N, 3394, 4491, 4769, 7028, 8701, 8860, 9540, 10398, 10400, 10873, 11719, 12362, 12705, 14308, 14766, 14783, 15043, 15301, 15326, 15671, 16093, 16158, 16223, 16234, 16362, 16519
K3305	Tib_10	Zhongba, Shigatse	G2b2a	73, 183, 263, 309+C, 315+C, 489, 709, 750, 1438, 1692, 2706, 3106N, 4216, 4680A, 4769, 4833, 5108, 5601, 6116, 6932, 7028, 8701, 8860, 8877, 9540, 10398, 10400, 10873, 11719, 12705, 13563, 14180, 14569, 14766, 14783, 15043, 15301, 15326, 16223, 16294, 16362
K3507	Tib_10	Zhongba, Shigatse	A11a1a3	73, 152, 235, 263, 309+C, 315+C, 523-524d, 663, 750, 1005, 1438, 1736, 2706, 3106N, 4248, 4769, 4824, 5899+XC, 6755, 7028, 8794, 8843, 8860, 9650, 10040, 11719, 12705, 13708, 14766, 15326, 16093, 16223, 16290, 16293C, 16319, 16519
K3484	Tib_10	Zhongba, Shigatse	C4a2b	73, 249d, 263, 310, 489, 750, 1438, 2232+A, 2706, 2887, 3106N, 3552A, 4696, 4715, 4769, 6026, 7028, 7196A, 8584, 8701, 8860, 9540, 9545, 10398, 10400, 10873, 11719, 11914, 11969, 12624, 12672, 12705, 13263, 14318, 14766, 14769, 14783, 15043, 15204, 15301, 15326, 15487T, 16223, 16298, 16327, 16357, 16519
K3804	Tib_10	Zhongba, Shigatse	M9a1a1c1b1a4	73, 263, 309+CC, 315+C, 489, 711, 750, 1041, 1438, 2706, 3106N, 3394, 4491, 4769, 5899+XC, 7028, 7142, 7697, 8701, 8860, 9242, 9540, 10398, 10400, 10873, 11719, 12705, 14308, 14417, 14766, 14783, 15043, 15301, 15326, 15470, 16223, 16234, 16316, 16362
K3803	Tib_10	Zhongba, Shigatse	M9a1a1c1b1a4	73, 263, 309+CC, 315+C, 489, 711, 750, 1041, 1438, 2706, 3106N, 3394, 4491, 4769, 5899+XC, 7028, 7142, 7697, 8701, 8860, 9242, 9540, 10398, 10400, 10873, 11719, 12705, 14308, 14417, 14766, 14783, 15043, 15301, 15326, 15470, 16223, 16234, 16316, 16362
K3511	Tib_10	Zhongba, Shigatse	A	73, 152, 182, 235, 263, 309+CC, 315+C, 663, 750, 1438, 1736, 2706, 3106N, 4248, 4769, 4824, 7028, 8794, 8860, 11719, 12705, 13488, 14766, 15326, 16223, 16248, 16290, 16319, 16362
K3481	Tib_10	Zhongba, Shigatse	A14a	73, 151, 152, 200, 235, 263, 310, 523-524d, 663, 735, 750, 1438, 1736, 2706, 3106N, 4248, 4769, 4824, 6272, 7028, 8334, 8640, 8794, 8860, 11719, 12004, 12468, 12705, 12990, 14766, 15326, 16093, 16223, 16290, 16319, 16362, 16519

K3805	Tib_10	Zhongba, Shigatse	A21b1	73, 152, 235, 263, 309+C, 315+C, 663, 750, 1438, 1736, 2706, 3106N, 4248, 4769, 4824, 7028, 8485, 8794, 8860, 11719, 12705, 13934, 14364, 14766, 15326, 16092, 16223, 16290, 16319, 16362
K3475	Tib_10	Zhongba, Shigatse	B4h1	73, 195, 263, 309+CC, 315+C, 523-524d, 750, 1438, 2706, 3106N, 3398, 4703, 4769, 5093, 7028, 7080, 8281-8289d, 8790, 8812, 8860, 11485, 11719, 13269, 13710, 14766, 15326, 16129, 16181-16183d, 16189, 16261
K3503	Tib_10	Zhongba, Shigatse	D4q	73, 200, 263, 309+C, 315+C, 489, 750, 1438, 2625, 2706, 3010, 3106N, 4769, 4883, 5178A, 7028, 8414, 8701, 8860, 9540, 10398, 10400, 10873, 11719, 12705, 14668, 14766, 14783, 15043, 15301, 15326, 16223, 16256, 16311, 16362, 16519
K3304	Tib_10	Zhongba, Shigatse	F1c1a1a1	73, 152, 249d, 263, 310, 523-524d, 573+XC, 709, 750, 1438, 2706, 3106N, 3970, 4769, 6392, 6599, 6962, 7028, 8860, 9053, 9647, 10310, 10454, 10609, 11719, 12406, 12441, 12882, 13759, 13928C, 14766, 15326, 16051, 16111, 16129, 16304, 16519
K3510	Tib_10	Zhongba, Shigatse	F1c1a1a2	73, 143, 152, 249d, 263, 310, 523-524d, 573+XC, 709, 750, 1438, 2706, 3106N, 3970, 4769, 6392, 6599, 6962, 7028, 7055, 7915, 8412, 8860, 9053, 9647, 10181, 10310, 10454, 10609, 10739, 11719, 12406, 12882, 13759, 13928C, 14766, 15326, 16111, 16129, 16266, 16304, 16519
K3509	Tib_10	Zhongba, Shigatse	G2a1	73, 263, 309+C, 315+C, 489, 709, 750, 1313C, 1438, 2706, 3106N, 3290, 4102, 4277, 4769, 4833, 5108, 5601, 7028, 7600, 8701, 8860, 9377, 9540, 9575, 10398, 10400, 10873, 11719, 12705, 13563, 13926, 14155, 14200, 14212, 14569, 14766, 14783, 14839, 15043, 15301, 15326, 16223, 16227, 16278, 16304, 16362
K3489	Tib_10	Zhongba, Shigatse	M62a1	73, 146, 150, 263, 489, 750, 1438, 2706, 2735, 3106N, 3511, 4561, 4763, 4769, 7028, 7664, 8149, 8701, 8860, 9540, 9935, 10398, 10400, 10873, 11719, 12705, 13708, 14766, 14783, 15043, 15301, 15326, 15510, 15520, 15629, 15721, 16147, 16189, 16193+CC, 16223, 16295, 16487, 16519
K3801	Tib_10	Zhongba, Shigatse	M9a1a1c1b1a13	73, 263, 309+C, 315+C, 489, 711, 750, 1041, 1438, 2706, 3106N, 3394, 3866, 4491, 4769, 5899+XC, 7028, 7142, 7697, 8701, 8860, 9083, 9242, 9540, 10343, 10398, 10400, 10873, 11719, 12705, 14308, 14417, 14766, 14783, 15043, 15301, 15326, 16223, 16234, 16316, 16362
K3492	Tib_10	Zhongba, Shigatse	M9a1a1c1b1a	73, 263, 309+C, 315+C, 489, 711, 750, 1041, 1438, 2706, 3106N, 3394, 4491, 4769, 5378, 5899+XC, 7028, 7142, 7697, 8701, 8860, 9242, 9540, 10398, 10400, 10873, 11719, 12705, 14308, 14417, 14766, 14783, 15043, 15301, 15326, 16223, 16234, 16316, 16362, 16519
K3474	Tib_10	Zhongba, Shigatse	M9a1a1c1b1a8	73, 263, 309+C, 315+C, 489, 711, 750, 1041, 1438, 2706, 3106N, 3394, 4491, 4769, 7028, 7142, 7697, 8628, 8701, 8860, 9242, 9540, 10398, 10400, 10873, 11719, 12705, 14308, 14417, 14766, 14783, 15043, 15301, 15326, 16223, 16234, 16316, 16362
K3101	Tib_10	Zhongba, Shigatse	M9a1a1c1b1a	73, 263, 309+C, 315+C, 489, 711, 750, 1041, 1438, 2706, 3106N, 3394, 4491, 4769, 5899+XC, 7028, 7142, 7697, 8701, 8860, 9242, 9540, 10398, 10400, 10873, 11719, 12705, 14308, 14417, 14766, 14783, 15043, 15301, 15326, 16223, 16234, 16316, 16362
K3096	Tib_10	Zhongba, Shigatse	M9a1b1e	73, 150, 152, 153, 263, 309+C, 315+C, 489, 709, 750, 1041, 1438, 2706, 3106N, 3394, 4491, 4769, 7028, 8701, 8860, 9540, 10398, 10400, 10873, 11719, 12362, 12705, 14308, 14766, 14783, 15043, 15301, 15326, 15671, 16093, 16158, 16223, 16234, 16362, 16519
K3812	Tib_10	Zhongba, Shigatse	A11a1a	73, 151, 152, 235, 263, 309+C, 315+C, 523-524d, 663, 750, 1005, 1438, 1736, 2706, 3106N, 4248, 4769, 4824, 5899+XC, 6755, 7028, 8794, 8843, 8860, 9650, 11719, 12705, 14766, 15326, 16093, 16223, 16290, 16293C, 16319
K3814	Tib_10	Zhongba, Shigatse	C4a2b	73, 249d, 263, 310, 489, 750, 1438, 1547+T, 2232+A, 2706, 2887, 2961, 3106N, 3552A, 4696, 4715, 4769, 6026, 7028, 7196A, 8584, 8701, 8860, 9540, 9545, 10398, 10400, 10873, 11719, 11914, 11969, 12624, 12672, 12705, 13263, 14318, 14766, 14769, 14783, 15043, 15204, 15301, 15326, 15487T, 16223, 16298, 16327, 16357, 16519
K3490	Tib_10	Zhongba, Shigatse	D4j3b	73, 263, 309+C, 315+C, 338, 489, 750, 1438, 2706, 3010, 3106N, 4769, 4883, 5178A, 7028, 8414, 8701, 8860, 9540, 10398, 10400, 10873, 11696, 11719, 12705, 14668, 14766, 14783, 14851, 15043, 15301, 15326, 16184, 16223, 16311, 16362, 16519
K3802	Tib_10	Zhongba, Shigatse	F1c1a1a3	73, 152, 249d, 263, 309+C, 315+C, 523-524d, 573+XC, 709, 750, 1438, 2706, 3106N, 3970, 4769, 6392, 6599, 6962, 7028, 8860, 9053, 9389, 9647, 10310, 10454, 10609, 11719, 12406, 12882, 13759, 13928C, 14766, 15326, 16111, 16129, 16266, 16304, 16362, 16519
K3493	Tib_10	Zhongba, Shigatse	M13a1b	73, 151, 152, 263, 310, 489, 750, 1438, 2706, 3106N, 3644, 4769, 5773, 6023, 6253, 6620, 7028, 8701, 8860, 9540, 10398, 10400, 10411, 10790, 10873, 11719, 12654, 12705, 13135, 14312, 14766, 14783, 15043, 15077, 15301, 15326, 15924, 16145, 16148, 16188, 16189, 16223, 16381
K3496	Tib_10	Zhongba, Shigatse	M9a1a1c1b1a1	73, 263, 309+C, 315+C, 489, 711, 750, 1041, 1438, 2706, 3106N, 3394, 4491, 4769, 5899+XC, 7028, 7142, 7697, 8567, 8701, 8860, 9242, 9540, 10398, 10400, 10873, 11719, 12705, 14308, 14417, 14766, 14783, 15043, 15301, 15326, 15924, 16086, 16223, 16234, 16316, 16362
K3808	Tib_10	Zhongba, Shigatse	M62b2a	73, 150, 187T, 204, 263, 309+C, 315+C, 489, 750, 1438, 2706, 2735, 3106N, 3511, 3693, 4561, 4769, 6305, 7028, 7364, 7664, 8149, 8701, 8860, 8932, 9540, 10398, 10400, 10873, 11719, 12669, 12705, 13434, 13708, 14766, 14783, 15016A, 15043, 15301, 15326, 15510, 15520, 15629, 15721, 15799, 16223, 16260, 16295, 16519

K3100	Tib_10	Zhongba, Shigatse	M9a1a1c1b1a	73, 263, 310, 489, 711, 750, 1041, 1438, 2416, 2706, 3106N, 3394, 4491, 4769, 5899+XC, 7028, 7142, 7697, 8701, 8860, 9242, 9540, 10398, 10400, 10873, 11719, 12705, 14110, 14308, 14417, 14766, 14783, 15043, 15301, 15326, 15769, 16176, 16223, 16234, 16311, 16316, 16362, 16519
K3097	Tib_10	Zhongba, Shigatse	M9a1a1c1b1a12	73, 263, 309+C, 315+C, 489, 711, 750, 1041, 1438, 2706, 3106N, 3394, 3720, 4491, 4769, 5899+XC, 7028, 7142, 7697, 8701, 8860, 9242, 9540, 10398, 10400, 10873, 11719, 12705, 14308, 14417, 14766, 14783, 15043, 15301, 15326, 16114G, 16223, 16234, 16316, 16362
K3307	Tib_10	Zhongba, Shigatse	M9a1b1c	73, 150, 152, 263, 310, 489, 750, 1041, 1438, 2706, 3106N, 3394, 4491, 4769, 7028, 8701, 8860, 9540, 10398, 10400, 10454, 10873, 11719, 12362, 12705, 14308, 14766, 14783, 15043, 15301, 15326, 15671, 16158, 16223, 16234, 16362, 16519
K3807	Tib_10	Zhongba, Shigatse	C4a2b2	73, 194, 249d, 263, 489, 750, 1438, 1677, 2232+A, 2706, 2887, 3106N, 3552A, 4696, 4715, 4769, 6026, 6221, 7028, 7196A, 8584, 8701, 8860, 9540, 9545, 10304, 10398, 10400, 10873, 11719, 11914, 11969, 12624, 12672, 12705, 13263, 14318, 14766, 14783, 15043, 15204, 15301, 15326, 15487T, 16223, 16298, 16311, 16327, 16357, 16519
K3499	Tib_10	Zhongba, Shigatse	D4j1	73, 185, 263, 309+C, 315+C, 489, 750, 1438, 2069, 2706, 3010, 3106N, 4263, 4769, 4883, 5178A, 5262, , 7028, 7058, 8281-8289d, 8414, 8701, 8860, 8950, 9540, 10398, 10400, 10873, 11696, 11719, 12705, 14305, 14668, 14766, 14783, 15043, 15301, 15326, 16223, 16260, 16362
K3506	Tib_10	Zhongba, Shigatse	M9a1a1c1b1a	73, 263, 310, 489, 711, 750, 1041, 1438, 2706, 3106N, 3394, 4069TC, 4491, 4769, 5899+XC, 7028, 7142, 7697, 8701, 8860, 9242, 9540, 9833, 10398, 10400, 10654, 10873, 11447, 11719, 12705, 14308, 14417, 14766, 14783, 15043, 15301, 15326, 16223, 16234, 16316, 16362
K3502	Tib_10	Zhongba, Shigatse	B6a1a	73, 263, 309+C, 315+C, 356+C, 750, 1438, 1719, 2706, 3106N, 4093, 4769, 5894C, 7028, 8281-8289d, 8860, 9452, 11719, 11914, 12950, 13928C, 14305, 14766, 15326, 16051, 16183C, 16189, 16193+CC, 16261, 16519, 16527
K3811	Tib_10	Zhongba, Shigatse	D4b2b8	73, 263, 310, 390, 489, 523-524d, 750, 1382C, 1438, 2706, 3010, 3106N, 3398, 4769, 4883, 5178A, 7028, 8020, 8414, 8701, 8860, 8964, 9296, 9540, 9824A, 10398, 10400, 10873, 11719, 12705, 14668, 14755, 14766, 14783, 15043, 15301, 15326, 15613, 16223, 16362, 16519
K3810	Tib_10	Zhongba, Shigatse	G2a1i	73, 263, 310, 489, 709, 750, 1438, 2706, 3106N, 4769, 4833, 5108, 5601, 7028, 7600, 8701, 8860, 9377, 9540, 9575, 10398, 10400, 10873, 11719, 12705, 13517T, 13563, 14200, 14569, 14766, 14783, 15043, 15301, 15326, 16129, 16223, 16231G, 16278, 16362
K3501	Tib_10	Zhongba, Shigatse	D4j1a1e	73, 195, 263, 310, 489, 750, 1438, 2706, 3010, 3106N, 4769, 4883, 5178A, 5262, 7028, 7581, 7783, 8414, 8701, 8860, 9540, 10398, 10400, 10873, 11696, 11719, 12130, 12358, 12705, 14668, 14766, 14783, 15043, 15295, 15301, 15326, 16086, 16223, 16362, 16399, 16519
K3486	Tib_10	Zhongba, Shigatse	M62b1	73, 150, 203, 204, 263, 309+C, 315+C, 489, 750, 1438, 2706, 2735, 3106N, 3511, 4561, 4769, 7028, 7664, 8084, 8149, 8701, 8860, 9540, 10398, 10400, 10873, 11719, 12705, 13708, 14058, 14766, 14783, 15043, 15301, 15326, 15510, 15520, 15629, 15721, 16223, 16260, 16274, 16295, 16318, 16320, 16519
K38217	Tib_11	Shannan	D4j1a1f	73, 263, 310, 489, 750, 1438, 2706, 3010, 3106N, 4769, 4883, 5178A, 5262, 7028, 7581, 7783, 8414, 8701, 8860, 9053, 9540, 10398, 10400, 10873, 11696, 11719, 12130, 12358, 12705, 14668, 14766, 14783, 15043, 15184, 15295, 15301, 15326, 16086, 16223, 16311, 16362
K38030	Tib_11	Shannan	M9a1b1c	73, 150, 152, 153, 263, 310, 489, 750, 1041, 1438, 2706, 3106N, 3394, 4491, 4769, 7028, 8701, 8860, 9540, 10398, 10400, 10454, 10517, 10873, 11719, 12362, 12705, 14308, 14766, 14783, 15043, 15132, 15301, 15326, 15671, 16158, 16223, 16234, 16265C, 16362, 16519
K38085	Tib_11	Shannan	M9a1a1c1b1a11	73, 263, 309+C, 315+C, 489, 711, 750, 1041, 1438, 2706, 3106N, 3311, 3394, 4491, 4769, 5492, 5899+XC, 7028, 7142, 7697, 8701, 8860, 9242, 9540, 10398, 10400, 10873, 11719, 12705, 14308, 14417, 14766, 14783, 15043, 15301, 15326, 16223, 16234, 16316, 16362
K38214	Tib_11	Shannan	C4a1a1a2	73, 249d, 263, 310, 489, 750, 1438, 1715, 2232+AA, 2706, 3106N, 3552A, 3576, 4715, 4769, 4884, 4958, 5918, 6026, 7028, 7196A, 7999, 8508, 8584, 8701, 8860, 9540, 9545, 10398, 10400, 10873, 11719, 11914, 11969, 12672, 12705, 13263, 14318, 14766, 14783, 15043, 15204, 15219, 15301, 15326, 15487T, 15968, 16093, 16129, 16223, 16298, 16327, 16519
K38626	Tib_11	Shannan	M9a1a1c1b1a	73, 195, 263, 309+C, 315+C, 489, 711, 750, 1041, 1438, 2706, 3106N, 3394, 4491, 4769, 5894C, 7028, 7142, 7697, 8701, 8860, 9242, 9540, 10398, 10400, 10873, 11719, 12705, 14308, 14417, 14766, 14783, 15043, 15301, 15326, 16223, 16234, 16316, 16362
K38722	Tib_11	Shannan	A11a1a3	73, 152, 235, 263, 309+C, 315+C, 523-524d, 663, 750, 1005, 1438, 1736, 2706, 3106N, 4248, 4295, 4769, 4824, 5899+XC, 6755, 7028, 8794, 8843, 8860, 9650, 11719, 12705, 13708, 14766, 15326, 16093, 16223, 16290, 16293C, 16319, 16357, 16519
K38615	Tib_11	Shannan	A11a1a4	73, 152, 235, 263, 310, 523-524d, 663, 750, 1005, 1438, 1736, 2083, 2523, 2557, 2625, 2706, 3106N, 4248, 4769, 4824, 5899+XC, 6755, 7028, 8794, 8843, 8860, 9055, 9650, 11719, 12705, 13926, 14766, 15326, 16093, 16223, 16290, 16293C, 16319, 16519
K38597	Tib_11	Shannan	A11a1a	73, 146, 152, 235, 263, 309+CC, 315+C, 523-524d, 663, 750, 1005, 1438, 1736, 2706, 3106N, 4248, 4769, 4824, 5581, 5899+XC, 6755, 7028, 8794, 8843, 8860, 9650, 11719, 12705, 14766, 15326, 16093, 16223, 16290, 16293C, 16319, 16519

K38355	Tib_11	Shannan	B4a4	73, 193, 263, 309+C, 315+C, 523-524d, 709, 750, 825A, 1106, 1185, 1211, 1438, 2523, 2557, 2625, 2706, 3106N, 3209T, 3338, 4769, 5438, 5465, 6929, 7028, 7853, 8281-8289d, 8860, 9123, 9233, 11719, 14751, 14766, 15326, 16182C, 16183C, 16189, 16217, 16261, 16299, 16519
K38704	Tib_11	Shannan	C4a2b2	73, 249d, 263, 489, 750, 1438, 1677, 2232+A, 2706, 2887, 3106N, 3552A, 4696, 4715, 4769, 6026, 7028, 7196A, 8584, 8701, 8860, 9540, 9545, 10304, 10398, 10400, 10873, 11719, 11914, 11969, 12624, 12672, 12705, 13263, 14318, 14766, 14783, 15043, 15148, 15204, 15301, 15326, 15487T, 16223, 16298, 16311, 16327, 16357, 16519
K38489	Tib_11	Shannan	D4b2b10	41, 73, 194, 263, 310, 489, 523-524d, 750, 1382C, 1438, 2706, 3010, 3106N, 4769, 4883, 5178A, 7028, 8020, 8414, 8701, 8860, 8964, 9296, 9540, 9824A, 10398, 10400, 10873, 11719, 12705, 14668, 14766, 14783, 15043, 15301, 15326, 16223, 16362, 16519
K38808	Tib_11	Shannan	M9a1a1c1b1a	73, 263, 309+C, 315+C, 489, 711, 750, 1041, 1438, 2706, 3106N, 3394, 4491, 4769, 5899+XC, 7028, 7142, 7697, 8251, 8701, 8860, 9242, 9540, 10398, 10400, 10873, 11719, 12705, 13434, 14308, 14417, 14766, 14783, 15043, 15202, 15301, 15326, 16223, 16234, 16316, 16362
K38132	Tib_11	Shannan	M9a1a1c1b1a	73, 263, 309+CC, 315+C, 489, 711, 750, 1041, 1438, 2706, 3106N, 3394, 4491, 4769, 5899+XC, 7028, 7142, 7697, 8701, 8860, 9242, 9540, 10398, 10400, 10873, 11719, 12705, 14308, 14417, 14766, 14783, 15043, 15301, 15326, 16223, 16234, 16316, 16362
K38129	Tib_11	Shannan	M9a1a1c1b1a	73, 263, 310, 489, 711, 750, 1041, 1438, 2706, 3106N, 3394, 4491, 4769, 5899+XC, 6710, 7028, 7142, 7697, 8701, 8860, 9242, 9540, 10398, 10400, 10873, 11719, 12705, 14308, 14417, 14766, 14783, 15043, 15301, 15326, 16223, 16234, 16316, 16362
K38015	Tib_11	Shannan	M9a1a1c1b1a2	73, 146, 263, 310, 489, 711, 750, 1041, 1438, 2706, 3106N, 3394, 4491, 4769, 5899+XC, 7028, 7142, 7697, 8701, 8860, 9242, 9540, 10398, 10400, 10873, 11719, 12705, 14308, 14417, 14766, 14783, 15043, 15301, 15326, 16093, 16223, 16234, 16316, 16362
K38122	Tib_11	Shannan	M9a1a1c1b1a2	73, 146, 263, 297, 309+C, 315+C, 489, 711, 750, 1041, 1438, 2706, 3106N, 3367, 3394, 3438, 4491, 4769, 5899+XC, 6446, 7028, 7142, 7697, 8701, 8860, 9242, 9540, 10398, 10400, 10873, 11719, 12528, 12705, 12727, 14308, 14417, 14766, 14783, 15043, 15301, 15326, 16051, 16129, 16223, 16234, 16316, 16362
K38530	Tib_11	Shannan	M9a1b	73, 150, 152, 153, 263, 309+C, 315+C, 489, 750, 1041, 1438, 2706, 3106N, 3394, 4491, 4769, 7028, 8701, 8853, 8860, 9540, 10398, 10400, 10873, 11719, 12362, 12705, 13470, 14308, 14766, 14783, 15043, 15301, 15326, 15671, 16223, 16234, 16362, 16519
K38422	Tib_11	Shannan	A11b	73, 146, 152, 235, 263, 309+CC, 315+C, 523-524d, 663, 750, 1438, 1736, 2706, 3106N, 3290, 4117, 4248, 4769, 4824, 7028, 8794, 8860, 9489, 9650, 11719, 12705, 14766, 15326, 15787, 16223, 16234, 16290, 16293C, 16319, 16519, 16527
K38661	Tib_11	Shannan	G3b	73, 263, 310, 489, 709, 750, 1438, 2706, 3106N, 4769, 4833, 5108, 7028, 8584, 8701, 8860, 9007, 9540, 10398, 10400, 10873, 11009, 11719, 12705, 13477, 14221, 14569, 14605, 14766, 14783, 15043, 15301, 15326, 15927, 16223, 16274, 16362
K38235	Tib_11	Shannan	B4d1'2'3	73, 263, 309+CC, 315+C, 556T, 573+XC, 750, 827, 1438, 2706, 3106N, 3757, 4769, 7028, 8281-8289d, 8512, 8860, 11719, 11914, 12142, 13942, 14766, 15326, 15535, 15930, 16183C, 16189, 16217, 16234, 16519
K38382	Tib_11	Shannan	D4j1a1f	73, 263, 310, 489, 524+ACAC, 750, 1438, 2706, 3010, 3106N, 4769, 4883, 5147, 5178A, 5262, 7028, 7581, 7783, 8414, 8701, 8860, 9053, 9540, 9650, 10398, 10400, 10873, 11696, 11719, 12130, 12358, 12705, 14668, 14766, 14783, 15043, 15295, 15301, 15326, 16086, 16192, 16223, 16362
K38470	Tib_11	Shannan	F1d1a2	73, 146, 249d, 263, 309+CC, 315+C, 523-524d, 750, 1438, 1734, 2706, 3106N, 3970, 4769, 5628, 6392, 6962, 7028, 7738, 8860, 10310, 10609, 11719, 12406, 12882, 13135, 13928C, 14766, 15326, 15402, 16145, 16304, 16519
K38315	Tib_11	Shannan	G2a1i	73, 263, 310, 489, 709, 750, 1438, 2706, 3106N, 4769, 4833, 5108, 5601, 6896, 7028, 7600, 8701, 8860, 9377, 9540, 9575, 9948, 10398, 10400, 10873, 11719, 12705, 13563, 14200, 14569, 14766, 14783, 15043, 15301, 15326, 16129, 16223, 16278, 16362
K38043	Tib_11	Shannan	M9a1a1c1b1a	73, 263, 279, 309+CC, 315+C, 489, 711, 750, 1041, 1438, 2706, 3106N, 3394, 4491, 4769, 5899+XC, 7028, 7142, 7697, 8701, 8854, 8860, 9242, 9540, 10398, 10400, 10873, 11447, 11506, 11719, 12705, 14308, 14417, 14766, 14783, 15043, 15301, 15326, 16223, 16234, 16316, 16362
K38746	Tib_11	Shannan	M9a1b1d	73, 150, 152, 153, 263, 310, 489, 750, 1041, 1438, 2706, 3106N, 3394, 4491, 4769, 7028, 7754, 8701, 8860, 9540, 10398, 10400, 10873, 11719, 12362, 12705, 14308, 14766, 14783, 15043, 15119, 15301, 15326, 15671, 16158, 16223, 16234, 16362, 16519
K38345	Tib_11	Shannan	F1c1a1a	73, 152, 249d, 263, 309+CC, 315+C, 523-524d, 573+XC, 709, 750, 1438, , 2706, 3106N, 3970, 4769, 6392, 6599, 6638, 6962, 7028, 8860, 9053, 9647, 10310, 10454, 10609, 11719, 11928, 12406, 12882, 13020, 13759, 13928C, 14766, 15326, 16111, 16129, 16266, 16304, 16519
K38744	Tib_11	Shannan	A15c1	73, 152, 235, 309+C, 315+C, 523-524d, 663, 750, 825A, 1185, 1211, 1249, 1263T, 1303, 1320, 1438, 1736, 2523, 2557, 2625, 2706, 3106N, 4248, 4769, 4824, 7028, 7609, 8459, 8794, 8860, 9052, 11719, 12705, 13111, 13899, 14067, 14766, 15262, 15326, 15924, 16092, 16223, 16290, 16319, 16327, 16362
K38765	Tib_11	Shannan	F1g	73, 249d, 263, 309+CC, 315+C, 523-524d, 750, 825A, 1185, 1211, 1438, 2389, 2523, 2557, 2625, 2706, 2857, 3106N, 3398, 3483, 3970, 4769, 6392, 6962, 7028, 8854, 8860, 10310, 10609, 11719, 12406, 12882, 13398, 13928C, 14766, 15326, 15442, 16183C, 16189, 16304, 16519

K38316	Tib_11	Shannan	M62b1a1a	73, 143, 150, 203, 204, 263, 309+C, 315+C, 489, 750, 1438, 2523, 2557, 2625, 2706, 2735, 3106N, 3511, 4200, 4561, 4769, 7028, 7664, 7844, 8149, 8281-8289d, 8701, 8860, 9540, 10398, 10400, 10873, 10978, 11431, 11719, 12705, 13708, 14766, 14783, 15043, 15301, 15326, 15510, 15520, 15629, 15721, 16169, 16223, 16260, 16295, 16519
K38128	Tib_11	Shannan	D4j1a1f	73, 263, 309+C, 315+C, 489, 750, 1438, 2706, 3010, 3106N, 4769, 4883, 5178A, 5262, 7028, 7581, 7783, 8414, 8701, 8860, 9053, 9540, 10398, 10400, 10873, 11696, 11719, 12130, 12358, 12705, 14668, 14766, 14783, 15043, 15295, 15301, 15326, 16086, 16223, 16362
K38725	Tib_11	Shannan	F1b1	73, 146, 249d, 263, 310, 523-524d, 750, 1438, 1943, 2706, 3106N, 3970, 4732, 4769, 5147, 6392, 6962, 7028, 7598, 8860, 10310, 10609, 10976, 11150, 11719, 12406, 12633, 12882, 13928C, 14476, 14766, 15326, 15437, 15479, 16183C, 16189, 16232A, 16249, 16304, 16311
K38307	Tib_11	Shannan	G1a1	73, 150, 263, 309+C, 315+C, 489, 709, 750, 1438, 2706, 3106N, 4769, 4833, 5108, 7028, 7867, 8200, 8654, 8701, 8860, 9540, 10398, 10400, 10873, 11719, 11908, 12705, 14569, 14766, 14783, 15043, 15301, 15323, 15326, 15497, 15745A, 15860, 16223, 16325, 16362, 16519
K38783	Tib_11	Shannan	M9a1b1c	73, 150, 152, 153, 263, 310, 489, 709, 750, 1041, 1438, , 2706, 3106N, 3394, 4491, 4769, 7028, 8701, 8860, 9000, 9540, 10398, 10400, 10454, 10873, 11719, 12351, 12362, 12705, 14308, 14766, 14783, 15043, 15301, 15326, 15671, 16158, 16223, 16234, 16288, 16362, 16519
K38156	Tib_11	Shannan	M9a1b1c	73, 150, 152, 153, 263, 309+C, 315+C, 489, 750, 1041, 1438, 2706, 3106N, 3394, 4491, 4769, 7028, 8701, 8860, 9540, 10398, 10400, 10454, 10873, 11719, 12362, 12705, 14308, 14766, 14783, 15043, 15301, 15326, 15671, 16158, 16172, 16223, 16234, 16362, 16519
K38098	Tib_11	Shannan	M9a1b1c	73, 150, 152, 153, 183, 263, 310, 489, 750, 1041, 1438, 2706, 3106N, 3394, 4491, 4769, 7028, 8701, 8860, 9540, 10398, 10400, 10454, 10873, 11719, 12362, 12705, 14308, 14766, 14783, 15043, 15301, 15326, 15671, 16158, 16223, 16234, 16362, 16519
K38663	Tib_11	Shannan	M9a1a1c1b1a	73, 263, 309+CC, 315+C, 489, 711, 750, , 1041, 1438, , , , 2706, 3106N, 3394, 4491, 4769, 5899+XC, 7028, 7142, 7697, 8701, 8860, 9242, 9540, 10398, 10400, 10873, 11719, 12705, 14308, 14417, 14766, 14783, 15043, 15301, 15326, 16051, 16223, 16234, 16316, 16362
K38216	Tib_11	Shannan	M9a1a1c1b1a	73, 263, 309+C, 315+C, 489, 711, 750, 1041, 1438, 2706, 3106N, 3394, 4491, 4769, 5899+XC, 7028, 7142, 7595, 7697, 8701, 8860, 9242, 9540, 10398, 10400, 10873, 11719, 12705, 14308, 14417, 14766, 14783, 15043, 15301, 15326, 16223, 16234, 16316, 16362
K38851	Tib_11	Shannan	A11a1a	73, 152, 235, 263, 309+C, 315+C, 523-524d, 663, 750, 1005, 1438, 1736, 2706, 3106N, 4248, 4769, 4824, 5899+XC, 6755, 7028, 8794, 8843, 8860, 9103, 9650, 10978, 11719, 12705, 14766, 15326, 15470, 16093TC, 16223, 16243, 16290, 16293C, 16319, 16519
K38528	Tib_11	Shannan	B4d4	73, 150, 263, 309+C, 315+C, 750, 827, 1438, 2706, 2755, 3106N, 4769, 5372, 6324C, 7028, 8281-8289d, 8860, 9306TC, 11719, 11914, 13359, 13942, 14766, 15326, 15535, 15930, 16183C, 16189, 16217, 16234, 16324, 16519
K38761	Tib_11	Shannan	B5b	73, 263, 309+C, 315+C, 523-524d, 709, 750, 960+XC, 1438, 1598, 2706, 2757, 3106N, 4418, 4769, 6101, 6719, 7028, 8281-8289d, 8584, 8784, 8829, 8860, 9950, 10398, 11719, 12361, 14384, 14527, 14766, 15077, 15223, 15326, 15508, 15662, 15851, 15927, 16140, 16182C, 16183C, 16189, 16243, 16519
K38849	Tib_11	Shannan	C7b	73, 249d, 263, 310, 489, 750, 1438, 2706, 3106N, 3552A, 4715, 4769, 5821, 5918, 6338, 7028, 7196A, 8584, 8701, 8860, 9540, 9545, 10398, 10400, 10873, 11101, 11719, 11914, 12705, 13263, 14318, 14766, 14783, 15301, 15326, 15487T, 15928, 16037, 16051, 16183C, 16189, 16223, 16278, 16298, 16327, 16519
K38756	Tib_11	Shannan	D4b2b8	73, 263, 310, 390, 489, 523-524d, 750, 1382C, 1438, 2706, 3010, 3106N, 3398, 4769, 4883, 5178A, 7028, 8020, 8414, 8701, 8860, 8964, 9296, 9540, 9824A, 10398, 10400, 10873, 11719, 12705, 14668, 14755, 14766, 14783, 15001, 15043, 15236, 15301, 15326, 15613, 16223, 16362, 16519
K38488	Tib_11	Shannan	D4g2a1c1	73, 263, 298, 309+C, 315+C, 489, 750, 1438, 2706, 3010, 3106N, 4394, 4769, 4883, 5178A, 5231, 7028, 8414, 8701, 8860, 9540, 10398, 10400, 10873, 11059, 11150, 11719, 12705, 12966A, 13104, 14668, 14766, 14783, 15043, 15301, 15326, 16223, 16240, 16274, 16362, 16519
K38274	Tib_11	Shannan	D4j1a1d	73, 195, 263, 310, 489, 750, 1438, 2706, 3010, 3106N, 4769, 4883, 5178A, 5262, 7028, 7581, 7783, 8414, 8701, 8860, 9540, 10398, 10400, 10873, 11696, 11719, 12130, 12358, 12705, 14668, 14766, 14783, 15043, 15295, 15301, 15326, 16086, 16223, 16274, 16362, 16519
K38410	Tib_11	Shannan	D5a2a	73, 150, 263, 310, 489, 523-524d, 750, 752, 1107, 2706, 3106N, 4769, 4883, 5178A, 5301, 7028, 8701, 8860, 9180, 9540, 10397, 10398, 10400, 10873, 11719, 11944, 12026, 12705, 14766, 14783, 15043, 15301, 15326, 16092, 16182C, 16183C, 16189, 16213, 16223, 16266, 16362
K38768	Tib_11	Shannan	F1c1a1a1	73, 152, 249d, 263, 310, 523-524d, 709, 750, 1438, 2706, 3106N, 3970, 4769, 6392, 6599, 6962, 7028, 8860, 9053, 9647, 10310, 10454, 10609, 11719, 12406, 12882, 13759, 13928C, 14766, 15244, 15326, 16111, 16129, 16304, 16519
K38741	Tib_11	Shannan	F1d	73, 146, 249d, 263, 309+CC, 315+C, 523-524d, 750, 1438, 1734, 2706, 3106N, 3970, 4769, 5460, 5628, 5794, 6392, 6962, 7028, 7738, 8410, 8860, 10310, 10609, 11092, 11719, 12406, 12882, 13928C, 14766, 15326, 15402, 16304, 16519
K38580	Tib_11	Shannan	F1d1a2a1	73, 146, 234, 249d, 263, 309+CC, 315+C, 523-524d, 750, 1438, 1734, 1888, 2706, 3106N, 3970, 4769, 5628, 6392, 6962, 7028, 7738, 8167, 8860, 10310, 10609, 11719, 12372, 12406, 12882, 13135, 13928C, 14766, 15326, 15402, 16145, 16189, 16193+CC, 16255, 16284, 16304, 16519

K38554	Tib_11	Shannan	F1g	73, 249d, 263, 309+CC, 315+C, 523-524d, 750, 1438, 2389, 2706, 3106N, 3398, 3421, 3970, 4769, 6023, 6392, 6962, 7028, 8860, 10310, 10609, 11719, 12406, 12882, 13928C, 14766, 15326, 16182C, 16183C, 16189, 16304, 16519
K38616	Tib_11	Shannan	G2b1b	73, 263, 309+C, 315+C, 489, 709, 750, 1438, 2706, 3106N, 4769, 4833, 4853, 5108, 5601, 7028, 8701, 8860, 8877, 9540, 10398, 10400, 10873, 11719, 12375, 12705, 13563, 14569, 14766, 14783, 15043, 15301, 15326, 16223, 16362
K38423	Tib_11	Shannan	G3a1a1a	16T, 73, 143, 150, 263, 309+C, 315+C, 489, 709, 750, 1438, 2706, 3106N, 4674, 4769, 4833, 5108, 7028, 8701, 8860, 8861, 9540, 10398, 10400, 10873, 11719, 11914, 12612, 12705, 14569, 14766, 14783, 15043, 15301, 15326, 15746, 16215, 16223, 16274
K38850	Tib_11	Shannan	M10a1b	73, 263, 310, 489, 573+XC, 709, 750, 1438, 2706, 3106N, 3172+C, 4140, 4769, 7028, 7250, 8701, 8793, 8856, 8860, 9111, 9540, 10398, 10400, 10646, 10873, 11719, 12549, 12705, 13152, 14502, 14766, 14783, 15040, 15043, 15071, 15218, 15301, 15326, 15913, 16066, 16223, 16257, 16311
K38282	Tib_11	Shannan	M61a	73, 152, 263, 309+C, 315+C, 489, 750, 980, 1193, 1342, 1438, 2706, 3106N, 3438, 4769, 5582C, 6253, 7028, 8269, 8701, 8790, 8860, 9540, 10398, 10400, 10873, 11719, 11810, 12705, 12732, 14766, 14783, 15043, 15301, 15326, 16093, 16223, 16270, 16362, 16381, 16519
K38588	Tib_11	Shannan	M62b2	73, 150, 178, 198, 204, 263, 310, 489, 750, 1438, 2706, 2735, 3106N, 3511, 3693, 4561, 4769, 6305, 7028, 7664, 8149, 8701, 8860, 9540, 10398, 10400, 10873, 11719, 12705, 13708, 14766, 14783, 15043, 15301, 15326, 15510, 15520, 15629, 15721, 16179, 16223, 16260, 16519
K38471	Tib_11	Shannan	M62b2	73, 150, 178, 194, 204, 263, 309+CC, 315+C, 489, 750, 1243, 1438, 2706, 2735, 3106N, 3511, 3693, 4561, 4769, 6305, 7028, 7664, 8149, 8701, 8860, 9540, 10398, 10400, 10873, 11719, 12705, 13708, 14766, 14783, 15043, 15301, 15326, 15510, 15520, 15629, 15721, 16223, 16260, 16295, 16519
K38695	Tib_11	Shannan	M9a1a1c1b1a	73, 263, 309+C, 315+C, 489, 711, 750, 1041, 1438, 2706, 3106N, 3394, 4123, 4491, 4769, 5899+XC, 7028, 7142, 7697, 8701, 8860, 9242, 9540, 10398, 10400, 10873, 11719, 12705, 13830, 14308, 14417, 14766, 14783, 15043, 15301, 15326, 16223, 16234, 16316, 16362, 16519
K38490	Tib_11	Shannan	M9a1a1c1b1a	73, 263, 309+C, 315+C, 489, 711, 750, 1041, 1438, 2706, 3106N, 3335, 3394, 4491, 4769, 5899+XC, 7028, 7142, 7697, 8251, 8701, 8860, 9242, 9540, 10398, 10400, 10873, 11719, 12705, 13899, 14308, 14417, 14766, 14783, 15043, 15232, 15301, 15326, 16223, 16234, 16316, 16362
K38377	Tib_11	Shannan	M9a1a1c1b1a	73, 263, 309+C, 315+C, 453, 489, 711, 750, 1041, 1438, 2706, 3106N, 3394, 4491, 4769, 5899+XC, 7028, 7142, 7697, 8701, 8860, 9242, 9540, 10398, 10400, 10873, 11719, 12705, 14308, 14417, 14766, 14783, 15043, 15301, 15326, 16223, 16234, 16316, 16362
K38805	Tib_11	Shannan	M9a1a1c1b1a2	73, 146, 263, 297, 310, 489, 711, 750, 1041, 1438, 2706, 3106N, 3394, 3438, 4491, 4769, 5899+XC, 6446, 7028, 7142, 7697, 8701, 8860, 9242, 9540, 10398, 10400, 10873, 11719, 12705, 14308, 14417, 14766, 14783, 15043, 15301, 15326, 16223, 16234, 16316, 16362
K38788	Tib_11	Shannan	M9a1a1c1b1a2	73, 146, 263, 309+CC, 315+C, 489, 711, 750, 1041, 1438, 2706, 3106N, 3394, 4136, 4491, 4769, 5899+XC, 6446, 7028, 7142, 7697, 8701, 8860, 9242, 9540, 10398, 10400, 10873, 11719, 12705, 14308, 14417, 14766, 14783, 15043, 15301, 15326, 16223, 16234, 16316, 16362
K38562	Tib_11	Shannan	M9a1a2a1b	73, 153, 207, 263, 309+C, 315+C, 489, 513, 750, 1041, 1438, 2706, 3106N, 3394, 4491, 4769, 7028, 7256, 8701, 8860, 9540, 10398, 10400, 10873, 11719, 12705, 14142A, 14308, 14766, 14783, 15043, 15301, 15326, 16145, 16223, 16234, 16316
K38696	Tib_11	Shannan	N9a5	73, 150, 263, 309+C, 315+C, 750, 1438, 2706, 3106N, 3729, 4227, 4769, 5231, 5417, 7028, 8860, 11719, 12358, 12372, 12705, 14766, 15184, 15326, 15883, 15944d, 16172, 16209, 16223, 16257G
K38684	Tib_11	Shannan	U1a1c1a	73, 263, 285, 310d, 750, 1438, 2218, 2706, 3106N, 4769, 4991, 6026, 7028, 7581, 8766, 8860, 11467, 11719, 12308, 12372, 12879, 13104, 14070, 14364, 14766, 15115, 15148, 15217, 15326, 15954C, 16182C, 16183C, 16189, 16249, 16465, 16519
K38531	Tib_11	Shannan	Z7	73, 152, 249d, 263, 310, 489, 523-524d, 750, 1438, 2352, 2706, 2780, 3106N, 4363, 4715, 4769, 4841, 6752, 7028, 7196A, 8584, 8598, 8701, 8860, 9090, 9214, 9540, 10398, 10400, 10653, 10873, 11719, 12705, 14766, 14783, 15043, 15301, 15326, 15487T, 15784, 16185, 16223, 16260, 16298, 16519
K38840	Tib_11	Shannan	M13a1b	73, 152, 263, 310, 489, 750, 1438, 1555, 2706, 3106N, 3644, 4769, 5773, 6023, 6253, 6620, 7028, 7746, 8701, 8860, 9540, 9983, 10398, 10400, 10411, 10790, 10873, 10978, 11719, 12705, 13135, 13236, 14766, 14783, 15043, 15301, 15326, 15924, 16145, 16148, 16188, 16189, 16223, 16244, 16381
K38730	Tib_11	Shannan	D4h1c2a	73, 263, 310, 489, 709, 750, 1438, 2706, 3010, 3106N, 3336, 3644, 4182, 4769, 4883, 5048, 5178A, 5899+XC, 6881, 7028, 7055, 7181, 7673, 8179, 8414, 8701, 8860, 9540, 10398, 10400, 10873, 11719, 12705, 13914A, 14162, 14668, 14766, 14783, 15043, 15301, 15326, 15924, 16174, 16223, 16311, 16362
K38686	Tib_11	Shannan	G2b2a	73, 183, 263, 309+CC, 315+C, 489, 709, 750, 1438, 1692, 2706, 3106N, 3796, 4216, 4680A, 4769, 4833, 5108, 5601, 6116, 6932, 7028, 8701, 8860, 8877, 9540, 10398, 10400, 10873, 11719, 12127, 12630, 12705, 12715, 13563, 14569, 14766, 14783, 15043, 15301, 15326, 15367, 16223, 16294, 16362
K38662	Tib_11	Shannan	M13a1b	73, 152, 195TC, 263, 310, 489, 750, 1438, 2706, 3106N, 3644, 4769, 5773, 6023, 6253, 6620, 7028, 8701, 8860, 9540, 10398, 10400, 10411, 10790, 10873, 11719, 12705, 13135, 14766, 14783, 15043, 15301, 15326, 15924, 16145, 16148, 16188, 16189, 16223, 16381, 16519

K38625	Tib_11	Shannan	M9a1a1c1b1a	73, 263, 309+C, 315+C, 489, 711, 750, 1041, 1438, 2706, 3106N, 3394, 4491, 4769, 5899+XC, 7028, 7142, 7697, 8701, 8860, 9242, 9540, 10398, 10400, 10873, 11719, 12705, 14308, 14417, 14766, 14783, 15043, 15301, 15326, 16223, 16234, 16316, 16362
K38533	Tib_11	Shannan	D4j1b	73, 263, 309+C, 315+C, 489, 750, 1438, 1734, 2706, 3010, 3106N, 4639, 4769, 4883, 5178A, 5262, 7028, 7055, 8414, 8701, 8860, 9428, 9540, 10398, 10400, 10873, 11696, 11719, 12705, 14552, 14668, 14766, 14783, 15043, 15301, 15326, 16223, 16362
K38529	Tib_11	Shannan	Z7	73, 152, 249d, 263, 310, 489, 523-524d, 750, 1438, 2352, 2706, 2780, 3106N, 4363, 4715, 4769, 4841, 6752, 7028, 7196A, 8584, 8598, 8701, 8860, 9090, 9214, 9540, 10398, 10400, 10653, 10873, 11719, 12705, 14766, 14783, 15043, 15301, 15326, 15487T, 15784, 16185, 16223, 16260, 16298, 16519
K38495	Tib_11	Shannan	G3a2	73, 143, 152, 195, 309+CC, 315+C, 489, 709, 750, 1438, 2706, 3106N, 4769, 4833, 5108, 7028, 7621, 8701, 8860, 9540, 10398, 10400, 10873, 11719, 12705, 14569, 14766, 14783, 15043, 15301, 15326, 15479, 15746, 16186, 16223, 16274, 16362
K38491	Tib_11	Shannan	M62b1b	73, 150, 203, 204, 263, 309+C, 315+C, 489, 750, 1438, 2706, 2735, 3030, 3106N, 3511, 4561, 4769, 7028, 7664, 8149, 8701, 8860, 9540, 10398, 10400, 10410, 10873, 11719, 12705, 13708, 14766, 14783, 15043, 15301, 15326, 15510, 15520, 15629, 15721, 16183C, 16189, 16223, 16260, 16295, 16519
K38457	Tib_11	Shannan	M13a1b	73, 152, 263, 310, 489, 750, 1438, 2706, 3106N, 3644, 4769, 5773, 6023, 6253, 6599, 6620, 7028, 8701, 8860, 9540, 10398, 10400, 10411, 10790, 10873, 11719, 12705, 13135, 14766, 14783, 15043, 15301, 15326, 15924, 16145, 16148, 16188, 16189, 16223, 16381
11518	Han	Shannxi	A11a2	73, 152, 263, 315+C, 523-524d, 663, 750, 1438, 1736, 2706, 4248, 4769, 4824, 7028, 8581, 8794, 8843, 8860, 9650, 11087, 11719, 12705, 14766, 15326, 15397, 16092, 16223, 16290, 16293C, 16319
7588	Han	Liaoning	A11a	73, 152, 235, 263, 315+CC, 523-524d, 663, 750, 1438, 1736, 2706, 4140A, 4248, 4769, 4824, 7028, 8794, 8843, 8860, 9632, 9650, 11719, 12705, 14674, 14766, 15326, 16093, 16223, 16263, 16290, 16293C, 16319, 16519
HN-SZ 517	Han	Hunan	A11a1	73, 152, 235, 263, 315+C, 523-524d, 663, 750, 1438, 1736, 2706, 4248, 4769, 4824, 5899+XC, 6755, 7028, 8794, 8843, 8860, 9650, 11719, 12705, 14766, 15326, 16223, 16290, 16291A, 16293C, 16319
Z11	Tib	Qinghai	A11a1a	73, 152, 235, 263, 309+C, 315+C, 523-524d, 663, 750, 1005, 1438, 1736, 2706, 4248, 4769, 4824, 5899+XC, 6755, 7028, 8794, 8843, 8860, 9650, 9867-10217d, 11719, 12705, 14706, 14766, 15326, 16223, 16290, 16293C, 16319
HN-SZ 59	Han	Hunan	A11a1	73, 152, 235, 263, 315+C, 523-524d, 663, 750, 1438, 1736, 2706, 4248, 4769, 4824, 5899+XC, 6755, 7028, 8794, 8843, 8860, 9650, 11719, 12705, 14766, 15326, 16223, 16291A, 16293C, 16319
Tib474	Tib	Rikaze	A11a1a	73, 152, 235, 263, 309+C, 315+C, 523-524d, 663, 750, 1005, 1393, 1438, 1736, 2706, 4248, 4769, 4824, 6755, 7028, 7774-7900d, 8794, 8840, 8843, 8860, 9650, 11719, 12705, 14766, 15326, 15381, 16093, 16126, 16172, 16223, 16290, 16293C, 16319, 16519
Tib282	Tib	Rikaze	A11a1a	73, 152, 235, 263, 309+CC, 315+C, 523-524d, 663, 750, 1005, 1344, 1438, 1736, 2706, 4248, 4769, 4824, 5899+XC, 6755, 7028, 8794, 8843, 8860, 9650, 11719, 12705, 14766, 15326, 16223, 16290, 16293C, 16319, 16359
R23	Bengali	Bengal	A11a1a	73, 152, 235, 263, 309+C, 315+C, 523-524d, 663, 750, 1005, 1438, 1736, 2706, 4248, 4769, 4824, 5899+XC, 6539, 6755, 7028, 8794, 8843, 8860, 9100, 9650, 11719, 12354, 12705, 14766, 15043, 15326, 16223, 16290, 16293C, 16319, 16519
Nep04	Nepalese	Nepal	A11a1a	73, 152, 235, 263, 309+C, 315+C, 523-524d, 663, 750, 1005, 1438, 1736, 2706, 4248, 4769, 4824, 5899+XC, 6755, 7028, 8563, 8794, 8843, 8860, 9650, 11719, 12705, 14766, 15326, 15479, 16093, 16223, 16290, 16293C, 16319
Dongxiang 63	Dongxiang	Gansu	A11a2	73, 152, 235, 263, 315+C, 523-524d, 663, 750, 1438, 1736, 2706, 4248, 4769, 4824, 7028, 8794, 8843, 8860, 9650, 11719, 12705, 13105, 14766, 15326, 15397, 16092, 16223, 16290, 16293C, 16319, 16352, 16519
K28864	Han	Liaoning	A11a2	73, 152, 263, 310, 523-524d, 663, 750, 1438, 1736, 2706, 3106N, 4248, 4769, 4824, 5899+XC, 7028, 8581, 8794, 8843, 8860, 9650, 11087, 11719, 12705, 14766, 15326, 15397, 16092, 16223, 16290, 16293C, 16319

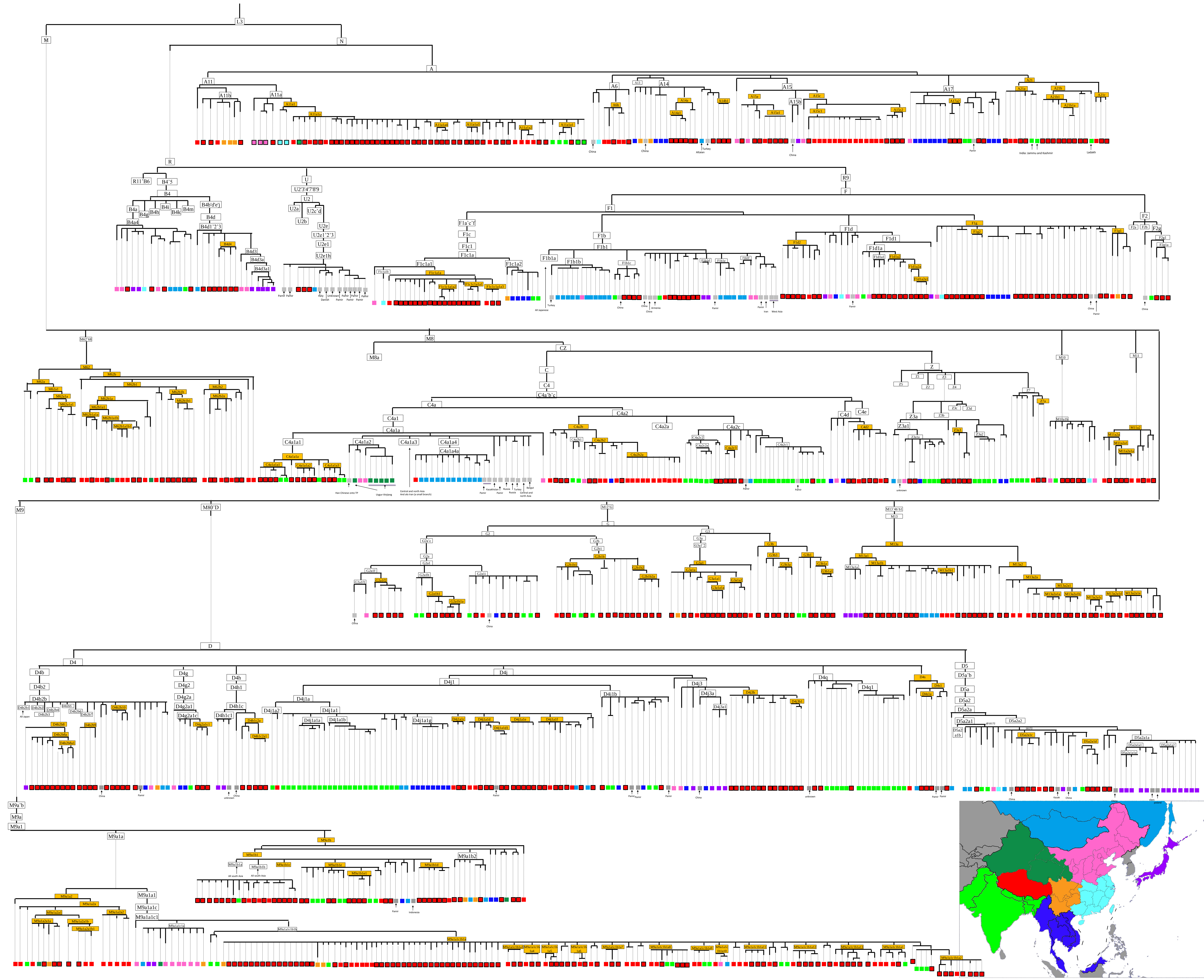

Figure S1 Schematic phylogenetic trees of Tibetan haplogroups based on whole mtDNA sequences from this study (black outline) and previous literature (no outline). Only haplogroups in more than two Tibetan samples were included in the tree. Lengths of each branch are in proportion to variants. Locations are indicated by different colors. Haplogroups with *de novo* differentiation in Tibetans are indicated in orange.

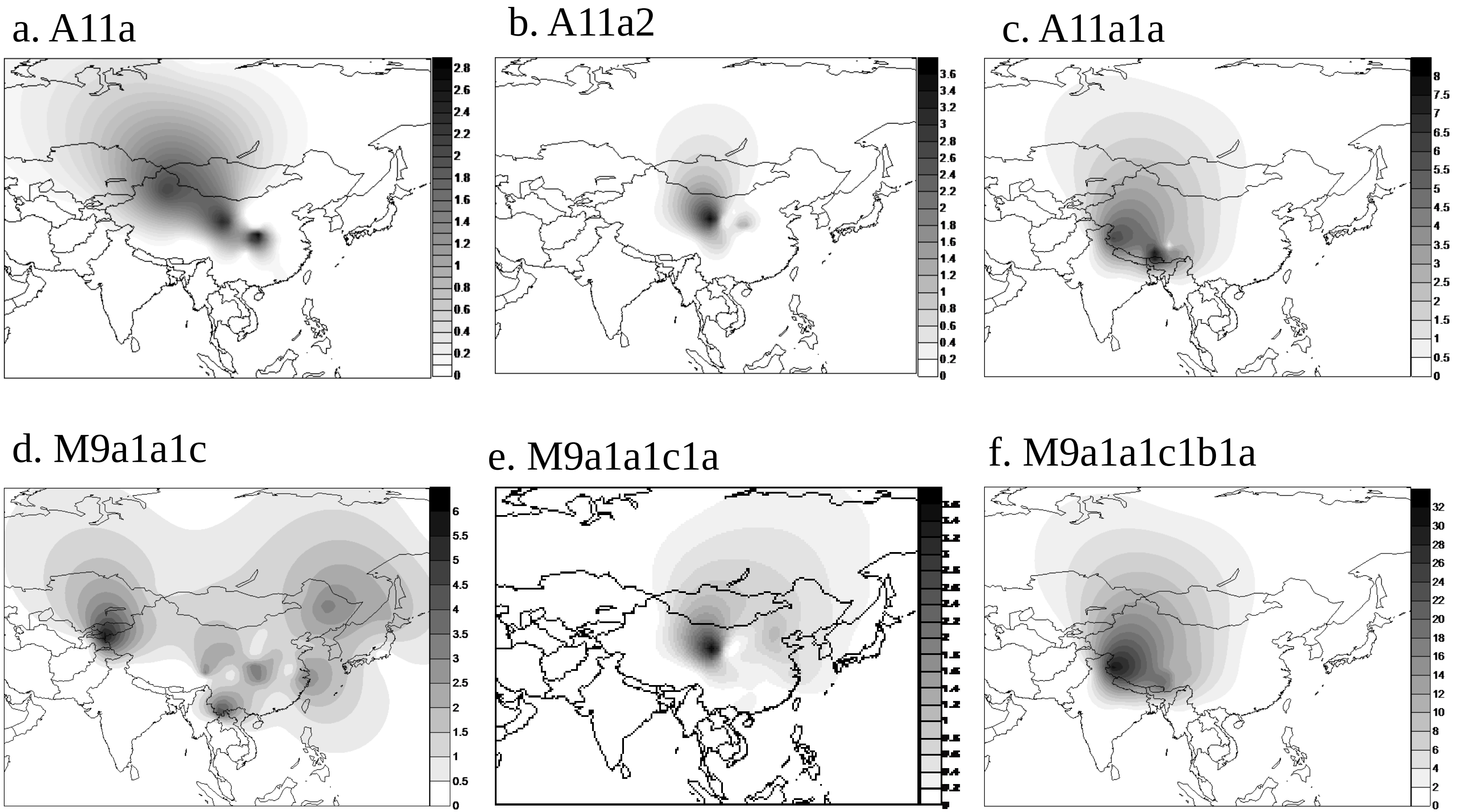

Figure S2. Contour maps of A11a and M9a1a1c1 and their sub-lineages (A11a2, A11a1a, M9a1a1c1a, and M9a1a1c1b1a) based on the HVS dataset (Table S3).

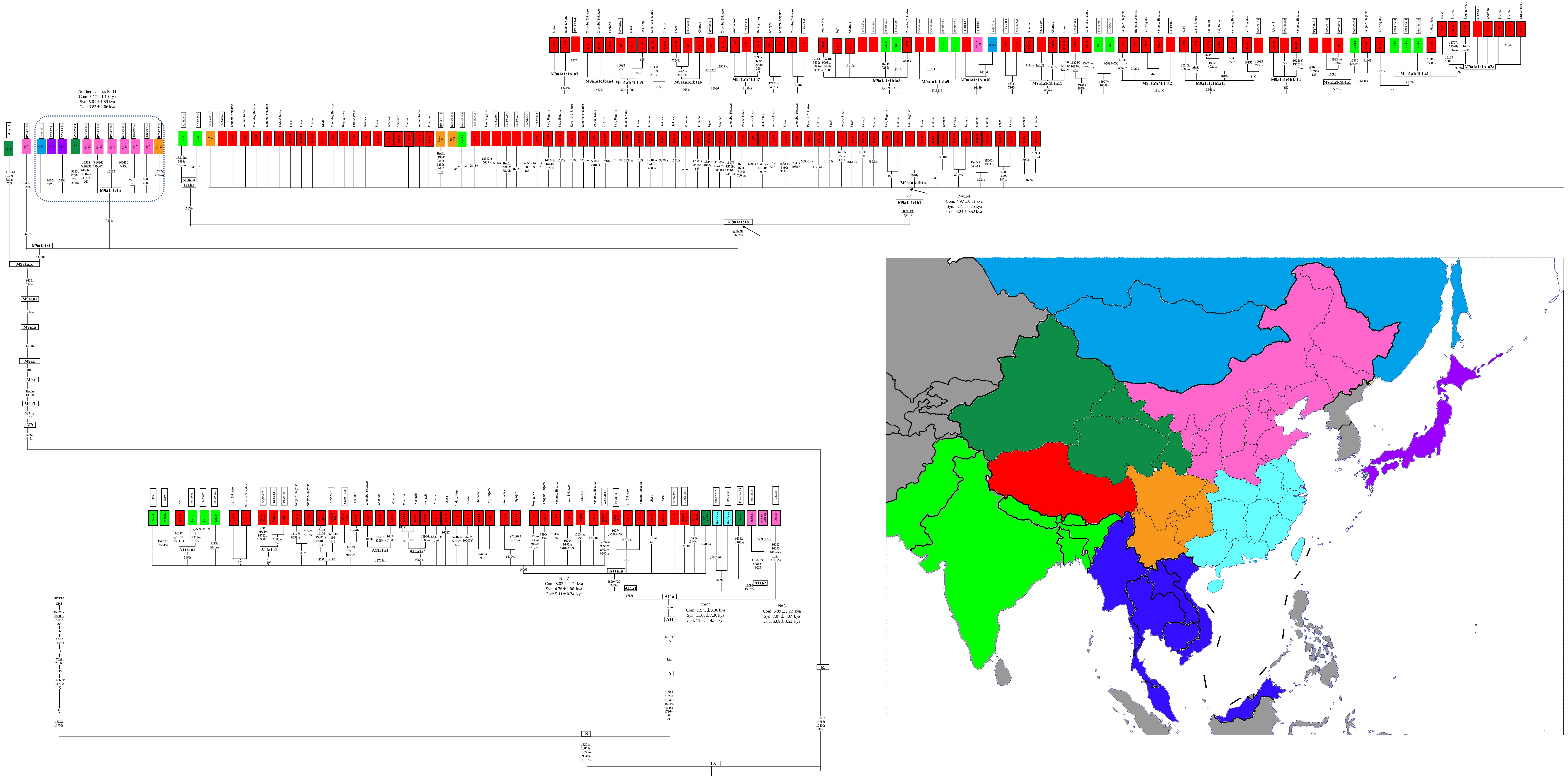

Figure S3. Phylogenetic trees of A11a1a and M9a1a1c1b1a. Nucleotide position numbers are consistent with the revised Cambridge reference sequence (rCRS), Suffixes C, G, and T refer to transversions; ‘d’, deletion; ‘s’, synonymous mutation; ‘ns’, non-synonymous mutation; ‘+’, insertion; recurrent mutations are underlined; ‘@’, reverse mutation; ‘h’, heterogeneity; ‘t’, change in transfer RNA; ‘r’, change in ribosomal RNA gene; nc, mutations at intergenic noncoding regions in segments 577–16023. Com, syn, and cod: coalescent age calculated based on complete genome substitutions, coding region synonymous substitutions, and coding region variants, respectively.

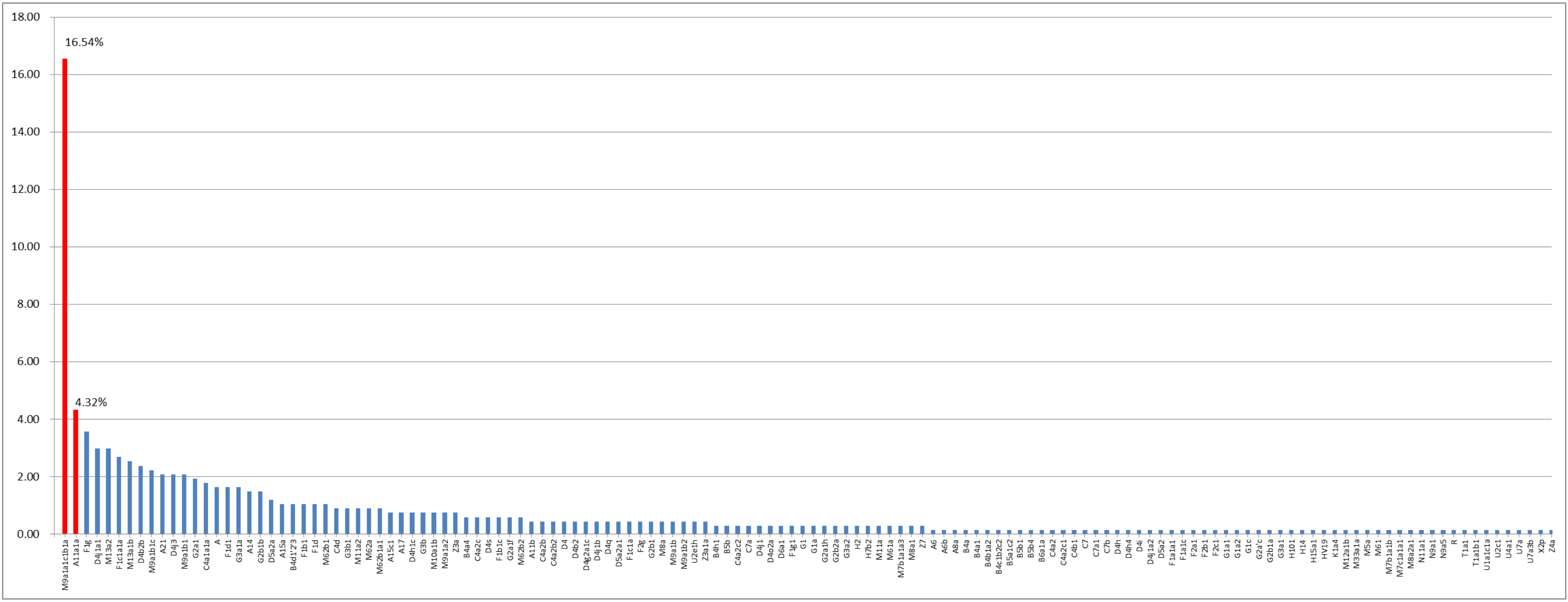

Figure S4. Frequencies of haplogroups in Tibetans. Haplogroups M9a1a1c1b1a and A11a1a are indicated in red, with remaining haplogroups indicted in blue.
